# Genomic innovations, transcriptional plasticity and gene loss underlying the evolution and divergence of two highly polyphagous and invasive *Helicoverpa* pest species

**DOI:** 10.1186/s12915-017-0402-6

**Published:** 2017-07-31

**Authors:** S. L. Pearce, D. F. Clarke, P. D. East, S. Elfekih, K. H. J. Gordon, L. S. Jermiin, A. McGaughran, J. G. Oakeshott, A. Papanikolaou, O. P. Perera, R. V. Rane, S. Richards, W. T. Tay, T. K. Walsh, A. Anderson, C. J. Anderson, S. Asgari, P. G. Board, A. Bretschneider, P. M. Campbell, T. Chertemps, J. T. Christeller, C. W. Coppin, S. J. Downes, G. Duan, C. A. Farnsworth, R. T. Good, L. B. Han, Y. C. Han, K. Hatje, I. Horne, Y. P Huang, D. S. T. Hughes, E. Jacquin-Joly, W. James, S. Jhangiani, M. Kollmar, S. S. Kuwar, S. Li, N-Y. Liu, M. T. Maibeche, J. R. Miller, N. Montagne, T. Perry, J. Qu, S. V. Song, G. G. Sutton, H. Vogel, B. P. Walenz, W. Xu, H-J. Zhang, Z. Zou, P. Batterham, O. R. Edwards, R. Feyereisen, R. A. Gibbs, D. G. Heckel, A. McGrath, C. Robin, S. E. Scherer, K. C. Worley, Y. D. Wu

**Affiliations:** 1 CSIRO Black Mountain, GPO Box 1700, Canberra, ACT 2600 Australia; 2 School of Biological Sciences, University of Melbourne, Parkville, Vic Australia; 3 Research School of Biology, Australian National University, Canberra, ACT Australia; 4 Hawksbury Institute for the Environment, Western Sydney University, Penrith, NSW Australia; 5 Southern Insect Management Research Unit, USDA-ARS, Stoneville, MS USA; 6 Human Genome Sequencing Center, Baylor College of Medicine, Houston, TX USA; 7 Biological and Environmental Sciences, University of Stirling, Stirling, UK; 8 School of Biological Sciences, University of Queensland, Brisbane St Lucia, QLD Australia; 9 John Curtin School of Medical Research, Australian National University, Canberra, ACT Australia; 10 Max Planck Institute of Chemical Ecology, Jena, Germany; 11 Sorbonnes Universités, UPMC Université Paris 06, Institute of Ecology and Environmental Sciences of Paris, Paris, France; 12 National Institute for Agricultural Research (INRA), Institute of Ecology and Environmental Sciences of Paris, Versailles, France; 13 Plant and Food Research, Mt Albert, Auckland New Zealand; 14 CSIRO, Narrabri, NSW Australia; 15 State Key Laboratory of Integrated Management of Pest Insects and Rodents, Institute of Zoology, Chinese Academy of Sciences, Beijing, 100101 China; 16 College of Plant Protection, Nanjing Agricultural University, Nanjing, Jiangsu China; 17 Max Planck Institute for Biophysical Chemistry, Gottingen, Germany; 18 Institute of Plant Physiology and Ecology, Shanghai Institutes of Biological Sciences, Chinese Academy of Sciences, Shanghai, China; 19 Key Laboratory of Forest Disaster Warning and Control of Yunnan Province, Southwest Forestry University, Kunming, 650224 China; 20 J. Craig Venter Institute, Rockville, MD USA; 21 School of Veterinary and Life Sciences, Murdoch University, Perth, WA Australia; 22 Chongqing Key Laboratory of Biochemistry and Molecular Pharmacology, Chongqing Medical University, Chongqing, 400016 China; 23 CSIRO, Floreat Park, WA Australia; 24 Department of Plant and Environmental Sciences, University of Copenhagen, Thorvaldsensvej, Denmark

## Abstract

**Background:**

*Helicoverpa armigera* and *Helicoverpa zea* are major caterpillar pests of Old and New World agriculture, respectively. Both, particularly *H. armigera,* are extremely polyphagous, and *H. armigera* has developed resistance to many insecticides. Here we use comparative genomics, transcriptomics and resequencing to elucidate the genetic basis for their properties as pests.

**Results:**

We find that, prior to their divergence about 1.5 Mya, the *H. armigera*/*H. zea* lineage had accumulated up to more than 100 more members of specific detoxification and digestion gene families and more than 100 extra gustatory receptor genes, compared to other lepidopterans with narrower host ranges. The two genomes remain very similar in gene content and order, but *H. armigera* is more polymorphic overall, and *H. zea* has lost several detoxification genes, as well as about 50 gustatory receptor genes*.* It also lacks certain genes and alleles conferring insecticide resistance found in *H. armigera.* Non-synonymous sites in the expanded gene families above are rapidly diverging, both between paralogues and between orthologues in the two species. Whole genome transcriptomic analyses of *H. armigera* larvae show widely divergent responses to different host plants, including responses among many of the duplicated detoxification and digestion genes.

**Conclusions:**

The extreme polyphagy of the two heliothines is associated with extensive amplification and neofunctionalisation of genes involved in host finding and use, coupled with versatile transcriptional responses on different hosts. *H. armigera’s* invasion of the Americas in recent years means that hybridisation could generate populations that are both locally adapted and insecticide resistant.

**Electronic supplementary material:**

The online version of this article (doi:10.1186/s12915-017-0402-6) contains supplementary material, which is available to authorized users.

## Background

A major question in evolutionary biology that becomes tractable with the advent of modern genomics is the genetic basis for the transitions between broad ‘generalist’ and narrow ‘specialist’ ecological niches [1–3]. Emerging empirical evidence suggests that the transition to specialism often involves a loss of function due to a loss of genetic material (deletions or *pseudogenisation* [4, 5]). However, there is less evidence, and little consensus, on how the gains of function presumptively underlying the evolution of generalism have been achieved at the genomic level. One of the two major mechanisms proposed attributes the acquisition of new functions to gene duplication followed by subfunctionalisation and then neofunctionalisation [6, 7], while the other invokes the development of more versatile regulatory networks and transcriptional responses to different environments [8–10]. The host range of herbivorous insects is a useful model to investigate this issue because many of the molecular systems associated with host finding and the digestion and detoxification of host resources have been identified [11]. Here we investigate this system in two ‘megapest’ species of caterpillars [12, 13] which have considerably broader host ranges than any of the other lepidopterans so far studied at the genomic level.

The closely related noctuid moths *Helicoverpa armigera* and *Helicoverpa zea* (commonly known as the cotton bollworm and corn earworm, respectively) have been major pests of modern agriculture in the Old and New World, respectively. In the last decade, however, *H. armigera* has also invaded the New World, firstly in South America [14, 15], probably as a result of international trade [16], but then spreading rapidly into Central America [17, 18] and, most recently, North America [18, 19]. In Brazil, it appears that it has now largely displaced *H. zea* [20, 21]*.* The costs of lost production and control for *H. armigera* in the Old World alone are conservatively estimated at more than $US 5 billion annually [22], while damages to Brazil’s 2012–2013 cropping season were estimated at between $US 0.8 to 2 billion [21].

*Helicoverpa zea* and *H. armigera* are morphologically similar [23, 24] and are believed to have diverged around 1.5 Mya as the result of a founder event establishing the former in the Americas [25, 26]. Nevertheless, two observations suggest important ecological differences between the two species which greatly affect their properties as pests. Firstly, *H. armigera* has been found on more than 300 host plants across 68 families (monocots as well as dicots: http://www.cabi.org/isc/datasheet/26757) around the world, including major crops such as cotton, soy, maize and a wide range of horticultural crops, whereas *H. zea* has been recorded from a more limited number of hosts, 123 species in 29 families, albeit still including major crops such as corn and soybean [27]. Secondly, *H. armigera* has demonstrated a great propensity to evolve resistance to chemical insecticides [28–30] and *Bacillus thuringiensis* (Bt)-transgenic crops [31, 32], whereas *H. zea* has remained more susceptible to major insecticides such as the pyrethroids [33, 34] and Bt crops [35, 36].

This paper explores the genomic bases for both the extreme polyphagy of the two heliothines and the differences in host range and insecticide resistance propensity between them. We find that the two genomes share very high levels of orthology, and that they both have larger complements of gene families involved in detoxification, digestion and chemosensory functions compared to other lepidopterans with more specialist feeding habits. This includes large clusters of carboxylesterases, trypsin- and chymotrypsin-like gut proteases and clusters of gustatory receptors, these clusters alone containing more than 100 additional genes. These genes are rapidly diverging from one another and show relatively high levels of polymorphism among resequenced lines of each species. Many of them prove to be differentially expressed when larvae are reared on different host plants. Thus, we find evidence that both gene duplication and neofunctionalisation as well as transcriptional versatility are associated with the species’ generalist niches. Importantly, however, we also find genomic differences between the two species which could explain their differences in host range and insecticide resistance; *H. armigera* has 50 extra gustatory receptors and several more detoxification genes, plus some genes and alleles specifically associated with resistance to major chemical and biological insecticides, that are missing in *H. zea.* Given this, plus the very high level of synteny we find between the two species’ genomes and evidence from other studies (e.g. Anderson et al. [37]) for hybridisation between them since *H. armigera* arrived in America, there is considerable scope for introgression to rapidly generate new heliothine ecotypes with novel combinations of traits relating to their pest status.

## Results and Discussion

### Genome assembly and annotation

For *H. armigera,* the final assembly freeze (‘csiro4bp’) has 997 scaffolds covering a total of 337 Mb and including 37 Mb of gaps. The N50 is 1.00 Mb, and the mean scaffold length is 338 kb (Table 1). This assembly was selected from several that were generated based on contig and scaffold length and integrity and gene assembly quality for a set of test genes. For *H. zea,* the final assembly freeze (‘csirohz5p5’) has 2975 scaffolds covering a total of 341 Mb, including 34 Mb of gaps. The N50 is 201 kb, and the mean scaffold length is 115 kb (Table 1). These overall genome sizes are very close to those previously determined by flow cytometry for these and closely related heliothine species [38]. However, they are smaller than those estimated from genome data for the original lepidopteran model genome, the silkworm *Bombyx mori* (431.7 Mb) [39] and its relative, the tobacco hornworm *Manduca sexta* (419 Mb) [40]. The N50 statistic for *H. armigera* in particular compares well to other lepidopteran draft assemblies, although the *B. mori* assembly has a significant proportion of the genome in larger scaffolds (Table 1).

**Table 1:** Genome assembly and annotation statistics

Species	*H. armigera*	*H. zea*	*B. mori* ^a^	*M. sexta* ^b^
Genome assembly	csiro4bp	csirohz5p5		
Assembly size (Mb)	337.07	341.15	431.7	419.42
Number of scaffolds	997	2975	43,622	20,870
Max. scaffold length (Mb)	6.15	1.85	16.12	3.25
N50 scaffold size (kb)	1000.4	201.5	3717.00	664.01
N90 scaffold size (kb)	175.3	52.3	43.1	46.4
Mean scaffold length (kb)	338.1	114.7	9.9	20.1
Median scaffold length (kb)	117.3	68.0	0.655	0.997
Number of contigs	24,228	34,676	88,842	38,380
N50 contig length (kb)	18.3	12.6	15.5	40.4
Mean contig length (kb)	12.4	8.6	4.86	10.4
Median contig length (kb)	7.4	5.4	NA	NA
Gene annotation			(NCBI)^c^	
Protein-coding	17,086	15,200^d^	15,007	27,404
InterPro domain	12,212	11,061	14,113	NA
GO	11,324	10,221	9462	NA
Pfam	10,700	9,795	11,753	NA
KEGG	4217	4004	6242	8611
Genomic features				
Repeat (%)	14.6	16.0	43.6	24.9
GC (%)	36.1	36.2	38.8	35.3
Coding (%)	6.7	5.9	4.1	10.4
Intron (%)	39.3	17.7	16.3	NA
Gene length (b)	9098	5306	6029	NA
Avg. protein length (aa)	442.8	444.7	458.5	531.1
microRNAs	251	232	487	98
Quality control: BUSCO % present (complete)				
Genome	94.3 (83)	93.2 (80)	91.6 (73)	93.7 (81)
Proteins (OGS)	94.6 (86)	90.7 (82)	93.6 (87)	92.9 (84)

N50 and N90 are computed on each assembly size as given in the table. The statistics for published *B. mori* and *M. sexta* genome assemblies are included for comparison, with references as follows:

^a^ *B. mori* v2 [39], ^b^ *M. sexta* [40], ^c^National Center for Biotechnology Information (NCBI) Gnomon models, ^d^Indicates plus 1192 partial gene models

*GO* Gene Ontology, *KEGG* Kyoto Encyclopedia of Genes and Genomes, *BUSCO* Benchmarking Universal Single-Copy Orthologues, *OGS* official gene set

Automated annotation of the *H. armigera* genome followed by some manual correction by domain experts (see below) yielded a final official gene set (OGS2) of 17,086 genes (Additional file 1: Table S1). This gene set was then used to derive a final OGS (OGS2) containing 15,200 good-quality gene models for *H. zea* (Additional file 1: Table S1). Orthologues of another 1192 *H. armigera* gene models were present as poor-quality models (i.e. much shorter than expected from their *H. armigera* orthologues) in the available *H. zea* assemblies and transcriptome data, making a total of 16,392 *H. armigera* genes for which orthologues could be identified in the *H. zea* genome. This left 694 *H. armigera* genes for which no *H. zea* orthologues were found. In the *H. zea* assemblies, on the other hand, 410 gene models more than 100 codons in length were identified that had no apparent *H. armigera* orthologue but these were generally incomplete models that lacked start codons. Nor could any of the very few Pfam domains that were found among the latter gene models be assigned to any of the major manually annotated gene families. These latter *H. zea* models were therefore not analysed further.

Application of the Benchmarking Universal Single-Copy Orthologues (BUSCO) pipeline [41] showed that the two *Helicoverpa* OGS2s compare well for completeness with the other lepidopteran genomes analysed. In particular, the *H. armigera* genome scored more highly on both the genome and protein analyses for genes present than do either of the well-characterised *B. mori* or *M. sexta* genomes (Table 1).

Nearly 83% (14,155) of the 17,086 genes identified in the *H. armigera* genome could be functionally annotated by searches against *B. mori* and *Drosophila melanogaster* proteome databases as matching proteins with functions described as other than “uncharacterised”. Most of these also have InterProScan domains or Gene Ontology (GO) annotations (Table 1; Additional file 2: Table S2).

**Fig. 1:**
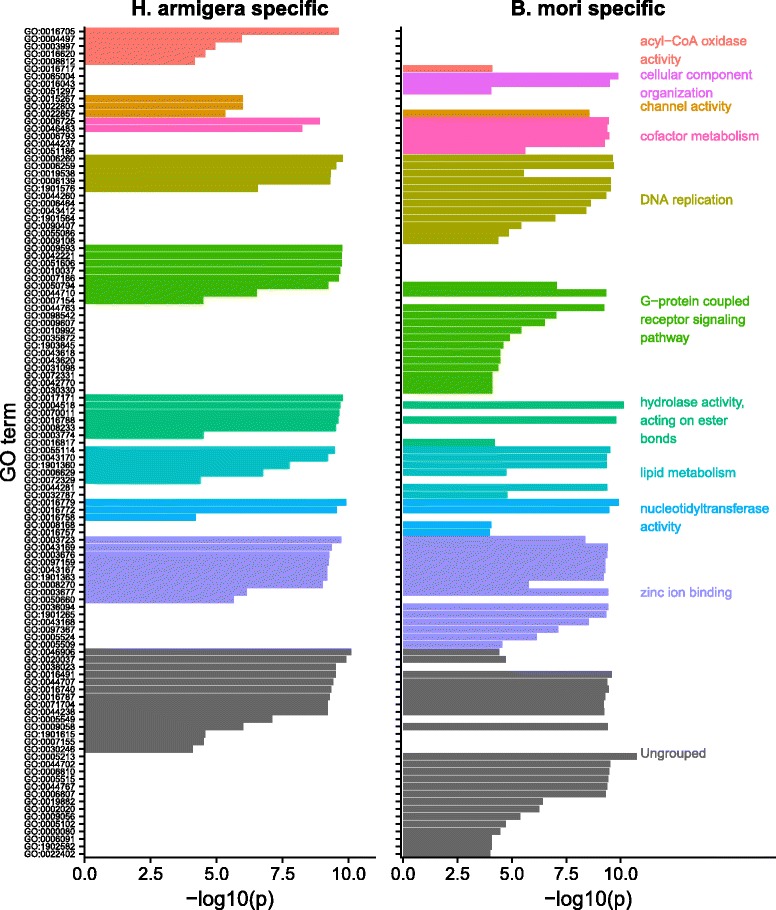
GO term analyses of gene gain/loss events in *H. armigera* vs *B. mori*. The *left panel* shows GO terms enriched in the *H. armigera* gene set vs *B. mori*, and the *right panel* shows those enriched in the *B. mori* gene set vs *H. armigera*

Orthologue mapping of the 17,086 *H. armigera* genes with the 15,007 National Center for Biotechnology Information (NCBI) Gnomon models for *B. mori* identified 10,612 direct orthologues. Of the genes in either of these species without direct orthologues in the other, 3043 of the *H. armigera* genes and 2479 of those from *B. mori* have GO annotations. For the *B. mori* genes with no *H. armigera* orthologue, the major over-represented annotations are chromatin structure and organisation, and DNA replication, with some genes also relating to chorion production (Fig. 1). In contrast, the *H. armigera* genes without known orthologues in *B. mori* are over-represented with annotations of signal transduction and sensory perception relating to taste and smell (corresponding to those terms labelled G protein coupled receptor signaling pathway), proteolysis and detoxification.

GO annotations were found for 237 of the 694 *H. armigera* genes without an identifiable match in the *H. zea* genome. The GO annotations most over-represented among these genes involved sensory perception and signal transduction of taste or smell (Additional file 3: Figure S1). Analysis of the 1192 genes present in *H. armigera* but with poor models in the *H. zea* genome showed that only those associated with retrotransposon-coding sequences were enriched; this is consistent with these genes lying in poorly assembled genomic regions rather than belonging to any biologically distinct functional group.

Using RepeatModeler, we estimated that the *H. armigera* and *H. zea* genomes contain 14.6% (49 Mb) and 16.0% (53 Mb) repeats, respectively, which was significantly less than the ~35% repetitive sequence found in the *B. mori* genome and the ~25% repetitive sequence found in the postman butterfly *Heliconius melpomene* by equivalent methods (Table 1; Additional file 4: Table S3). Most (~84%) of the repeats in both *Helicoverpa* genomes consisted of unclassified repeats, with less than 1% of each genome consisting of simple repeats or low-complexity regions. A total of 682 unique complex repeats were found in *H. armigera*, and 97 of these had similarities to Dfam hidden Markov models (HMMs) [42] from other species. In concordance with Coates et al. [38], who identified 794 transposable elements (TEs) among bacterial artificial chromosome (BAC) clones from *H. zea*, a little over half of all TEs identified were type I elements (retrotransposed) in *H. armigera* (53%) and *H. zea* (also 53%), and about half of those were long interspersed nuclear elements (LINEs) (Additional file 4: Table S3). Gypsy elements were the most numerous long terminal repeat (LTR) elements identified in both genomes, although LTR elements were less abundant in *H. zea* than in *H. armigera*, possibly reflecting poorer genome assembly quality. For both genomes, the most abundant of the type II elements (DNA transposon-like) that could be classified belonged to the hAT family.

An extensive microRNA (miRNA) catalogue (http://www.mirbase.org) has been developed for *B. mori* [43–45] and (as of August 2016) contains 563 mature miRNA sequences, the most for any insect. Two recent papers have also identified miRNAs in *H. armigera* [46, 47]. We have identified 301 potential miRNAs in *H. armigera* by combining the ones previously identified for this species and those identified through our own sequencing of small RNAs (Additional file 5: Table S4). Of these, 134 appear to be conserved (*E* value ≤ 0.001) between *H. armigera* and *B. mori*, and 251 and 232 of them, respectively, could be found in our *H. armigera* and *H. zea* assemblies, although these numbers dropped to 183 and 161, respectively, when only perfect matches were allowed. Several of the *H. armigera* and *H. zea* miRNAs occur within 1 kb of others, but there is only one cluster of more than two (*H. armigera* scaffold_103; *H. zea* scaffold_688).

### Genome organisation

We next investigated the proportion of the *H. armigera* genome showing syntenic relationships with *B. mori* chromosomes. We found that 569 *H. armigera* scaffolds (93% of the assembled genome) carried at least two contiguous *H. armigera* genes which had identifiable orthologues on the same *B. mori* chromosome, and so could be used in this analysis. Of these scaffolds, 536 only contained genes with orthologues on the same *B. mori* chromosome (Additional file 3: Figure S2). The remaining scaffolds contained two or three discrete blocks of synteny mapping to different chromosomes and may therefore represent non-syntenous relationships or misassemblies. The 536 scaffolds above represent 75.6% of the assembled genome and indicate a very high level of synteny across these two widely separated lepidopterans. This bears out the conclusions of high conservation of macro and micro synteny in Lepidoptera from other studies [48–50].

We then investigated the synteny between the two heliothine assemblies. Of the 2975 scaffolds in the considerably more fragmented *H. zea* assembly, 2367 had good-quality gene models corresponding to *H. armigera* genes. A total of 1761 of these scaffolds (83% of the assembled *H. zea* genome) each contained at least two contiguous genes forming a synteny block with an *H. armigera* scaffold (Additional file 3: Figure S2). As with the *H. armigera*/*B. mori* comparison above, most of the 1761 scaffolds (1512, covering 62% of the assembled genome) correspond to a single *H. armigera* scaffold, with the remainder (249, covering 21% of the genome) comprising multiple distinct blocks of synteny to different *H. armigera* scaffolds. As above, the latter could indicate either non-syntenous relationships or misassemblies. Notwithstanding the limitations due to the more fragmented *H. zea* genome, these analyses again indicate a high level of synteny between the species.

### Annotation of gene families related to detoxification, digestion, chemosensation and defense

The gene families involved in detoxification, digestion and chemoreception were manually checked and annotated following application of an EXONERATE-based dedicated pipeline using all available sequences and complementary DNAs (cDNAs) to augment the automatically generated models. This yielded a total of 908 *H. armigera* and 832 *H. zea* genes. Other automatically generated gene models were manually annotated as belonging to gene families concerned with stress response and immunity, as well as to cuticular protein, ribosomal protein and transcription factor families. Additional file 6: Table S5 gives the names and locations of the total of 2378 *H. armigera* and 2269 *H. zea* genes processed in these ways.

The five major detoxification gene families (cytochrome P450s (P450s), carboxyl/cholinesterases (CCEs), glutathione S-transferases (GSTs), uridine diphosphate (UDP)-glucuronosyltransferases (UGTs) and ATP-binding cassette transporters (ABCs)) are very similar in size in *H. armigera* and *H. zea* (Table 2; Additional file 4: Sections 1–5). The slightly greater numbers recovered in the former species might be due in part to the higher quality of the assembly for that species. We also compared these numbers with those obtained with the same curation pipeline for the monophagous *B. mori* and the pest species *M. sexta*, which is oligophagous on Solanaceae (see Additional file 4: Sections 1–5) and, for the P450s, CCEs and GSTs, also for another pest, the diamondback moth *Plutella xylostella*, which is oligophagous on Brassicaceae (see Additional file 4: Sections 1–3). Relatively little difference from these other species was evident for the ABCs and UGTs, but quite large differences were found for the other detoxification families. The number of genes encoding P450s, CCEs and GSTs in the two heliothines are similar to or slightly larger than those of one of the other pest species, *M. sexta*, but substantially larger than those in *B. mori* and the other pest, *P. xylostella* — twice as large in the case of the GSTs and 20–40% larger in the case of the P450s and CCEs.

**Table 2:** Detoxification, digestive and chemosensory receptor gene families

Gene family	Clan/clade/group	*H. armigera*	*H. zea*	*Ha-Hz K*_a_ */K*_s_^b^	*B. mori* ^c^	*M. sexta*
P450s	M	10	10	0.061	11	16
	2	8	8	0.029	7	8
	3	46	42	0.076	31	45
	4	50	48	0.083	30	34
	Total	114	108		79	103
CCEs	Dietary/detox^a^	71 (8)	67 (9)	0.117	52 (8)	67 (9)
	Hormone/semiochemical processing	13 (5)	13 (5)	0.071	13 (5)	16 (6)
	Neuro-developmental	13 (10)	13 (10)	0.022	13 (10)	13 (10)
	Total	97 (23)	93 (24)		78 (23)	96 (25)
GSTs	Delta/epsilon	25	24	0.124	14	16
	Sigma	11	10	0.106	2	8
	Theta	1	1	0.063	1	1
	Zeta	2	2	0	2	2
	Omega	3	3	0.047	4	4
	Total	42	40		23	31
UGTs	UGT33	22	19	0.102	13	16
	UGT40	8	7	0.116	12	9
	Other	16	16	0.114	19	19
	Total	46	42		44	44
ABCs	A	7	7	0.036	7	7
	B	11	11	0.033	9	9
	C	11	11	0.019	11	11
	G	17	17	0.009	16	16
	Other	8	8	0.007	8	11
	Total	54	54		51	54
Serine proteases: major digestive clades	Trypsins^a^	51 (15)	46 (15)	0.159	17 (6)	^d^
	Chymotrypsins^a^	49 (4)	44 (4)	0.067	28 (3)	^d^
Lipases	Acid^a^	28 (1)	28 (1)	0.117	32 (1)	^d^
	Neutral^a^	61 (10)	60 (9)	0.061	25 (2)	^d^
Chemosensory receptor proteins	GRs	213	166	0.292	69	45
	ORs	84	82	0.090	72	73
	OBPs	40	40	0.074	40	45
	CSPs	29	29	0.056	22	21

See Additional file 6: Table S5 and Additional file 4: Sections 1–8 for details of genes, functions and names in each family

^a^Catalytically inactive sequences (although not necessarily without function) in parentheses

^b^Averaged *K*_a_/*K*_s_ for orthologous members of the subfamily

^c^Figures based on the official gene sets, with further analysis as described in Additional file 4: Section 13

^d^These figures are not available in the official gene sets at the level of detail required

*GR* gustatory receptor, *OR* olfactory receptor, *OBP* odorant-binding protein, *CSP* chemosensory protein

**Fig. 2:**
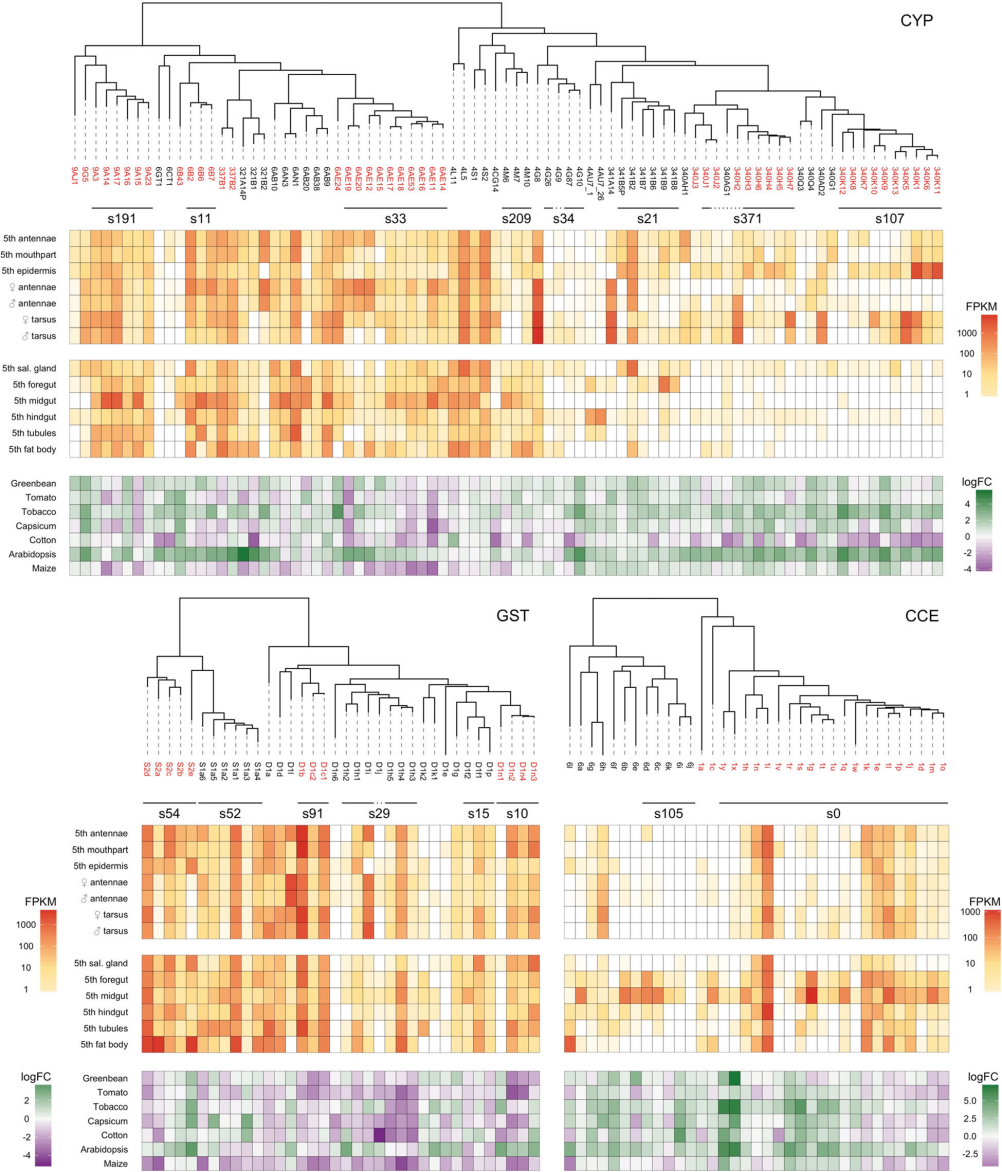
Phylogenetic, physical and transcriptional relationships within the major detoxification gene clusters. Selected clades of P450s, GSTs and CCEs, containing genes associated with detoxification functions, are shown. Clades discussed more extensively in the text are highlighted in *red*. Further details about the gene names and their associated OGS numbers are given in Additional file 4: Sections 1–3. *Bars below the gene names* indicate genes within a distinctive genomic cluster on a specific scaffold with the number shown; see Additional file 4: Sections 1–3 for further details. The clade 1 CCEs are specifically indicated. The phylogenetic order shown does not reflect the physical order of genes within a cluster. Expression is given as fragments per kilobase of transcript per million mapped reads (*FPKM*) for the tissue/developmental stage transcriptomes and log2(fold change) (*logFC*) for the host-response transcriptomes

Notably, the differences in the *H. armigera* P450s, CCEs and GSTs are largely reflected in those of their subgroups that are generally associated with xenobiotic detoxification — the P450 clans 3 and 4, the detoxification and digestive CCE clades and the GST delta and sigma classes [51–53] (Fig. 2). Of particular note is the large cluster of CCEs in clade 1, with 21 genes for *H. armigera*, all located in one cluster of duplicated genes on scaffold_0. Twenty genes from this clade were also recovered from *H. zea*, and 26 from *M. sexta*, but only eight from *B. mori* (Additional file 4: Section 2). There were also large P450 clusters: the CYP340K cluster (10 genes) on scaffold_107 and the CYP340H cluster (six genes) on scaffold_371, both in clan 4, plus the clan 3 CYP6AE genes (11) on scaffold_33. Excepting the relatively low numbers for *P. xylostella*, the differences in P450s, CCEs and GSTs are consistent with the hypothesised positive relationship of detoxification gene number to host range [11], with the net difference of the heliothines from *B. mori* and *P. xylostella* across the three families being at least 50 genes (Additional file 4: Sections 1–3).

**Fig. 3:**
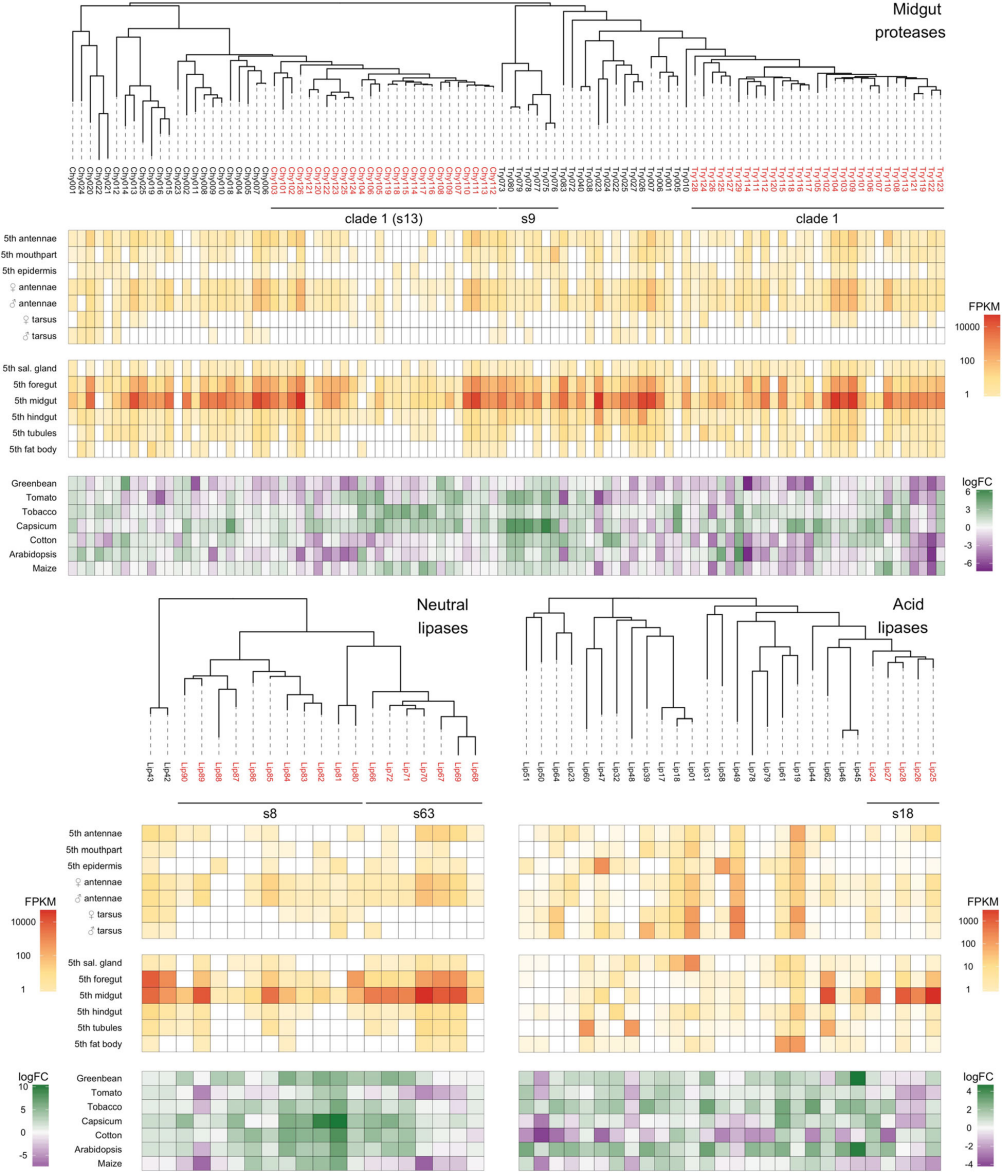
Phylogenetic, physical and transcriptional relationships within the major digestion gene clusters. Selected clades of serine proteases and lipases containing genes associated with digestive functions are shown. For the serine proteases, chymotrypsins (on the *left*) and trypsins (*right*) are shown as a single tree; the neutral and acid lipases are shown separately. Clades discussed more extensively in the text are highlighted in *red*. Further details about the gene names and their associated OGS numbers are given in Additional file 4: Sections 6, 7. *Bars below the gene names* indicate genes within a distinctive genomic cluster on a specific scaffold with the number shown; see Additional file 4: Sections 6, 7 for further details. The clade 1 chymotrypsins and trypsins are specifically indicated; for the latter, no single scaffold is shown because the cluster spans scaffolds 306, 5027, 842 and 194. The phylogenetic order shown does not reflect the physical order of genes within a cluster. Expression is given as FPKM for the tissue/developmental stage transcriptomes and logFC for the host-response transcriptomes

Consistent with their role in host use, the digestive proteases and neutral lipases are also similar in number in *H. armigera* and *H. zea,* and more numerous in both than in *B. mori* (Table 2) (comparable quality annotations not being available for *M. sexta* or *P. xylostella*). The differences are again substantial: ~200% in the case of the trypsins and neutral lipases, and ~50% for the chymotrypsins, giving well over a 50-gene difference in total. As above, many of the differences can be attributed to amplifications of particular gene clusters (Fig. 3; Additional file 4: Section 6). In *H. armigera*, there are 29 clade 1 trypsin genes, with 28 in a single genomic cluster, and 26 clade 1 chymotrypsin genes in a single genomic cluster (Fig. 3; Additional file 4: Section 6). While the largest cluster of acid lipases comprises just five genes, there are several expanded clusters of neutral lipases, the largest three containing 13, seven and five genes, respectively (Fig. 3 (showing two of these clusters); Additional file 4: Section 7).

Only one of the four families of chemosensory proteins, the gustatory receptors (GRs), showed large differences in number between the four species (Table 2; Additional file 4: Section 8, and see also [54]). In this case, *H. armigera* had 28% more genes than *H. zea* (213 vs 166, respectively), far more than would be expected simply from the difference between the two species in assembly quality. This concurs with the finding above that the GO terms most enriched among the *H. armigera* genes without *H. zea* equivalents included sensory perception and signal transduction of taste or smell. In fact, 47 (20%) of the 237 genes in this category for which we found GO terms were GRs. *H. armigera* also had about three times as many GRs as *B. mori*, and four times as many as *M. sexta* (213 vs 69 and 45, respectively). The difference from *B. mori* is again consistent with the enrichment of GO terms concerned with sensory perception and signal transduction related to taste or smell found among the *H. armigera* genes without equivalents in *B. mori*, as discussed above for Fig. 1. Notably, the oligophagous *M. sexta* has even fewer GR genes than *B. mori*; we do not know why this is so.

Few differences were evident among the two heliothines and *B. mori* in the numbers of genes involved in stress response and immunity (Additional file 4: Section 9) or in groups of genes important for larval growth, such as the cuticular proteins and transcription factors (Additional file 4: Section 10). The largest single cluster of duplicated genes we found anywhere in the *H. armigera* genome involved 60 cuticular protein RR-2 genes, the corresponding clusters in *H. zea* and *B. mori* comprising 58 and 54 genes, respectively (Additional file 4: Section 10). Full details of the genes in these families and functional classifications are provided in Additional file 6: Table S5.

### Evolutionary analyses of major gene family expansions in *H. armigera* and *H. zea*

Phylogenetic analysis revealed several major duplication events of detoxification and digestion-associated genes within the heliothine lineage which pre-dated the divergence of the two species but nevertheless occurred relatively recently within this lineage. For example, radiations of 11 CYP6AEs in clan 3, 25 CYP340s and 15 CYP4s in clan 4 (Additional file 4: Section 1), 15 of the clade 1 CCEs (Additional file 4: Section 2) and 23 each of the clade 1 trypsins and chymotrypsins (Additional file 4: Section 6) were found in the heliothine lineage. Many of these duplicated genes have been associated with rapid amino acid sequence divergence; for example, divergences within the three large clusters (i.e. clade 1 in each case) of CCEs, trypsins and chymotrypsins in *H. armigera* have resulted in identity ranges of 45–91%, 47–95% and 48–98%, respectively. Dating analyses using the Bayesian Markov chain Monte Carlo (MCMC) method in Bayesian evolutionary analysis by sampling trees (BEAST) v2.4.3 [55] showed that most of the duplications occurred from more than 1.5 to about 7 Mya (Additional file 4: Table S6; Additional file 7). This range pre-dates the estimate by Mallet et al. [25] and Behere et al. [26] of around 1.5 Mya for the divergence of *H. armigera* and *H. zea*, a date supported by our analysis below.

Phylogenetic analyses of the GRs (Additional file 4: Section 8) showed that the very large numbers of those genes in the heliothines compared to *B. mori* were also largely due to recent amplifications within the heliothine lineage. On the other hand, the larger number of GRs in *H. armigera* than *H. zea* could be attributed to the loss of genes in the *H. zea* lineage, since our divergence dating puts those amplifications earlier than the *H. zea/H. armigera* split. Furthermore, the fact that 12 of the 20 genes among the 2269 manually curated *H. zea* gene models which had internal stop codons were GRs (cf. none in *H. armigera*; Additional file 4: Section 8) suggests that the process of GR gene loss in *H. zea* may be ongoing.

We next carried out several analyses on the evolutionary changes in the above major gene families. As noted, a large body of empirical evidence from a wide range of insect species enables us to partition the clades within the P450, CCE and GST families into those that have been recurrently associated with detoxification functions and those for which there is little or no empirical evidence of such functions. Nine of the *H. armigera* genes in the detoxification lineages, but none of the genes in the other lineages, were found to be missing in the *H. zea* assembly. We then compared the rates of amino acid sequence divergence between the two heliothines for P450, CCE and GST genes in these two sorts of lineages. We found that the *K*_a_/*K*_s_ statistics in the lineages directly associated with detoxification functions generally diverged in amino acid sequence more rapidly between the two heliothines than did other lineages in these families (Table 2). Finally, we used Tajima’s relative rate test to screen for heterogeneity in rates of amino acid sequence divergence among closely related paralogues in these lineages (Table 3; Additional file 4: Table S7), finding that 42% (19/45) of the pairs in the detoxification lineages yielded significantly different rates, whereas only 14% (2/14) of pairs in other lineages in these families did so. Significant differences in rates were also observed for several major digestive clades, particularly among the chymotrypsins, and for several GR lineages (Additional file 4: Table S7).

**Table 3:** Detoxification gene clades showing enhanced sequence divergence in *H. armigera* and gene loss in *H. zea*

Family	Clan/group	Gene number in *H. armigera*	Gene pairs tested	Significant rate difference (*p* < 0.05)	*H. armigera* genes not in *H. zea*
P450	Detox, clan 3	43	9	3	3
	Detox, clan 4	47	11	5	2
	Other	6	4	2	0
CCE	Detox	55	19	7	4
	Other	16	9	0	0
GST	Detox	36	8	4	2
	Other	3	1	0	0

Tajima’s relative rate tests were performed on the numbers listed of *H. armigera* paralogue gene pairs in the major detoxification groups; for each group examined, the number of pairs showing a significant rate difference is given. Also listed are the numbers of genes in the relevant clades missing in the *H. zea* assembly. The P450, CCE and GST families are partitioned in these analyses into lineages for which there is empirical evidence for detoxification functions and those for which there is little or no such evidence. More details of the specific genes involved and comparable data for the proteases, lipases and GRs are given in Additional file 4: Table S7

Overall, the picture emerging from the evolutionary analyses is of extensive recent amplification and rapid sequence divergence among several clades of the detoxification, dietary and GR gene families in the heliothine lineage prior to the *H. armigera*/*H. zea* split, with the subsequent loss of some detoxification and more GR genes in *H. zea*. We propose that the gene amplification and diversification prior to the split reflect the emergence of this highly polyphagous branch of the heliothine megapest lineage, while the subsequent loss of genes in *H. zea* reflects its contraction to a somewhat narrower host range than that of *H. armigera*. We do not know how their host species differed in pre-agricultural times, but, notwithstanding considerable overlap, there are now some differences between them. Cunningham and Zalucki [27] list hosts from 68 plant families for *H. armigera* but only from 29 families for *H. zea*. Many papers on the ecology of *H. zea* cite its heavy dependence on maize, soy and, in some cases, their wild relatives [56–61], while some major papers on *H. armigera* [57, 62, 63] stress that large populations of the species live on diverse wild hosts outside agricultural areas.

### Transcriptomic profiles of the detoxification and digestive genes across tissues and developmental stages

A profile of tissue/stage-specific gene expression was built up from 31 RNA-seq-based transcriptomes from either whole animals or specific tissues/body parts, with 15 of the latter being from fifth instar larvae and 12 from adults (Additional file 4: Table S8). These included tissues important in sensing, detoxification or digestion in adults (antennae and tarsi of each sex) and larvae (mouthparts, salivary gland, gut, tubules, fat body and epidermis). Transcripts from a total of 13,099 genes were detected at levels sufficient to analyse, including 303 of the 353 genes from the detoxification families and 145 of the 193 from the digestion families above (see Additional file 4: Sections 1–7 for full details); the chemosensory genes generally showed too little expression for meaningful analyses.

The results for the P450 clans, CCE clades and GST classes most often associated with detoxification and/or where we found the largest differences in gene number between the species above are summarised in Fig. 2. Relatively high expression (fragments per kilobase of transcript per million mapped reads (FPKM) >30) was found for many of the CYP6s and CYP9s in various detoxification and digestion-related tissues and for some of the CYP4s in various detoxification-related tissues; for one particular clade of delta GSTs and most of the sigma GSTs in most detoxification and digestive tissues; and for about half of the CCEs in clades 1, 6 and 16, mostly in digestive tissues, principally fifth instar midguts. The ABC transporters were expressed in most tissues screened, with one particular lineage (the ABCG subfamily) expressed at higher levels in several detoxification-related tissues and also salivary glands, while relatively high UGT expression was found for the UGT-40 lineage in various detoxification and digestive tissues (Additional file 4: Sections 4, 5).

For the digestion-related families, Fig. 3 shows that expression of most midgut proteases was high in fifth instar midguts and to a lesser extent foreguts, with little expression elsewhere. Interestingly, as was the case with the clade 1 CCEs, particular subclades of the clade 1 trypsins and chymotrypsins were only expressed at low levels in any of the digestive (or detoxification) tissues. The lipases showed a more complex pattern of expression, with the galactolipases among the neutral lipases (the clusters containing HarmLipases 33–37 and 66–71) and a recently diverged cluster of acid lipases (HarmLipases 24–28) among the minority heavily expressed in mid- or foregut. On the other hand, the medium- (8–16 residues) and large- (21–26 residues) lidded neutral lipases (HarmLipases 09, 40, 54–56, 04 and 77, and 02, 03, 38 and 93; i.e. groups 5, 7 and 8b respectively in Additional file 4: Section 7), as well as several triacylglycerol and miscellaneous other lipases, were expressed in a range of other tissues (mainly fat body, salivary gland, silk gland and cuticle).

### Larval growth and transcriptomic responses of the detoxification and digestion genes on different hosts

*H. armigera* larvae were raised on seven different species of host plant known to differ in their quality as hosts [64] plus the soy-based standard laboratory diet used in the first transcriptomics experiment above. The laboratory colony is normally maintained on the standard diet, but remains capable of completing its life cycle on host plants such as cotton [65]. Use of this colony allows ready comparison of the responses to different host plants at the whole genome level.

**Fig. 4:**
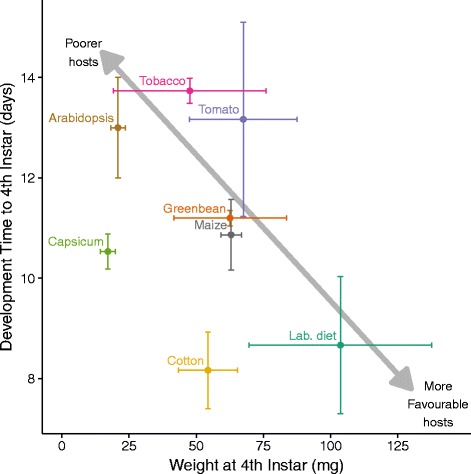
Effects of rearing diet on development time and weight gain. The mean weights and development times with their standard errors are plotted for larvae from each diet

The experiment was designed to measure developmental time to, and weight and gene expression profiles at, a specific developmental stage, i.e. instar 4 plus 1 day. All hosts allowed larvae to develop to this point. There were large differences in the performance of the larvae on the eight diets, with mean development time to harvest varying between 7 and 15 days and mean weight at harvest varying between 13 and 150 mg (Fig. 4). The laboratory diet was clearly the most favourable, with the larvae developing relatively rapidly and growing to the largest size, while Arabidopsis was clearly the poorest, giving the longest development time for a very low larval weight. Maize and green bean yielded midrange values for both measures. Cotton and Capsicum produced relatively small but rapidly developing larvae, whereas tomato and tobacco produced relatively large but slowly developing larvae. It is of interest that the diet allowing most rapid completion of development was in fact cotton; this was also found to be the case by Liu et al. [64].

Gene expression was then profiled at the defined developmental point. Read mapping of RNA-seq data for the whole fourth instar larvae to the OGS2 yielded data for 11,213 genes at analysable levels (a minimum level of 5 reads per million across three libraries). Differential expression (DE) on plant hosts compared to the control diet was then calculated for each of these genes, with 1882 found to be differentially expressed on at least one host (Additional file 8: Table S9). These 1882 genes included 185 of the 546 genes in the detoxification and digestion-related families above (analysable data having been obtained for 452 of the 546). This was a highly significant, greater than threefold enrichment (hypergeometric test *p* = 1.5 × 10^–48^) of these families compared to their representation in the genome overall. The 185 DE genes included approximately one-third of each of the detoxification and digestion sets. The chemosensory proteins were only poorly represented among the 11,213 genes with analysable data; only 10 GRs were analysable and none of them were differentially expressed.

Initial analysis of DE genes in the major detoxification and digestion-related gene families (Figs. 2 and 3) found wide variation in transcriptional responses among both the hosts and the genes. Nevertheless, some clear patterns emerged. Most of the genes in the five detoxification families were upregulated on the least favoured diet, Arabidopsis, and for four of these families most of the genes screened were downregulated on cotton. For the P450s and CCEs, tobacco also elicited a broadly similar upregulation response to Arabidopsis. For the GSTs, most genes were downregulated on every host other than Arabidopsis, with maize eliciting the most frequent downregulated response. The UGTs also produced downregulated responses on several hosts other than Arabidopsis, but in this case maize elicited some upregulated responses. Most ABC transporters were upregulated on every host other than cotton and to a lesser extent Capsicum.

Many of the genes in the five detoxification-related families which were most prone to differential regulation across the various hosts occurred in physical clusters. These genes included the CYP340K cluster on scaffold_107, the CYP340H cluster on scaffold_371, the CYP341 genes on scaffold_21, the clade 1 esterases mentioned above and a large cluster of 13 UGT33 genes on scaffold_562. Many others, although not always physically clustered, were nevertheless closely related in a phylogenetic sense, for example, the GSTD1n, GSTS2, ABCB and ABCC lineages. In a few of these cases, such as the CYP340 and 341 clusters and the GSTD1n lineage, some of the genes within each cluster/lineage showed similar patterns of DE. However, in most cases, different genes within each cluster or lineage reacted differently to the different hosts. Thus, considerable regulatory evolution has accompanied the diversification of coding sequences within these clusters and lineages.

Importantly, many of the genes in the detoxification families most prone to DE on the various host plants were not necessarily ones that had been heavily expressed in the tissues related to detoxification or digestion on the laboratory diet. Genes prone to host plant-related DE that had been highly expressed in the tissues on the laboratory diet included some CYP6s, CYP337s and delta GSTs. However, genes prone to DE on the different hosts that had shown little expression in the tissues on the laboratory diet included several CYP340s, clade 1 CCEs, ABCs and UGTs (Fig. 2). This accords with empirical evidence that many detoxification genes are inducible in response to xenobiotic exposure [51–53].

Many of the midgut proteases also showed DE on different host plants (Fig. 3). Overall, the proteases were more likely to be downregulated on the host plants compared to the protein-rich soy-based laboratory diet, this effect being most pronounced on green bean, cotton and Arabidopsis. These downregulatory responses were most evident in certain regions of the clade 1 trypsin and chymotrypsin clusters. On the other hand, Capsicum and to a lesser extent tobacco elicited several upregulatory responses in other regions of these two clusters, with some specific genes, e.g. Try116 and Try118, showing divergent responses on green bean and Capsicum. For Capsicum and to a lesser extent tomato, upregulatory responses were also evident in the cluster of seven trypsin genes on scaffold_9. Coordinated changes across several hosts were evident for Tryp114–120 within the clade 1 trypsin cluster but, as with the detoxification genes above, even closely linked genes within genomic clusters generally diverged in their transcriptional responses across the panel of diets.

Many of the acid lipases, but only a phylogenetically restricted minority of the neutral lipases (clades 1 and 2, each with nine genes), also showed significant DE across the various diets (Fig. 3). In contrast to the proteases, the diet-responsive lipases were most often upregulated on the host plants as opposed to the laboratory diet, which is consistent with the fact that laboratory diets generally have higher levels of free fatty acids than the host plants [66]. Interestingly, tobacco, Arabidopsis and to a lesser extent green bean elicited similar responses from many of the genes in both sets of lipases. Otherwise, however, the lipases showed a diversity of host responses more akin to the diversity seen in the other gene families above. Thus, there were relatively few cases of closely related lipase genes within clusters showing the same expression profiles across the various diets and, as with the other systems above, those that did generally involved the most recently diverged clusters (e.g. the neutral lipases HarmLipases 82–84; 67, 69 and 70; and 66, 71 and 72; Additional file 4: Section 7).

Fewer genes implicated in growth and morphogenesis and stress responses showed DE across the hosts (Additional file 4: Sections 9, 10) than did the families above, although some involved in growth and morphogenesis showed DE on cotton and Arabidopsis, and some stress response genes showed DE on Capsicum. The cotton-specific expression changes may be due to the faster rate of developmental stage progression on this host, meaning that more gene families, pathways and networks show variable expression at any particular time point.

**Fig. 5:**
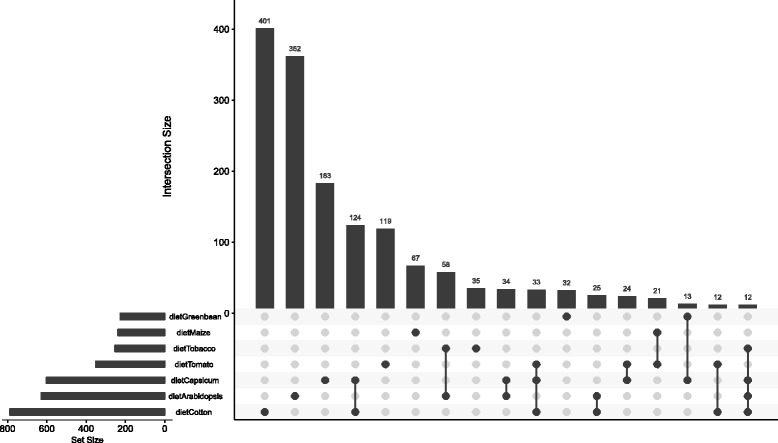
Numbers of genes differentially expressed on each of the different diets. The seven diets are listed at the bottom of the figure, with the total numbers of DE genes on each diet shown by the horizontal histogram at the *lower left*. The main histogram shows the number of DE genes summed for each diet individually and for various diet combinations. The diets for which each number is calculated are denoted by *black dots*, representing either a single diet plant or a combination of multiple different diets. See also Additional file 3: Figure S3 for a principal component analysis showing the relationships among the transcriptional responses to the different diets

Overall, most (1199) of the total set of 1882 DE genes across the genome were only identified as DE on a single diet, suggesting a specific response to the particular characteristics of the host plant (Fig. 5). Each host plant elicited DE in at least 200 genes, with cotton, Arabidopsis and Capsicum each affecting more than 600. The most common shared responses involved genes that were differentially expressed on cotton and Capsicum (124 genes) and to a lesser extent on Arabidopsis and tobacco (58 genes). Notably, Arabidopsis and tobacco were the poorest hosts (long developmental time and low larval weight), and cotton and Capsicum were also relatively inefficiently used (shorter developmental time, but still relatively low weight gain) (Fig. 4).

### Integrating the tissue/developmental stage and host-response transcriptomics

Two weighted gene co-expression networks were constructed, one for each of the tissue/developmental stage and host-response data sets, using sets of 13,099 and 7977 rigorously filtered genes, respectively (see Methods). Each network assigned each gene in the data set to a co-expression module containing genes with the most similar expression profiles to it.

**Fig. 6:**
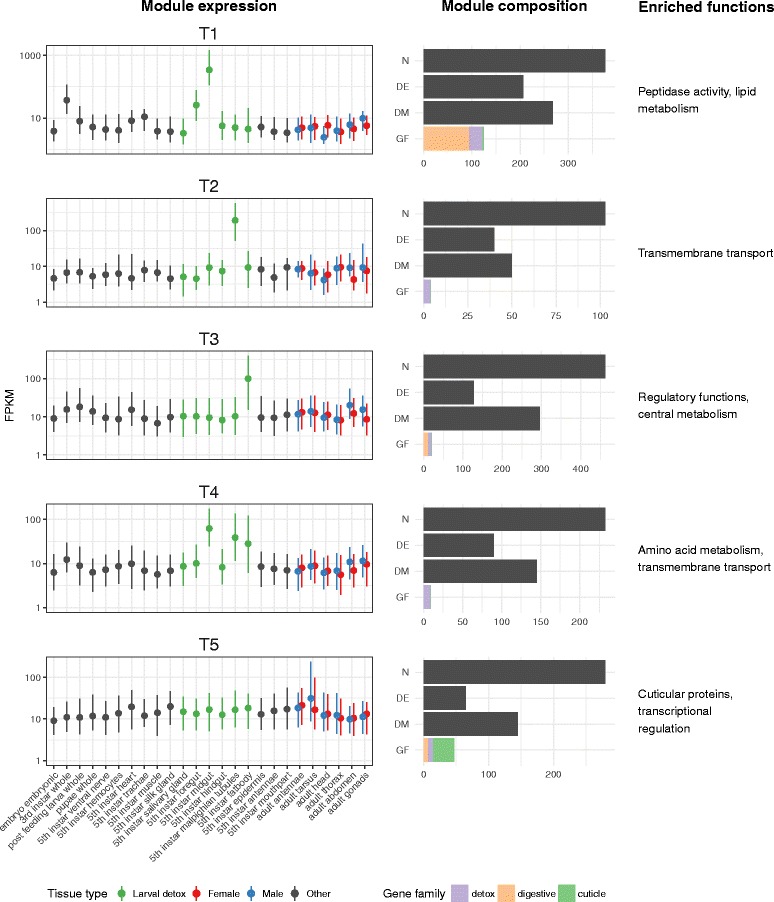
Expression profiles for selected co-expression modules from the tissue/developmental stage transcriptomic experiment that are enriched for diet-responsive genes. The five modules for which expression profiles are shown are those most enriched for genes called as DE in the host-response experiment (see text). Expression (*FPKM*) profiles for each module are shown on the *left*, with the tissue types (see text) identified by colour as in the legend. The composition of each module is described in the *central panels*, showing the total number (*N*) of genes per module, the number that are *DE*, the number in all diet co-expression modules (*DM*) and the number in the major gene family (*GF*) classes defined by the *key below*. Major functions enriched in each module are noted on the *right* of the figure

Five of the 47 co-expression modules recovered from the tissue/developmental stage network were highly enriched for genes among the 1882 identified above as differentially expressed in response to diet; 529 of the 1456 genes in these five modules were among the 1882 DE genes (Fig. 6). These five modules highlight the important tissues involved in that response, with, as expected, tissues implicated in detoxification and digestion being strongly represented: four of these modules contained genes expressed specifically in the larval fore/midgut (T1), the Malpighian tubules (T2), the fat body (T3) or in all detoxification/digestion tissues (T4). The fifth module (T5) corresponds to genes expressed in the sensory apparatus (larval antenna/mouthparts and adult antennae/tarsus), highlighting that sensory/behavioural responses play a key role in host plant adaptation in *H. armigera* [27].

**Fig. 7:**
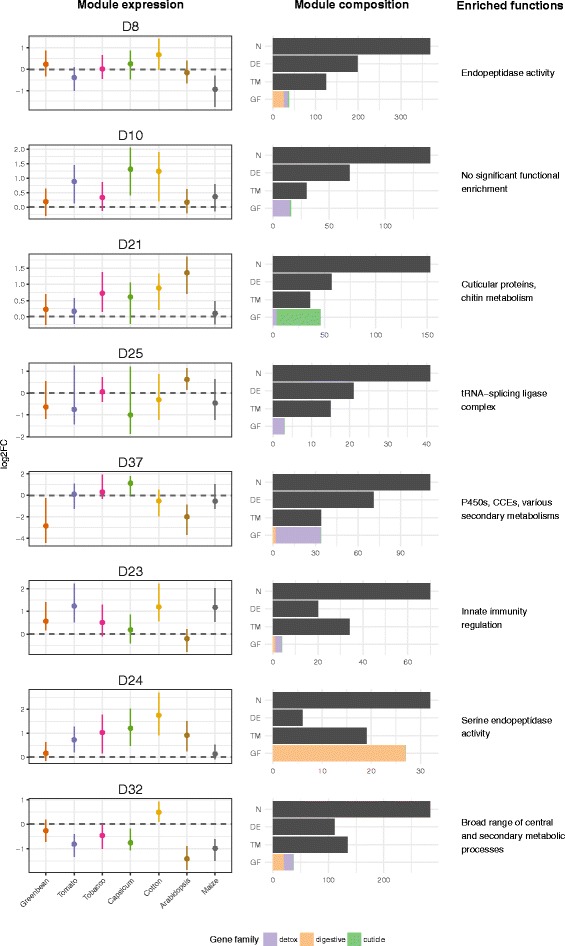
Expression profiles for selected co-expression modules from the host-response transcriptomic experiment. The eight modules for which expression profiles are shown are those most enriched for DE genes. Four of these modules (see text) are also significantly enriched in genes from the detoxification- and digestion-related families. Expression (*log2FC*) profiles for each module are shown on the *left*. The composition of each module is described in the *central panels*, showing the total number (*N*) of genes per module, the number that are *DE*, the number in the five tissue/developmental stage modules T1–T5 (*TM*) and the number in the major gene family (*GF*) classes defined by the *key below*. Major functions enriched in each module are noted on the *right* of the figure. See Additional file 4: Section 11 for more detailed analyses of the host-response network including aspects illustrated by the co-expression modules D20 and D3

The host-response co-expression network yielded 37 modules, of which nine were enriched for genes in the 1882 DE gene set above (675 of the 1485 genes in these nine modules being DE genes) and are therefore most likely to contain networks of genes involved in host response (Fig. 7). Four (D8, D10, D21 and D25) of these nine modules were also significantly enriched for the 546 genes in the families identified a priori as containing general detoxification (D10) and digestion (D8 — specifically protease) related functions (Fig. 7), as was one further module, D37 (Additional file 4: Table S10a; Additional file 9: Table S10b). Five of the nine modules (D8, D10 and D25 again, as well as D23 and D24) were also significantly enriched for the 1456 genes in the five stage/tissue co-expression modules involving tissues with detoxification- and digestion-related functions (Additional file 4: Table S10a), consistent with these modules’ enrichment for DE genes. Three further diet modules were identified as also enriched for genes in these developmental modules, one of which (D37, the other two being D3 and D32), as noted, had also been enriched for the 546 a priori identified genes in detoxification/digestion gene families (Additional file 4: Table S10a). D37 is of particular note, being specifically enriched (27 of its 32 members) for midgut trypsin and chymotrypsin sequences in the two large clusters shown in Fig. 3; while expressed at relatively low levels on the control laboratory diet, these genes were all upregulated on several of the plant hosts.

Unsurprisingly, the three diet modules D8, D10 and D25, which were significantly enriched for all three sets of genes above (i.e. the 1882 DE genes, the 546 in the key gene families and 1456 in the five key tissue/developmental stage modules), were all over-represented with GO terms covering functional annotations such as catabolism, amylase, endopeptidase, carboxylester hydrolase and monooxygenase (Additional file 3: Figure S4). D25 alone contains 11 P450s from clans 3 and 4, 10 CCEs, including six from clade 1, nine UGTs, two delta class GSTs, a trypsin and a lipase. Notably also the transcription factors in these modules — three each in D8 and D10 and one more in D25 (Additional file 4: Section 11) — are candidates for the crucial upstream regulatory roles controlling host responses (see also Additional file 4: Section 10; Additional file 10). The plants on which these modules with significant numbers of the transcription factors (e.g. D8 and D10) were most upregulated — cotton, Capsicum and Arabidopsis — were among the most problematic or inefficiently used of the hosts tested.

Taken together, the expression data illustrate the considerable extent to which the *H. armigera* larval host response involves coordinated expression, on a tissue-specific basis, of specific genes, including a significant number of those in the major detoxification- and digestion-related families. Further, the diversity of co-expression patterns across the different host plants emphasises the transcriptomic plasticity of *H. armigera* larvae. It will be of great interest now to test whether *H. zea* shows comparable levels of transcriptomic plasticity on similar hosts.

### Resequencing data

Whole genome sequence data from a total of four *H. armigera* lines and five *H. zea* lines/individuals were analysed to further investigate the genetic relationships between the two species. In addition to the reference lines for the two species, from Australia and North America, respectively, the sample included two Chinese and one African-derived *H. armigera* lines and four *H. zea* individuals from North America. Single-nucleotide polymorphisms (SNPs) in the nine resequenced genomes were called in two ways, one from each of the two species’ reference sequences.

**Fig. 8:**
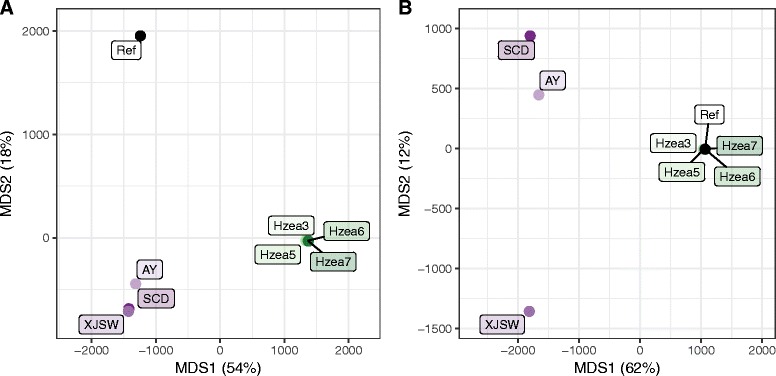
Population structure. Results of MDS analyses, using (**a**) *H. armigera* and (**b**) *H. zea* as the reference strain. The proportion of variance explained by each dimension is given as a percentage on the axis label. To include the reference strains on these plots, genotypes for each reference strain were recoded as 0/0

When the SNPs were called from the *H. armigera* reference sequence, a multi-dimensional scaling (MDS) analysis placed the resequenced genomes for each species very close to each other and well separated from the other species, but the *H. armigera* reference line was well separated from both these groups, albeit closer to the other *H. armigera* than the *H. zea* samples (Fig. 8a). When the SNPs were called from the *H. zea* reference line, the MDS placed all five *H. zea* sequences close to one another and well separated from all the *H. armigera* samples, but the latter could then be separated in the second MDS dimension, with one Chinese sequence (SW) slightly removed from both the other Chinese sequence (AY) and the African-collected laboratory strain (SCD) (Fig. 8b). The separation of the *H. armigera* reference from the other *H. armigera* lines (Fig. 8a) probably reflects the fact that the *H. armigera* reference line represents a distinct subspecies, *H. armigera conferta*, which is present only in Australia, New Zealand and some south-west Pacific islands [23, 37]. Notwithstanding their differing geographic ranges, both subspecies are found in a very wide range of ecological habitats, and there is no evidence as yet that they differ in their ability to inhabit any specific ecology [27, 57, 63, 67]. Whole genome sequences of comparable quality of the two *H. armigera* subspecies will be needed to identify particular genome sequences distinguishing the two.

With both MDS analyses supporting the view that *H. armigera* and *H. zea* are indeed separate species, we next estimated the date of the divergence between *H. armigera* and *H. zea* by conducting a coalescence analysis using sequence data for 16 recently diverged loci (Additional file 3: Figure S5; Additional files 11 and 12). The resulting tree, with *H. punctigera* as the outgroup, confirmed *H. armigera* and *H. zea* as two distinct species. The divergence dates between the three species were then estimated by applying the coalescence to the 12 most rapidly evolving of the 16 genes [68]. We calculated that *H. armigera* and *H. zea* diverged 1.4+/–0.1 Mya, their lineage and that leading to *H. punctigera* diverged 2.8+/– 0.2 Mya and the Australian *H. armigera* lineage diverged from the other analysed *H. armigera* lineages 0.9+/–0.1 Mya. Our coalescent analyses are therefore consistent with the general assumption in indicating that all our *H. zea* lines diverged from *H. armigera* prior to the divergence among the sequenced *H. armigera* lines (although Leite et al. [20] had suggested *H. zea* was the basal lineage). The estimate for the *H. armigera*/*H. zea* split agrees well with previous estimates of around 1.5 Mya for this date, based on biochemical genetics [25] and mitochondrial DNA (mtDNA) phylogenies [26] using a mutation rate estimate of 2% per million years in Drosophila mitochondrial DNA [69]. We find no evidence for introgression between the species since. Our estimates also concur with those of Cho et al. [12] in placing *H. punctigera* basal to the *H. armigera*/*H. zea* lineage, although the date of this divergence has not previously been estimated.

Estimates of genome-wide diversity (pi) were consistently about twice as high within the resequenced *H. armigera* genomes as they were within the resequenced *H. zea* genomes (Additional file 3: Figure S6), regardless of which species was used as the reference. Interestingly, however, the *H. armigera* sequences showed lower diversity values for non-synonymous sites compared with synonymous sites than did *H. zea* (Additional file 3: Figures S6, S7). Thus, although there was greater heterozygosity overall in the *H. armigera* samples, their non-synonymous sites showed more evidence of selective constraint than did the *H. zea* samples. Note that the absolute values for diversity shown in Additional file 3: Figure S6 (~0.015 for *H. armigera* and 0.004 for *H. zea*) are lower than those reported by others (e.g. see [37, 70]), probably due to the more stringent filtering used to allow us to compare individuals from the two species (see Methods). Nevertheless, the relative levels of polymorphism are consistent across all these studies.

Consistent with the estimates of heterozygosity, Bayesian skyline plot analysis using the resequencing data consistently estimated a much (~10×) greater contemporary effective population size for *H. armigera* than for *H. zea* (*N*_*e*_ ~ 2.5 × 10^8^ and 2.5 × 10^7^ respectively). In addition, our estimates of effective population size change through time indicated an expansion in *H. armigera* around 6–8 Mya. By contrast, the effective population size of *H. zea* increased only slowly from about 1.5 Mya. All these values were obtained using the corresponding reference genomes to call the SNPs, but essentially the same results were obtained whichever reference genome was used (data not shown).

We found small but significant positive correlations between *H. armigera* and *H. zea* in the pattern of variation in pi across their genomes. This was true for both their synonymous and non-synonymous sites, although the correlation was slightly stronger for the synonymous sites (rho = 0.421 cf. 0.387, *p* < 0.001 for both; Additional file 3: Figure S7). This difference is to be expected, as lineage-specific selective pressures will result in greater diversity between the species at non-synonymous sites. The size of the correlations seen for both the synonymous and non-synonymous sites implies that, while a large proportion of variance in diversity across genomic bins is shared across the two species, the majority (~0.6) of this variance is in fact not shared between them.

### Candidate insecticide resistance genes

Paralleling its greater host range, *H. armigera* is also considerably more prone to develop insecticide resistance than *H. zea*, even though many populations of both are heavily exposed to insecticides [30, 71]. *H. armigera* has developed resistance to many chemical insecticides, including organochlorines, organophosphates, carbamates and pyrethroids (see [30, 72–74] for reviews), and, more recently, to the Cry1Ab, Cry1Ac and Cry2Ab Bt toxins delivered through transgenic crops [75]. By contrast, in *H. zea* significant levels of resistance have only been found for organochlorines and pyrethroids and, even then, relatively infrequently [30].

In most of the *H. armigera* cases at least one of the underlying mechanisms is known, but specific mutations explaining some of the resistance have only been identified for three of them, specifically the metabolic resistance to pyrethroids and the Cry1Ab and Cry2Ab resistances [31, 32, 76, 77]. However, in several of the other cases bioassay and biochemical information on the resistance in *H. armigera* or *H. zea*, together with precedent molecular studies from other species, indicate the genes likely to be involved. We therefore screened our sequence data for the presence of intact copies of those genes, their expression profiles and mutations recurrently found to confer resistance in other species. The reference Australian *H. armigera* colony and the resequenced African strain are known to be susceptible to most if not all the insecticides above, but the two Chinese lines could be resistant to pyrethroids and possibly other chemical insecticides [71, 78]. The Chinese AY line had also been shown to be resistant to the Cry1Ac Bt toxin [79]. The reference *H. zea* line is susceptible to all the insecticides above, and the resequenced lines were also derived from populations known not to have any significant resistances. The results of our screens are detailed in Additional file 4: Section 12 and summarised below.

Resistance due to insensitive target sites has been demonstrated for organochlorines, organophosphates and pyrethroids in *H. armigera*. These resistances would be expected to involve gamma-aminobutyric acid (GABA)-gated chloride ion channels, acetylcholinesterase-1 or possibly acetylcholinesterase-2 and voltage-gated sodium channels, respectively. We found good models of the key genes, with wild-type sequences lacking known resistance mutations, in both species. The transcriptome data show them to be well expressed in neural tissue. Both *H. armigera* and *H. zea* were found to have orthologues of certain additional GABA-gated chloride ion channel genes found in other Lepidoptera; although these genes have sequence variations at locations associated with resistance mutations in other insects, none of these changes in Lepidoptera have been associated with resistance (Additional file 4: Section 12).

Resistance due to enhanced metabolism of the insecticide has been demonstrated for organophosphates and pyrethroids in *H. armigera*. The organophosphate resistance is correlated with the upregulation of several clade 1 carboxylesterases [80], particularly CCE001g, but which of the overexpressed CCEs actually causes the resistance remains unknown. The pyrethroid resistance is mainly caused by enhanced P450-mediated metabolism, and much of this is due to novel CYP337B3 genes resulting from fusions of parts of the adjacent CYP337B1 and CYP337B2 genes through unequal crossing over [76, 81]. Although CYP337B3 alleles have been identified at various frequencies in populations around the world, there was no evidence, either from screening for reads that cross the fusion junction or from read densities for the constituent sequences, for their existence in any of the sequenced lines for either species. Another P450 gene that is interesting in relation to insecticide resistance is the CYP6AE14 gene. This P450 was originally implicated in the metabolism of a particular insecticidal compound produced by cotton (gossypol) but is now thought to have a more general role in detoxifying various plant defense chemistries [82–84]. Notably, we find no evidence of the CYP6AE14 gene in any of our *H. zea* genome or transcriptome data.

Several molecular mechanisms have been reported for resistances to Bt toxins in *H. armigera.* They involve disruptions to the cadherin [31] or ABCC2 transporter [77] proteins in the larval midgut for the Cry1Ab/c toxins, and to ABCA2 proteins for the Cry2Ab toxin [32]. All these resistance mutations are recessive. We find intact gene models for these genes in both reference genomes and the resequenced lines. Although the AY strain is known to be resistant to Cry1Ac, that resistance is dominant [79] and therefore likely to be due to mutation in an unknown gene different from those mentioned above.

The genomes of both species therefore contain good models of the genes encoding the target sites for several classes of chemical insecticides and Bt toxins for which target site resistance has been reported in *H. armigera* or other species. This would be expected given the known essential neurological functions of the chemical insecticide targets and the indications of important functions for the Bt targets provided by the fitness costs in the absence of Bt commonly associated with Bt resistance mutants [85]. Notably, however, we found two presence/absence differences in genes implicated in metabolic resistance to chemical insecticides or plant toxins in *H. armigera*. In both cases, as described above, the gene has been found in *H. armigera* populations but not in our *H. zea* data. One is the chimeric CYP337B3 gene, and the other is CYP6AE14. These cases may represent benefits to *H. armigera* from specific neofunctionalisations enabled by the extensive duplication of its detoxification genes. Also relevant here is our evidence for this species’ diverse upregulatory responses of various detoxification genes to different hosts. Given emerging evidence for similar sorts of upregulatory responses to various insecticides [72], and the abilities of some of the detoxification enzymes to bind/transform a wide range of insecticides [86–88], its unusually large repertoire of detoxification enzymes may provide *H. armigera* with a high level of metabolic tolerance to many insecticidal chemistries.

## Conclusions

A major characteristic of the two heliothine genomes which could explain those species’ polyphagy and pest status concerns their complements of genes involved in host finding and host use. The ~3000 annotated genes we found in *H. armigera* but not in *B. mori* were enriched for GO terms relating to taste and smell, proteolysis and detoxification. *H. armigera* had over 70 more genes in families associated with detoxification (mainly P450s, CCEs and GSTs), over 90 more in families associated with digestion (midgut proteases and neutral lipases) and over 150 more chemosensory proteins (almost all GRs), for a total of over 300 additional genes across these families. Comparisons of *H. zea* to *B. mori* showed the same pattern, albeit *H. zea* has fewer GRs, in particular, than does *H. armigera*.

These numbers provide stronger support within Lepidoptera for a positive correlation, previously proposed on the basis of automated annotations, between the sizes of three major detoxification gene families studied here (P450s, CCEs and GSTs) and host range [11]. The two heliothines, with a host range extending across many families and at least 14 orders, average 247 genes in these families (Additional file 3: Figure S8). In contrast, three species (*M. sexta*, *Chilo suppressalis* and *P. xylostella*) which are less polyphagous, with host plants limited to a single family in each case, have an average of 180 genes, and four others (*B. mori*, *Danaus plexippus*, *Melitaea cinxia* and *H. melpomene*), whose host range is limited to a single species or genus of plants, have an average of 159 genes.

Several lines of evidence also emerge from both our genomic and transcriptomic analyses which suggest that the additional detoxification, digestive and GR genes in the two *Helicoverpa* species contribute directly to greater functional versatility. Firstly, many of the duplicated genes have been associated with rapid amino acid sequence divergence, for example within the respective largest clusters in *H. armigera* of CCEs, trypsins and chymotrypsins, and relative rate tests among paralogues in these clusters show evidence for functional divergence. Secondly, transcriptomic analysis shows that many of the duplicated genes in the detoxification- or digestion-related families are expressed in relevant tissues and are enriched several fold among the 1882 genes that were found to be significantly differentially expressed on different hosts.

Thus, the extreme polyphagy that has evolved in the two ‘megapest’ heliothines appears to have been associated with the duplication and neofunctionalisation of many genes involved in host finding or host use, prior to their divergence, and, at least for the detoxification- and digestion-related genes, with a diversification of their expression patterns in response to different hosts.

We estimate that the two heliothine species diverged about 1.4 Mya, in good agreement with earlier suggestions. We found no evidence for introgression between the species since their divergence, and our phylogenetic and comparative analyses show a possible genomic basis for the functional divergence between them, consistent with *H. zea* having a somewhat narrower host range than *H. armigera*. We find that *H. zea* has lost some genes in specific P450, CCE and GST lineages directly associated with detoxification functions and as many as 28% of its GRs since its divergence from *H. armigera*. We also find evidence that GR gene loss in *H. zea* may be ongoing. Evolutionary rate tests among the GRs and in the P450, CCE and GST lineages directly associated with detoxification also showed rapid divergence between orthologues in the two species. These findings suggest that their respective host plants have selected for some different detoxification capabilities and gustatory responses in the two species.

The ability of *H. armigera* in particular to develop resistance to all known classes of insecticides is the other fundamental reason for its megapest status. *H. zea* has not shown this ability to the same extent. The genomes of both species contain good models of the genes encoding the target sites for several classes of chemical insecticides and Bt toxins for which target site resistance has been reported in *H. armigera* or other species, as well as several genes implicated in metabolic resistance. There are, however, two P450 genes implicated in metabolic resistance to chemical insecticides or plant toxins that are present in *H. armigera* populations but not in our *H. zea* data. One is the chimeric CYP337B3 gene associated with pyrethroid resistance, numerous alleles of which are found worldwide, and the other is CYP6AE14, thought to be associated with plant allelochemical detoxification. These cases may represent benefits to *H. armigera* from specific neofunctionalisations enabled by the extensive duplication of its detoxification genes.

That both reference genomes contain good models for most of the genes implicated in metabolic or target site resistance to insecticides through mutation suggests that the higher resistance propensity of *H. armigera* may be largely due to another factor. This is its greater genetic polymorphism, which is about twice that of *H. zea*. Many insecticide resistances have been shown to arise from pre-existing rather than new mutations in candidate genes [65, 89, 90], and so *H. armigera* may be better pre-adapted for resistance than is *H. zea*. The greater population size we estimate for *H. armigera* (~10 times greater than that of *H. zea*) would also assist in this by allowing for the retention of a greater pool of potentially useful rare variants.

The demographics of both *Helicoverpa* species have been changing rapidly over the last decade in the Americas, with the incursion and proliferation of *H. armigera* into South America and its progressive spread into central and North America. It is unclear whether, and where, it might replace *H. zea*, but recent data [37] suggest that some level of hybridisation and introgression of *H. armigera* genes into *H. zea* could already be occurring in South America. Our data do not speak directly to the issue of replacement vs co-existence of the species in the absence of introgression, but they do provide two important insights in respect of introgression. Firstly, the high levels of orthology and synteny between the genomes provide no reason to anticipate genome incompatibilities or hybrid/backcross breakdown to occur over time. Secondly, some key differences found between the genomes, the additional GRs, the CYP6AE14 implicated in tolerance to plant defense chemistry, the CYP337B3 gene conferring synthetic pyrethroid resistance and the various Bt resistance genes in *H. armigera* being obvious examples, could provide the basis for generating novel hybrid ecotypes that are both locally adapted and insecticide resistant.

## Methods

### Reference *H. armigera* genome data and assemblies

DNA was extracted from the offspring of a single pair of the GR laboratory colony of *H. armigera* maintained in Canberra. The colony derives from collections in the 1980s from cotton fields in the Namoi Valley in New South Wales, Australia, and has been maintained on a suitable laboratory diet since then. DNA extraction was performed from whole, late stage pupae using a standard phenol chloroform protocol.

Library construction and sequencing was performed at the Baylor College of Medicine, Human Genome Sequencing Center (BCM HGSC), Houston, TX, USA. Several different types of sequencing libraries were generated — a few for the 454 sequencing platform but most for the Illumina platform. Raw data were pre-processed to remove low-quality reads and bases.

An AllpathsLG [91] assembly of the Illumina data (from a 180-bp paired-end (PE) and 3-kb, 6-kb and 8-kb mate pair (MP) libraries) and a 20-kb MP 454 library produced a scaffold N50 of 1 Mb. This assembly, termed csiro4b, formed the basis for the final genome freeze, as described in Additional file 4: Section 13. Further AllpathsLG assemblies used different combinations and subsets of the available data as input (Additional file 4: Table S26). A Celera Assembler with the Best Overlap Graph (CABOG) [92] assembly of contigs was also made using selected 454 and Illumina data. These other assemblies were used in confirmation or repair of gene models during the annotation process described below. The csiro4b assembly was then corrected at 100 locations with sequences identified as giving correct gene models from the other assemblies or transcriptome data, to generate the patched genome freeze csiro4bp. Further details of the GR colony, sequencing data and assembly methods are provided in Additional file 4: Section 13.

### *H. armigera* transcriptomics

Material from the GR colony was also used in the two major transcriptomics experiments, either whole organisms or dissected tissues for the tissue/developmental transcriptome atlas (see Additional file 4: Table S8) and whole fourth instar larvae for the experiment investigating the effects of diet (see below). Total RNA from all samples was extracted by grinding the material in ‘RLT’ solution, and RNA from the equivalent of 30 mg of tissue from each sample was then purified using an RNeasy mini kit (Qiagen, Victoria, Australia). RNA was eluted in water, with a minimum yield of 40 μg. RNA quality and quantity in an aliquot of each sample were determined by electrophoresis on an Agilent 2100 Bioanalyser (Agilent Technologies, Santa Clara, CA, USA) chip system and by UV absorption on a NanoDrop spectrophotometer ND-1000 (ThermoFisher Scientific, Waltham, MA, USA). The remaining RNA from each sample was precipitated with ethanol and sodium acetate and stored at –80 °C. Library construction and RNA sequencing were done at BCM HGSC.

An initial comprehensive transcriptome assembly using all the RNA-seq reads from both these transcriptomics experiments was generated using TopHat and Cufflinks [93, 94]. A second assembly, following trimming of PE reads (100 b) to 80 b using the FASTX-Toolkit (http://hannonlab.cshl.edu/fastx_toolkit), was then generated using Trinity [95], as described in detail in Kanost et al. [40].

MicroRNAs were sequenced from total RNA harvested from first instar larvae, the midguts of fourth instar larvae and from pupae, again all from the GR colony. After phenol/chloroform extraction and ethanol precipitation, the total RNA was resuspended in diethyl pyrocarbonate (DEPC)-treated MQ water, quantified with a NanoDrop Spectrophotometer ND-1000 and quality checked in an Agilent 2100 Bioanalyser. About 100 ng of total RNA was denatured at 70 °C for 1 min, followed by chilling on ice and Illumina sequencing (Geneworks, Adelaide, Australia).

### Annotation of the *H. armigera* genome

This step involved automated annotation with MAKER and Program to Assemble Spliced Alignments (PASA2). The first step in our automated annotation of csiro4b involved the MAKER pipeline [96]. The Augustus [97], Semi-HMM-based Nucleic Acid Parser (SNAP) [98] and GeneMark [99] ab initio gene prediction tools incorporated in MAKER were trained using a set of manually curated genes (see below). As detailed in Additional file 4: Section 13, the process was then repeated several times with inclusion of the RNA-seq assemblies and additional evidence databases consisting of gene sets predicted from other insect genomes. A customised method using the OrthoMCL [100] and CD-HIT [101] pipelines was then used to assess the quality of the predicted genes from each of the nine MAKER runs and to consolidate the genes from the various MAKER runs into a consensus set (Additional file 4: Section 13). The nine MAKER runs and OrthoMCL + CD-HIT approach together produced 18,636 distinct proteins.

Many protein models produced by MAKER resulted from fusions of adjacent duplicated genes. However, these problems were resolved in a comprehensive re-annotation using JAMg (http://jamg.sourceforge.net) as per Papanicolaou et al. [102]. Briefly, the MAKER, protein domain evidence, Kassiopeia [103], GeneMark, RNA-seq coverage, intron-spanning cDNA reads and previously manually curated genes were provided as evidence with respectively increasing weight to the Augustus de novo gene predictor. This multi-layered output was then reconciled using EVidenceModeler [104] and annotated for untranslated regions (UTRs) and alternative transcription using the RNA-seq data and PASA2 [104, 105], yielding 22,818 transcript models. A reference unigene set (i.e. containing a single protein model for each locus), termed the official gene set 1 (OGS1; Additional file 4: Section 13), was derived from this. Finally, 1088 manually annotated gene models for specific gene families (see below) replaced the corresponding automated gene models, giving OGS2. Scipio [106] was used to derive genome location coordinates for the manually annotated gene models.

#### Functional annotation of gene models in key families

The automatically generated gene models for the key detoxification, digestion and chemosensory gene families were cross-checked and manually curated using all available sequences, cDNAs and gene models. For the detoxification and digestion families this included the use of a specially developed gene finding and alignment pipeline (Additional file 4: Section 13); where the models generated differed from those in the final assemblies, the latter were then patched appropriately. Other families listed in the comprehensive family annotation table (Additional file 2: Table S2) were annotated based on either the use of custom perl scripts to identify proteins with specific motifs (e.g. the cuticular proteins) or by the semi-automated screening of Basic Local Alignment Search Tool (BLAST)-derived annotations.

#### Whole genome functional annotations

The OGS2 protein sequences were analysed using a custom version of the InterProScan pipeline [107], including the GO [108], Pfam [109], PROSITE [110] and Simple Modular Architecture Research Tool (SMART) [111] annotations. Proteins carrying relevant domains identified by these analyses were flagged for confirmation as members of specific gene families. GO term assignments were extensively used in custom pipelines built on the GO database and in the Biological Networks Gene Ontology tool (BiNGO) plugin [112] for Cytoscape [113]. To analyse functional enrichment in specific gene sets, GO terms were summarised through semantic similarity filtering and visualised using REVIGO [114].

#### Repeats and microRNAs

Repeat sequences in the genome were identified using RepeatModeler [115]. All previously identified lepidopteran repeats were first obtained from RepBase and used to query the *H. armigera* genome. These repeats were then used as known repeat libraries for 10 iterations of RepeatModeler runs using RepeatScout and rmblast. The repeats recovered were then masked in the *H. armigera* genome using RepeatMasker. RNA sequence data for miRNA analysis were first processed using custom perl scripts, and then miRNAs were predicted using miRDeep2 [116]. Further analysis against known miRNAs from other insects was undertaken using miRBase19 [117].

### Reference *H. zea* genome and transcriptome assemblies and annotation

Genome sequencing for *H. zea* used DNA extracted from pupae of a laboratory colony established prior to introduction of transgenic Bt crops and maintained without infusing feral insects for at least 25 years [118]. This laboratory colony was highly susceptible to all Bt toxins compared to feral *H. zea* [118–120]. Virgin males and females were used to inbreed the insects through three generations of single-pair matings. Male pupae of the final generation were used to obtain high molecular weight genomic DNA for preparing Illumina sequencing libraries. Libraries were constructed and sequenced as for *H. armigera* above.

An AllpathsLG assembly of the Illumina data produced an N50 of 196 kb (Hz-csiro5 in Additional file 4: Table S27). Again, a series of further AllpathsLG assemblies used different combinations and subsets of the input data as listed in Additional file 4: Table S27. Correction and patching of Hz-csiro5 to produce the final *H. zea* genome freeze (hz5p5) is described in Additional file 4: Section 13, together with further details of the *H. zea* colony and the sequencing data and assembly methods used.

Transcriptome data used in annotation of the *H. zea* genome included a preliminary assembly of 454 and Illumina RNA-seq data. All 454 data were obtained from a pool of RNA starting with 24–48 h embryos, all larval stages, pupae and adult males and females. The Illumina RNA-seq data were from 24–48 h embryos and third instar larvae. The larvae were treated with sublethal doses of Cry1Ac, novaluron, cypermethrin and Orthene to induce genes involved in xenobiotic degradation that may not normally be expressed. The 454 libraries were normalised. RNA sequence data were assembled with Trinity (version trinityrnaseq_r20140413p1) using genome-guided and de novo assembly methods as above for *H. armigera*.

The *H. zea* genomes were screened using the *H. armigera* OGS2 gene model protein sequences and Scipio [106] to identify the best possible gene models for *H. zea*. See Additional file 4: Section 13 for details.

### Orthology and evolutionary analyses of target gene families

Gene models for the detoxification- and digestion-related gene families in *H. armigera* and *H. zea* were obtained as described above. For other species analysed in Table 2, the automatically generated gene models and official gene sets were cross-checked and manually curated by domain specialists using available sequences, cDNAs and gene models generated by the EXONERATE-based dedicated pipeline. Current annotations of *B. mori* and *M. sexta* members of these families were cross-checked and in some cases revised by a similar procedure, albeit in this case the few models that differed from those in the genome assembly were not patched into that assembly. All our final gene models for these families for the three species are summarised in Additional file 6: Table S5. Other families of interest whose gene models are listed in this table were identified and annotated either using custom perl scripts to screen for proteins with specific motifs (e.g. the cuticular proteins) or by semi-automated screening of BLAST-derived annotations.

The phylogenetic methods used to analyse the evolutionary processes operating in most gene families were as described in the Methods for Supplementary Figures 19–21 of Kanost et al. [40]. Briefly, we used multiple sequence alignment software (MAFFT) [121] with the linsi option to make a multiple sequence alignment, which we then masked for sites with more than 50% gaps or ambiguous characters. Phylogenetic analyses were then carried out using IQ-TREE [122], which implements an ultrafast bootstrap method [123] and ModelFinder, a new model-selection method that greatly improves the accuracy of phylogenetic estimates [124]. Having found the optimal model for each family, we then inferred the most likely tree for it using IQ-TREE, with bootstrap scores inferred using the ultrafast bootstrap method. Two other phylogenetic methods were used for a few data sets. PhyML [125] was used for some smaller data sets, and for the lower quality GR data set Randomised Axelerated Maximum Likelihood (RAxML) [126] was used. Trees were illustrated using the R package ggtree [127].

Divergence dating analyses among subsets of gene families within or across different species or lines used the Bayesian MCMC method in BEAST v2.4.3 [55]. Protein sequences aligned using MAFFT as described above for the phylogenetic analyses were used to inform coalignment of nucleotide sequences using a custom perl script. Where necessary, the site models were unlinked to enable different evolutionary rates at each locus (as determined in IQ-TREE above), but clock and tree models were linked so that they would not vary among locus partitions. An XML input file was then generated for BEAST v2.4.3 using BEAUti v2.4.3. The prior for *t*_MRCA_ (time to the Most Recent Common Ancestor) and root height were set at a lognormal distribution, with a mean of ln (1.5) and a standard deviation of 0.01. A strict molecular clock with a uniform distribution was applied using the mutation rate determined for *H. melpomene* of 2.9 × 10^–9^ (95% confidence interval, 1.3 × 10^−9^ through 5.5 × 10^−9^) substitutions per site per generation [128]. A generation time of 0.25 year corresponding to the midrange defined by Fitt [67] for subtropical and temperate regions was used for some analyses. Trees were annotated in TreeAnnotator v2.4.3 [129] and visualised in FigTree v1.4.2 [130].

Relative rate tests of *H. armigera* genes used the nearest paralogues shown in the phylogenetic trees for each family in Additional file 4: Sections 1–8. Protein sequences aligned using MAFFT as described above for the phylogenetic analyses were used to inform coalignment of nucleotide sequences using a custom perl script. Tajima’s relative rate tests [131] were done in Molecular Evolutionary Genetics Analysis (MEGA) software [132].

### Tissue/developmental transcriptomic atlas

Thirty-one GR samples reared on standard diet were collected for this analysis, four from whole organisms of specific life stages and 27 from tissues or body parts of feeding fifth instar larvae or adults. Details of the samples are given in Additional file 4: Table S8. RNA and library preparation and sequencing were as described above.

### Diet transcriptomics experiment

Patterns of gene expression were compared between larvae raised on different host plants. The plants were selected to maximise the diversity of responses that might be observed [64]. The set comprised one monocot, maize, *Zea mays* (larval RNA libraries M-3, GenBank BioSamples 6608687-9), and plants from four dicotyledonous plant families: Malvaceae, cotton, *Gossypium hirsutum* (larval RNA libraries Ct1-3, GenBank BioSamples 6608702-4); Brassicaceae, thale cress, *Arabidopsis thaliana* (larval RNA libraries AR1-3, GenBank BioSamples 6608666-8); Fabaceae, green bean, *Phaseolus vulgaris* (larval RNA libraries GB1-3, GenBank BioSamples 6608675-7) and Solanaceae, tobacco, *Nicotiana tabacum* (larval RNA libraries Tb1-3, GenBank BioSamples 6608696-8), tomato, *Lycopersicon esculentum* (larval RNA libraries TM1-3, GenBank BioSamples 6608699-701) and hot pepper, *Capsicum frutescens* (larval RNA libraries Hp1-3, GenBank BioSamples 6608678-80). For reference, larvae were also raised on a standard laboratory diet [133, 134] (larval RNA libraries Sd1-3, GenBank BioSamples 6608693-5).

About 10 larvae from the GR colony were transferred to plants or the laboratory diet in triplicate within 24 h of hatching and without exposure to any previous diet. Each replicate consisted of one pot containing either a single plant for the larger species or several plants for the smaller species. Larvae were transferred to plants when flowers had started to form but before any fruit was present. The plants were grown under the same glasshouse conditions, and each of the three replicates used larvae from a different cohort of the laboratory culture. As pointed out by others [64, 135], larvae raised on an artificial diet prior to such a host-response experiment are seen as offering the advantage of not being primed for any particular plant host.

In order to harvest all larvae at a comparable developmental stage irrespective of the host plant, six larvae from each replicate were collected from the plants when they had returned to feeding one day after moulting to the fourth instar. The time taken to reach this stage was noted, and the larvae were weighed; they were then immediately cut with dissecting scissors into three or four pieces. Their RNA was preserved by immediately dropping the pieces into RNAlater solution (Ambion, Austin, TX, USA), which was held initially on ice to allow the solution to diffuse into the tissue and then frozen at –80 °C.

Total RNA was prepared from the six larvae comprising each replicate as per the methods described above, except that the libraries for sequencing were made at the United States Department of Agriculture-Agricultural Research Service (USDA-ARS, Stoneville, MS, USA). RNA sequencing was done at BCM HGSC as above.

It was not possible to undertake parallel diet transcriptomic experiments on *H. zea* in this study, since it is not found in Australia and therefore subject to stringent biosecurity quarantine prohibitions. Such a follow-up study would therefore need to be undertaken in a country known to harbour both species.

### Transcriptome analyses

Sequencing reads were cleaned using Trimmomatic [136] to remove adapter sequence and low-quality reads. Passing reads were aligned to the *H. armigera* csiro4bp assembly with the subread aligner implemented in the Rsubread package [137]. A maximum of three mismatches were allowed in the alignment, and the best scoring alignment for each read was reported. The numbers of reads per library that overlapped with the predicted transcripts described above were summarised at the gene level with featureCounts [138]. To be considered for further analysis, a minimum level of five reads per million across three libraries was required. In the case of the developmental/tissue atlas, an alternative inclusion criterion of at least 20 reads per million in at least one library was allowed to capture genes that may have been expressed in only a single life stage or tissue sampled. These criteria resulted in 13,099 and 11,213 genes being considered expressed in the developmental/tissue atlas and host use analysis, respectively, with a total of 13,689 unique genes across the two data sets.

Read counts were normalised between samples using the trimmed mean of *M*-values method [139] and converted to log2 counts per million values (log2cpm) with associated quality weights using the voom-limma pipeline [140]. For the host use experiment, gene expression was modelled simply as a factor of the diet the larvae were raised on. To remove the effects of unwanted variation due to latent variables not correlated with larval diet, three surrogate variables [141, 142] were estimated from the data and included in the expression model. Genes with a significant difference in expression relative to the control diet (false discovery rate adjusted *p* value less than 0.05) and a log2 fold change in expression greater than 1.5 were considered to be diet-responsive.

For a broader analysis of gene expression, we constructed gene co-expression networks from our expression data to identify sets of genes that show correlated expression profiles. Additional filtering criteria were used to ensure that only genes that displayed some level of expression variation were considered in the network construction. The criteria for inclusion were that the mean log2cpm expression value had to be greater than 1 and the standard deviation of the value had to be greater than 0.5. Similar to the previous filtering step, an additional acceptance criterion was included for the tissue data set to allow for genes expressed in only a small number of libraries to be included. The extra criterion for this data set was that any gene with a standard deviation greater than 2 was included. Unsigned, weighted correlation networks were produced from both the diet and tissue/developmental data sets with the R package weighted correlation network analysis (WGCNA) [143]. The power parameter used for each network was 11 and 8, respectively, chosen as the lowest value with a scale-free topology fit *R* squared greater than 0.85. Gene expression modules were determined from a topological overlap matrix, and modules with highly correlated eigengene expression patterns (>0.85) were merged.

### Resequencing experiments and analyses

Three additional *H. armigera* lines, one from Africa and two from China, and four additional *H. zea* individuals, all from the USA, were sequenced as a database for various population genomic analyses. The African *H. armigera* strain, SCD, originated from the Ivory Coast in the 1970s and was maintained in the laboratory without exposure to insecticides or Bt toxins for more than 130 generations of mass mating before DNA preparation. One Chinese line, SW, was founded in 2012 from 150 moths collected in cotton fields from Shawan in the Xinjiang Uygur Autonomous Region. SW was reared for 17 mass-mating generations in the laboratory without exposure to insecticides or Bt toxins before DNA preparation. The other Chinese line, AY, was started from a single pair of moths collected in 2011 from Anyang in Henan Province [79]. AY, which survived the diagnostic Cry1Ac concentration of 1 μg/cm^2^, was reared for more than 30 generations before DNA preparation. For these SCD, SW and AY lines of *H. armigera*, DNA was prepared from individual male pupae. The DNA was then used in construction of 500b PE libraries which were quantified and sequenced on an Illumina HiSeq2000 platform at the Beijing Genomics Institute (BGI, Shenzhen, China) using standard in-house protocols.

The four *H. zea* individuals had been collected as larvae from wild host plants in Bolivar County, Mississippi. DNA was prepared from their thoraces when they emerged as adults and used for constructing sequencing libraries using an Illumina Nextera library construction kit. Genomic DNA libraries were size fractionated on a Pippin Prep instrument (Sage Science Inc., Beverly, MA, USA) to obtain 550 ± 20 b fragments (inset size 400–450 b) and quantified using a KAPA library quantification kit (KAPA Biosystems, Wilmington, MA, USA). An equimolar pool of the four libraries was sequenced on an Illumina HiSeq2500 instrument at the USDA-ARS Genomics and Bioinformatics Research Unit, Stoneville, MS, USA.

Sequence reads from each line or individual were error corrected using Blue [144] and aligned to the *H. armigera* reference genome with the Genomic Short-read Nucleotide Alignment Program (GSNAP) [145]. To ensure that the choice of reference genome did not influence our results, reciprocal alignments of all lines or individuals against the *H. zea* reference genome were also performed. Using the Genome Analysis Toolkit (GATK) [146] we applied duplicate removal and local realignment around indels followed by SNP genotyping using standard hard filtering parameters as per the GATK Best Practices recommendations [147, 148]. As an extra step to allow us to better compare sequences from the two species, we imposed the additional filtering criterion that a variant must be genotyped across all sequenced lines or individuals to be included in our analysis.

Genetic relationships between *H. armigera* and *H. zea* were examined using MDS on SNP data files generated for all sequences in our data set, including both the *H. armigera* and *H. zea* reference sequences.

Coalescence analysis was performed on 16 loci (see Additional file 3: Figure S5; Additional files 11 and 12), representing genes present across all of the *H. armigera* and *H. zea* samples, including both reference sequences, as well as in the outgroup *H. punctigera* (i.e. *n* = 10 for each locus). The set of loci selected for this analysis were one-to-one orthologues across all samples, with only up to 1% of sites in a given locus being soft-masked (i.e. for sequencing coverage <10×) or heterozygous. These criteria resulted in a set of well-conserved loci across these 10 samples being used subsequently in the coalescence analysis in BEAST v2.4.3 [149]. All loci were first aligned independently using the linsi option in MAFFT v7.182 [121]. IQ-TREE v1.4.1 [122] was then used with the -m TESTNEWONLY option to determine the best-fit evolutionary rate model for each locus. BEAUti v2.4.3 (StarBeast template) was used to generate a BEAST XML input file, setting individual rate models for each locus as identified in IQ-TREE, and unlinking tree models. A Yule process for the multi-species coalescent, and a ‘linear with constant root’ population size prior were the parameters selected to generate the BEAST input file. The analysis was run for >100 × 10^6^ MCMC chains to reach convergence of tree likelihoods and to get effective sample size (ESS) values >200 (assessed in Tracer v1.6.0 [150]). The BEAST analysis produced an overall species tree for *H. armigera*, *H. zea* and *H. punctigera*, as well as individual gene trees for each locus. The latter were fed to DensiTree v2.2.2 [55] to check whether the topology is consistent with the overall species tree. In instances of conflict between the gene and species trees, we investigated the loci in question to assess whether we could find evidence for incomplete lineage sorting between *H. armigera* and *H.* ze*a*.

The historical effective population sizes and their changes over time were estimated for *H. armigera* and *H. zea* using the Bayesian skyline plot method as implemented in BEAST v1.8.2 [151]. The data sets used were genome-wide SNPs called separately for each of the following samples: for *H. armigera*, sequences from the AY, SW and SCD lines against the *H. armigera* reference genome; and for *H. zea*, the four individuals described above against the *H. zea* reference genome. The two sets of samples were also called against the other species’ genome as a control. MCMC samples were based on 10^8^ generations, logging every 1000 steps, with the first 10^7^ generations discarded as burn-in. We used a piecewise linear skyline model, an HKY substitution model and a strict clock with the mean substitution rate as determined for *H. melpomene* of 2.9 × 10^–9^ (95% confidence interval, 1.3 × 10^–9^ through 5.5 × 10^–9^) substitutions per site per generation [128].

To examine synonymous and non-synonymous diversity between the two species, we analysed nucleotide diversity (pi) in our resequenced *H. armigera* and *H. zea* samples (i.e. excluding the reference strains). We explored mean genomic diversity further by examining all polymorphic sites (i.e. ~8.2 M SNPs called across the genome). Diversity measurements only counted windows where there were a minimum of 10 SNPs per 10-kb genome window.

## Additional files

Additional file 1: Table S1. Names and locations for all *H. armigera* and *H. zea* genes annotated in their respective official gene set (*OGS*). (XLSX 2251 kb)

Additional file 2: Table S2. GO numbers assigned to *H. armigera* and *B. mori* genes. (XLSX 1573 kb)

Additional file 3: Figure S1. GO term analyses of gene gain/loss events in *H. armigera* vs *B. mori* and *H. zea* **Figure S2.** Synteny between the *Helicoverpa* assemblies and with *B. mori*. **Figure S3.** Principal component analysis of the most variably expressed genes across the different diets. **Figure S4.** GO terms enriched in the three key co-expression modules from the diet transcriptomics experiment. **Figure S5.** Coalescence species tree and dating analysis. **Figure S6.** Genome-wide nucleotide diversity estimates for the resequenced *H. armigera* and *H. zea* lines species using the *H. armigera* (A) and *H. zea* (B) reference sequences. **Figure S7.** Genome-wide synonymous and non-synonymous nucleotide diversity estimates and the correlations between them for the resequenced *H. armigera* and *H. zea* lines using the *H. armigera* reference sequence. **Figure S8.** Gene numbers in major detoxification and gustatory response families for nine lepidopterans. **Figure S26.** Transcriptome profiles of the *H. armigera* GRs on different hosts. **Figure S29.** Transcriptome profile of genes with GO growth annotation. **Figure S30.** Transcriptome profile of 240 transcription factors. **Figure S31.** Transcriptome profile of genes for cytoplasmic ribosomal proteins. **Figure S32.** Transcriptome profile of genes for cuticular proteins. (PDF 2725 kb)

Additional file 4: Table S3. Repeats recovered from the *H. armigera* and *H. zea* genomes. **Table S6.** Most recent expansions within major gene families of *H. armigera* analysed using *BEAST. **Table S7.** Details of Tajima’s relative rate tests on closely related *H. armigera* paralogues in the major detoxification and digestion gene families and GRs, together with the numbers of genes in the relevant clades missing in the *H. zea* assembly. **Table S8.** Details of the tissues and life stages sampled for the transcriptome atlas. **Table S10a.** Characteristics of the 37 diet transcriptome co-expression modules in term of enrichment for various groups of genes. Section 1: Detailed analysis of P450s in *H armigera*, *H. zea*, *B. mori*, *M. sexta* and *P. xylostella.* Section 2: Detailed analysis of CCEs in *H armigera*, *H. zea*, *B. mori*, *M. sexta* and *P. xylostella.* Section 3: Detailed analysis of GSTs in *H armigera*, *H. zea*, *B. mori, M. sexta* and *P. xylostella.* Section 4: Detailed analysis of UGTs in *H armigera*, *H. zea*, *B. mori* and *M. sexta.* Section 5: Detailed analysis of ABC transporters in *H. armigera*, *H. zea*, *M. sexta* and *P. xylostella.* Section 6: Detailed analysis of midgut serine proteases in *H. armigera*, *H. zea* and *B. mori.* Section 7: Detailed analysis of lipases in *H armigera*, *H. zea* and *B. mori.* Section 8: Detailed analysis of GR genes in *H. armigera* and *H. zea.* Section 9: Detailed analysis of stress response and immunity genes in *H. armigera* and *H. zea.* Section 10: Detailed analysis of some gene families related to larval growth. Section 11: Additional insights from diet transcriptome modules. Section 12. Detailed analysis of genes related to insecticide resistance in *H. armigera* and *H. zea.* Section 13. Detailed methods for the *Helicoverpa* genome assemblies and annotation. (DOCX 11091 kb)

Additional file 5: Table S4. MicroRNAs in *H. armigera* and *H. zea.* (XLSX 37 kb)

Additional file 6: Table S5. Details of all the *H. armigera* and *H. zea* gene models that were manually curated or manually allocated to gene families or functional groups. (XLSX 245 kb)

Additional file 7: Sequence alignment files for gene families analysed in Additional file 4: Table S6. (XLSX 12 kb)

Additional file 8: Table S9. Complete list of 11,213 H. armigera genes for which transcriptome data were analysed. (ZIP 116 kb)

Additional file 9: Table S10b. Details of the 37 diet transcriptome co-expression modules in term of enrichment for various groups of genes. (XLSX 625 kb)

Additional file 10: Table S23. List of 129 *H. armigera* transcription factors (*TFs*) mapped to *D. melanogaster* TFs in networks. (ZIP 19 kb)

Additional file 11: Sequence alignment files for genes analysed in Additional file 3: Figure S5. (TAB 2 kb)

Additional file 12: Phylogenetic trees for genes analysed in Additional file 3: Figure S5. (ZIP 21 kb)

## References

[1] Zayed A, Packer L, Grixti JC, Ruz L, Owen RE, Toro H (2005). Increased genetic differentiation in a specialist versus a generalist bee: implications for conservation. Conserv Genet..

[2] Ali JG, Agrawal AA (2012). Specialist versus generalist insect herbivores and plant defense. Trends Plant Sci..

[3] Berger D, Walters RJ, Blanckenhorn WU (2014). Experimental evolution for generalists and specialists reveals multivariate genetic constraints on thermal reaction norms. J Evol Biol..

[4] Liu Z, Liu G, Hailer F, Orozco-terWengel P, Tan X, Tian J (2016). Dietary specialization drives multiple independent losses and gains in the bitter taste gene repertoire of Laurasiatherian mammals. Frontiers Zool..

[5] Yoshida K, Saunders DGO, Mitsuoka C, Natsume S, Kosugi S, Saitoh H (2016). Host specialization of the blast fungus Magnaporthe oryzae is associated with dynamic gain and loss of genes linked to transposable elements. BMC Genomics..

[6] Hughes AL (1994). The evolution of functionally novel proteins after gene duplication. Proc Roy Soc Lond B: Biol Sci..

[7] Lynch M, Conery JS (2000). The evolutionary fate and consequences of duplicate genes. Science..

[8] Carroll SB (2000). Endless forms: the evolution of gene regulation and morphological diversity. Cell..

[9] Gilbert SF, Bosch TCG, Ledon-Rettig C (2015). Eco-Evo-Devo: developmental symbiosis and developmental plasticity as evolutionary agents. Nat Rev Genet..

[10] Huang Y, Agrawal AF (2016). Experimental evolution of gene expression and plasticity in alternative selective regimes. PLoS Genet..

[11] Rane RV, Walsh TK, Pearce SL, Jermiin LS, Gordon KHJ, Richards S (2016). Are feeding preferences and insecticide resistance associated with the size of detoxifying enzyme families in insect herbivores?. Curr Opin Insect Sci..

[12] Cho S, Mitchell A, Mitter C, Regier J, Matthews M, Robertson R (2008). Molecular phylogenetics of heliothine moths (Lepidoptera: Noctuidae: Heliothinae), with comments on the evolution of host range and pest status. Syst Entomol..

[13] Gordon K, Tay WT, Collinge D, Williams A, Batterham P, Goldsmith MR, Marec F, Miller T (2010). Genetics and molecular biology of the major crop pest genus Helicoverpa. Molecular biology and genetics of the Lepidoptera.

[14] Czepak C, Albernaz KC, Vivan LM, Guimarães HO, Carvalhais T (2013). First reported occurrence of Helicoverpa armigera (Hübner) (Lepidoptera: Noctuidae) in Brazil. Pesq Agro Trop..

[15] Tay WT, Soria MF, Walsh T, Thomazoni D, Silvie P, Behere GT (2013). A brave new world for an old world pest: Helicoverpa armigera (Lepidoptera: Noctuidae) in Brazil. PLoS ONE..

[16] Tay WT, Walsh TK, Downes S, Anderson C, Jermiin LS, Wong TFK (2017). Mitochondrial DNA and trade data support multiple origins of *Helicoverpa armigera* (Lepidoptera, Noctuidae) in Brazil. Sci Rep.

[17] Europhyte. Interceptions of harmful organisms in commodities imported into the EU Member States and Switzerland. 2014. http://ec.europa.eu/food/sites/food/files/plant/docs/ph_biosec_europhyt-interceptions-2014_summary.pdf.

[18] APHIS (Animal and Plant Health Inspection Service). Detection of Old World bollworm (*Helicoverpa armigera*) in Florida. 2015. p. 1–2. http://www.aphis.usda.gov/plant_health/plant_pest_info/owb/downloads/DA-2015-43.pdf.

[19] Kriticos DJ, Ota N, Hutchison WD, Beddow J, Walsh T, Tay WT (2015). The potential distribution of invading Helicoverpa armigera in North America: is it just a matter of time?. PLoS ONE..

[20] Leite NA, Alves-Pereira A, Correa AS, Zucchi MI, Omoto C (2014). Demographics and genetic variability of the New World bollworm (Helicoverpa zea) and the Old World bollworm (Helicoverpa armigera) in Brazil. PLoS ONE..

[21] Sosa-Gómez DR, Specht A, Paula-Moraes SV, Lopes-Lima A, Yano SAC, Micheli A (2016). Timeline and geographical distribution of Helicoverpa armigera (Hübner) (Lepidoptera, Noctuidae: Heliothinae) in Brazil. Rev Brasil Entomol..

[22] Sharma HC (2005). Heliothis/Helicoverpa management: emerging trends and strategies for future research.

[23] Hardwick DF (1965). The corn earworm complex. Mem Ent Soc Canad..

[24] Pogue MG (2004). A new synonym of Helicoverpa zea (Boddie) and differentiation of adult males of H. zea and H. armigera (Hubner) (Lepidoptera : Noctuidae : Heliothinae). Ann Ent Soc Amer.

[25] Mallet J, Korman A, Heckel DG, King P (1993). Biochemical genetics of Heliothis and Helicoverpa (Lepidoptera, Noctuidae) and evidence for a founder event in Helicoverpa zea. Ann Ent Soc Amer..

[26] Behere GT, Tay WT, Russell DA, Heckel DG, Appleton BR, Kranthi KR (2007). Mitochondrial DNA analysis of field populations of *Helicoverpa armigera* (Lepidoptera: Noctuidae) and of its relationship to *H. zea*. BMC Evol Biol.

[27] Cunningham JP, Zalucki MP. Understanding heliothine (Lepidoptera: Heliothinae) pests: what is a host plant? J Econ Entomol. 2014;107:881–96. 10.1603/ec14036 25026644

[28] Armes NJ, Jadhav DR, Bond GS, King ABS. Insecticide resistance in Helicoverpa armigera in South India. Pest Manag Sci. 1992;34:355–64.

[29] Daly JC. Ecology and genetics of insecticide resistance in Helicoverpa armigera: Interactions between selection and gene flow. Genetica. 1993;90:217–26.

[30] McCaffery AR. Resistance to insecticides in heliothine Lepidoptera: a global view. Phil Trans Roy Soc Lond B Biol Sci. 1998;353:1735–50.

[31] Xu X, Yu L, Wu Y. Disruption of a cadherin gene associated with resistance to Cry1Ac {delta}-endotoxin of Bacillus thuringiensis in Helicoverpa armigera. Appl Environ Microbiol. 2005;71:948–54. 10.1128/AEM.71.2.948-954.2005 PMC546791 15691952

[32] Tay WT, Mahon RJ, Heckel DG, Walsh TK, Downes S, James WJ, et al. Insect resistance to Bacillus thuringiensis toxin Cry2Ab is conferred by mutations in an ABC transporter subfamily A protein. PLoS Genet. 2015;11:e1005534. 10.1371/journal.pgen.1005534 PMC4652872 26583651

[33] Sparks TC. Development of insecticide resistance in Heliothis zea and Heliothis virescens in North America. Bull Ent Soc Amer. 1981;27:186–92.

[34] Jacobson A, Foster R, Krupke C, Hutchison W, Pittendrigh B, Weinzierl R (2009). Resistance to pyrethroid insecticides in Helicoverpa zea (Lepidoptera: Noctuidae) in Indiana and Illinois. J Econ Entomol..

[35] Moar W, Dennehy T, Anilkumar K, Head G (2010). Bt resistance in Helicoverpa zea (Boddie): from biology to monitoring. Southwest Entomol..

[36] Luttrell RG, Jackson RE (2012). Helicoverpa zea and Bt cotton in the United States. GM Crops Food..

[37] Anderson CJ, Tay WT, McGaughran A, Gordon K, Walsh TK (2016). Population structure and gene flow in the global pest, Helicoverpa armigera. Molec Ecol..

[38] Coates BS, Abel CA, Perera OP (2016). Estimation of long terminal repeat element content in the Helicoverpa zea genome from high-throughput sequencing of bacterial artificial chromosome pools. Genome..

[39] The International Silkworm Genome Consortium (2008). The genome of a lepidopteran model insect, the silkworm Bombyx mori. Insect Biochem Molec Biol.

[40] Kanost MR, Arrese EL, Cao X, Chen Y-R, Chellapilla S, Goldsmith MR (2016). Multifaceted biological insights from a draft genome sequence of the tobacco hornworm moth, Manduca sexta. Insect Biochem Molec Biol..

[41] Simão FA, Waterhouse RM, Ioannidis P, Kriventseva EV, Zdobnov EM (2015). BUSCO: assessing genome assembly and annotation completeness with single-copy orthologs. Bioinformatics..

[42] Wheeler TJ, Clements J, Eddy SR, Hubley R, Jones TA, Jurka J (2013). Dfam: a database of repetitive DNA based on profile hidden Markov models. Nucleic Acids Res..

[43] He P-A, Nie Z, Chen J, Chen J, Lv Z, Sheng Q (2008). Identification and characteristics of microRNAs from Bombyx mori. BMC Genomics..

[44] Kozomara A, Griffiths-Jones S (2011). miRBase: integrating microRNA annotation and deep-sequencing data. Nucleic Acids Res.

[45] Wang X, Tang S-M, Shen X-J (2014). Overview of research on Bombyx mori microRNA. J Insect Sci..

[46] Ge X, Zhang Y, Jiang J, Zhong Y, Yang X, Li Z (2013). Identification of microRNAs in Helicoverpa armigera and Spodoptera litura based on deep sequencing and homology analysis. Int J Biol Sci..

[47] Lomate PR, Mahajan NS, Kale SM, Gupta VS, Giri AP (2014). Identification and expression profiling of Helicoverpa armigera microRNAs and their possible role in the regulation of digestive protease genes. Insect Biochem Molec Biol..

[48] Beldade P, Saenko SV, Pul N, Long AD (2009). A gene-based linkage map for Bicyclus anynana butterflies allows for a comprehensive analysis of synteny with the lepidopteran reference genome. PLoS Genet..

[49] d’Alencon E, Sezutsu H, Legeai F, Permal E, Bernard-Samain S, Gimenez S (2010). Extensive synteny conservation of holocentric chromosomes in Lepidoptera despite high rates of local genome rearrangements. Proc Natl Acad Sci U S A..

[50] Sahara K, Yoshido A, Shibata F, Fujikawa-Kojima N, Okabe T, Tanaka-Okuyama M (2013). FISH identification of Helicoverpa armigera and Mamestra brassicae chromosomes by BAC and fosmid probes. Insect Biochem Molec Biol..

[51] Feyereisen R, Gilbert LI, Iatrou K, Gill SS (2005). Insect cytochrome P450. Comprehensive molecular insect science. Vol 4, Biochemistry and molecular biology.

[52] Oakeshott JG, Claudianos C, Campbell PM, Newcomb RD, Russell RJ, Gilbert LI, Iatrou K, Gill SS (2005). Biochemical genetics and genomics of insect esterases. Comprehensive molecular insect science. Vol. 5, Pharmacology.

[53] Ranson H, Hemingway J, Gilbert LI, Iatrou K, Gill SS (2005). Glutathione transferases. Comprehensive molecular insect science. Vol. 5, Pharmacology.

[54] Xu W, Papanicolaou A, Zhang H-J, Anderson A (2016). Expansion of a bitter taste receptor family in a polyphagous insect herbivore. Sci Rep..

[55] Bouckaert R, Heled J (2014). DensiTree 2: seeing trees through the forest. bioRxiv. Cold Spring Harbor Labs Journals.

[56] Neunzig HH (1963). Wild host plants of the corn earworm and the tobacco budworm in eastern North Carolina. J Econ Entomol..

[57] Matthews M (1999). Heliothine moths of Australia.

[58] Sudbrink DL, Grant JF (1995). Wild host plants of Helicoverpa zea and Heliothis virescens (Lepidoptera: Noctuidae) in eastern Tennessee. Environ Entomol..

[59] Blanco CA, Teran-Vargas AP, Lopez JDJ, Kauffman JV, Wei X (2007). Densities of Heliothis virescens and Helicoverpa zea (Lepidoptera: Noctuidae) in three plant hosts. Florida Entomol..

[60] de Lange ES, Balmer D, Mauch-Mani B, Turlings TCJ (2014). Insect and pathogen attack and resistance in maize and its wild ancestors, the teosintes. New Phytol..

[61] Olmstead DL, Nault BA, Shelton AM (2016). Biology, ecology, and evolving management of Helicoverpa zea (Lepidoptera: Noctuidae) in sweet corn in the United States. J Econ Entomol..

[62] Manjunath TM, Bhatnagar VS, Pawar CS, Sithananthan S, King EG, Jackson RD (1989). Economic importance of *Heliothis* species in India and an assessment of their natural enemies and host plants. Proceedings of the Workshop on Biological Control of Heliothis: Increasing the Effectiveness of Natural Enemies.

[63] Fitt GP, Bailey WJ, Ridsdill-Smith J (1991). Host selection in the Heliothinae. Reproductive behavior of insects.

[64] Liu ZD, Li DM, Gong PY, Wu KJ (2004). Life table studies of the cotton bollworm, Helicoverpa armigera (Hubner) (Lepidoptera: Noctuidae), on different host plants. Environ Entomol..

[65] Mahon RJ, Downes SJ, James B (2012). Vip3A resistance alleles exist at high levels in Australian targets before release of cotton expressing this toxin. PLoS ONE..

[66] Christeller JT, Poulton J, Markwick NM, Simpson RM (2010). The effect of diet on the expression of lipase genes in the midgut of the lightbrown apple moth (Epiphyas postvittana Walker; Tortricidae). Insect Molec Biol..

[67] Fitt GP (1989). The ecology of Heliothis species in relation to agroecosystems. Annu Rev Entomol..

[68] Kimbrell DA, Beutler B (2001). The evolution and genetics of innate immunity. Nat Rev Genet..

[69] Powell JR, Caccone A, Amato GD, Yoon C (1986). Rates of nucleotide substitution in Drosophila mitochondrial-DNA and nuclear-DNA are similar. Proc Natl Acad Sci U S A..

[70] Song SV, Downes S, Parker T, Oakeshott JG, Robin C (2015). High nucleotide diversity and limited linkage disequilibrium in Helicoverpa armigera facilitates the detection of a selective sweep. Heredity..

[71] Yang Y, Li Y, Wu Y (2013). Current status of insecticide resistance in Helicoverpa armigera after 15 years of Bt cotton planting in China. J Econ Entomol..

[72] Farnsworth CA, Teese MG, Yuan G, Li Y, Scott C, Zhang X (2010). Esterase-based metabolic resistance to insecticides in heliothine and Spodopteran pests. J Pest Sci..

[73] Heckel DG, Goldsmith MR, Marec F (2009). Molecular genetics of insecticide resistance in Lepidoptera. Molecular biology and genetics of the Lepidoptera.

[74] Oakeshott JG, Farnsworth CA, East PD, Scott C, Han Y, Wu Y (2013). How many genetic options for evolving insecticide resistance in heliothine and Spodopteran pests?. Pest Manag Sci..

[75] Tabashnik BE (2015). ABCs of insect resistance to Bt. PLoS Genet..

[76] Joußen N, Agnolet S, Lorenz S, Schoene SE, Ellinger R, Schneider B (2012). Resistance of Australian Helicoverpa armigera to fenvalerate is due to the chimeric P450 enzyme CYP337B3. Proc Natl Acad Sci U S A..

[77] Xiao Y, Zhang T, Liu C, Heckel DG, Li X, Tabashnik BE (2014). Mis-splicing of the ABCC2 gene linked with Bt toxin resistance in Helicoverpa armigera. Sci Rep..

[78] Farnsworth CA. Esterases and *Helicoverpa armigera*; a study of the involvement of esterases in resistance to synthetic pyrethroids and Bt insecticides in the cotton bollworm *H. armigera*. PhD thesis. Canberra: Australian National University; 2014.

[79] Jin L, Wei Y, Zhang L, Yang Y, Tabashnik BE, Wu Y (2013). Dominant resistance to Bt cotton and minor cross-resistance to Bt toxin Cry2Ab in cotton bollworm from China. Evol Appl..

[80] Han Y, Wu S, Li Y, Liu J-W, Campbell PM, Farnsworth C (2012). Proteomic and molecular analyses of esterases associated with monocrotophos resistance in Helicoverpa armigera. Pestic Biochem Physiol..

[81] Rasool A, Joußen N, Lorenz S, Ellinger R, Schneider B, Khan SA (2014). An independent occurrence of the chimeric P450 enzyme CYP337B3 of Helicoverpa armigera confers cypermethrin resistance in Pakistan. Insect Biochem Molec Biol..

[82] Mao Y-B, Cai W-J, Wang J-W, Hong G-J, Tao X-Y, Wang L-J (2007). Silencing a cotton bollworm P450 monooxygenase gene by plant-mediated RNAi impairs larval tolerance of gossypol. Nat Biotechnol..

[83] Gordon KHJ, Waterhouse PM (2007). RNAi for insect-proof plants. Nat Biotech..

[84] Krempl C, Heidel-Fischer HM, Jimenez-Aleman GH, Reichelt M, Menezes RC, Boland W (2016). Gossypol toxicity and detoxification in Helicoverpa armigera and Heliothis virescens. Insect Biochem Molec Biol..

[85] Gassmann AJ, Carrière Y, Tabashnik BE (2009). Fitness costs of insect resistance to Bacillus thuringiensis. Annu Rev Entomol..

[86] Feyereisen R, Gilbert LI (2012). Insect CYP, genes and P450 enzymes. Insect molecular biology and biochemistry.

[87] Cheesman MJ, Traylor MJ, Hilton ME, Richards KE, Taylor MC, Daborn PJ (2013). Soluble and membrane-bound Drosophila melanogaster CYP6G1 expressed in Escherichia coli: purification, activity, and binding properties toward multiple pesticides. Insect Biochem Molec Biol..

[88] Teese MG, Farnsworth CA, Li Y, Coppin CW, Devonshire AL, Scott C (2013). Heterologous expression and biochemical characterisation of fourteen esterases from Helicoverpa armigera. PLoS ONE..

[89] Hartley CJ, Newcomb RD, Russell RJ, Yong CG, Stevens JR, Yeates DK (2006). Amplification of DNA from preserved specimens shows blowflies were preadapted for the rapid evolution of insecticide resistance. Proc Natl Acad Sci U S A..

[90] Mahon RJ, Olsen KM, Downes S (2008). Isolations of Cry2Ab resistance in Australian populations of Helicoverpa armigera (Lepidoptera: Noctuidae) are allelic. J Econ Entomol..

[91] Gnerre S, MacCallum I, Przybylski D, Ribeiro FJ, Burton JN, Walker BJ (2011). High-quality draft assemblies of mammalian genomes from massively parallel sequence data. Proc Natl Acad Sci U S A..

[92] Miller JR, Delcher AL, Koren S, Venter E, Walenz BP, Brownley A (2008). Aggressive assembly of pyrosequencing reads with mates. Bioinformatics..

[93] Trapnell C, Pachter L, Salzberg SL (2009). TopHat: discovering splice junctions with RNA-Seq. Bioinformatics..

[94] Trapnell C, Williams BA, Pertea G, Mortazavi A, Kwan G, van Baren MJ (2010). Transcript assembly and quantification by RNA-Seq reveals unannotated transcripts and isoform switching during cell differentiation. Nat Biotech..

[95] Grabherr MG, Haas BJ, Yassour M, Levin JZ, Thompson DA, Amit I (2011). Full-length transcriptome assembly from RNA-Seq data without a reference genome. Nat Biotech..

[96] Cantarel BL, Korf I, Robb SMC, Parra G, Ross E, Moore B (2008). MAKER: an easy-to-use annotation pipeline designed for emerging model organism genomes. Genome Res..

[97] Stanke M, Diekhans M, Baertsch R, Haussler D (2008). Using native and syntenically mapped cDNA alignments to improve de novo gene finding. Bioinformatics..

[98] Korf I (2004). Gene finding in novel genomes. BMC Bioinformatics..

[99] Lomsadze A, Ter-Hovhannisyan V, Chernoff YO, Borodovsky M (2005). Gene identification in novel eukaryotic genomes by self-training algorithm. Nucleic Acids Res..

[100] Li L, Stoeckert CJ, Roos DS (2003). OrthoMCL: identification of ortholog groups for eukaryotic genomes. Genome Res..

[101] Li W, Godzik A (2006). Cd-hit: a fast program for clustering and comparing large sets of protein or nucleotide sequences. Bioinformatics..

[102] Papanicolaou A, Schetelig MF, Arensburger P, Atkinson PW, Benoit JB, Bourtzis K (2016). The whole genome sequence of the Mediterranean fruit fly, Ceratitis capitata (Wiedemann), reveals insights into the biology and adaptive evolution of a highly invasive pest species. Genome Biol..

[103] Hatje K, Kollmar M (2014). Kassiopeia: a database and web application for the analysis of mutually exclusive exomes of eukaryotes. BMC Genomics..

[104] Haas BJ, Salzberg SL, Zhu W, Pertea M, Allen JE, Orvis J (2008). Automated eukaryotic gene structure annotation using EVidenceModeler and the program to assemble spliced alignments. Genome Biol..

[105] Haas BJ, Delcher AL, Mount SM, Wortman JR, Smith RK, Hannick LI (2003). Improving the Arabidopsis genome annotation using maximal transcript alignment assemblies. Nucleic Acids Res..

[106] Keller O, Odronitz F, Stanke M, Kollmar M, Waack S (2008). Scipio: using protein sequences to determine the precise exon/intron structures of genes and their orthologs in closely related species. BMC Bioinformatics..

[107] Jones P, Binns D, Chang H-Y, Fraser M, Li W, McAnulla C (2014). InterProScan 5: genome-scale protein function classification. Bioinformatics..

[108] Ashburner M, Ball CA, Blake JA, Botstein D, Butler H, Cherry JM (2000). Gene Ontology: tool for the unification of biology. Nat Genet..

[109] Punta M, Coggill PC, Eberhardt RY, Mistry J, Tate J, Boursnell C (2012). The Pfam protein families database. Nucleic Acids Res..

[110] Sigrist CJA, de Castro E, Cerutti L, Cuche BA, Hulo N, Bridge A (2013). New and continuing developments at PROSITE. Nucleic Acids Res..

[111] Letunic I, Doerks T, Bork P (2012). SMART 7: recent updates to the protein domain annotation resource. Nucleic Acids Res..

[112] Maere S, Heymans K, Kuiper M (2005). BiNGO: a Cytoscape plugin to assess overrepresentation of Gene Ontology categories in Biological Networks. Bioinformatics..

[113] Shannon P, Markiel A, Ozier O, Baliga NS, Wang JT, Ramage D (2003). Cytoscape: a software environment for integrated models of biomolecular interaction networks. Genome Res..

[114] Supek F, Bošnjak M, Škunca N, Šmuc T (2011). REVIGO summarizes and visualizes long lists of gene ontology terms. PLoS ONE..

[115] Smit AFA, Hubley R, Green P. RepeatMasker Open 4.0. http://www.repeatmasker.org.

[116] Friedlaender MR, Mackowiak SD, Li N, Chen W, Rajewsky N (2012). miRDeep2 accurately identifies known and hundreds of novel microRNA genes in seven animal clades. Nucleic Acids Res.

[117] Kozomara A, Griffiths-Jones S (2013). miRBase: integrating microRNA annotation and deep sequencing data. Nucleic Acids Res.

[118] Ali MI, Luttrell RG, Young SY (2006). Susceptibilities of Helicoverpa zea and Heliothis virescens (Lepidoptera: Noctuidae) populations to Cry1Ac insecticidal protein. J Econ Entomol..

[119] Luttrell RG, Wan L, Knighten K (1999). Variation in susceptibility of noctuid (Lepidoptera) larvae attacking cotton and soybean to purified endotoxin proteins and commercial formulations of Bacillus thuringiensis. J Econ Entomol..

[120] Ali MI, Luttrell RG (2011). Susceptibility of Helicoverpa zea and Heliothis virescens (Lepidoptera: Noctuidae) to Vip3A insecticidal protein expressed in VipCot™ cotton. J Invert Path..

[121] Katoh K, Standley DM (2013). MAFFT Multiple sequence alignment software Version 7: improvements in performance and usability. Mol Biol Evol..

[122] Nguyen L-T, Schmidt HA, von Haeseler A, Minh BQ (2015). IQ-TREE: a fast and effective stochastic algorithm for estimating maximum-likelihood phylogenies. Mol Biol Evol..

[123] Minh BQ, Nguyen MAT, von Haeseler A (2013). Ultrafast approximation for phylogenetic bootstrap. Mol Biol Evol..

[124] Kalyaanamoorthy S, Minh BQ, Wong TFK, von Haeseler A, Jermiin LS (2017). ModelFinder: fast model selection for accurate phylogenetic estimates. Nat Methods..

[125] Guindon S, Dufayard J-F, Lefort V, Anisimova M, Hordijk W, Gascuel O (2010). New algorithms and methods to estimate Maximum-Likelihood phylogenies: Assessing the performance of PhyML 3.0.. Syst Biol.

[126] Stamatakis A (2014). RAxML version 8: a tool for phylogenetic analysis and post-analysis of large phylogenies. Bioinformatics..

[127] Yu G, Smith DK, Zhu H, Guan Y, Lam TT-Y (2017). GGTREE: an R package for visualization and annotation of phylogenetic trees with their covariates and other associated data. Methods Ecol Evol..

[128] Keightley PD, Pinharanda A, Ness RW, Simpson F, Dasmahapatra KK, Mallet J (2015). Estimation of the spontaneous mutation rate in Heliconius melpomene. Mol Biol Evol..

[129] Rambaut A, Drummond AJ. TreeAnnotator. http://beast.bio.ed.ac.uk.

[130] Rambaut A. FigTree. http://tree.bio.ed.ac.uk/software/figtree.

[131] Tajima F (1993). Simple methods for testing the molecular evolutionary clock hypothesis. Genetics..

[132] Kumar S, Stecher G, Tamura K (2016). MEGA7: molecular evolutionary genetics analysis Version 7.0 for bigger datasets. Mol Biol Evol.

[133] Mahon RJ, Olsen KM, Garsia KA, Young SR (2007). Resistance to Bacillus thuringiensis toxin Cry2Ab in a strain of Helicoverpa armigera (Lepidoptera: Noctuidae) in Australia. J Econ Entomol..

[134] Teakle RE, Jensen JM. Heliothis punctiger. In: Singh R, Moore RF, editors. Handbook of insect rearing. Vol 2. Amsterdam: Elsevier, 1985. p. 312–22.

[135] Reigada C, Guimaraes KF, Parra JRP. Relative fitness of Helicoverpa armigera (Lepidoptera: Noctuidae) on seven host plants: a perspective for IPM in Brazil. J Insect Sci. 2016;16. pii: 3. doi: 10.1093/jisesa/iev158. 10.1093/jisesa/iev158 PMC4725259 26798139

[136] Bolger AM, Lohse M, Usadel B (2014). Trimmomatic: a flexible trimmer for Illumina sequence data. Bioinformatics..

[137] Liao Y, Smyth GK, Shi W (2013). The Subread aligner: fast, accurate and scalable read mapping by seed-and-vote. Nucleic Acids Res..

[138] Liao Y, Smyth GK, Shi W (2014). featureCounts: an efficient general purpose program for assigning sequence reads to genomic features. Bioinformatics.

[139] Robinson MD, Oshlack A (2010). A scaling normalization method for differential expression analysis of RNA-seq data. Genome Biol..

[140] Law CW, Chen Y, Shi W, Smyth GK (2014). voom: precision weights unlock linear model analysis tools for RNA-seq read counts. Genome Biol..

[141] Leek JT, Storey JD (2007). Capturing heterogeneity in gene expression studies by surrogate variable analysis. PLoS Genet..

[142] Leek JT, Johnson WE, Parker HS, Jaffe AE, Storey JD (2012). The sva package for removing batch effects and other unwanted variation in high-throughput experiments. Bioinformatics..

[143] Langfelder P, Horvath S (2008). WGCNA: an R package for weighted correlation network analysis. BMC Bioinformatics..

[144] Greenfield P, Duesing K, Papanicolaou A, Bauer DC (2014). Blue: correcting sequencing errors using consensus and context. Bioinformatics..

[145] Wu TD, Nacu S (2010). Fast and SNP-tolerant detection of complex variants and splicing in short reads. Bioinformatics..

[146] McKenna A, Hanna M, Banks E, Sivachenko A, Cibulskis K, Kernytsky A (2010). The Genome Analysis Toolkit: a MapReduce framework for analyzing next-generation DNA sequencing data. Genome Res..

[147] DePristo MA, Banks E, Poplin R, Garimella KV, Maguire JR, Hartl C (2011). A framework for variation discovery and genotyping using next-generation DNA sequencing data. Nat Genet..

[148] Van der Auwera GA, Carneiro MO, Hartl C, Poplin R, del Angel G, Levy-Moonshine A (2013). From FastQ data to high confidence variant calls: the Genome Analysis Toolkit best practices pipeline. Curr Protoc Bioinformatics..

[149] Bouckaert R, Heled J, Kühnert D, Vaughan T, Wu CH, Xie D (2014). BEAST 2: a software platform for Bayesian evolutionary analysis. PLoS Comp Biol..

[150] Rambaut A, Suchard MA, Xie D, Drummond AJ. Tracer. http://beast.bio.edu.ac.uk/Tracer.

[151] Drummond AJ, Rambaut A (2007). BEAST: Bayesian evolutionary analysis by sampling trees. BMC Evol Biol..

