# Erratum to: Genomic innovations, transcriptional plasticity and gene loss underlying the evolution and divergence of two highly polyphagous and invasive Helicoverpa pest species

**DOI:** 10.1186/s12915-017-0413-3

**Published:** 2017-08-15

**Authors:** S. L. Pearce, D. F. Clarke, P. D. East, S. Elfekih, K. H. J. Gordon, L. S. Jermiin, A. McGaughran, J. G. Oakeshott, A. Papanicolaou, O. P. Perera, R. V. Rane, S. Richards, W. T. Tay, T. K. Walsh, A. Anderson, C. J. Anderson, S. Asgari, P. G. Board, A. Bretschneider, P. M. Campbell, T. Chertemps, J. T. Christeller, C. W. Coppin, S. J. Downes, G. Duan, C. A. Farnsworth, R. T. Good, L. B. Han, Y. C. Han, K. Hatje, I. Horne, Y. P. Huang, D. S. T. Hughes, E. Jacquin-Joly, W. James, S. Jhangiani, M. Kollmar, S. S. Kuwar, S. Li, N-Y. Liu, M. T. Maibeche, J. R. Miller, N. Montagne, T. Perry, J. Qu, S. V. Song, G. G. Sutton, H. Vogel, B. P. Walenz, W. Xu, H-J. Zhang, Z. Zou, P. Batterham, O. R. Edwards, R. Feyereisen, R. A. Gibbs, D. G. Heckel, A. McGrath, C. Robin, S. E. Scherer, K. C. Worley, Y. D. Wu

**Affiliations:** 1 CSIRO Black Mountain, GPO Box 1700, Canberra, ACT 2600 Australia; 2 School of Biological Sciences, University of Melbourne, Parkville, Vic Australia; 3 Research School of Biology, Australian National University, Canberra, ACT Australia; 4 Hawksbury Institute for the Environment, Western Sydney University, Penrith, NSW Australia; 5 Southern Insect Management Research Unit, USDA-ARS, Stoneville, MS USA; 6 Human Genome Sequencing Center, Baylor College of Medicine, Houston, TX USA; 7 Biological and Environmental Sciences, University of Stirling, Stirling, UK; 8 School of Biological Sciences, University of Queensland, Brisbane St Lucia, QLD Australia; 9 John Curtin School of Medical Research, Australian National University, Canberra, ACT Australia; 10 Max Planck Institute of Chemical Ecology, Jena, Germany; 11 Sorbonnes Universités, UPMC Université Paris 06, Institute of Ecology and Environmental Sciences of Paris, Paris, France; 12 National Institute for Agricultural Research (INRA), Institute of Ecology and Environmental Sciences of Paris, Versailles, France; 13 Plant and Food Research, Mt Albert, Auckland New Zealand; 14 CSIRO, Narrabri, NSW Australia; 15 State Key Laboratory of Integrated Management of Pest Insects and Rodents, Institute of Zoology, Chinese Academy of Sciences, Beijing, 100101 China; 16 College of Plant Protection, Nanjing Agricultural University, Nanjing, Jiangsu China; 17 Max Planck Institute for Biophysical Chemistry, Gottingen, Germany; 18 Institute of Plant Physiology and Ecology, Shanghai Institutes of Biological Sciences, Chinese Academy of Sciences, Shanghai, China; 19 Key Laboratory of Forest Disaster Warning and Control of Yunnan Province, Southwest Forestry University, Kunming, 650224 China; 20 J. Craig Venter Institute, Rockville, MD USA; 21 School of Veterinary and Life Sciences, Murdoch University, Perth, WA Australia; 22 Chongqing Key Laboratory of Biochemistry and Molecular Pharmacology, Chongqing Medical University, Chongqing, 400016 China; 23 CSIRO, Floreat Park, WA Australia; 24 Department of Plant and Environmental Sciences, University of Copenhagen, Thorvaldsensvej, Denmark

## Erratum

Upon publication of the original article [1], it was noticed that Dr Papanicolaou’s surname was spelt incorrectly. The correct spelling is “Papanicolaou”, as shown in the author list of this erratum.
