# Supplementary material for: Genomic innovations, transcriptional plasticity and gene loss underlying the evolution and divergence of two highly polyphagous and invasive Helicoverpa pest species
Source: BMC Biol. 2017 Jul 31;15:63. doi: 10.1186/s12915-017-0402-6 (PMC5535293; doi:10.1186/s12915-017-0402-6)

**FIGURE S1. GO term analyses of gene gain/loss events in *H. armigera* vs *B. mori* and *H. zea*.** The left panel shows GO terms enriched in the *H. armigera* gene set vs *B. mori*, and the right panel shows those enriched in the *H. armigera* gene set vs *H. zea*. The major term enriched in *H. armigera* relative to *H. zea*, for proteins associated with G-protein coupled receptor signalling, refers to the GRs. GO numbers assigned to all *H. armigera* genes and to the NCBI Gnomon gene models for *B. mori* can be downloaded as Additional file 2.

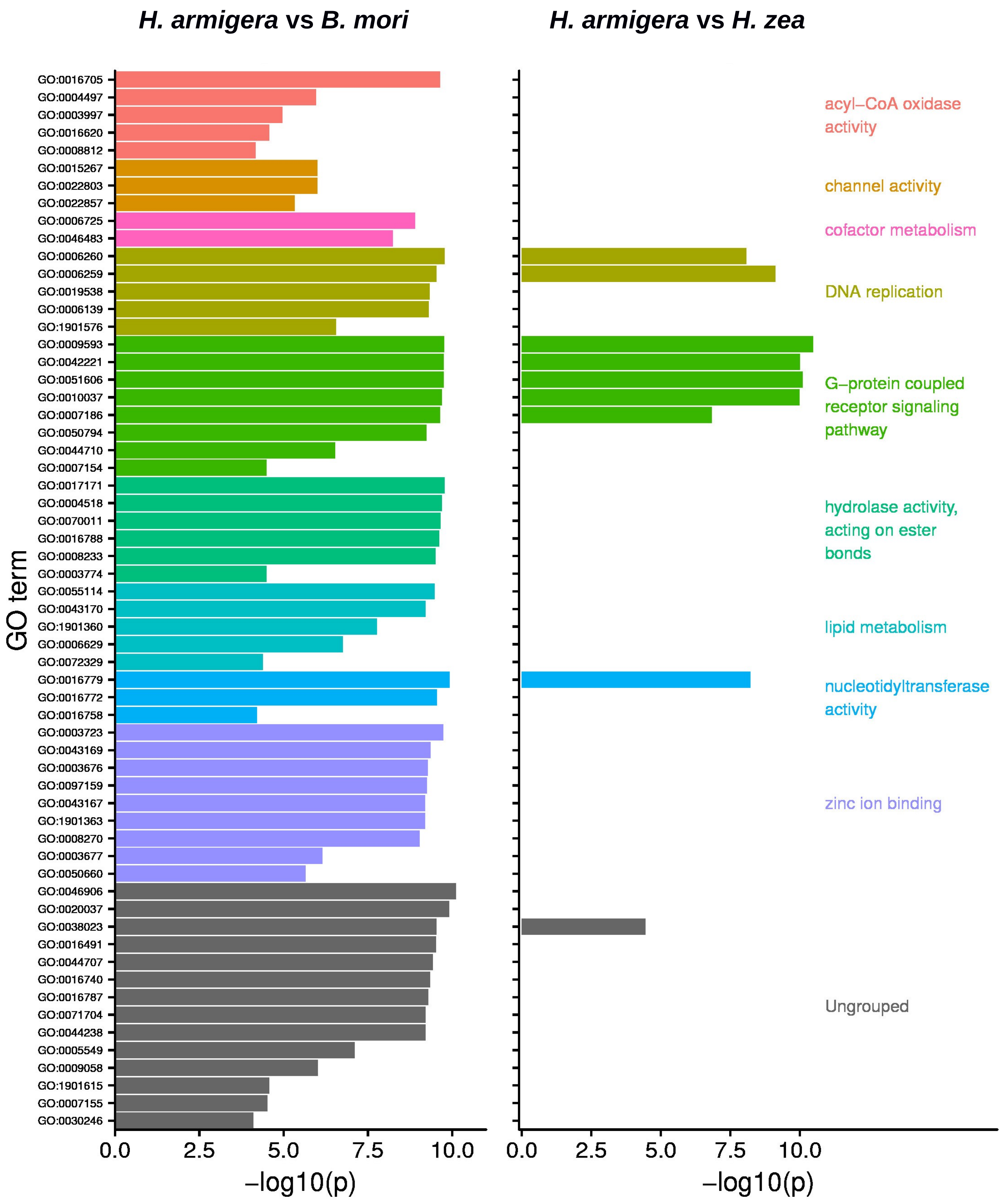

**FIGURE S2. Synteny between the *Helicoverpa* assemblies and with *B. mori*.** All scaffolds in the *Helicoverpa* assemblies were assessed using the ortholog matches given in Additional file 1: Table S1. A. Numbers of *H. armigera* scaffolds (vertical axis) matching sequences located on one, two or three *B. mori* chromosomes (shown on the horizontal axis). Only those scaffolds found to carry at least two contiguous *H. armigera* genes which had identifiable orthologs on the same *B. mori* chromosome were assessed. B. Numbers of *H. zea* scaffolds (vertical axis) matching sequences located on one to four *H. armigera* scaffolds (horizontal axis). This comparison used all direct orthologs between the two *Helicoverpa* species.

**A.**

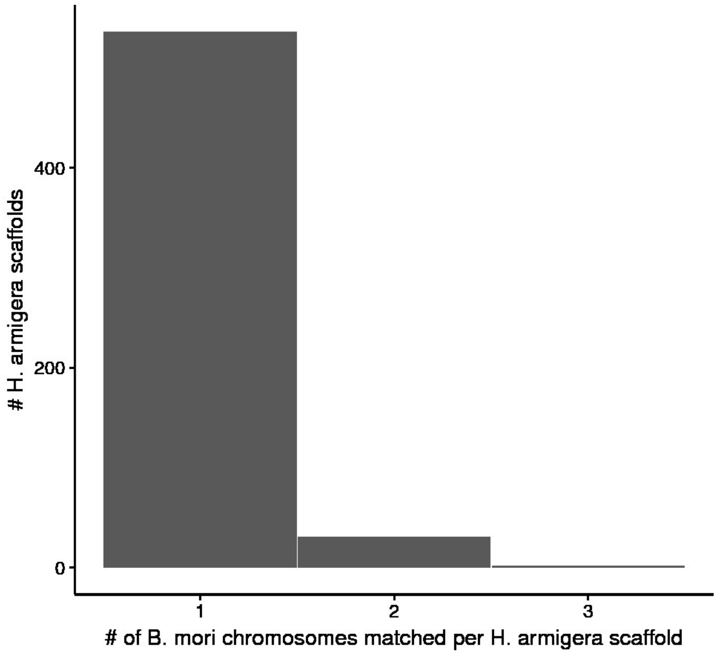


**B.**

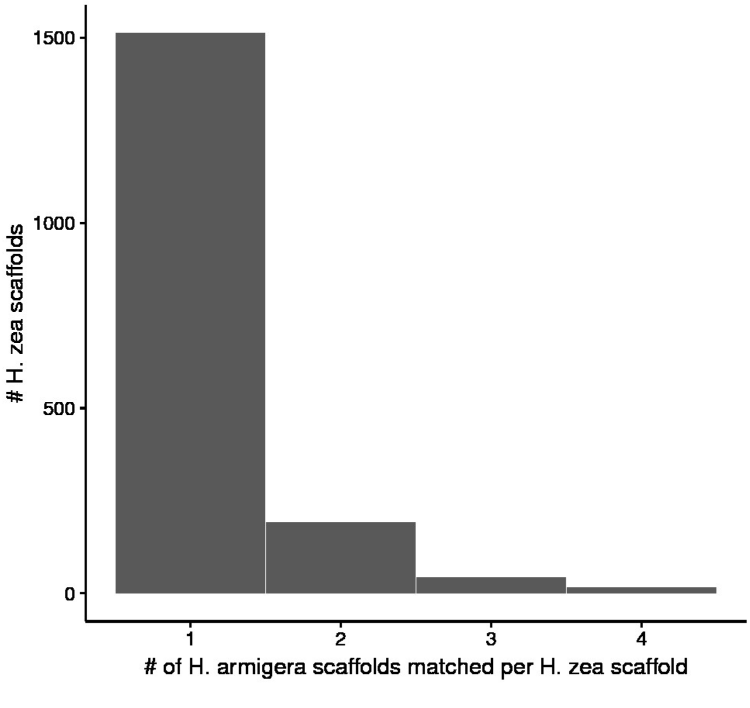

**Figure S3. Principal Component Analysis of the most variably expressed genes across the different diets.** The figure shows analysis of the 1882 DE genes identified in Additional file 8: Table S9. The first two components (A) separate the expression on cotton from other diets while the third and fourth components (B) resolve the remaining diets.

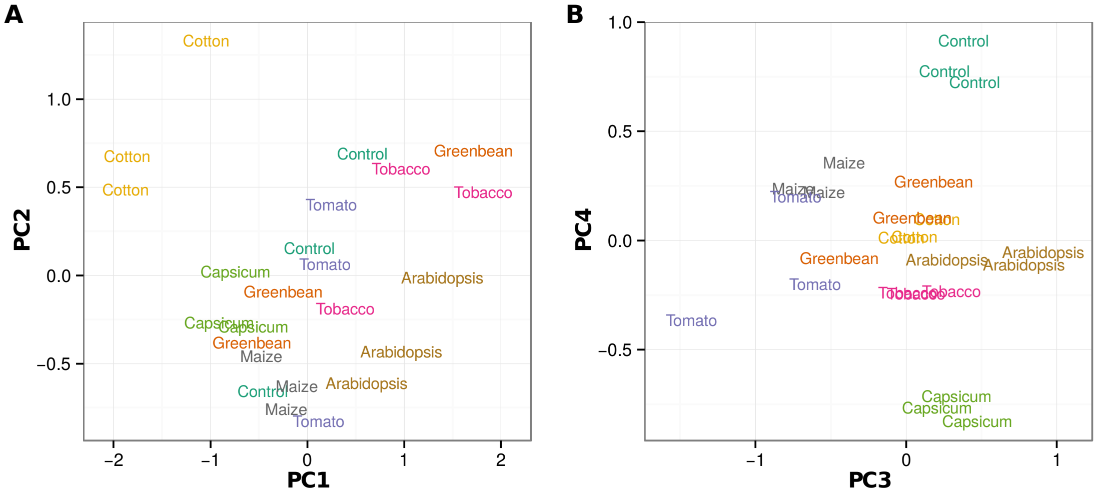

**FIGURE S4. GO terms enriched in the three key co-expression modules from the diet transcriptomics experiment.** The figure shows GO terms enriched in the modules D8, D25 and D10, with REViGO-derived functional classifications on the right.

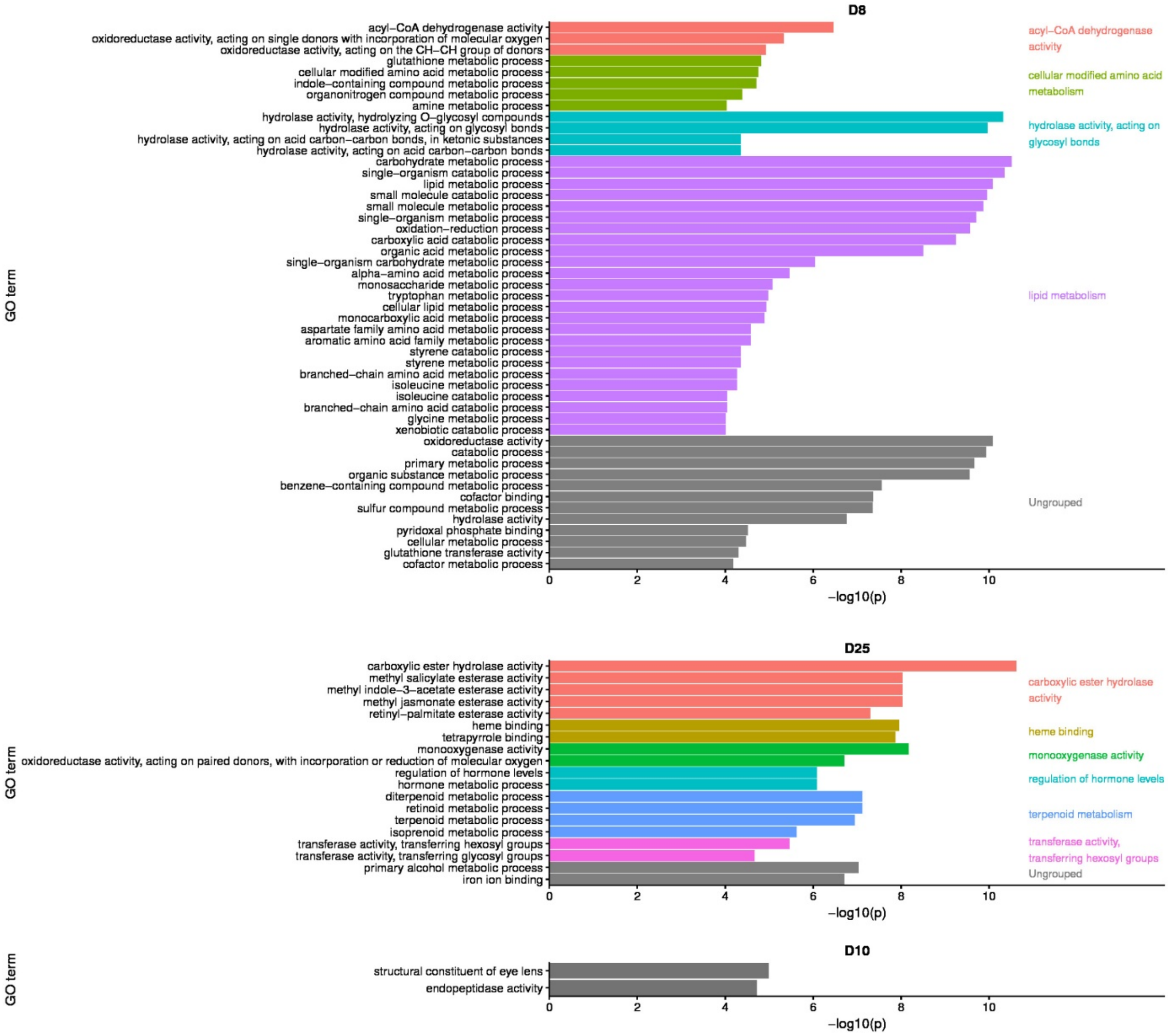

**FIGURE S5. Coalescence species tree and dating analysis.** Divergence analysis performed in BEAST for *H. armigera*, *H. zea*, and *H. punctigera*. Uncertainty in the 16 coalescence gene trees is reflected with shading. (*): Date of splits between *H. armigera* and *H. zea* = 1.4 +/- 0.1 Mya. (**): date of split of *H. punctigera* from *H. armigera* and *H. zea* =2.8 +/-0.2 Mya. Sequence alignments used for all 16 genes are available as Additional file 11. The following genes (six CYP450s, one GST, two serine proteases and three lipases) were used in divergence dating: HaOGno's 200003, 200028, 200067, 200080, 200096, 200098, 200232, 200445, 200489, 200565, 200610, 200612; the trees are available in Additional file 12.

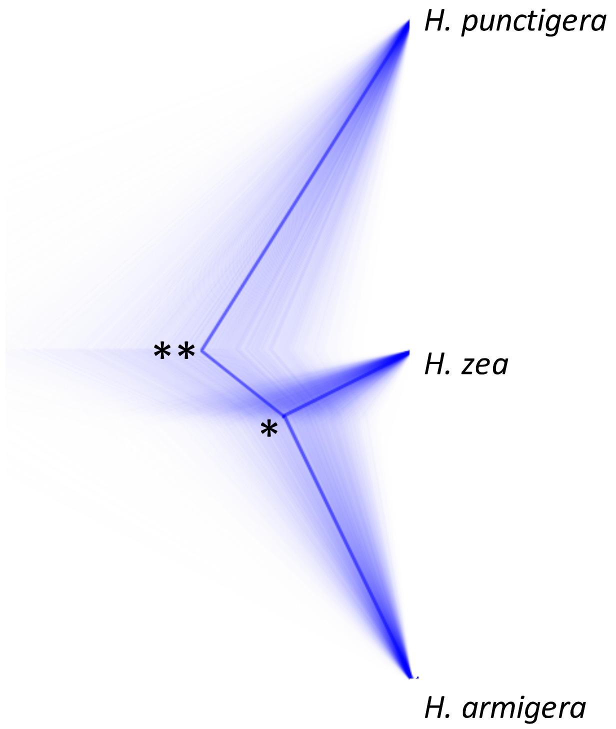

**FIGURE S6. Mean genome-wide nucleotide diversity estimates for the resequenced *H. armigera* and *H. zea* lines species using the *H. armigera* (A) and *H. zea* (B) reference sequences.** Only genomic windows with a minimum of at least 10 SNPs were used for these plots; bars within density plots represent quartile ranges of 0.25 and 0.75, with 0.50 represented as a black circle.

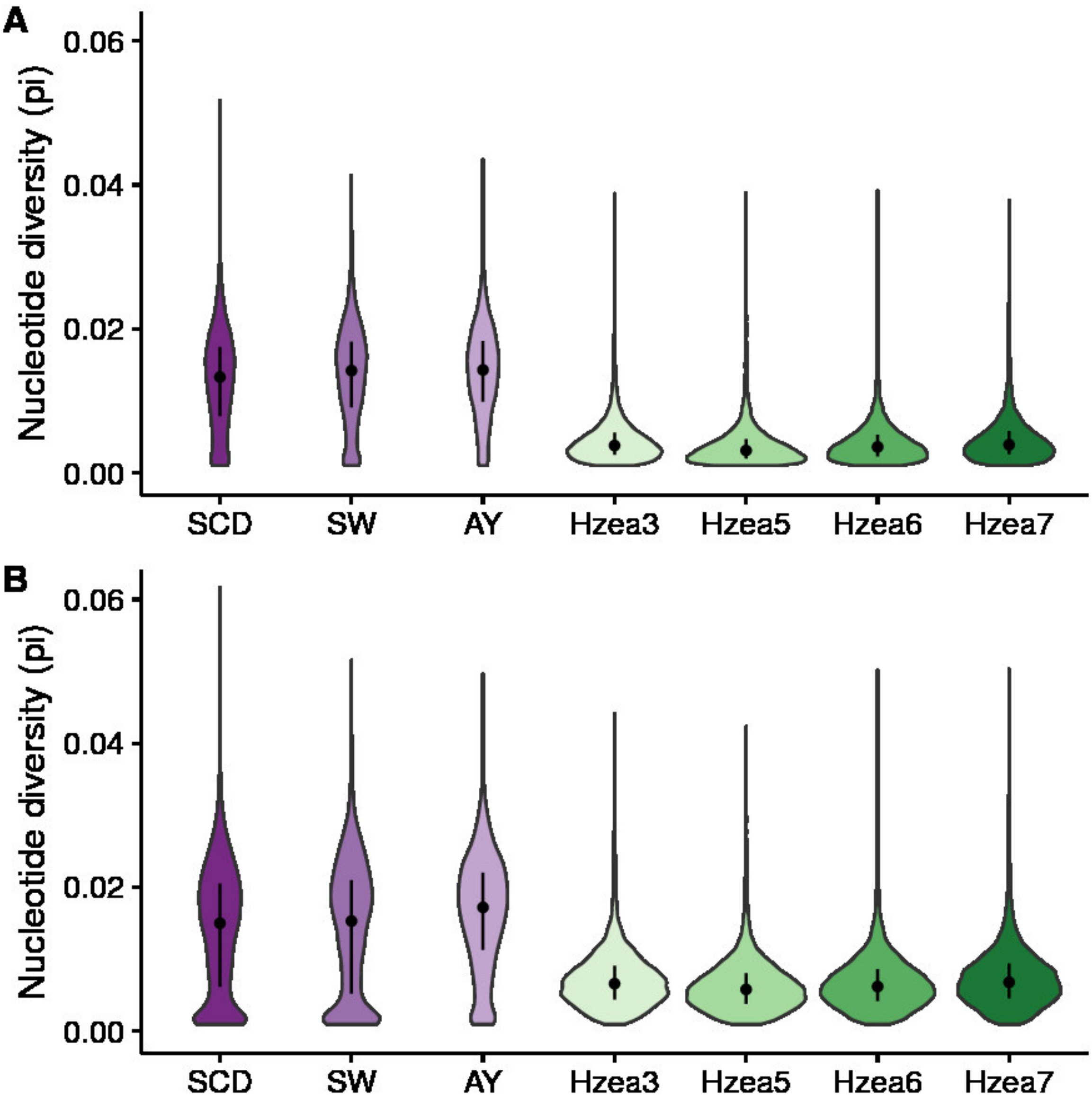

**FIGURE S7. Genome-wide synonymous and non-synonymous nucleotide diversity estimates and the correlations between them for the resequenced *H. armigera* and *H. zea* lines using the *H. armigera* reference sequence.** Range of genome-wide diversity measures for each strain using (A) synonymous variants only; (B) non-synonymous variants only – bars within density plots represent quartile ranges of 0.25 and 0.75, with 0.50 represented as a black circle. Patterns of correlated genome-wide diversity in 10 kb windows between *H. armigera* and *H. zea*, using SNP subsets: (C) synonymous variants only; (D) non-synonymous variants only. Only genomic windows with a minimum of at least 10 SNPs were used for all four plots.

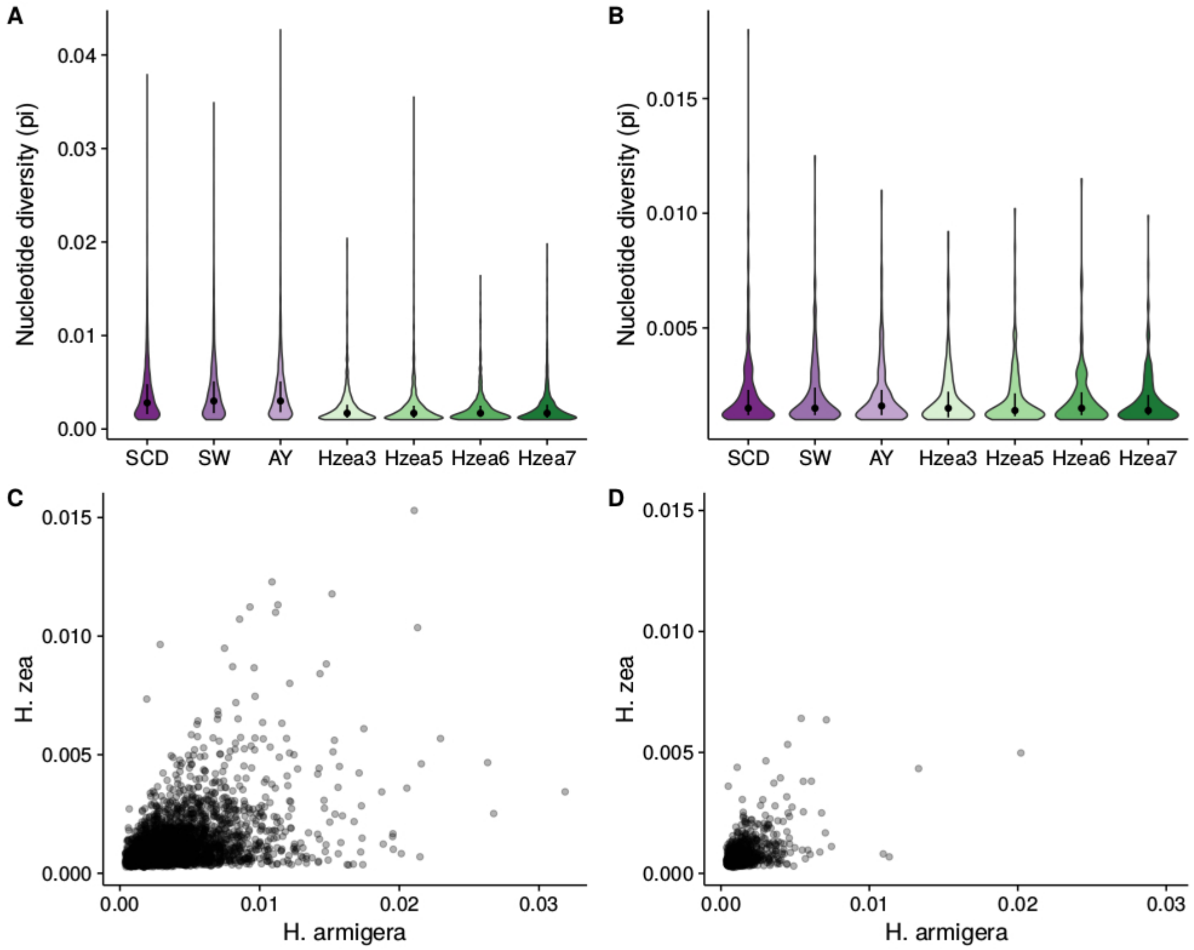

**FIGURE S8. Gene numbers in major detoxification and gustatory response families for nine lepidopterans.** The phylogeny of lepidopteran families is from Wahlberg et al. (2013) and of the Nymphalidae from Wahlberg et al. (2009). Data for P450s, CCEs and GSTs are from Rane et al. (2016) for seven of the species and this paper for the two *Helicoverpa* species. Data for GRs are as cited in Table 2 for *H. armigera*, *H. zea*, *B. mori* and *M. sexta*. For the other species, the data are from Briscoe et al. (2013) for *H. melpomene*, You et al. (2013) for *P. xylostella*, Zhan et al. (2011) and Engsontia et al. (2014) for *D. plexippus*, and our preliminary analyses from the OGS (which are therefore likely to be underestimates) for *M. cinxia* (Ahola et al. 2014) and *C. suppressalis* (Yin et al. 2014). The approximate divergence time of the heliothine mega-pest lineage (Cho et al. 2008) is indicated.

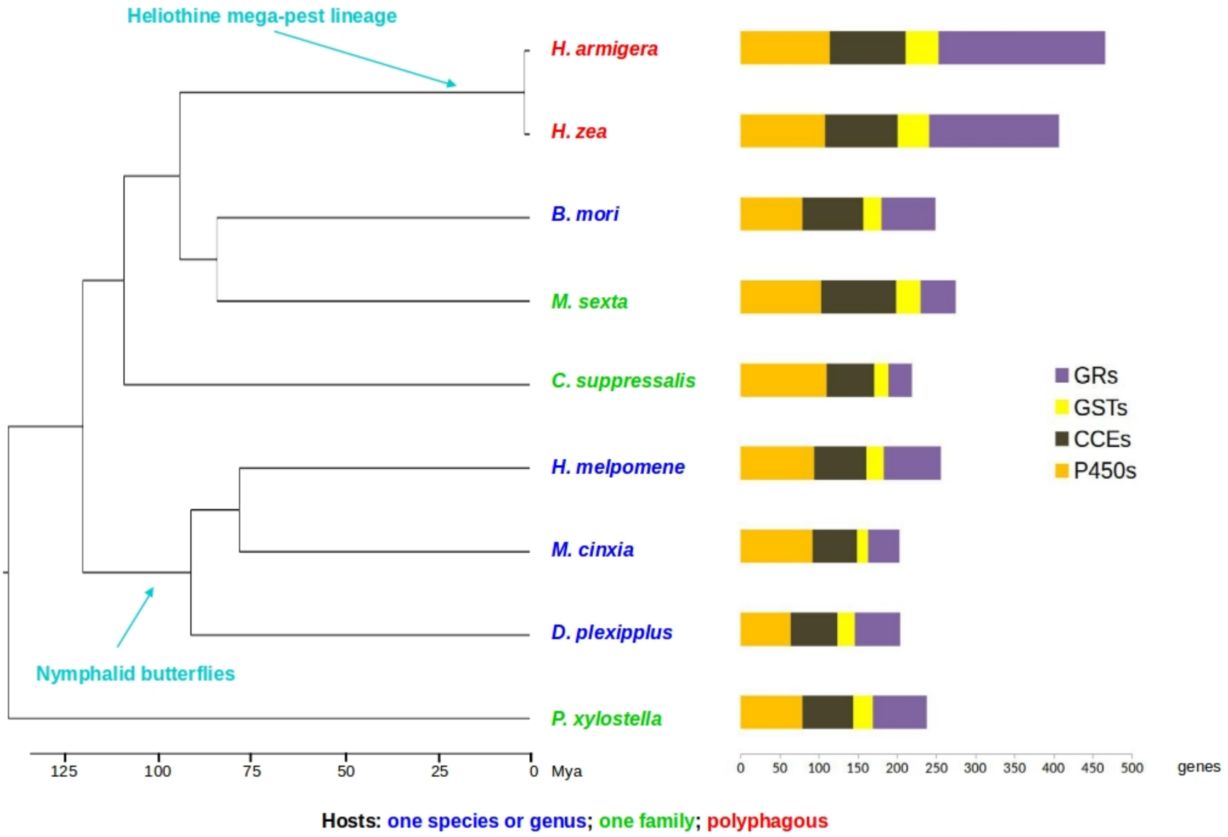

Ahola V, Lehtonen R, Somervuo P, Salmela L, Koskinen P, Rastas P,  Valimaki N, et al. (2014). The Glanville fritillary genome retains an ancient karyotype and reveals selective chromosomal fusions in Lepidoptera. Nat.Comm. 5:4737.

Briscoe AD, Macias-Muñoz A, Kozak KM, Walters JR, Yuan F, Jamie GA, Martin SH, et al. (2013). Female behaviour drives expression and evolution of gustatory receptors in butterflies. PLOS Genet. 9: e1003620. Cho S, Mitchell A, Mitter C, Regier J, Matthews M, Robertson R (2008). Molecular phylogenetics of heliothine moths (Lepidoptera: Noctuidae: Heliothinae), with comments on the evolution of host range and pest status. Syst. Ent. 33: 581-594.

Engsontia P, Sangket U, Chotigeat W, Satasook C (2014). Molecular evolution of the odorant and gustatory receptor genes in lepidopteran insects: implications for their adaptation and speciation. J Molec. Evol. 79: 21–39.

Rane RV, Walsh TK, Pearce SL, Jermiin LS, Gordon KHJ, Richards S, Oakeshott JG (2016). Are feeding preferences associated with the size of detoxifying enzyme families in insect herbivores? Curr. Opin.  Insect Sci., 13: 70-76.

Wahlberg N, Wheat CW, Peña C (2013). Timing and Patterns in the Taxonomic Diversification of Lepidoptera (butterflies and moths). PLOS ONE 8: e80875.

Wahlberg N, Leneveu J, Kodandaramaiah U, Pena C, Nylin S, Freitas AVL, Brower AVZ (2009). Nymphalid butterflies diversify following near demise at the Cretaceous/Tertiary boundary. Proc. Roy. Soc. (Lond.) B. Biol. Sci. 276: 4295–4302.

Yin,C., Liu,Y., Liu,J., Xia, H, Huang, S, Lin, Y, Han, Z, et al. (2014). ChiloDB: a genomic and transcriptome database for an important rice insect pest Chilo suppressalis. Database 2014: 1-7.

You M, Yue Z, He, W, Yang X, Yang G, Xie M, et al. (2013). A heterozygous moth genome provides insights into herbivory and detoxification. Nat. Genet. 45:220-225.

Zhan S, Merlin C, Boore JL, Reppert SM (2011). The monarch butterfly genome yields insights into long-distance migration. Cell 147, 1171–1185.

**Figure S26. Transcriptome profiles of the *H. armigera* GRs on different hosts.**

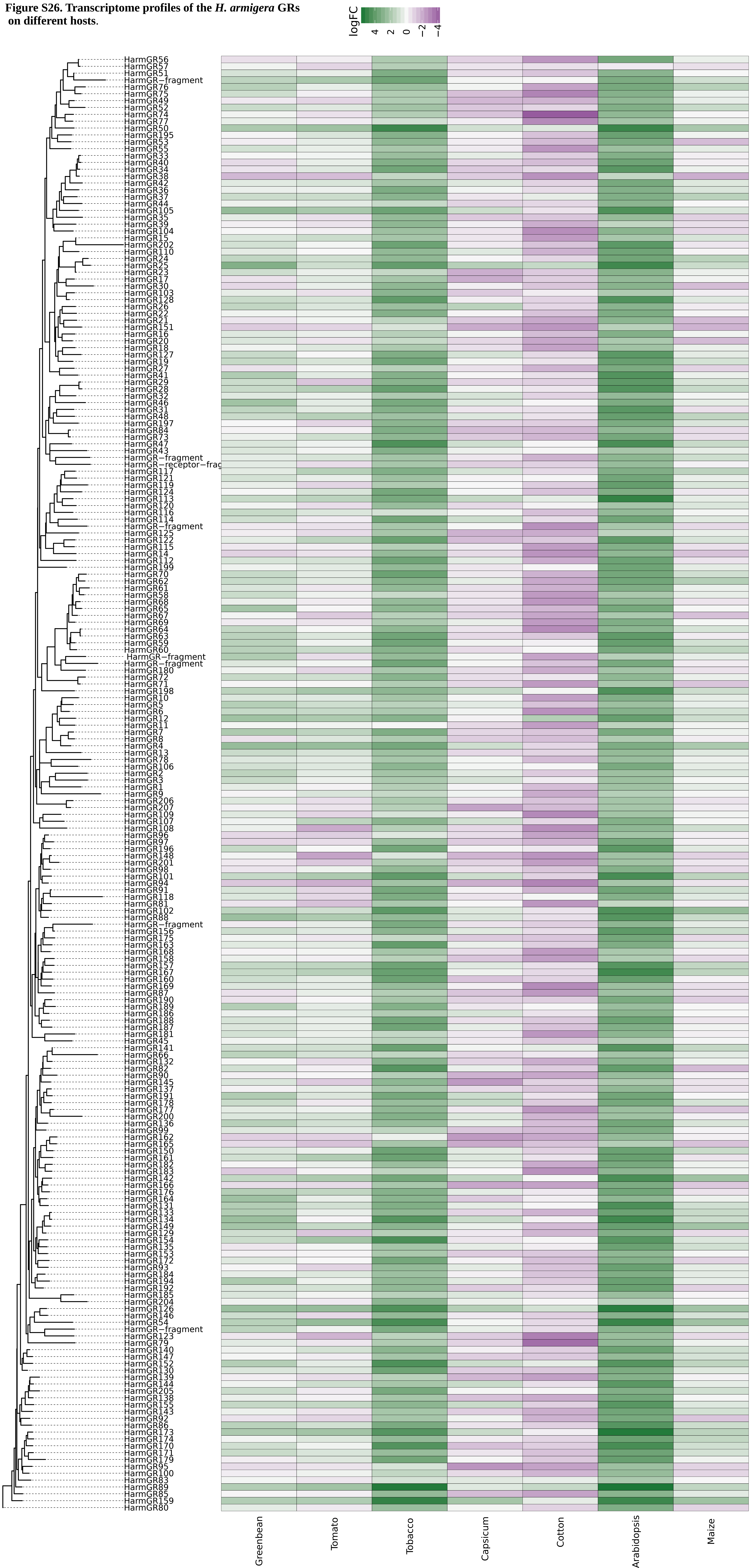

**Figure S29. Transcriptome profile of genes with GO Growth annotation.** The genes were ordered based on heirarchical clustering of their normalised tissue expression patterns. See Additional file 6: Table S5 for gene annotations.

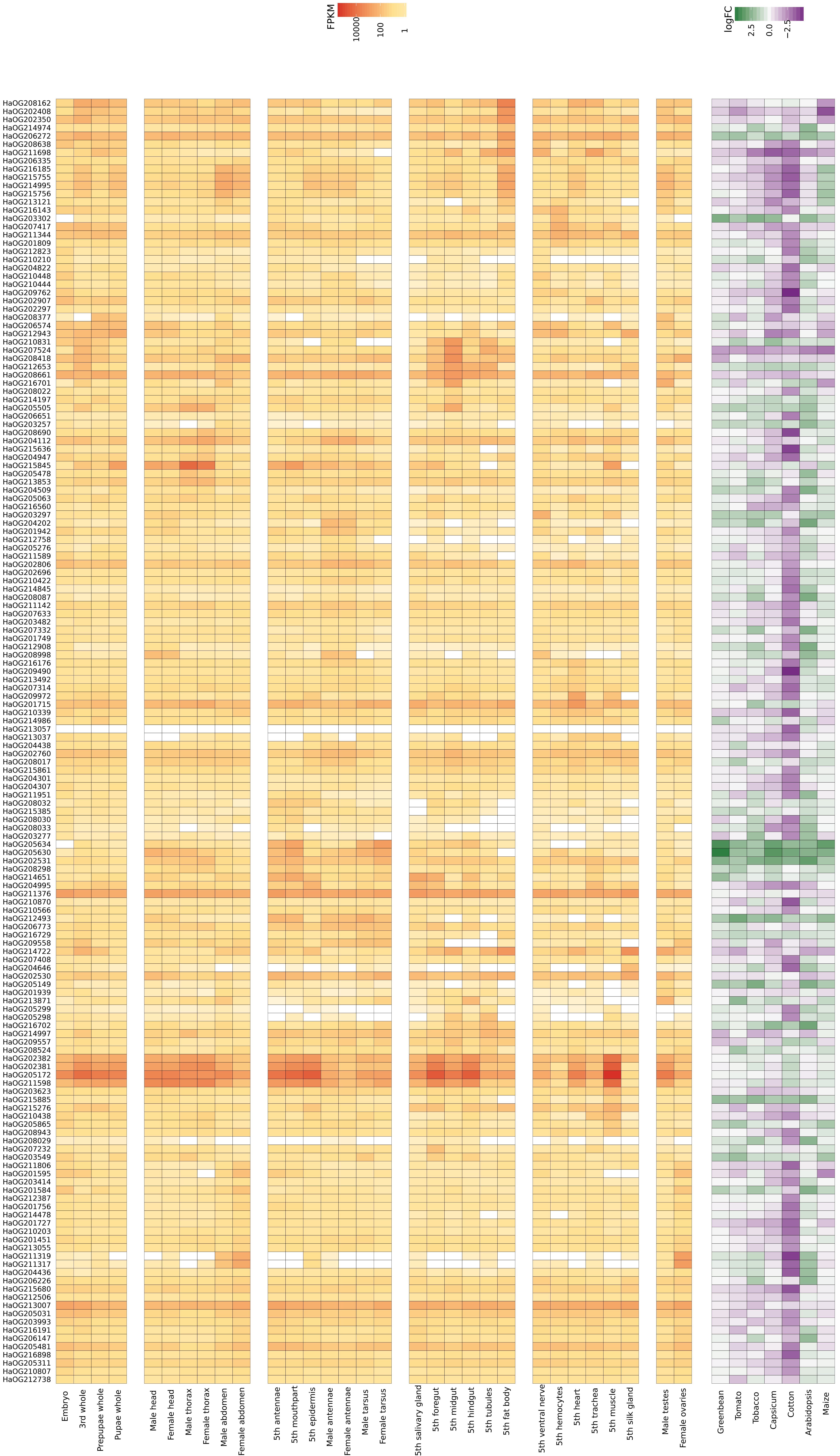

**Figure S30. Transcriptome profile of 240 transcription factors.** The genes were ordered based on heirarchical clustering of their normalised tissue expression patterns. See Additional file 6: Table S5 for gene annotations.

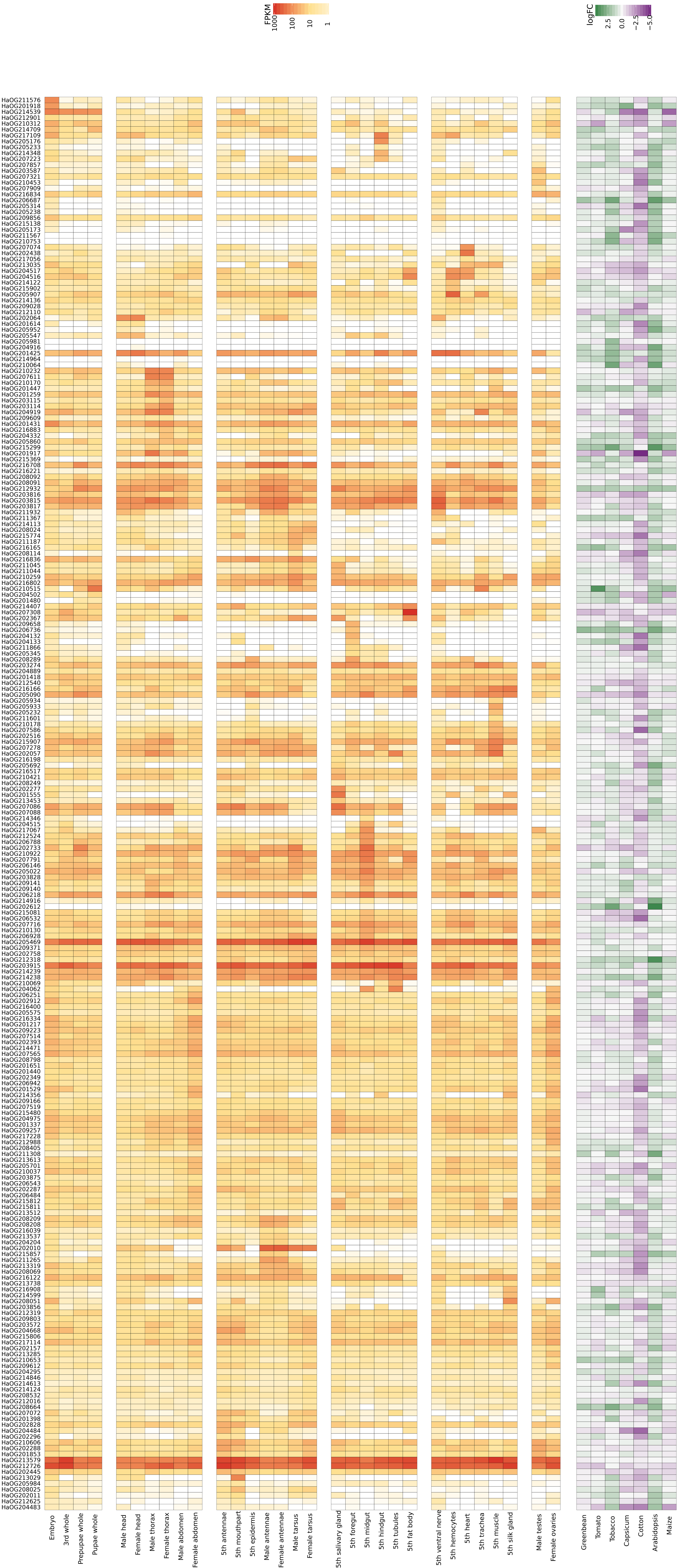

**Figure S31. Transcriptome profile of genes for cytoplasmic ribosomal proteins.**
See Additional file 6: Table S5 for gene annotations.

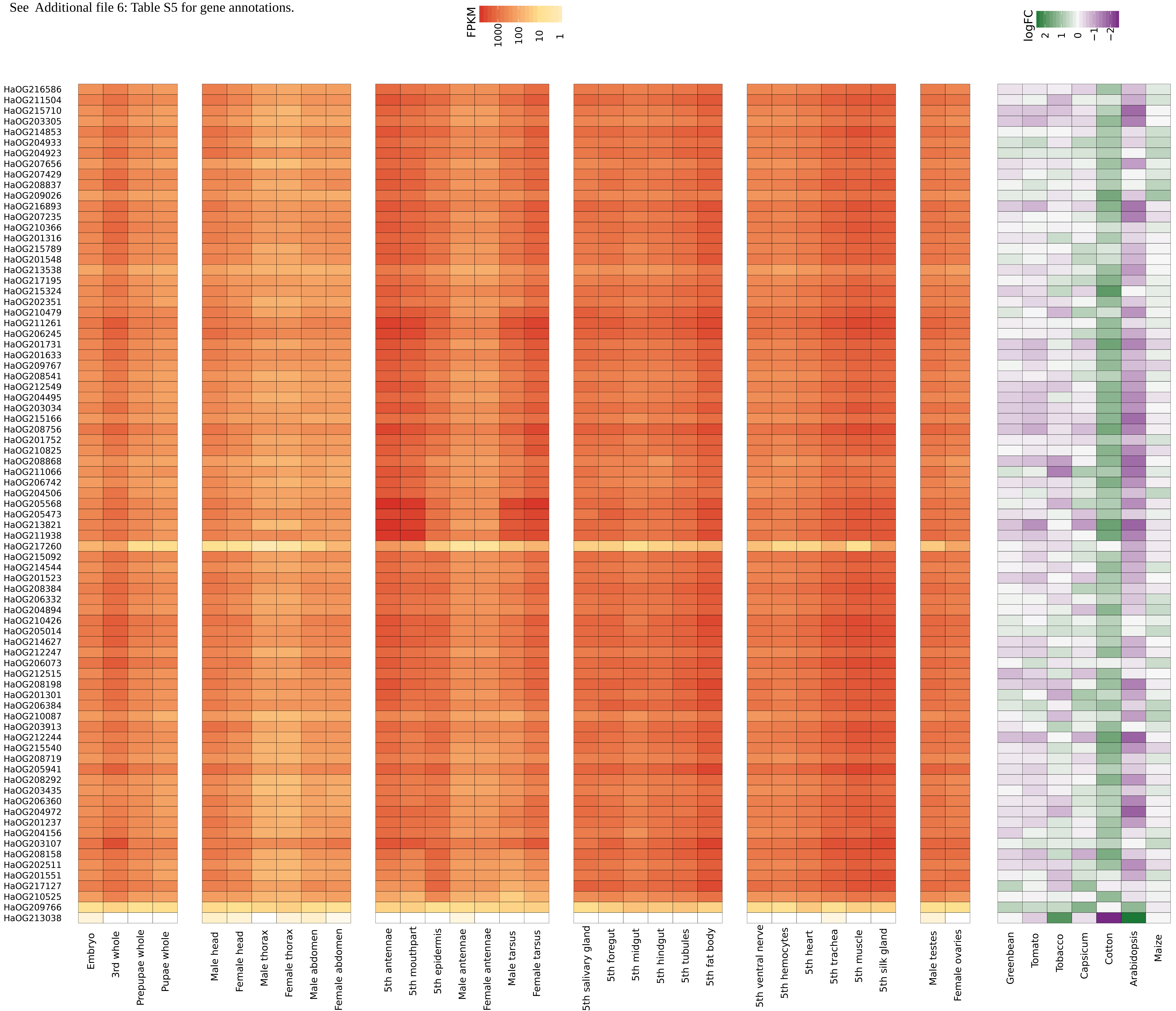

**Figure S32. Transcriptome profile of genes for cuticular proteins.**
See Additional file 6: Table S5 for gene annotations.

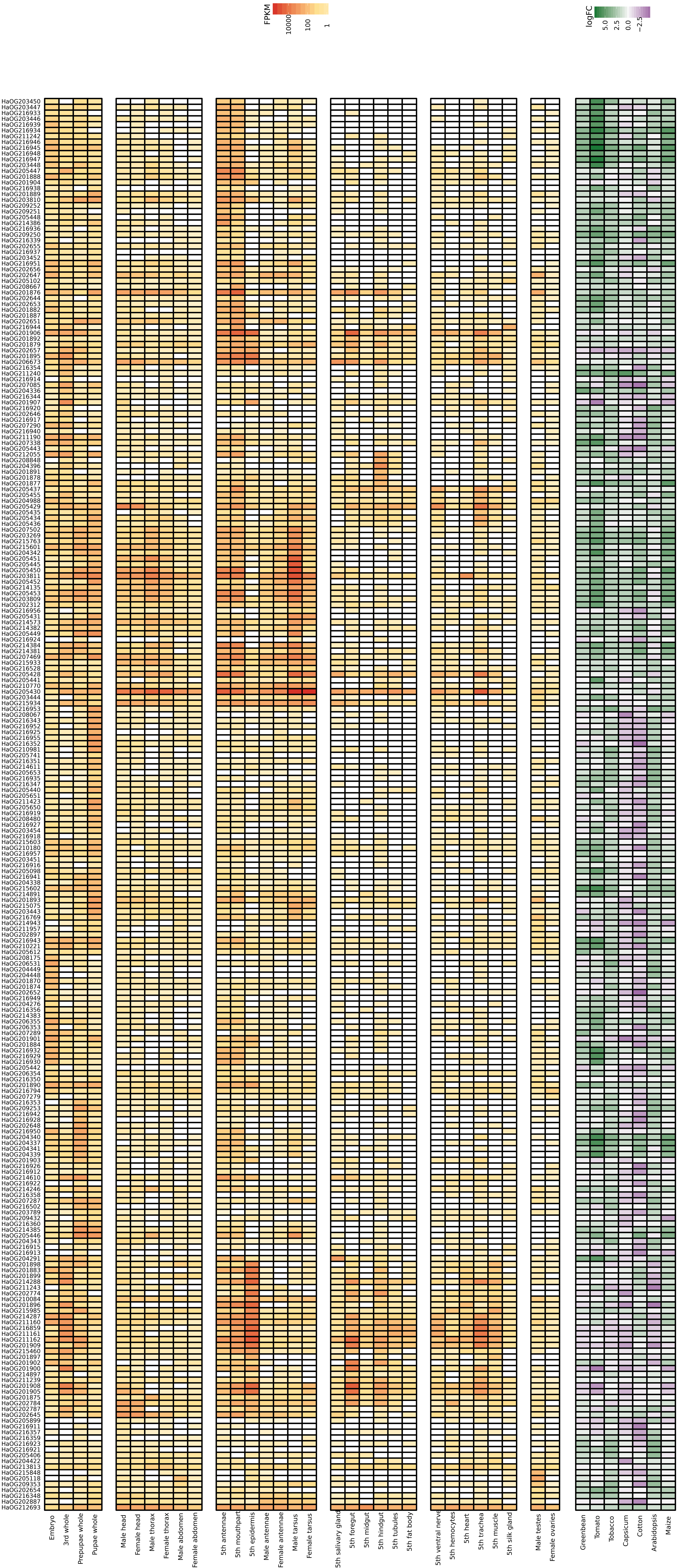

Supplement: Supplementary file 3 — GO term analyses of gene gain/loss events in H. armigera vs B. mori and H. zea Figure S2. Synteny between the Helicoverpa assemblies and with B. mori. Figure S3. Principal component analysis of the most variably expressed genes across the different diets. Figure S4. GO terms enriched in the three key co-expression modules from the diet transcriptomics experiment. Figure S5. Coalescence species tree and dating analysis. Figure S6. Genome-wide nucleotide diversity estimates for the resequenced H. armigera and H. zea lines species using the H. armigera (A) and H. zea (B) reference sequences. Figure S7. Genome-wide synonymous and non-synonymous nucleotide diversity estimates and the correlations between them for the resequenced H. armigera and H. zea lines using the H. armigera reference sequence. Figure S8. Gene numbers in major detoxification and gustatory response families for nine lepidopterans. Figure S26. Transcriptome profiles of the H. armigera GRs on different hosts. Figure S29. Transcriptome profile of genes with GO growth annotation. Figure S30. Transcriptome profile of 240 transcription factors. Figure S31. Transcriptome profile of genes for cytoplasmic ribosomal proteins. Figure S32. Transcriptome profile of genes for cuticular proteins. (PDF 2725 kb) [file 12915_2017_402_MOESM3_ESM.pdf]
