# Supplementary material for: Genomic innovations, transcriptional plasticity and gene loss underlying the evolution and divergence of two highly polyphagous and invasive Helicoverpa pest species
Source: BMC Biol. 2017 Jul 31;15:63. doi: 10.1186/s12915-017-0402-6 (PMC5535293; doi:10.1186/s12915-017-0402-6)
Supplement: Supplementary file 4 — Repeats recovered from the H. armigera and H. zea genomes. Table S6. Most recent expansions within major gene families of H. armigera analysed using *BEAST. Table S7. Details of Tajima’s relative rate tests on closely related H. armigera paralogues in the major detoxification and digestion gene families and GRs, together with the numbers of genes in the relevant clades missing in the H. zea assembly. Table S8. Details of the tissues and life stages sampled for the transcriptome atlas. Table S10a. Characteristics of the 37 diet transcriptome co-expression modules in term of enrichment for various groups of genes. Section 1: Detailed analysis of P450s in H armigera, H. zea, B. mori, M. sexta and P. xylostella. Section 2: Detailed analysis of CCEs in H armigera, H. zea, B. mori, M. sexta and P. xylostella. Section 3: Detailed analysis of GSTs in H armigera, H. zea, B. mori, M. sexta and P. xylostella. Section 4: Detailed analysis of UGTs in H armigera, H. zea, B. mori and M. sexta. Section 5: Detailed analysis of ABC transporters in H. armigera, H. zea, M. sexta and P. xylostella. Section 6: Detailed analysis of midgut serine proteases in H. armigera, H. zea and B. mori. Section 7: Detailed analysis of lipases in H armigera, H. zea and B. mori. Section 8: Detailed analysis of GR genes in H. armigera and H. zea. Section 9: Detailed analysis of stress response and immunity genes in H. armigera and H. zea. Section 10: Detailed analysis of some gene families related to larval growth. Section 11: Additional insights from diet transcriptome modules. Section 12. Detailed analysis of genes related to insecticide resistance in H. armigera and H. zea. Section 13. Detailed methods for the Helicoverpa genome assemblies and annotation. (DOCX 11091 kb) [file 12915_2017_402_MOESM4_ESM.docx]

**Supplementary Tables**

**Table S3. Major classes of transposable elements in the *H. armigera* and *H. zea* genomes.** The table shows the proportion each class contributes to the total for each genome.

| TE-type |  | *H. armigera* | *H. zea* | *H. zea* * |
| --- | --- | --- | --- | --- |
| Type I (reverse transcribed) |  |  |  |  |
| LTR |  | 0.12 | 0.08 | 0.14 |
| Non-LTR | LINE | 0.27 | 0.27 | 0.33 |
| Non-LTR | Uncharacterised | 0.14 | 0.17 | 0.06 |
|  | Total | 0.41 | 0.44 | 0.39 |
| Type 2 (DNA mediated) |  |  |  |  |
| DNA transposons |  | 0.45 | 0.48 | 0.42 |
| Uncharacterised |  | 0.02 | 0.000001 | 0.04 |
|  | Total | 0.47 | 0.48 | 0.46 |

* Figures from Coates et al. (2016).

Coates, B, Abel. CA, Perera, OP (2016). Estimation of long-terminal repeat element content in the *Helicoverpa zea* genome from high-throughput sequencing of bacterial artificial chromosome (BAC) pools. Genome,. doi: 10.1139/gen-2016-0067.

**TABLE S6. Most recent expansions within major gene families of *H. armigera* analysed using *Beast.** Minimum divergence times (million generations ago, mga, +- SE) for the most recent gene duplication within or between the species shown in each column. The averages of the three most recent duplications (where available, else the number of genes in the superscript note) in each clade are shown, with the most recent gene pair duplication within a species listed. All estimates were calculated from aligned nucleotide coding sequences using the *H. melpomene* mutation rate as described in the Materials and Methods. The gene alignments used (one file per clan) are available as Additional file 7[.](ftp://ftp.csiro.au/Helicoverpa/SI_6_Table_gene-alignments.zip)

| Gene family | Clan/sub-family | Clade | *H. armigera / H. zea* | *H. armigera* only | *H. armigera / B. mori* | *B. mori only* |
| --- | --- | --- | --- | --- | --- | --- |
| CCE | Clade 1 | clade 1 | 3.3 ± 0.4 | 13.6 ± 1.6 | 105 ± 11 | 108 ± 1 |
| CYP450 | Clan-3 | CYP6 | 5.6 ± 1.5 ^(2)^ | 13.4 ± 1.4 | 92.0 ± 11.3 | 55.1 ± 2.3 |
| CYP450 | Clan-4 | CYP340 | ND | 18.4 ± 2.4 | 170 ± 7 ^(2)^ | 8.2 ^(1)^ |
| CYP450 | Clan-4 | CYP4 | 6.5 ^(1)^ | 29.5 ± 5.2 ^(2)^ | 84.0 ± 1.7 ^(2)^ | NA |
| Serine protease | Trypsin | clade1 | 5.0 ^(1)^ | 15.9 ± 2.7 | 109 ± 14 | 27.8 ± 0.5 |
| Serine protease | Chymotrypsin | clade1 | 3.2 ^(1)^ | 6.8 ± 1.1 | 98.3 ± 7.5 | 51.2 ^(1)^ |

^(1)^ only one gene pair tested.

^(2)^ only two gene pairs tested.

ND: not done; NA: none available

**TABLE S7. Details of Tajima’s relative rate tests on closely related *H. armigera* paralogs in the major detoxification and digestion gene families and different pairs of GRs, together with the numbers of genes in the relevant clades missing in the *H. zea* assembly.** The P450, CCE and GST families are partitioned in these analyses into lineages for which there is empirical evidence for detoxification functions and those for which there is little or no such evidence.

| Family | Clan/group* | Clade | Total genes | Genes tested | Pairs with significant rate difference (P<0.05) | *H. armigera* gene names for pairs with significant rate differences | *H. armigera* genes not in *H. zea* |
| --- | --- | --- | --- | --- | --- | --- | --- |
| CYP450 | Detox-clan3 | 321, 324, 332, 337 | 11 | 4 | 0 |  |  |
|  | Detox-clan3 | CYP6 | 24 | 4 | 2 | CYP6AE14, 16 | CYP6AE14, 18, 59 |
|  | Detox-clan3 | CYP9 | 8 | 1 | 1 | CYP9A15 |  |
|  | Detox-clan4 | CYP340 | 25 | 5 | 3 | CYP340H5, K6, K12 | CYP340H3, H5 |
|  | Detox-clan4 | CYP341 | 7 | 2 | 1 | CYP341B8 |  |
|  | Detox-clan4 | CYP4 | 15 | 4 | 1 | CYP4AU2 |  |
|  | Other-clan2 | CYP18 | 2 | 1 | 1 | CYP18B1 |  |
|  | Other-clan2 | CYP305 | 1 | 1 | 0 |  |  |
|  | Other-clanM | CYP301 | 2 | 1 | 0 |  |  |
|  | Other-clanM | CYP428 | 1 | 1 | 1 | CYP428A1 |  |
| CCE | Detox | 1 | 23 | 7 | 4 | CCE001i, l, m, u | CCE001d, o, t |
|  | Detox | 3, 6, 8, 19 | 17 | 6 | 0 |  |  |
|  | Detox | 11 | 4 | 2 | 1 | CCE011b | CCE011d |
|  | Detox | 16 | 11 | 4 | 2 | CCE016g, c |  |
|  | Other - hormone-semiochemical processing | 20-22, 24, 30 | 16 | 5 | 0 |  |  |
| GST | Detox | Delta | 25 | 6 | 3 | GSTD1x, j, k | GSTD1t |
|  | Other | Omega | 3 | 1 | 0 |  |  |
|  | Detox | Sigma | 11 | 2 | 1 | GSTS1d | GSTS1e |
| UGT |  | 33 | 22 | 6 | 2 | UGT33M1, B7A | UGT33B7B, B9A, F1B |
|  |  | 40 | 8 | 3 | 1 | UGT40F1 | UGT40F1 |
| Serine protease | Trypsin | 1 | 29 | 5 | 2 | Tryp103, 121 | Tryp101, 116, 117, 121, 126 |
|  |  | 3 | 4 | 1 | 1 | Tryp001 |  |
|  |  | 5 | 6 | 2 | 0 |  |  |
|  |  | 8 | 7 | 1 | 1 | Tryp077 |  |
|  | Chymotrypsin | 1 | 26 | 6 | 5 | Chym101, 108, 116, 122, 124 | Chym115, 116, 124 |
|  |  | 4 | 5 | 1 | 1 | Chym009 | Chym011 |
|  |  | 7, 8, 11 | 7 | 3 | 0 |  |  |
| Lipase | Acid | 1, 3 | 18 | 6 | 0 |  |  |
|  |  | 2 | 10 | 3 | 2 | Lipase26,27 |  |
|  | Neutral | 1, 5, 8-12 | 38 | 11 | 0 |  |  |
|  |  | 2 | 9 | 3 | 1 | Lipase88 |  |
|  |  | 3 | 9 | 3 | 2 | Lipase07,92 |  |
|  |  | 4 | 2 | 1 | 0 |  | Lipase42 |
| GR |  |  | 213 | 35 | 7 | GR190, 150, 139, 176, 61, 54, 205 | 47; incl. GR139, 176 |

**TABLE S8. Details of the tissues and life stages sampled for the transcriptome atlas.**

| RNA Library # | Genbank Biosample # | Developmental stage | Sex | Tissue | Number of individuals |
| --- | --- | --- | --- | --- | --- |
| 1 | 6608634 | Embryo | Mixed | Embryo | 100s |
| 2 | 6608635 | 3rd instar | Mixed | Whole animal | 7 |
| 3 | 6608636 | post feeding larvae to prepupae | Mixed | Whole animal | 8 |
| 4 | 6608637 | Pupae | Mixed | Whole animal | 14 |
| 5 | 6608638 | adult | Male | Abdomen | 12 |
| 6 | 6608639 | adult | Female | Abdomen | 12 |
| 7 | 6608640 | adult | Female | Head | 18 |
| 8 | 6608641 | adult | Male | Head | 18 |
| 9 | 6608642 | feeding 5th instar | Mixed | Foregut | >10 |
| 10 | 6608643 | feeding 5th instar | Mixed | Midgut 2 | >10 |
| 11 | 6608644 | feeding 5th instar | Mixed | Hindgut | >10 |
| 12 | 6608645 | feeding 5th instar | Mixed | Malph. Tubules | >10 |
| 13 | 6608646 | feeding 5th instar | Mixed | silk gland 2 | >10 |
| 14 | 6608647 | feeding 5th instar | Mixed | thoracic fat body | >10 |
| 15 | 6608648 | feeding 5th instar | Mixed | fat body | >10 |
| 16 | 6608649 | feeding 5th instar | Mixed | ventral nerve | >10 |
| 17 | 6608650 | feeding 5th instar | Mixed | Hemocytes | 12 |
| 18 | 6608651 | feeding 5th instar | Mixed | salivary gland 1 | >10 |
| 19 | 6608652 | feeding 5th instar | Mixed | Heart | >10 |
| 20 | 6608653 | feeding 5th instar | Mixed | Trachea | >10 |
| 21 | 6608654 | feeding 5th instar | Mixed | muscle 2 | >10 |
| 22 | 6608655 | Adult | Female | ovaries - female tract | 8 |
| 23 | 6608656 | adult | Male | testes - male tract | 8 |
| 24 | 6608657 | feeding 5th instar | Mixed | epidermis/cuticle | >10 |
| 25 | 6608658 | adult | Male | Antennae |  |
| 26 | 6608659 | adult | Female | Antennae |  |
| 30 | 6608660 | adult | Male | Thorax |  |
| 31 | 6608661 | adult | Female | Thorax |  |
| 32 | 6608662 | feeding 5th instar | Mixed | Antennae |  |
| 33 | 6608663 | feeding 5th instar | Mixed | Mouthpart |  |
| 34 | 6608664 | adult | Male | Tarsus |  |
| 35 | 6608665 | adult | Female | Tarsus |  |

**TABLE S9. Complete list of 11,213 *H. armigera* genes for which transcriptome data were analysed.** This table identifies the 1,882 genes found to be differentially expressed on one or more hosts, and whether they were up- or down-regulated (denoted by 1 or -1 respectively) on each host. Also listed are which of the 37 diet transcriptome co-expression modules or the 5 tissue/developmental stage modules each gene has been assigned to, if any. The table is available as Additional file 8.

**Table S10a. Characteristics of the 37 diet transcriptome co-expression modules.** This table summarizes the proportion of genes in each module derived from all annotated families, from the 546 genes in the manually annotated detoxification/digestion gene families, or that are among the 1882 DE genes. It also summarizes enrichment for genes in the five developmental transcriptome modules implicated in host use. The complete table, Table S10b, including notes on gene function, and detailed breakdowns of gene families represented within modules, is available as Additional file 9.

| Diet-module | Number of genes | % of module genes in annotated families | % of module genes in detox/ digestive families | % of all module genes in annotated families that are detox genes | % of module genes that are DE | % of module genes in tissue/ developmental stage modules T1-5 | Transcription factors |
| --- | --- | --- | --- | --- | --- | --- | --- |
| D1 | 51 | 7.8% | 2.0% | 25.0% | 2.0% | 9.8% | 1 |
| D2 | 97 | 24.7% | 6.2% | 25.0% | 3.1% | 9.3% |  |
| D3 | 53 | 43.4% | 1.9% | 4.3% | 37.7% | 17.0% |  |
| D4 | 316 | 18.7% | 0.0% | 0.0% | 17.7% | 4.7% | 5 |
| D5 | 182 | 7.7% | 0.5% | 7.1% | 12.6% | 3.8% | 1 |
| D6 | 286 | 9.8% | 1.0% | 10.7% | 7.0% | 10.8% | 8 |
| D7 | 343 | 9.6% | 3.2% | 33.3% | 26.2% | 6.4% | 4 |
| D8 | 284 | 24.3% | 14.4% | 59.4% | 39.1% | 47.5% | 3 |
| D9 | 855 | 11.0% | 2.6% | 23.4% | 7.3% | 6.0% | 7 |
| D10 | 369 | 19.2% | 9.8% | 50.7% | 53.7% | 33.6% | 3 |
| D11 | 231 | 15.6% | 3.9% | 25.0% | 5.2% | 7.4% | 1 |
| D12 | 574 | 28.0% | 4.4% | 15.5% | 26.8% | 10.6% | 4 |
| D13 | 470 | 12.6% | 1.9% | 15.3% | 23.2% | 10.0% | 11 |
| D14 | 439 | 7.5% | 1.1% | 15.2% | 8.4% | 9.1% | 9 |
| D15 | 58 | 15.5% | 1.7% | 11.1% | 1.7% | 1.7% | 4 |
| D16 | 114 | 10.5% | 1.8% | 16.7% | 22.8% | 8.8% | 3 |
| D17 | 58 | 5.2% | 0.0% | 0.0% | 1.7% | 0.0% | 1 |
| D18 | 168 | 13.7% | 0.6% | 4.3% | 26.2% | 17.9% | 1 |
| D19 | 297 | 16.2% | 4.7% | 29.2% | 33.0% | 14.8% | 4 |
| D20 | 34 | 26.5% | 11.8% | 44.4% | 79.4% | 32.4% |  |
| D21 | 140 | 20.0% | 11.4% | 57.1% | 48.6% | 21.4% | 3 |
| D22 | 74 | 8.1% | 0.0% | 0.0% | 24.3% | 13.5% | 2 |
| D23 | 153 | 39.2% | 3.3% | 8.3% | 37.3% | 23.5% | 1 |
| D24 | 41 | 9.8% | 7.3% | 75.0% | 51.2% | 36.6% |  |
| D25 | 111 | 41.4% | 30.6% | 73.9% | 64.0% | 30.6% | 1 |
| D26 | 64 | 14.1% | 3.1% | 22.2% | 29.7% | 1.6% | 3 |
| D27 | 42 | 19.0% | 2.4% | 12.5% | 19.0% | 7.1% | 4 |
| D28 | 44 | 11.4% | 11.4% | 100.0% | 56.8% | 20.5% | 2 |
| D29 | 77 | 28.6% | 2.6% | 9.1% | 33.8% | 19.5% | 2 |
| D30 | 58 | 6.9% | 1.7% | 25.0% | 8.6% | 10.3% |  |
| D31 | 84 | 11.9% | 6.0% | 50.0% | 20.2% | 11.9% |  |
| D32 | 70 | 30.0% | 5.7% | 19.0% | 28.6% | 48.6% | 1 |
| D33 | 61 | 14.8% | 1.6% | 11.1% | 21.3% | 8.2% | 2 |
| D34 | 24 | 4.2% | 0.0% | 0.0% | 0.0% | 4.2% | 1 |
| D35 | 29 | 17.2% | 6.9% | 40.0% | 13.8% | 20.7% | 1 |
| D36 | 25 | 12.0% | 4.0% | 33.3% | 24.0% | 4.0% |  |
| D37 | 32 | 84.4% | 84.4% | 100.0% | 18.8% | 59.4% |  |

**Supplementary Sections**

**Section 1. Detailed analysis of P450s in *H armigera*, *H. zea*, *B. mori*, *M. sexta* and *P. xylostella***

**Section 2. Detailed analysis of CCEs in *H armigera*, *H. zea*, *B. mori*, *M. sexta* and *P. xylostella***

**Section 3. Detailed analysis of GSTs in *H armigera*, *H. zea*, *B. mori,* *M. sexta* and *P. xylostella***

**Section 4. Detailed analysis of UGTs in *H armigera*, *H. zea*, *B. mori* and *M. sexta***

**Section 5. Detailed analysis of ABC transporters in *H. armigera*, *H. zea*, *B. mori*, *M. sexta* and *P. xylostella***

**Section 6. Detailed analysis of midgut serine proteases in *H. armigera*, *H. zea* and *B. mori***

**Section 7. Detailed analysis of lipases in *H armigera*, *H. zea* and *B. mori***

**Section 8. Detailed analysis of GR genes in *H. armigera* and *H. zea***

**Section 9. Detailed analysis of stress response and immunity genes in *H. armigera* and *H. zea***

**Section 10. Detailed analysis of some gene families related to larval growth**

**Section 11. Additional insights from diet transcriptome modules**

**Section 12. Detailed analysis of genes related to insecticide resistance in *H. armigera* and *H. zea***

**Section 13. Detailed methods for the *Helicoverpa* genome assemblies and annotation**

**Section 1. Detailed analysis of P450s in *H armigera*, *H. zea*, *B. mori,* *M. sexta* and *P. xylostella***

The P450 genes of Lepidoptera can be grouped into four ‘clans’, the CYP2, CYP3, CYP4 and mitochondrial (M) clans, as has been commonly found elsewhere in the class Insecta. Several families can be found in each clan (Table S11), with most genes belonging to the CYP3 and CYP4 clans. These are also the clans most often associated with detoxification functions and insecticide resistance. Typically for the P450s, we find extremes in the distributions of P450s across the five Lepidoptera analysed here, ranging from arthropod-wide orthologs to lineage-specific sets of paralogs. Lepidoptera-wide orthologs predominate in the smaller clans 2 and M but are uncommon in clans 3 and 4 and, overall, account for only about a quarter of the *Helicoverpa* P450s (Fig. S9).

We find the highly conserved and arthropod-wide clans 2 and M genes necessary for moulting hormone biosynthesis (called Halloween genes in Drosophila, CYP302A1, CYP306A1, CYP307A2, CYP314A1, CYP315A1) and inactivation (CYP18). There appears to be a lepidopteran-specific duplication of the latter. Two tandemly arrayed CYP18 genes (CYP18A1 and CYP18B1) are clustered head to head with CYP306A1. In *B. mori*, the duplication was followed by subfunctionalisation, with the two paralogs having different tissue-specific expression profiles (Li et al. 2014).

We also find a number of conserved insect-specific genes. These include the clan 2 CYP15C1 gene which encodes the stereospecific epoxidase of the farnesoate (and homofarnesoate) precursors of the juvenile hormones in the corpora allata (Daimon et al. 2012). CYP15C1 is the ortholog of the CYP15A1 (Helvig et al. 2004) found in other insects (except higher Diptera). They also include the lepidopteran paralogs of CYP4G1, encoding the long chain fatty aldehyde decarbonylase involved in cuticular hydrocarbon biosynthesis (Qiu et al. 2012). Although most insect genomes carry two of these paralogs, the lepidopteran genomes shown in the phylogeny in Fig. S9 have several additional CYP4G1 duplications which appear to be relatively recent, with four and five paralogs in the Bombycoidae and Noctuidae. High expression of CYP4G25 in *Antheraea yamamai* has been linked to the entry into diapause of pharate first instar larvae (Yang et al. 2008).

All the lepidopteran genomes analysed also include copies of the clan M CYP301A1 genes that are widely distributed and highly conserved in insects. Null mutants of this gene in Drosophila have a distinct cuticular phenotype (Sztal et al. 2012). Similarly, we find the insect-wide orthologs of CYP303A1, encoded by the Drosophila nompH gene of unknown function (Willingham and Keil 2004), as well as the CYP301B1 and CYP49A1 genes, but the biochemical function of the enzymes they encode is unknown.

Our analysis also reveals a small set of CYP families and subfamilies which are common to the lepidopteran species studied but not found (to date) in any other insect orders. This ‘lepidopteran-specific P450 set’ includes several series of 1:1 orthologs in the CYP3 (CYP6AW1, CYP9AJ1, CYP365A1, CYP338A1), CYP4 (CYP366A1 and CYP367A1) and mitochondrial (CYP339A1 and CYP428A1) clans. Together with their high degree of sequence conservation, this suggests that these subfamilies encode P450s involved in lepidopteran-specific aspects of physiology. The presence of small, conserved, subfamilies within clans (CYP3,CYP4) otherwise characterized by multiple duplications supports the view that during evolution of the P450 superfamily, physiological functions and detoxification functions can switch back and forth along some branches of the tree (Sezutsu et al. 2013).

One of the lepidopteran-specific P450s above, CYP428A1, has some peculiar sequence features. It encodes a highly divergent member of the mitochondrial P450 clan. The conserved I-helix Thr in other P450s is replaced by Pro and the region around the invariant Cys shows a gap of three residues, yielding PFG...ExCPA/G (CYP428A1) as compared to the PFGxGxxxCxG/A consensus for other P450s. However, it retains the conserved C-helix WxxxR motif and the K-helix ExxR motif. Nothing is yet known of the function of CYP428A1 but the missing Thr in the I-helix and the gap in the Cys loop suggest that it is not a typical monooxygenase.

In the larger families of P450 genes in clans 3 and 4, we find particularly pronounced gene clusters formed by recent duplications (‘blooms’) in the two *Helicoverpa* species for some P450s, eg CYP340s (60 - 83 % and 50 – 89 % identity across the CYP340H and CYP340K clusters respectively) and CYP6AEs (49 – 93 % identity across the cluster) in particular.

The total number of P450 genes in the various lepidopteran genomes seems to relate to feeding habits (*B. mori* is monophagous on mulberry, *M. sexta* and *P. xylostella* oligophagous on Solanaceae and Brassicaceae respectively and the two *Helicoverpa* highly polyphagous). Nonetheless, when the distribution of genes within families and subfamilies is analyzed, it becomes apparent that there is a set of P450 families and sub-families with relatively small numbers of genes which is common to the three species. This set has approximately the same number of genes in the three species. Within this ‘lepidopteran P450 set’ are several sets of 1:1 orthologs that may be involved in lepidopteran-specific aspects of physiology. In the families with larger numbers of genes, we find blooms, recent series of duplications, often located on chromosomal gene clusters, in *M. sexta* (e.g. CYP333) and the two *Helicoverpa* (e.g. CYP340), but not in *B. mori*. It is generally thought that the diversity generated by the blooms allows these P450s to respond to environmental challenges. Notably in this respect, one *H. armigera* P450, CYP6AE14, which empirical evidence implicates in the detoxification of plant defense compounds, is missing in the genome of *H. zea*, and *H. zea* appears to have slightly narrower host range than *H. armigera* (see main text).

The transcriptome data in Fig. S10 show show relatively high expression across diverse tissues and stages for many P450s in the CYP4 clan, including in a variety of external body parts (epidermis, antennae, mouthparts tarsus) relating directly to environmental interactions and internal organs (gut, fat body, salivary glands) related to digestion, as might be expected for enzymes with detoxification functions. Interestingly the P450s in the other major clan associated with detoxification show lower levels of expression overall, mainly focussed in the external body parts and with relatively little expression in digestive tissues. Many enzymes in both clans however show differential expression on different diets; both clans show upregulation in Arabidopsis, with several CYP4s downregulated on maize and several CYP3s downregulated on cotton.

**Table S11: Distribution of the P450s from the five Lepidoptera across the major clans and families within them.** All genes were identified as described in Materials and Methods in the main text. Data for *B. mori*, *M. sexta* and *P. xylostella* are taken from Kanost et al. (2016). Files showing full alignments of the five species genomes are available from LSJ, KHJG or RF on request.

| P450 clan | P450 families | *H. armigera* | *H. zea* | *B. mori* | *M. sexta* | *P. xylostella* | Notes |
| --- | --- | --- | --- | --- | --- | --- | --- |
| 2 | CYP15 | 1 | 1 | 1 | 1 | 1 | A |
| 2 | CYP18 | 2 | 2 | 2 | 2 | 2 | B |
| M | CYP301 | 2 | 2 | 2 | 2 | 2 | M |
| M | CYP302 | 1 | 1 | 1 | 1 | 1 | C |
| 2 | CYP303 | 1 | 1 | 1 | 1 | 1 | H |
| 2 | CYP304 | 1 | 1 |  | 1 | 1 |  |
| 2 | CYP305 | 1 | 1 | 1 | 1 | 1 |  |
| 2 | CYP306 | 1 | 1 | 1 | 1 | 1 | D |
| 2 | CYP307 | 1 | 1 | 1 | 1 | 1 | E |
| M | CYP314 | 1 | 1 | 1 | 1 | 1 | F |
| M | CYP315 | 1 | 1 | 1 | 1 | 1 | G |
| 3 | CYP321 | 3 | 3 | 0 | 0 | 2 |  |
| 3 | CYP324 | 3 | 3 | 1 | 3 |  |  |
| 3 | CYP332 | 3 | 2 | 1 | 3 |  |  |
| M | CYP333 | 2 | 2 | 3 | 8 | 3 | N |
| 3 | CYP337 | 2 | 2 | 2 | 3 | 1 |  |
| 3 | CYP338 | 1 | 1 | 1 | 1 | 1 | Q |
| M | CYP339 | 1 | 1 | 1 | 1 | 1 | R |
| 4 | CYP340 | 25 | 23 | 10 | 5 | 11 | O |
| 4 | CYP341 | 7 | 7 | 5 | 7 | 2 |  |
| 3 | CYP354 | 1 | 1 | 1 | 1 | 1 | S |
| 3 | CYP365 | 1 | 1 | 1 | 1 | 1 | T |
| 4 | CYP366 | 1 | 1 | 1 |  | 1 |  |
| 4 | CYP367 | 2 | 2 | 2 | 2 | 2 |  |
| M | CYP428 | 1 | 1 | 1 | 1 | 1 | L |
| M | CYP49 | 1 | 1 | 1 | 1 | 1 | P |
| 4 | CYP4 | 15 | 15 | 12 | 16 | 9 | K |
| 3 | CYP6 | 24 | 21 | 17 | 20 | 22 | U |
| 3 | CYP9 | 8 | 8 | 7 | 10 | 6 | V |
|  | Total | 114 | 108 | 79 | 96 | 79 |  |

Notes to Table:

A: CYP15C1 is the epoxidase involved in JH biosynthesis in the corpora allata (Daimon et al. 2012). It is the ortholog of CYP15A1 (Helvig et al. 2004) found in other insects (except higher Diptera). In Lepidoptera CP15C1 is a farnesoid acid epoxidase, whereas in other insects CYP15A1 is a methyl farnesoate epoxidase.

B: Ecdysteroid 26 hydroxylase/oxidase. The 18A/B duplication appears to be lepidopteran specific, with each paralog having a tissue-specific expression (Li et al. 2014).

C: 22-hydroxylase of the ecdysone biosynthetic pathway. ortholog of the Halloween gene dib

D: 25-hydroxylase of the ecdysone biosynthetic pathway. ortholog of the Halloween gene phm

E: Catalyzes unknown early step of the ecdysone biosynthetic pathway. paralog of the Halloween gene spo

F: 20-hydroxylase of the ecdysone biosynthetic pathway. ortholog of the Halloween gene shd

G: 2-hydroxylase of the ecdysone biosynthetic pathway. ortholog of the Halloween gene sad

The known endocrine functions of P450s are therefore fully represented in Lepidoptera, with a unique CYP18 duplication.

H: Insect-wide ortholog of the Drosophila gene nompH of unknown function (Willingham and Keil 2004)

K: paralogs of the oxidative decarbonylase gene involved in the last step of cuticular hydrocarbon biosynthesis (Qiu et al. 2012). These are insect-specific genes, not found in Crustacea or further. Most insects have two CYP4G paralogs (except the honey bee and the pea aphid that have only one). The higher number found in Lepidoptera is a special case. High expression of CYP4G25 in *Antheraea yamamai* has been linked to the entry into diapause of pharate first instar larvae (Yang et al. 2008).

L: CYP428A1 is a lepidopteran-specific gene. It encodes a highly divergent member of the mitochondrial P450 clan of unknown function. The conserved I-helix Thr is replaced by Pro and the region around the invariant Cys shows a gap of three residues:

PFG...ExCPA/G (CYP428A1)

PFGxGxxxCxG/A (consensus)

However, the CYP428A1 sequences have the conserved C-helix WxxxR and the K-helix ExxR. The missing Thr in the I-helix and the gap in the Cys loop suggests that CYP428A1 is not a typical monooxygenase and that it may not even have a bound heme cofactor.

M: CYP301: insect-wide paralogs of unknown function

N: CYP333 is a lepidopteran version of the various mitochondrial ‘detoxification’ P450s that are typical of arthropods and distinguish them from vertebrates, where all mitochondrial clan P450s have endogenous, physiological functions (reviewed in Feyereisen 2011). *M. sexta* has apparently a small bloom in the CYP333 family.

O: CYP340 has bloomed in *H. armigera*/*H. zea*.

P: CYP49: insect-wide paralogs (close to CYP301). unknown function.

Q: CYP338A1 : Lepidopteran ortholog ?

R: CYP339A1 : Mitochondrial clan lepidopteran ortholog ?

S: CYP354 : Lepidopteran ortholog ?

T: CYP365 : Lepidopteran ortholog ?

U: CYP6AW : Lepidopteran ortholog ?

V: CYP9AJ : Lepidopteran ortholog ?

Re Notes Q – V: Even in the widely distributed CYP6 and CYP9 families that comprise many P450s clearly involved in detoxification, there are some subfamilies of small gene numbers with apparent orthologs among Lepidoptera (CYP6AW and CYP9AJ). This suggests that these subfamilies encode P450s with conserved (physiological ?) functions. It supports the view that during evolution of the P450 superfamily, physiological functions and detoxification functions can switch back and forth along some branches of the tree (Sezutsu et al. 2013). CYP304 is not universally distributed in insects (no clear pattern) and missing in *B. mori*. Its role is uncertain.

**Figure S9. The P450 phylogeny across the five lepidopterans.** Clan identifiers are given above each phylogram. Phylogenetic methods are described in Materials and Methods in the main text.

**M**

**
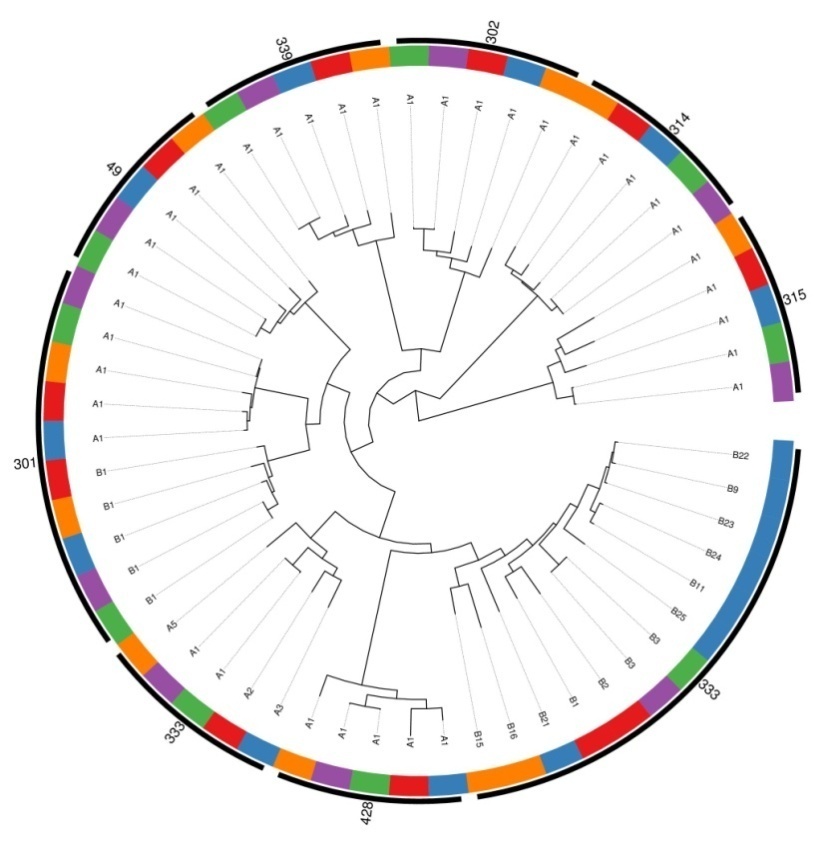
**

**2**

**
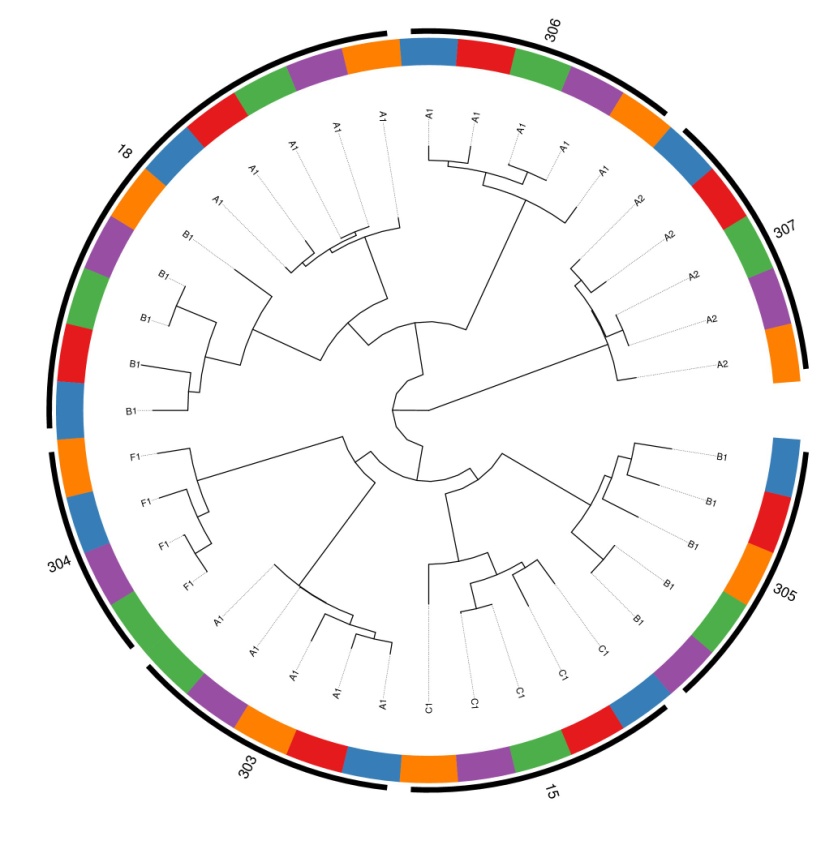
**

**
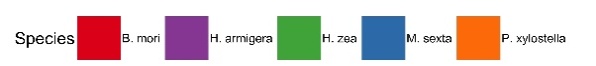
**

**3**


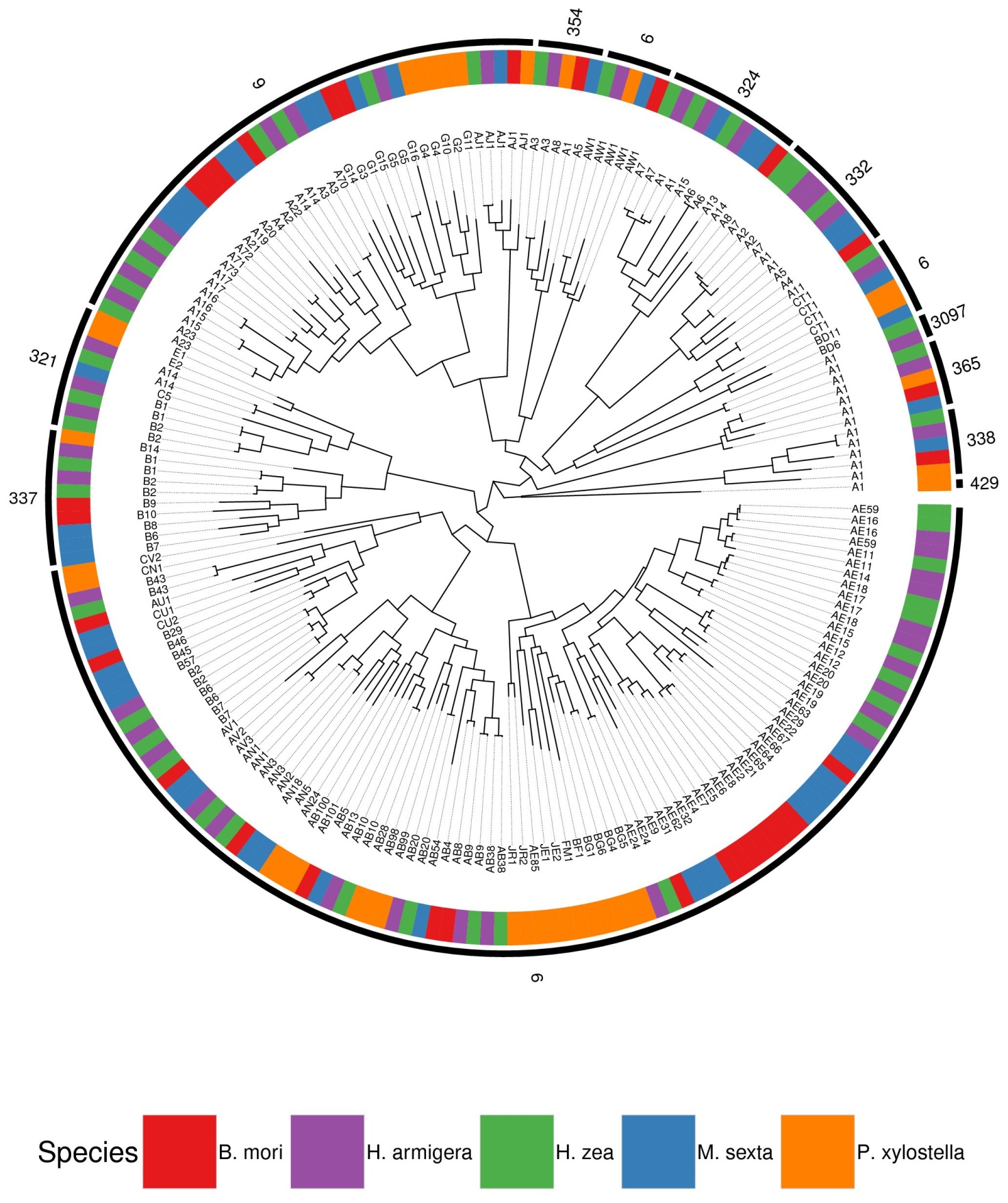


**4**


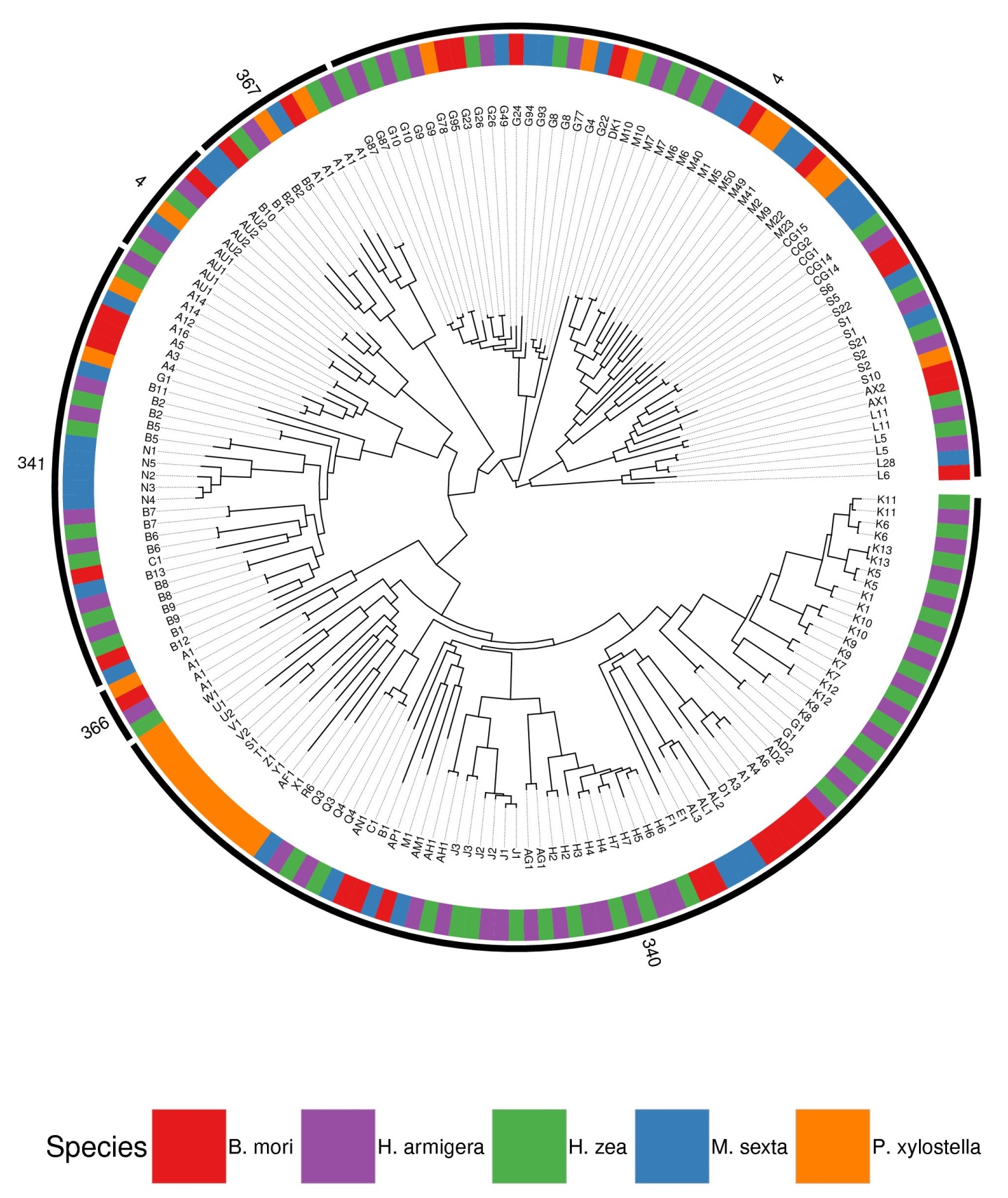


**Figure S10. Transcriptome profiles of the *H. armigera* clan 3 and clan 4 P450s.**

**
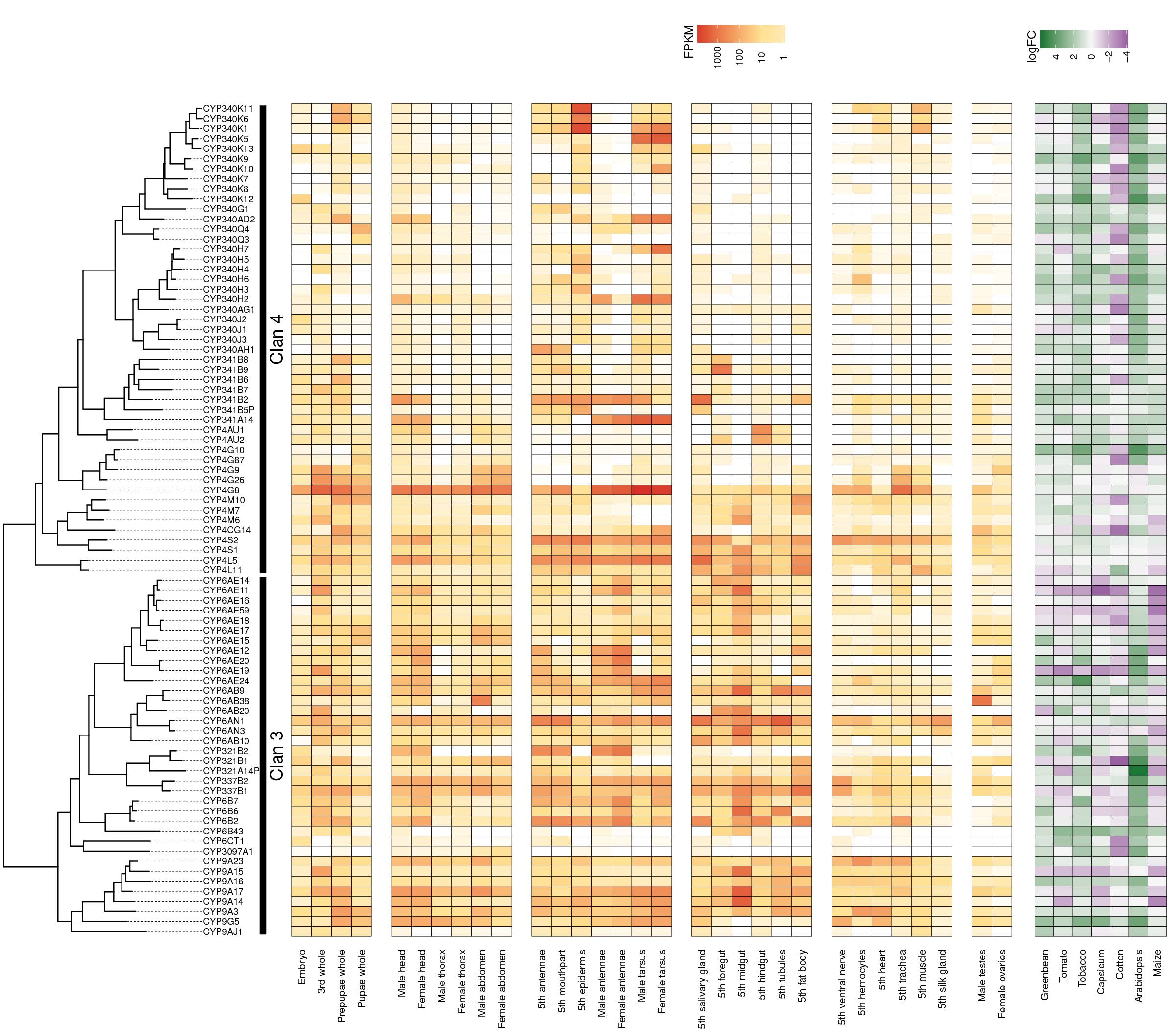
**

**References**

Ali JG, Agrawal AA (2012). Specialist versus generalist insect herbivores and plant defense. Trends Plant Sci. 17, 293-302.

Daimon T, Kozaki T, Niwa R, Kobayashi I, Furuta K, Namiki T, et al. (2012). Precocious metamorphosis in the juvenile hormone deficient mutant of the silkworm, *Bombyx mori*. PLoS Genetics 8, e1002486.

Feyereisen R (2011). Arthropod cypomes illustrate the tempo and mode of P450 evolution. Biochem. Biophys. Acta 1814, 19-28.

Helvig C, Koener JF, Unnithan GC, Feyereisen R (2004). CYP15A1, the cytochrome P450 that catalyses epoxidation of methyl farnesoate to juvenile hormone III in cockcroach corpora allata. Proc. Natl. Acad. Sci. USA 101 4024-4029.

Li Z, Ling L, Zeng B, Xu J, Aslam AF, You L, et al. (2014). CYP18A1 regulates tissue-specific steroid hormone inactivation in *Bombyx mori*. Insect Biochem Molec Biol. 54, 33-41.

Qiu Y, Tittiger C, Wicker-Thomas C, Le Goff G, Young S, Wajnberg E, et al. (2012). An insect-specific P450 carbonic decarbonylase for cuticular hydrocarbon biosynthesis. Proc. Natl. Acad. Sci. USA 109 14858-14863.

Sezutsu H, Le Goff G, Feyereisen R (2013). Origins of P450 diversity. Phil. Trans. Roy. Soc. Lond. Series B, Biol. Sci.368 20121428.

Sztal T, Chung H, Berger S, Currie PD, Batterham P, Daborn PJ (2012). A cytochrome P450 widely conserved in insects in involved in cuticle formation. PLoS ONE 7, e36544.

Willingham AT, Keil T (2004). A tissue specific cytochrome P450 required for the structure and function of Drosophila sensory organs. Mech. Dev. 121, 1289-1297.

Yang P, Tanaka H, Kuwano E, Suzuki K (2008). A novel P450 gene (CYP4G25) of the silkworm Antheraea yamamai: cloning and expression pattern in pharate first instar larvae in relation to diapause. J. Insect Physiol. 54, 636-643.

**Section 2. Detailed analysis of CCEs in *H. armigera*, *H. zea*, *B. mori,* *M. sexta* and *P. xylostella***

We have named the 97 CCEs recovered from the *H. armigera* genome according to the nomenclature system originally developed for a subset of them found among midgut cDNAs from this species and the CCEs recovered from a draft *B. mori* genome by Teese et al. (2010) and Wu et al. (2011). Some reannotation of individual *H. armigera* CCEs from those earlier studies has been necessary given the more robust gene models which the genome data now enables and some have also been renamed in order to maximise the correspondence between the names of orthologs in the different species (Fig. S11; Table S12 and see below). We have then applied the same naming system to the 93 CCEs we found in the *H. zea* genome. We also use the updated annotations of *B. mori* CCEs in Kanost et al. (2016), as well as their annotations of the genomic complements of *M. sexta* and *P. xylostella* CCEs, which again use the same system of nomenclature.

As Fig. S11 shows, the name of each CCE has four elements: firstly two letters identifying the species (eg Ha, Hz, etc); secondly ‘CCE’; thirdly a three number code identifying the clade (001, 002, etc); and finally a single lower case letter uniquely identifying the particular paralog within that species and clade (a, b, c, etc). Orthologous CCEs in each species are given the same names apart from the two first letters identifying the species (eg HaCCE027a, HzCCE027a, etc). However, where the orthology breaks down within a clade, the final letter in the name will vary independently in the different species (eg HaCCE001c and BmCCE001c are not orthologous).

Teese et al. (2010), Durand et al. (2010) and Kanost et al. (2016) have also clustered the lepidopteran CCEs into three high level clade groupings into which Oakeshott et al. (2005, 2010), Claudianos et al. (2006), Strode et al. (2008) and Saad et al. (2015) found dipteran, hymenopteran and some other CCEs could be organised. This integration allowed functional insights for many uncharacterised CCEs to be drawn by extrapolation from empirically determined functional assignments for other, closely related CCEs. We have therefore organised the heliothine CCEs into the same three major clade groupings. However we draw attention in the discussion below to a few discrepancies in the original phylogenetic rationale for the placement of certain clades into these groupings that have become apparent as more genomes have been added to the analyses.

The most distinct of the three groups (clades 027-033) contains a set of proteins which are generally catalytically incompetent (lacking functional catalytic triads, the exception being the acetylcholinesterases (AChEs; clade 027), contain additional membrane-associated domains, and, where known, perform various neuro/developmental functions. This group might not be expected to be associated with host use or metabolic insecticide resistance and indeed all five Lepidoptera included in our analysis have the same complements of this group, involving 13 sets of strictly orthologous sequences. These complements are also very similar to those reported in the dipteran and hymenopteran genomes (Saad et al. 2015), the major differences being an additional set of neuroligin orthologs (clade 030) in the lepidopterans and additional glutactins (clade 031) in the Diptera. Notably two sets of neuroligin orthologs (HaCCE030c and HaCCE030d in *H. armigera*) are tightly linked in a conserved microsyntenous arrangement in all species where the relevant local assemblies permit comparison (Oakeshott et al. 2005; Claudianos et al. 2006 and, for *H. armigera*, see locations given in Additional file 1: Table S1, where the two scaffolds carrying these genes are in fact placed adjacently on chr15 by synteny to *B. mori*). The level of conservation in several clades in this group is such that they have discernible vertebrate orthologs (Oakeshott et al. 2005; Claudianos et al. 2006). Our phylogeny, like that of Kanost et al. (2016), maintains a monophyletic organisation of clades 027-030 and 033 but separates clades 031 and 032 into different parts of the network.

The second group of clades (020-026) has been mainly associated with hormone or semiochemical processing. Our phylogeny splits this group in two, one subgroup comprising clades 020-023 and the other 024-026. The first subgroup includes one clade of known lepidopteran Juvenile Hormone Esterases (JHEs) but intriguingly its other clades all lack functional catalytic triads (in this case without extra membrane-associated domains) and contain no members with known functions. All four of these clades show a high level of orthology within the Lepidoptera but little representation in the Diptera and none in the Hymenoptera, which suggests they have important conserved functions in Lepidoptera but not the other two orders. On the other hand the clades in the second subgroup almost all have competent catalytic triads, are widely distributed across the annotated lepidopteran, dipteran and hymenopteran genomes, and contain some members whose functions have been studied intensively (Oakeshott et al. 2005, 2010; Claudianos et al. 2006). Clades 025 and 026 each consist of a single set of orthologs in the five lepidopterans (except that clade 026 is missing from the *P. xylostella* assembly), although massive amplifications (can be >100 copies) of Clade 026 CCEs have been associated with organophosphate insecticide resistance in certain Hemiptera (Field et al. 1999; Oakeshott et al. 2005 and references therein). There is little orthology and significant variation in the size of clade 024 in the Lepidoptera but no obvious relationship between its size and host range, *P. xylostella* having just one member, *B. mori* two, the two heliothines three, and *M. sexta* four.

The third group of CCEs comprises clades 001 to 019 and 034 and accounts for over two thirds of the CCEs in each genome. Except for clades 13 and 15, which sit in another (albeit only weakly supported) lineage, it consists of relatively recently diverged monophyletic lineages. The great majority of these CCEs have competent catalytic triads, the clades 002 and 003 sequences being some notable exceptions. Enzymes in several of the clades have been implicated in dietary and detoxification functions and the group is therefore labelled as such. However several specific enzymes in clades 006, 008 and 010 in particular have also been linked in functional studies to pheromone or other semiochemical processing, and Durand et al. (2010) have noted that many of the *Spodoptera littoralis* enzymes in this group are expressed at significant levels in both antennae and digestive tissues. This is confirmed by our own transcriptional analyses of the *H. armigera* CCEs (Fig. S12). Orthology across the five lepidopterans is uncommon in this group, largely being confined to small clades such as 002-005 and 010-014. Moreover many of the clades, including the three most numerous ones, clades 001, 006 and 016, are specific to Lepidoptera. The two heliothines and the Solonaceae-specific oligophage *M. sexta* have 67-71 members of this group, while the specialist *B. mori* only has 52, which supports the hypothesised relationship between detoxification gene complements and host range. Within clade 001 in particular, lineage-specific radiations are evident. The great majority of the large radiation in this clade in *M. sexta*, for example, occurred independently of the heliothine radiation. Interestingly however the Brassicaceae-specific oligophage *P. xylostella* only has 41, perhaps suggesting that relatively few plant defence compounds in crucifers are detoxified esteratically.

**Table S12. Distribution of lepidopteran CCEs across functional groups.** All genes were identified as described in Materials and Methods in the main text. Data for *B. mori*, *M. sexta* and *P. xylostella* are taken from Kanost et al. (2016). Files showing full alignments of the five species genomes are available from LSJ, KHJG or JGO on request.*

| Functional groups, *sensu* Teese et al. (2010) | *H. armigera* | | *H.  zea* | | *M.  sexta* | | *B.  mori* | | *P. xylostella* | | Clade(s) |
| --- | --- | --- | --- | --- | --- | --- | --- | --- | --- | --- | --- |
| **Dietary/Detoxification Clades** |  | |  | |  | |  | |  | |  |
| Larval midgut esterases of unknown function | 23 | (2) | 20 | (1) | 27 | (2) | 8 | (1) | 7 | (1) | CCE001 |
| Non-catalytic proteins, membrane-associated | 2 | (2) | 2 | (2) | 2 | (2) | 2 | (2) | 3 | (3) | CCE002 |
| Odorant degrading esterases and related genes | 21 | (1) | 20 | (2) | 16 | (2) | 22 | (2) | 9 |  | CCE006, CCE007, CCE008, CCE010, CCE011 |
| Generally cytoplasmic proteins, including dipteran and hymenopteran resistance genes | 16 | (1) | 16 | (2) | 15 | (1) | 11 |  | 8 |  | CCE016, CCE017, CCE018, CCE019, CCE034 |
| Unknowns | 9 | (2) | 9 | (2) | 7 | (2) | 9 | (3) | 14 | (5) | CCE003, CCE004, CCE005, CCE012, CCE013, CCE014, CCE015 |
| Total | 71 | (8) | 67 | (9) | 67 | (9) | 52 | (8) | 41 | (9) |  |
| **Hormone/Semiochemical Processing Clades** | | | | | | | | | | | |
| Group containing lepidopteran JHE | 3 |  | 3 |  | 5 |  | 4 |  | 4 | (1) | CCE020 |
| Non-catalytic proteins, with endoplasmic reticulum retention signals | 3 | (3) | 3 | (3) | 3 | (3) | 3 | (3) | 3 | (3) | CCE021 |
| Integument esterases | 3 |  | 3 |  | 4 | (1) | 2 |  | 1 |  | CCE024 |
| Dipteran JHE orthologues | 1 |  | 1 |  | 1 |  | 1 |  | 1 |  | CCE025 |
| Pheromone degrading and hemipteran resistance esterases | 1 |  | 1 |  | 1 |  | 1 |  | 1 |  | CCE026 |
| Unknowns | 2 | (2) | 2 | (2) | 2 | (2) | 2 | (2) | 1 | (1) | CCE022, CCE023 |
| Total | 13 | (5) | 13 | (5) | 16 | (6) | 13 | (5) | 11 | (5) |  |
| **Neuro-developmental Clades** | | | | | | | | | | | |
| Acetylcholinesterases | 2 |  | 2 |  | 2 |  | 2 |  | 2 |  | CCE027 |
| Non-catalytic cholinesterase-like adhesion molecules (CLAMs) | 11 | (10) | 11 | (10) | 11 | (10) | 11 | (10) | 11 | (10) | CCE028, CCE029, CCE030, CCE031, CCE032, CCE033 |
| Total | 13 | (10) | 13 | (10) | 13 | (10) | 13 | (10) | 13 | (10) |  |
| Grand Total | 97 | (23) | 93 | (24) | 96 | (25) | 78 | (23) | 65 | (24) |  |

* The numbers of CCEs and their assignments to clades for *B. mori*, *M. sexta* and *P. xylostella* are as in Kanost et al. (2016), except clade 033 was incorrectly assigned to a functional group in Kanost et al. (2016). The names of previously identified *H. armigera* CCEs are generally as per Teese et al. (2010) but with the following differences. Their HaCCE001a, -b and -c become HaCCE01l, -m and -k respectively; their HaCCE001f is subsumed in HaCCE001u and -t; their HaCCE006b, -d and -e become HaCCE006d, -a and -b respectively; their HaCCE016a, -b, -c and -d become HaCCE016c, -f, -j and -g respectively; their HaCCE021b becomes HaCCE021c and is replaced by a new HaCCE021b (ortholog of BmCCE021b); their HaCCE024a becomes HaCCE024c;  their HaCCE033a becomes HaCCE07b and is replaced by a new HaCCE033a. Values in brackets indicate the number of catalytically inactive esterases. These are defined as those sequences lacking at least one of the three canonical catalytic triad residues typical of a serine hydrolase. Whilst lacking a catalytic serine in the expected position, it is notable clade 002 and 030c sequences possess a serine one residue further along in the nucleophilic elbow and otherwise retain the other residues of the catalytic triad as well as all of the structural features otherwise expected of the nucleophilic elbow. It is therefore possible that these esterases possess catalytic activity.

**Figure S11. Phylogeny for the CCEs from the five Lepidoptera.** Phylogenetic methods are described in Materials and Methods in the main text. CCE gene identifiers within clades are shown in upper case for legibility.


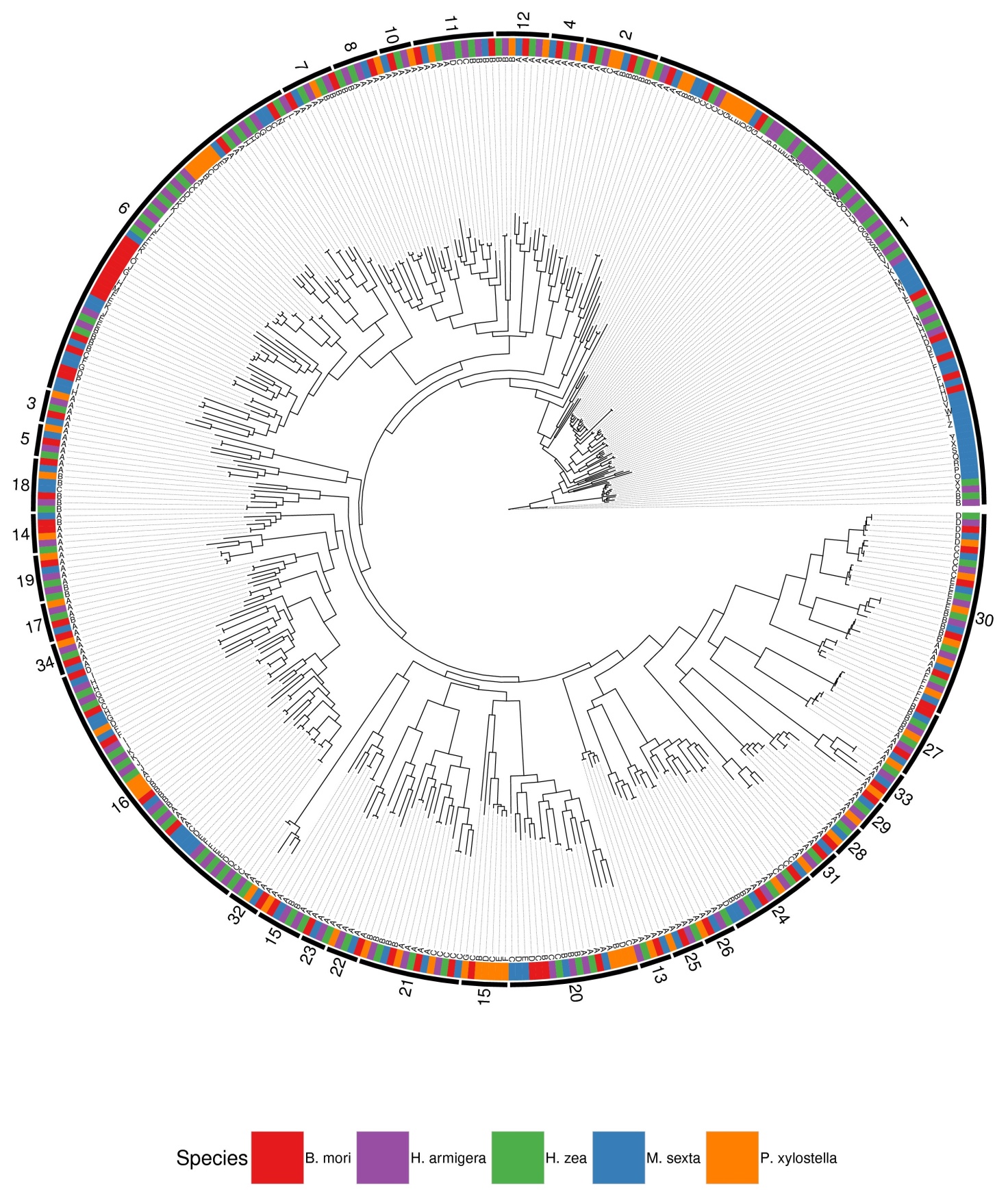


**Figure S12. Transcriptomic profiles for the *H. armigera*** [**CCEs.**](ftp://ftp.csiro.au/Helicoverpa/SI_Figure_2A2.pdf)


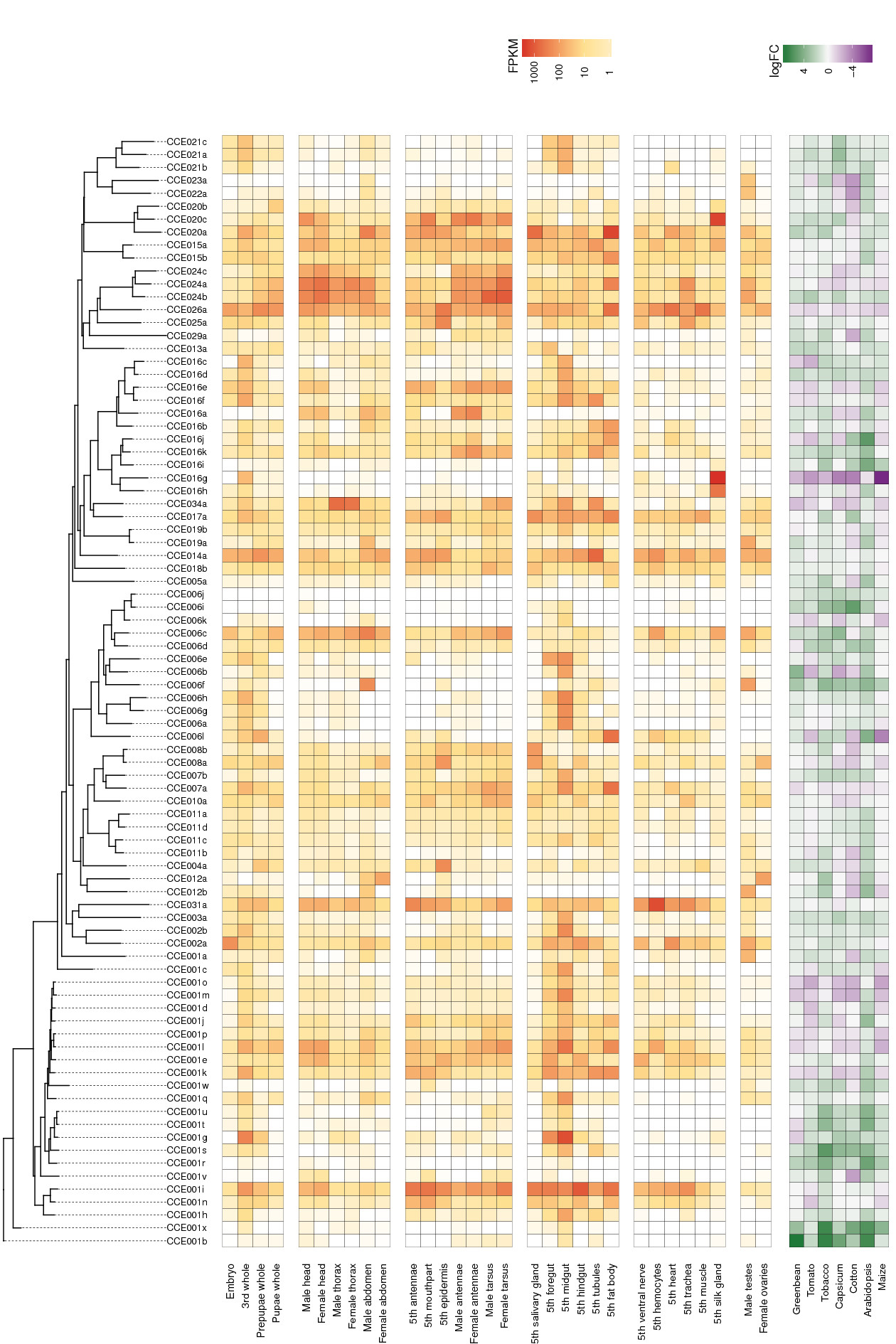


**References**

Claudianos C, Ranson H, Johnson RM, Biswas S, Schuler MA, Berenbaum MA et al. (2006). A deficit of detoxifying enzymes: pesticide sensitivity and environmental response in the honeybee. Insect Mol. Biol. 15, 615-636.

Durand N, Carot-Sans G, Chertemps T, Montagne N, Jacquin-Joly E, Maibeche-Coisne M (2010). A diversity of putative carboxylesterases are expressed in the antennae of the noctuid moth *Spodoptera littoralis*. Insect Mol. Biol. 19, 87-97.

Field LM, Blackman RL, Tyler-Smith C, Devonshire AL (1999). Relationship between amount of esterase and gene copy number in insecticide-resistant *Myzus persicae* (Sulzer). Biochem. J. 339, 737-742.

Kanost MR, Arrese EL, Cao X, Chen Y-R, Chellapilla S, Goldsmith MR et al. (2016). Multifaceted insights from a draft genome sequence of the tobacco hornworm moth, *Manduca sexta*. Insect Biochem. Molec. Biol. 76, 118-147.

Oakeshott JG, Claudianos C, Campbell PM, Newcomb RD, Russell RJ (2005) Biochemical genetics and genomics of insect esterases. In (Gilbert LI, Iatrou K, Gill SS, eds) Comprehensive Insect Molecular Science Vol 5, Pharmacology. Elsevier pp 309-380.

Oakeshott JG, Johnson RM, Berenbaum MR, Ranson H, Cristino AS, Claudianos C (2010) Metabolic enzymes associated with xenobiotic and chemosensory responses in *Nasonia vitripennis*. Insect Mol. Biol. 19 (Supp. 1), 147-163.

Saad BM, Barribeau SM, Bloch G, de Graaf DC, Dearden P, Elsik CG (2015) The genomes of two bumblebee species with primitive eusocial organisation. Genome Biol. 16, 10.1186/s 13059-015-0623-3.

Strode C, Wondji CS, David JP, Hawkes NJ, Lumjuan N, Nelson DR (2008). Genomic analysis of detoxification genes in the mosquito *Aedes aegypti*. Insect Mol. Biol. 38, 113-123.

Teese MG, Campbell PM, Scott C, Gordon KHG, Southton A, Hovan D et al. (2010). Gene identification and proteomic analysis of the esterases of the cotton bollworm, *Helicoverpa armigera*. Insect Biochem. Molec. Biol. 40, 1-16.

Wu S, Yang Y, Yuan G, Campbell PM, Teese MG, Russell RJ, Oakeshott JG et al. (2011). Overexpressed esterases in a fenvalerate resistant strain of the cotton bollworm, *Helicoverpa armigera*. Insect Biochem. Molec. Biol. 41, 14-21.

**Section 3. Detailed analysis of GSTs in *H. armigera*, *H. zea*, *B. mori*, *M. sexta* and *P. xylostella***

GSTs are substantially more numerous in the genomes of the two polyphagous heliothines than they are in the genomes of the other three lepidopterans for which high quality manual annotations are available. The differences are entirely due to expansions in two of the five GST classes (Table S13, Fig. S13), specifically the delta and sigma classes associated with host use (Shi et al., 2012; Enayati et al., 2005, Rane et al. 2016) and resistance to insecticides (Ranson and Hemingway 2005, Dukre et al. 2009, You et al. 2015) in a wide range of species, including lepidopterans. Interestingly one of the two oligophages, *M. sexta*, also has a smaller expansion in the sigma class, although the other, *P. xylostella*, does not.

The expansion of the delta class in the heliothines arises because of duplication events in several delta lineages, although the radiation in one lineage (the GSTs D1h-D1l) accounts for about half the difference. The number of duplication events in the delta class expansion means that sequence divergence across the class is substantial, with amino acid identities in the range 26% to 98% across the whole class.

The sigma expansion in the heliothines is due to a single radiation and interestingly this occurs in the same lineage as the *M. sexta* radiation, albeit more recently than the latter. However there is still considerable post-duplication divergence in the sigma class, with amino acid identities ranging over 26 – 79 % in the heliothine case. Not all the heliothine GSTs have all the consensus residues known to be required for catalytic activity, with some in the GSTD, GSTO and GSTS classes having variants that may be inactive. HarmGSTD1a and HzeaGSTD1a, like those from the other three Lepidoptera, appear to have a glycine replacing the active site serine; glycine is unlikely to be catalytic but the action of another serine, tyrosine or arginine in the region might contribute to activity. For all five species analysed, GSTO3 lacks the active site cysteine normally found in the GSTO class; it appears to have been replaced by asparagine, based on the latter’s location adjacent to the conserved proline residue. Finally, HarmGSTS1k and HzeaGSTS1k, which have no orthologs among the other three lepidopteran genomes, lack active site tyrosines and may be inactive, although a conserved arginine in the active site region could conceivably contribute to catalytic activity.

Across the various hosts tested in the diet response experiment (see Fig. S14), only Arabidopsis elicits any increase for most of the GSTD and GSTS genes, although it is only very slight for most of these genes. Maize as a diet generally elicits a decline in GST expression where there is any change at all, whereas the other hosts tested saw mixed effects on GST gene expression. Some clades showed evidence of coordinated expression responses, with the expansion noted above (GSTD1h-k plus GSTD1n) showing significant reductions on all hosts other than Arabidopsis. The clade comprising GSTD1p, -c and -b was another one showing coordinated expression declines on all hosts, but other clades showed variable gene expression responses, indicating possible neofunctionalization within these clades.

**Table S13. Distribution of lepidopteran GSTs across major classes.** All genes were identified as summarised in Materials and Methods in the main text. Data for *B. mori*, *M. sexta* and *P. xylostella* are taken from Kanost et al. (2016). Files showing full alignments of the five species genomes are available from LSJ or KHJG on request.

| Class | *H. armigera* | *H. zea* | *B. mori* | *M. sexta* | *P. xylostella* |
| --- | --- | --- | --- | --- | --- |
| Delta | 25 | 24 | 14 | 16 | 14 |
| Sigma | 11 | 10 | 2 | 8 | 2 |
| Theta | 1 | 1 | 1 | 1 | 1 |
| Zeta | 2 | 2 | 2 | 2 | 2 |
| Omega | 3 | 3 | 4 | 4 | 5 |
| Total | 42 | 40 | 23 | 31 | 24 |

**Figure S13. Phylogeny of the GSTs across the five Lepidoptera.** Phylogenetic methods are described in Materials and Methods in the main text.

**
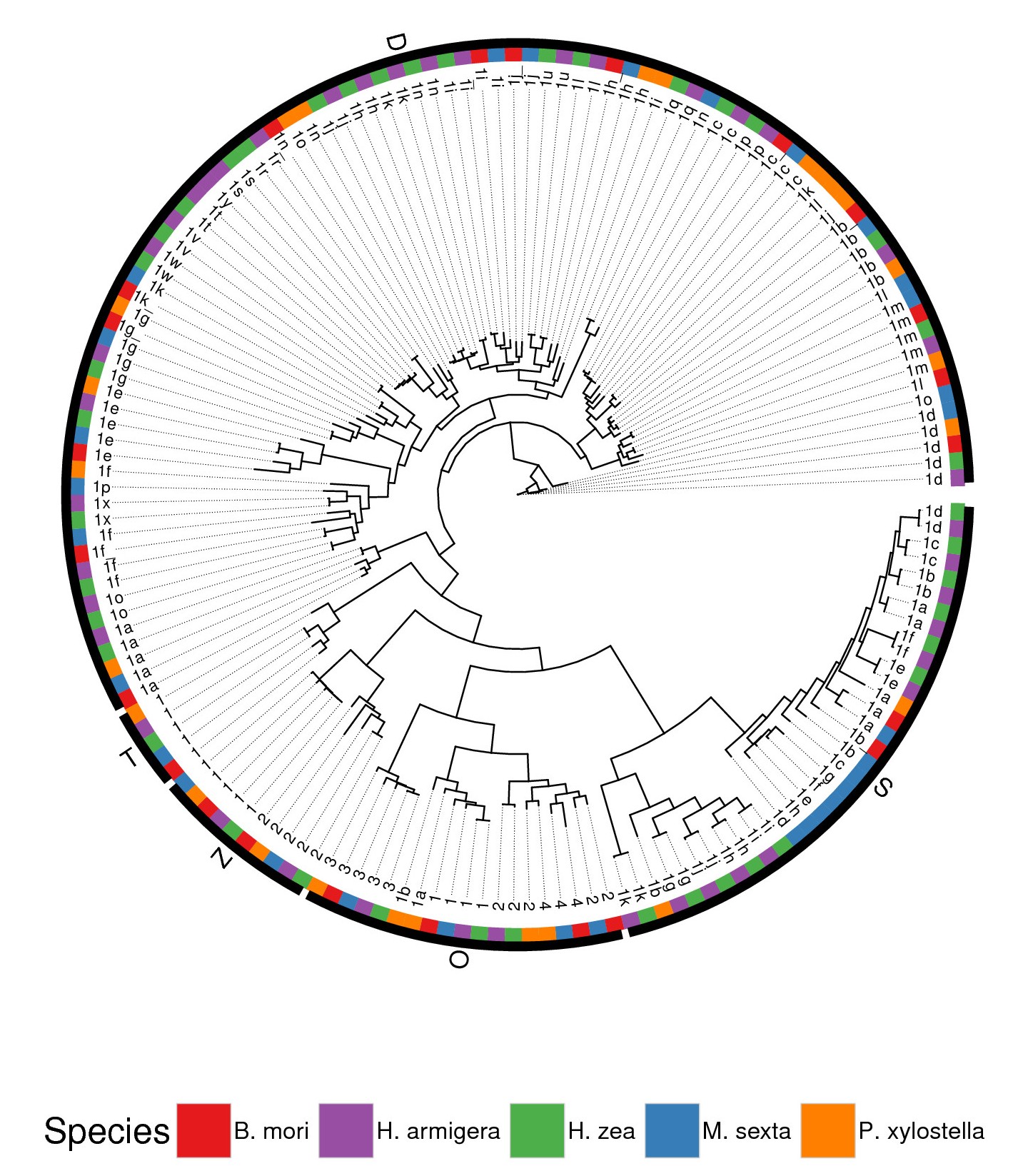
**

**Figure S14. Transcriptomic profiles of the *H. armigera*** [**GSTs.**](ftp://ftp.csiro.au/Helicoverpa/SI_Figure_3A2.pdf)

**
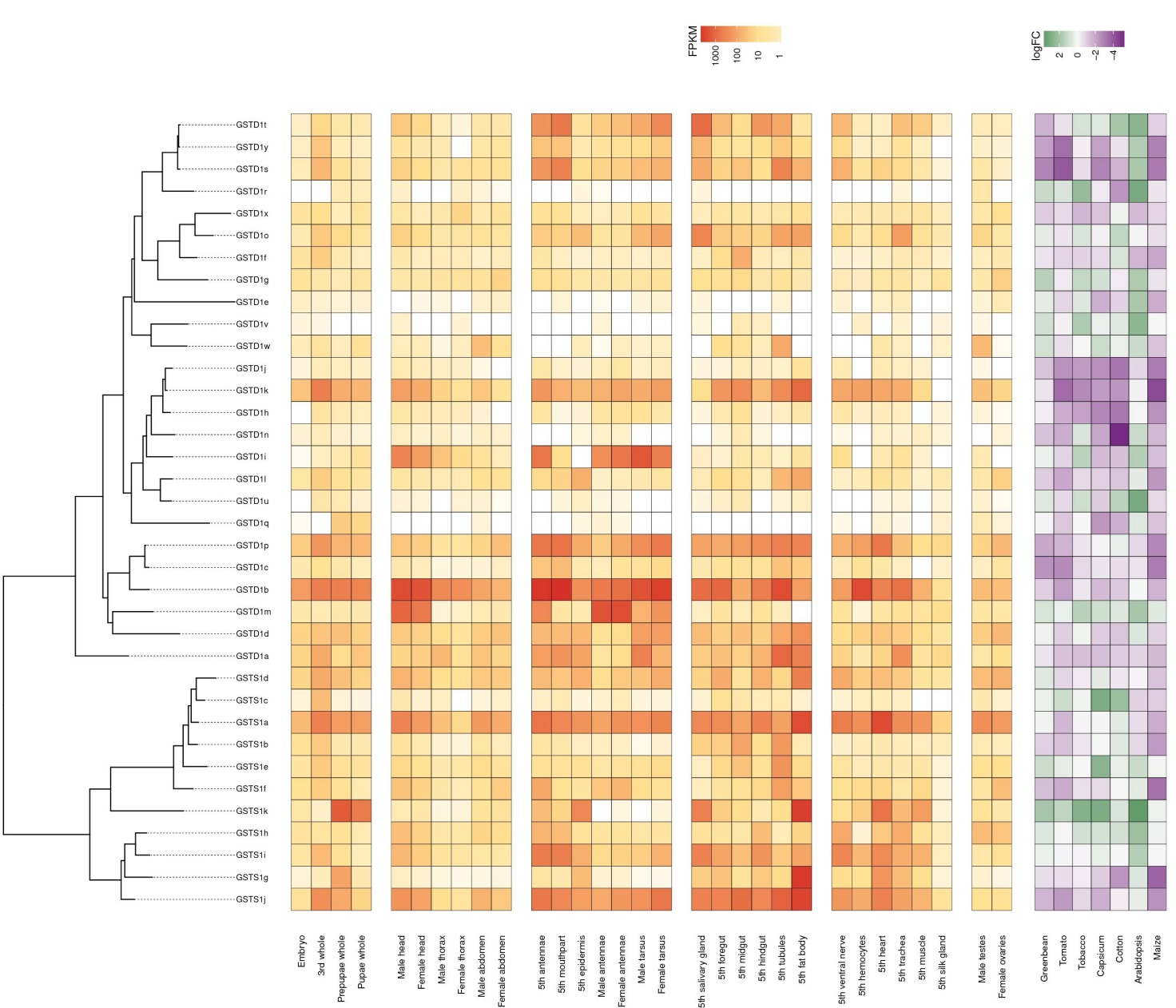
**

**.**

**.**

**References**

Dukre AS, Moharil MP, Ghodki BS, Rao NGV (2009). Role of glutathione S-transferase in imparting resistance to pyrethroids in Plutella xylostella (L.). Int. J. Integr. Bio. 6,17-21.

Enayati AA, Ranson H, Hemingway J (2005). Insect glutathione transferases and insecticide resistance. Insect Molec. Biol. 14, 3-8.

Rane, R.V., Walsh, T.K., Pearce, S.L. Jermiin, L.S., Gordon, K.H.J., Richards, S., Oakeshott, J. G. (2016) Are feeding preferences associated with the size of detoxifying enzyme families in insect herbivores? Curr. Opin. Insect Sci. 13, 70-76.

Ranson H, Hemingway J. (2005) Glutathione Transferases. In (Gilbert LI, Iatrou K, Gill SS, eds) Comprehensive Molecular Insect Science. Elsevier, Amsterdam, Vol 5, pp 383-402.

Shi H, Pei L, Gu S, Zhu S, Wang Y, Zhang Y, Li B (2012). Glutathione S-transferase (GST) genes in the red flour beetle, Tribolium castaneum, and comparative analysis with five additional insects. Genomics 100, 327-335.

# You Y, Xie M, Ren N, Cheng X, Li J, Ma X, Zou M, et al. (2015). Characterization and expression profiling of glutathione S-transferases in the diamondback moth, Plutella xylostella (L.) BMC Genomics 16, 152.

**Section 4. Detailed analysis of UGTs in *H armigera*, *H. zea*, *B. mori* and *M. sexta.***

The overall numbers of UGT genes in the two *Helicoverpa* species are very similar to those in *B. mori* and *M. sexta* but there are significant differences between the species in particular clades (Table S14, Fig. S15**)**. In particular the UGT33 family is relatively large in *H. armigera*, and to a lesser extent *H. zea*, due to an expansion of the cluster on scaffold_562, while the UGT40 family is relatively large in *B. mori* and UGT340 is relatively large in *M. sexta*. The extensive expansion of the UGT33 family in *H. armigera* resulted in genes with sequence divergences ranging from 54 – 97 %.

Across the various hosts tested in the diet response experiment (see Fig. S16), only Arabidopsis elicits any increase for most of the UGT genes, particularly for the UGT33 and UGT40 families. Many genes in these two families showed considerable variation in their transcriptional responses to different hosts. Some recently duplicated clades showed evidence of coordinated expression responses on all hosts tested, e.g. UGT40F1/2 or UGT33F1A/B.

**Table S14.** **UGT families in *H armigera*, *H. zea*, *B. mori* and *M. sexta*.** The *B. mori* gene numbers are corrected from those in Ahn et al. (2012) according to our comprehensive re-analysis as summarised in Materials and Methods in the main text. The *M. sexta* genes are taken from Kanost et al. (2016). Files showing full alignments of the four species’ UGTs are available from LSJ or KHJG on request.

| UGT family | *H. armigera* | *H. zea* | *B. mori* | *M. sexta* |
| --- | --- | --- | --- | --- |
| UGT33 | 22 | 19 | 13 | 16 |
| UGT34 | 1 | 1 | 1 | 1 |
| UGT39 | 1 | 1 | 2 | 1 |
| UGT40 | 8 | 7 | 12 | 9 |
| UGT41 | 4 | 4 | 3 | 1 |
| UGT42 | 2 | 2 | 3 | 3 |
| UGT43 | 1 | 1 | 1 | 0 |
| UGT44 | 1 | 1 | 1 | 1 |
| UGT45 | 0 | 0 | 0 | 1 |
| UGT46 | 3 | 3 | 3 | 2 |
| UGT47 | 1 | 1 | 1 | 1 |
| UGT48 | 1 | 1 | 1 | 1 |
| UGT50 | 1 | 1 | 1 | 1 |
| UGT340 | 0 | 0 | 2 | 6 |
| Total | 46 | 42 | 44 | 44 |

**Figure S15. Phylogeny of the UGTs across *H. armigera*, *H. zea*, *B. mori* and *M. sexta*.** Phylogenetic methods are described in Materials and Methods in the main text.


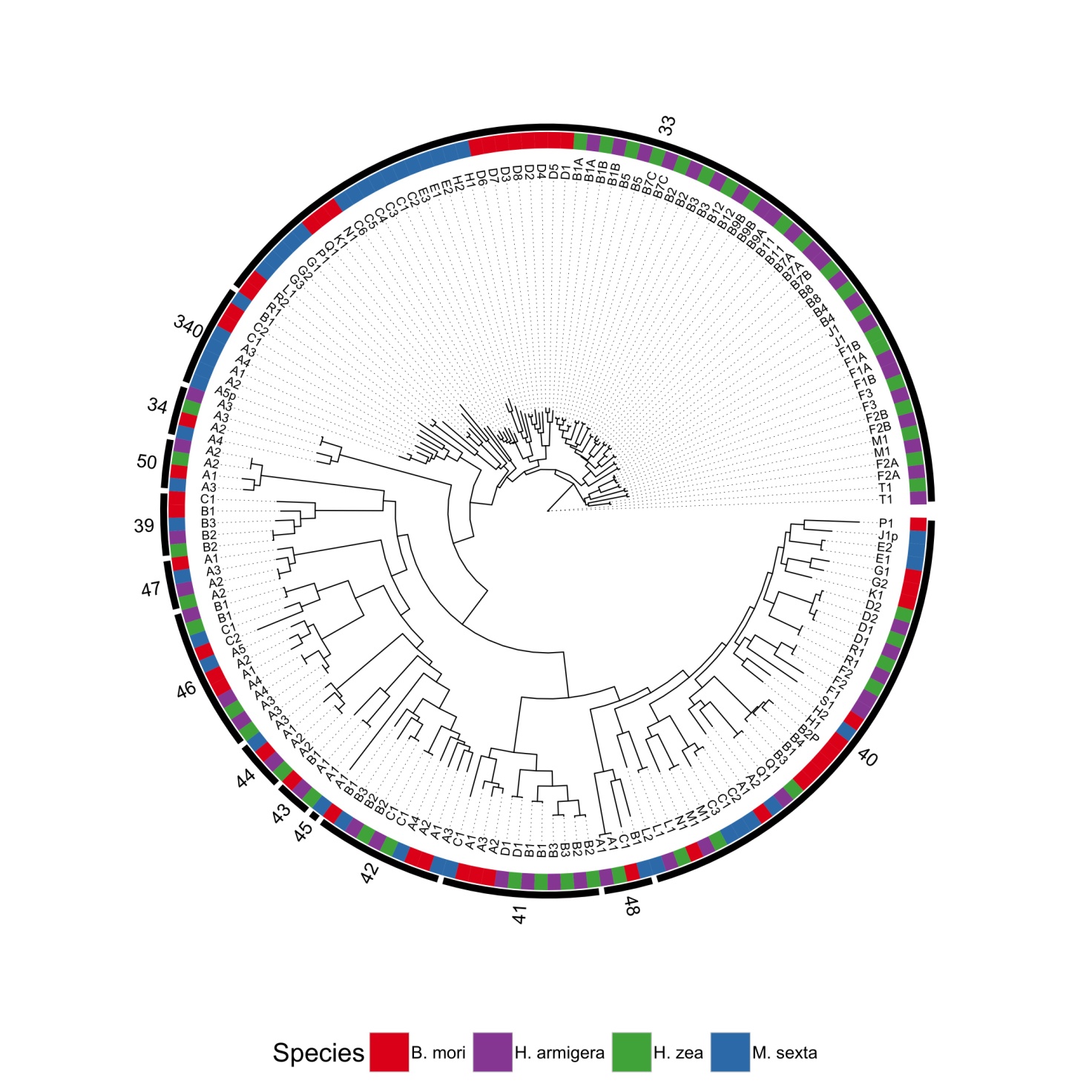


**Figure S16. Transcriptomic profiles of the *H. armigera* UGTs.**

**
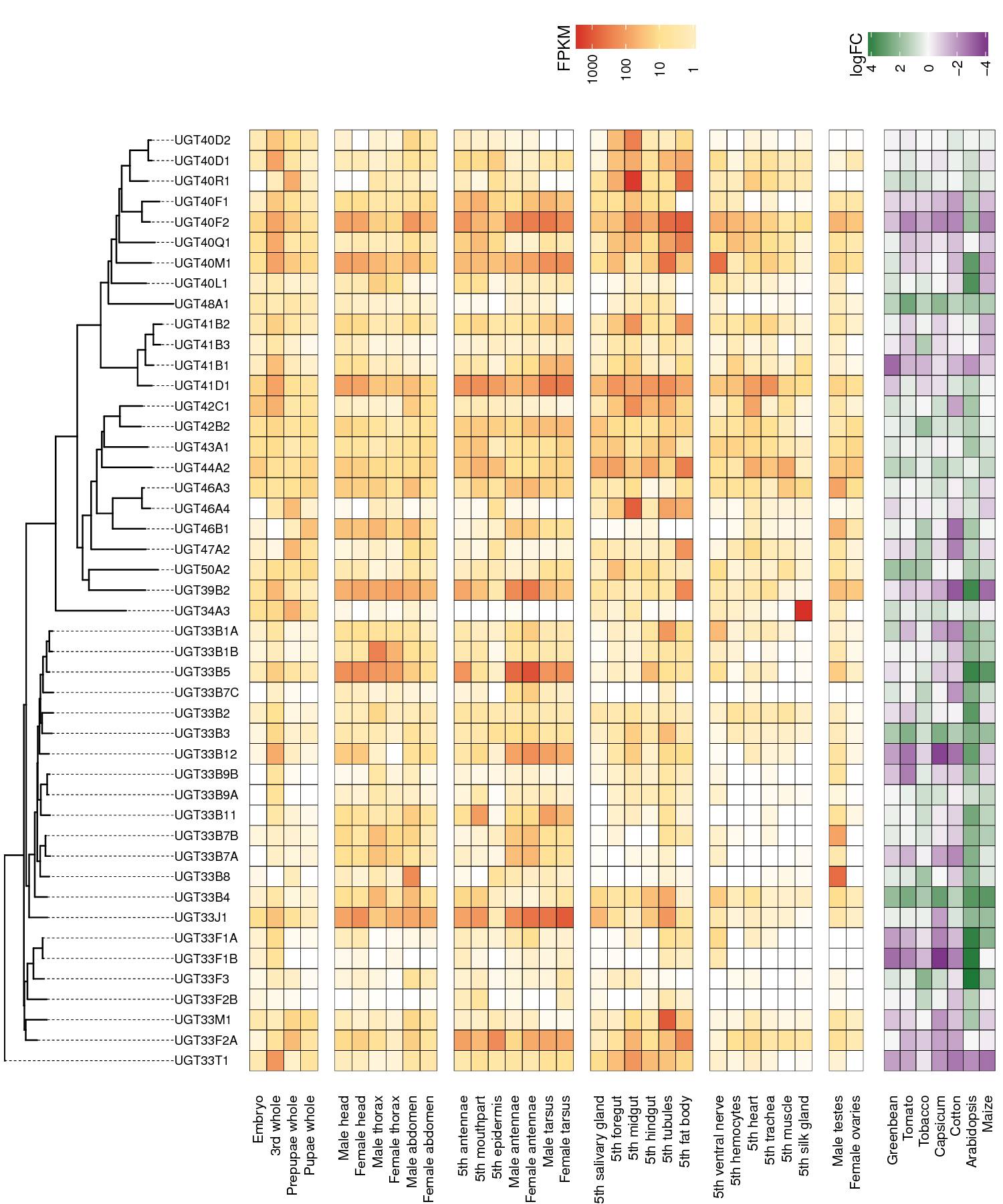
**

**References**

Ahn S-J, Vogel H, Heckel DG. (2012). Comparative analysis of the UDP-glycosyltransferase multigene family in insects. Insect Biochem. Mol. Biol. 42,133–147.

Kanost MR, Arrese EL, Cao X, Chen Y-R, Chellapilla S, Goldsmith MR et al. (2016). Multifaceted insights from a draft genome sequence of the tobacco hornworm moth, *Manduca sexta*. Insect Biochem. Molec. Biol. 76, 118-147.

**Section 5. Detailed analysis of ABC transporters in *H armigera*, *H. zea*, *B. mori*, *M. sexta* and *P. xylostella***

The ATP-binding cassette (ABC) superfamily of proteins contains a number of membrane-bound, ATP-driven transporters that are involved in cellular transport, Phase III detoxification and other cellular processes (Broehan et al. 2013). They can be divided into several sub-families (A-H) and, in general, they comprise two transmembrane domains and two cytoplasmic nucleotide-binding domains that function to hydrolyze ATP. ABC transporters play a role in pesticide detoxification, transporting metabolised compounds out of cells and are directly implicated in resistance to Cry toxins as binding sites in *Lepidoptera*  (Dermauw et al. 2014; Tabashnik 2015; Tay et al. 2015). The ABC complement in both *H. armigera* and *H. zea* is slightly larger than that of *B. mori* with 54 genes identified in both species and small expansions have occurred in subfamilies B and G (Table S15). Other Lepidoptera show very similar numbers for each subfamily, apart from the expansions reported for most of them, especially subfamilies A and C, in *P. xylostella* (Qi et al. 2016; see Table S15). Overall, synteny is highly conserved between the species and one to one orthologs exist in all cases between *H. armigera* and *H. zea*. All the othologue pairs between these two species yield Ka/Ks ratios less than 0.1 (summarised in Table S15), suggesting that they evolving more slowly than some of the other detoxification gene families considered here.

Expression of these genes in *H. armigera* does vary somewhat between developmental stages, with some genes showing expression generally and some with more tissue specific expression patterns (Fig. S17). One striking example of the latter is ABCA3 (HaOG200339) which is only expressed in testes, prepupae, pupae and hindguts. In contrast ABCG12 (HaOG200311) is widely expressed in all 31 tissue types and stages examined. Interestingly ABC transporter genes thought to be involved in resistance to Bt toxins are expressed in the midgut where Bt toxins are thought to act (ABCA2 - HaOGS200329, ABCA2 - HaOGS200331). Furthermore, seven ABC genes significantly regulated in response to host plant (Fig. S17) are also identified as expressed in the intestinal tissue. One of these genes is the Cry2Ab resistance associated gene ABCA2 which perhaps has implications for sensitivity to Cry2Ab related to host plant.

The ABC transporters also showed significant variation in transcriptional response to the various hosts plants tested in this study (Fig. S17). The six genes showing most significant DE were from three subfamilies, two each from the A, B, and C subfamilies. No host failed to elicit any significant effect, but all differed, with different blocks of ABC genes down-regulated on tobacco, capsicum, cotton and arabidopsisis in particular, and up-regulated on maize. The two members of the ABCB subfamily, one strongest on maize (HaOG200349-ABCB1) and the other on cotton (HaOG200306-ABCB3), were among those identified by Bretschneider et al. (2016) as significantly upregulated in response to diet additives (to tomatine, and nicotine and taxol, respectively). The gene identified by Bretschneider et al. (2016) as a general secondary response gene (HaOG200350-ABCB3) was mildly upregulated but not significantly so on the hosts studied in the present work.

**Table S15. ABC numbers in *H. armigera*, *H. zea, B. mori, M. sexta* and *P. xylostella.*** Note that whereas the numbers for the first three species are from our own analysis and curations as described in Materials and Methods in the main text, the number for *M. sexta* and *P. xylostella* have been taken from Qi et al. (2016), who used different gene finding and curation methods. Alignment files for the first three species are available from KHJG on request.

| Sub-family | *H. armigera* | *H. zea* | *Ha-Hz Ka/Ks* | *B. mori* | *M. sexta* | *P. xylostella* |
| --- | --- | --- | --- | --- | --- | --- |
| A | 7 | 7 | 0.036 | 7 | 7 | 15 |
| B | 11 | 11 | 0.033 | 9 | 9 | 14 |
| C | 11 | 11 | 0.019 | 11 | 11 | 21 |
| D | 2 | 2 | 0.0053 | 2 | 2 | 3 |
| E | 1 | 1 | 0.001 | 1 | 1 | 1 |
| F | 3 | 3 | 0.0078 | 3 | 3 | 3 |
| G | 17 | 17 | 0.0087 | 16 | 16 | 19 |
| H | 2 | 2 | 0.0062 | 2 | 3 | 6 |
| Total | 54 | 54 |  | 51 | 52 | 82 |

**Figure S17. Transcriptome profiles of the *H. armigera* ABCs in subfamilies A, B, C and G.** The phylogeny on the left reflects these four subfamilies, showing genes assigned to each.

**
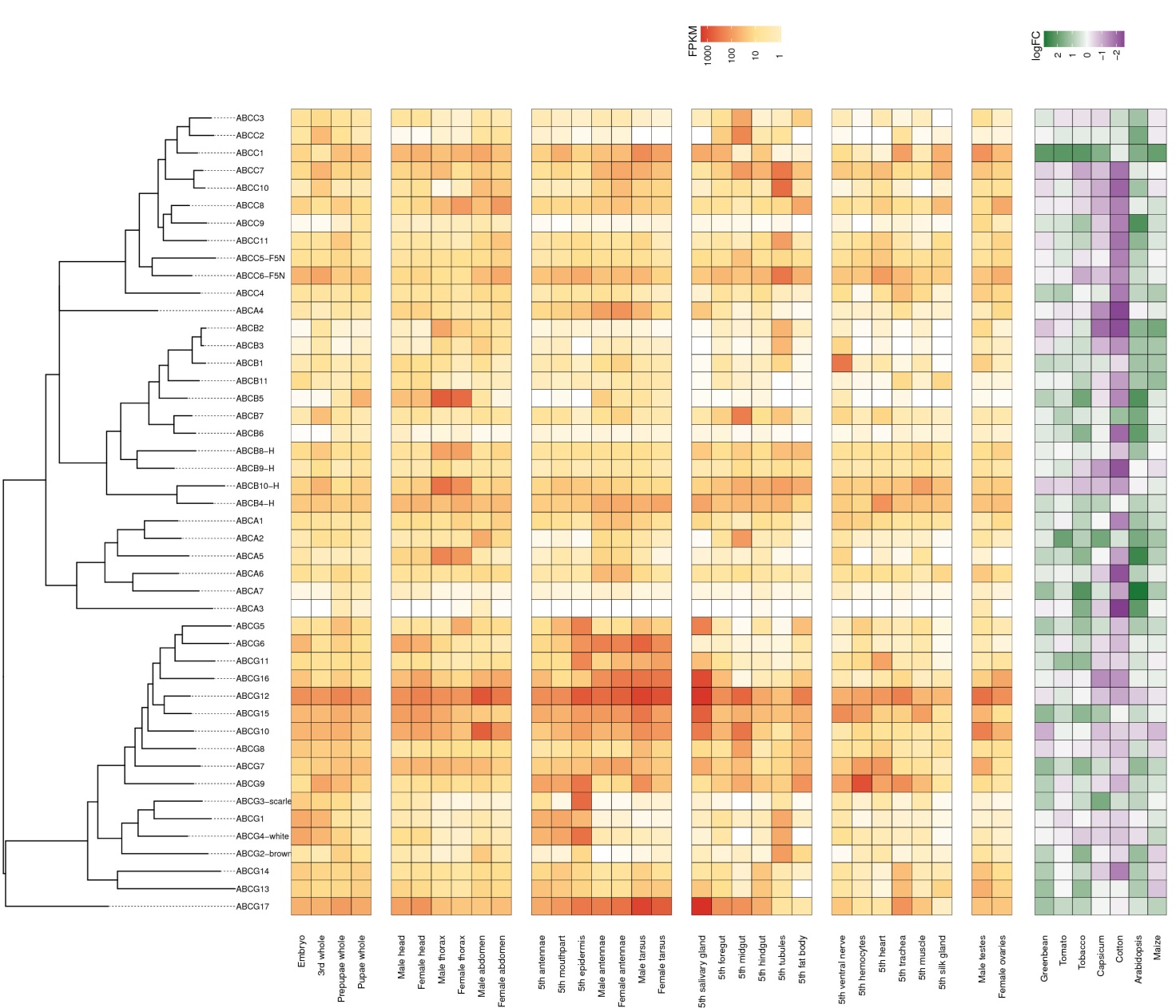
**

**References**

Bretschneider A, Heckel DG, Vogel H (2016). Know your ABCs: characterisation and gene expression dynamics of ABC transporters in the polyphagous herbivore *Helicoverpa armigera*. Insect Biochem. Molec. Biol. 72, 1-9.

Broehan G, Kroeger T, Lorenzen M, Merzendorfer H (2013). Functional analysis of the ATP-binding casette (ABC) transporter gene family of *Tribolium castaneum*. BMC Genomics 14, 6.

Dermauw, W., Van Leeuwen, T., 2014. The ABC gene family in arthropods: comparative genomics and role in insecticide transport and resistance. Insect Biochem. Mol. Biol. 45, 89–110.

Gahan LJ, Pauchet Y, Vogel H, Heckel DG. (2010) An ABC transporter mutation is correlated with insect resistance to *Bacillus thuringiensis* Cry1Ac toxin. Plos Genet. 6 PubMed PMID: ISI:000285578900026.

Park Y, Gonzalez-Martinez R, Navarro-Cerrillo G, Chakroun M, Kim Y, Ziarsolo P, et al. (2014) ABCC transporters mediate insect resistance to multiple Bt toxins revealed by bulk segregant analysis. BMC Biol. 12, 46.

Qi W, Ma X, He W, Chen W, Zou M, Gurr GM, Vasseur L, You M. (2016). Characterization and expression profiling of ATP-binding cassette transporter genes in the diamondback moth, Plutella xylostella (L.). BMC Genomics 17:760.

Sturm A, Cunningham P, Dean M. (2009). The ABC transporter gene family of Daphnia pulex. BMC Genomics. 10,170.

Tabashnik BE (2015). ABCs of insect resistance to Bt. PLoS Genet 11, e1005646.

Tanaka S, Miyamoto K, Noda H, Jurat-Fuentes JL, Yoshizawa Y, Endo H, et al. (2013). The ATP-binding cassette transporter subfamily C member 2 in *Bombyx mori* larvae is a functional receptor for Cry toxins from *Bacillus thuringiensis*. FEBS J. 280,1782-1794.

Tay WT, Mahon RJ, Heckel DG, Walsh TK, Downes S, James WJ, et al. (2015). Insect resistance to Bacillus thuringiensis toxin Cry2Ab is conferred by mutations in an ABC transporter subfamily A protein. PLoS Genet. 11, e1005534.

**Section 6. Detailed analysis of midgut serine proteases in *H. armigera*, *H. zea* and *B. mori***

The serine proteases comprise a large family of proteins in insects that serve multiple functions in digestion, defence and regulation of various developmental processes (Zou et al. 2006, Ross et al. 2003). In lepidopteran insects the serine proteases are the predominant digestive endopeptidases, contributing around 95% of the proteolytic activity (Srinivasan et al. 2006). It is likely that polyphagous insects such as *H. armigera* have evolved a diverse repertoire of digestive proteases to allow for qualitative differences in the protein composition of their diet at different developmental stages or when feeding on different host plants (Patankar et al. 2001). In particular, it has been suggested that structural and functional diversity of digestive proteases provides a mechanism whereby insects can circumvent host plant defense mediated through damage-induced expression of protease inhibitors (Broadway 1995, 1996). In *H. armigera* larvae, inclusion of plant protease inhibitors in laboratory diet has been shown to alter the expression pattern of midgut proteases (Bown et al. 1997, 2004, Kuwar et al. 2015). Levels of both trypsin- and chymotrypsin-like enzymes were affected, with some genes being up-regulated and others down-regulated over time. Interestingly, the expression of serine protease homologs that are presumed to be catalytically inactive due to the absence of one or more residues of the catalytic triad was also altered in response to the presence of protease inhibitors in the diet (Bown et al. 1997, Kuwar et al. 2015), implicating these proteins of unknown function as part of the adaptive response. A more detailed understanding of the interaction between digestive proteases and dietary protease inhibitors, and the mechanism underlying the differential regulation of midgut protease expression in response to the presence of inhibitors has been limited by our lack of knowledge of the full complement of these enzymes in *H. armigera*. The availability of a high quality genome assembly now allows us to address this long-standing problem.

**The *H. armigera* gene sets**

Detailed manual curation of the serine proteases was limited to the sub-family of presumptive digestive enzymes. Genes were identified by taking the complete set of serine protease-like sequences that were automatically annotated by the Maker and PASA pipelines and then filtering that set according to the following criteria; presence of the His-Asp-Ser catalytic triad, presence of a canonical zymogen-activation cleavage site (RIVGG), single-domain sequence, and each gene, or at least one gene in each tandem cluster, was expressed in the larval midgut. Genes that met these criteria were then sub-divided into putative trypsin or chymotrypsin-like sequences on the basis of substrate specificity inferred from the amino acid present at position 189 (based on amino acid numbering relative to bovine chymotrypsin). Enzymes were designated as trypsin-like if the residue at position 189 was D, E, R, L, T, or as chymotrypsin-like if the residue was G, S or N. Sequences were classified as catalytically active if there was a H residue at amino acid position 57, a D residue at position 102 and a S residue at position 195 (all numbering relative to bovine chymotrypsin), or as inactive if at least one of these catalytic triad residues was mutated. This process yielded a set of 62 trypsin-like and 53 chymotrypsin-like sequences. For completeness, this set of 115 genes was used to search two independent *H. armigera* genome assemblies using the BLAST algorithm to check for any additional complete or partial protease genes.

Where possible all gene models were confirmed by comparison to previously sequenced full-length cDNAs or transcripts derived by *de novo* assembly from the larval tissue and developmental-stage RNA-Seq database. In general genomic models were accurate for genes occurring in single copy or as members of small (two to four gene) clusters. However, the larger tandem gene clusters in the CSIRO4b assembly frequently had multiple assembly errors. Correction of these errors was achieved by searching several independent genome assemblies for correctly assembled versions of the relevant gene. A model was considered correct if identical sequences, with conserved flanking genes in the same orientations, could be recovered from at least two computationally independent (e.g. AllPaths and CABOG) assemblies. Prediction of the very large Clade 1 trypsin and chymotrypsin clusters also drew on BAC assemblies which, although not correctly assembled, did indicate that these genes occur as multiple, structurally distinct haplotypes in the *H. armigera* genome. Accordingly, the gene cluster as presented in the final version of the corrected assembly represents a consensus sequence set that includes all of the genes that could be identified from different versions of the cluster. For these two large tandem gene clusters, no single haplotype could be unambiguously assembled. The genes identified are listed in Table S16 and the phylogenetic analysis of genes and clusters is given in Fig. S18.

Of the 51 trypsin-like sequences, over half (29) occurred in the single large Clade 1 cluster and fifteen sequences were classified as catalytically inactive. Three of these occurred in the genome as single genes, while the remainder belonged to tandem gene clusters, with the largest comprising 7 genes on scaffold_9. A single gene (HaOG200450) within the 29 gene Clade 1 cluster was inactive. For the 49 midgut chymotrypsin-like sequences, four were predicted to be catalytically inactive. Of these, one occurred as a single gene and the other three were each a member of three spatially distinct clusters, including one gene from the large 26 gene Clade 1 cluster. Transcription analysis on different hosts shows a degree of correlated expression for subsets of the genes in the large clusters (Fig. S19).

**Gene sets for *H. zea* and *B. mori***

The *H. zea* genes were identified in the *H. zea* OGS on the basis of their orthologies with the *H. armigera* gene set, as outlined in the main text. Of the 51 trypsins and 49 chymotrypsins identified in the *H. armigera* genome, 46 and 44, respectively, had orthologs in the *H. zea* genome (Table 2 of the main text and Fig. S18). All of the three trypsin-like and five chymotrypsin-like sequences present in *H. armigera* but absent from the *H. zea* assembly belonged to multi-gene clusters and their apparent absence in *H. zea* may be due to assembly problems in those clusters. No gene was found in *H. zea* that did not have a clear ortholog in *H. armigera*. All proteins identified as catalytically inactive in *H armigera* were also inactive in *H. zea* and no correctly assembled proteases were predicted to be inactive in *H. zea* but active in *H. armigera*.

The *B. mori* genome appears to have a greatly reduced complement of digestive proteases compared to *H. armigera*. Only 17 trypsin-like and 28 chymotrypsin-like sequences related to genes in *H. armigera* were recovered from the *B. mori* genome. Much of the difference can be accounted for by the massive expansion of the Clade 1 trypsin and chymotrypsin clusters in *H. armigera*. The Clade 1 trypsin cluster in *B. mori* contained only 7 genes compared to 29 in *H. armigera*, and the Clade 1 chymotrypsin cluster in *B. mori* had only 3 genes compared to 26 genes in *H. armigera* (Fig. S18). Evolution of protease genes by duplications giving rise to tandem arrays is common in both *H. armigera* and *B. mori*, but the arrays tend to be larger in *H. armigera*. Apart from the major Clade 1 expansions, the major expansions of trypsins in the heliothine pests were on scaffold_0 and scaffold_113, and scaffold_9; and for chymotrypsins they were on scaffold_9. Interestingly, *B. mori* showed evidence for two modest expansions of chymotrypsin genes, on scaffold_247 and scaffold_393 (Fig. S18). For all proteases identified as catalytically inactive in *B. mori,* the corresponding *H. armigera* ortholog was also catalytically inactive, including a tandem cluster of trypsin-like sequences containing multiple inactive enzymes.

**Table S16.** **Summary of numbers and locations of digestive serine protease genes in *H. armigera*, *H. zea* and *B. mori.*** Details for individual genes are given in Additional file 6: Table S5. Genes were identified as in Materials and Methods in the main text. Files showing full alignments of the genes are available from KHJG or PDE on request.

| Trypsin/ Chymotrypsin | clade* | HaOG2-nos | HaSP-ids | Ha scaffold_ids | *H. armigera* ** | *H. zea*** | *B. mori* ** |
| --- | --- | --- | --- | --- | --- | --- | --- |
| T | 1 | HaOG200444-71, HaOG200395 | HarmTryp101-29 | scaffold_306/ scaffold_5027/ scaffold_194 | 29 (1) | 24 (1) | 7 |
| T | 2 | HaOG200364 | HarmTryp010 | scaffold_132 | 1 (1) | 1 (1) | 1 (1) |
| T | 3 | HaOG200355, HaOG200359-61 | HarmTryp001, HarmTryp005-7 | scaffold_0/  scaffold_113 | 4 | 4 | 1 |
| T | 4 | HaOG200392, HaOG200394 | HarmTryp038, HarmTryp040 | scaffold_41/  scaffold_45 | 2 | 2 | 1 |
| T | 5 | HaOG200376-81 | HarmTryp022-7 | scaffold_212 | 6 (5) | 6 (5) | 5 (4) |
| T | 6 | HaOG200426 | HarmTryp072 | scaffold_9 | 1 | 1 | 1 |
| T | 7 | HaOG200437 | HarmTryp083 | scaffold_90 | 1 (1) | 1 (1) | 1 (1) |
| T | 8 | HaOG200427, HaOG200429-34 | HarmTryp073, HarmTryp075-80 | scaffold_9 | 7 (7) | 7 (7) | 0 |
| C | 1 | HaOG200479-504 | HarmChym101-26 | scaffold_13 | 26 (1) | 23 (1) | 3 (1) |
| C | 2 | HaOG200515 | HarmChym018 | scaffold_218 | 1 | 1 | 2 |
| C | 3 | HaOG200475-8 | HarmChym004-7 | scaffold_13 | 4 (1) | 4 (1) | 1 (1) |
| C | 4 | HaOG200473, HaOG200505-8 | HarmChym002, HarmChym008-11 | scaffold_113/ scaffold_13 | 5 | 4 | 2 |
| C | 5 | HaOG200522 | HarmChym023 | scaffold_393 | 1 | 1 | 5 |
| C | 6 | HaOG200509 | HarmChym012 | scaffold_13 | 1 (1) | 1 (1) | 1 (1) |
| C | 7 | HaOG200510-1 | HarmChym013-4 | scaffold_137 | 2 | 2 | 2 |
| C | 8 | HaOG200512-3 | HarmChym015-6 | scaffold_137 | 2 | 2 | 2 |
| C | 9 | HaOG200516 | HarmChym019 | scaffold_24 | 1 (1) | 1 (1) | 1 |
| C | 10 | HaOG214207 | HarmChym025 | scaffold_59 | 1 | 1 | 1 |
| C | 11 | HaOG200517-9 | HarmChym020-2 | scaffold_247 | 3 | 3 | 6 |
| C | 12 | HaOG200524 | HarmChym024 | scaffold_446 | 1 | 0 | 1 |
| C | 13 | HaOG200472 | HarmChym001 | scaffold_101 | 1 | 1 | 1 |

**Figure S18.** **Phylogeny for the digestive serine proteases of *H. armigera*, *H. zea* and *B. mori.*** [Phylogenetic methods are described in the Material and Methods in the main text.](ftp://ftp.csiro.au/Helicoverpa/SI_Figure_6A1.pdf)


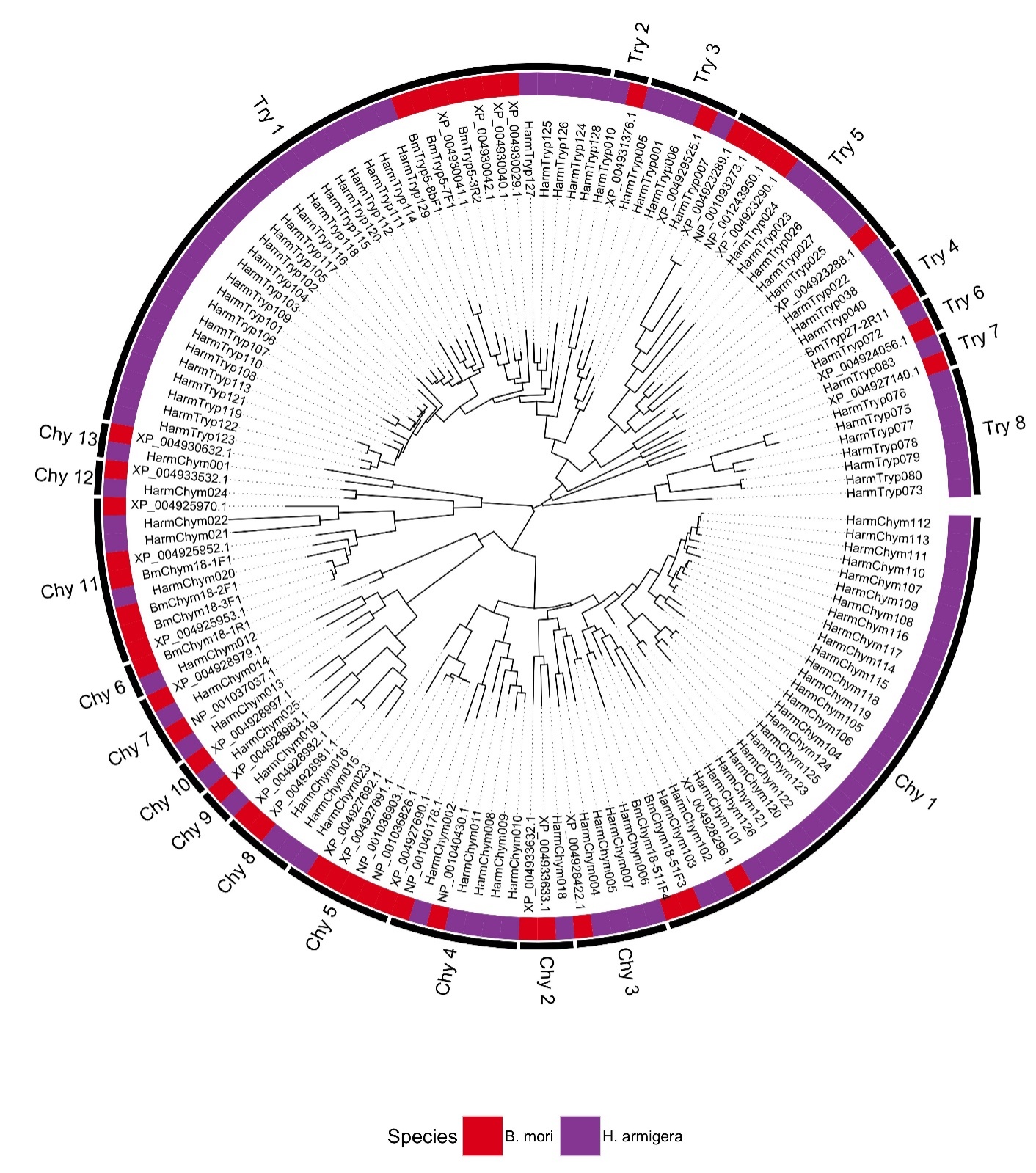


**Figure S19.** **Transcriptome profiles for the *H. armigera* digestive serine proteases.**

**
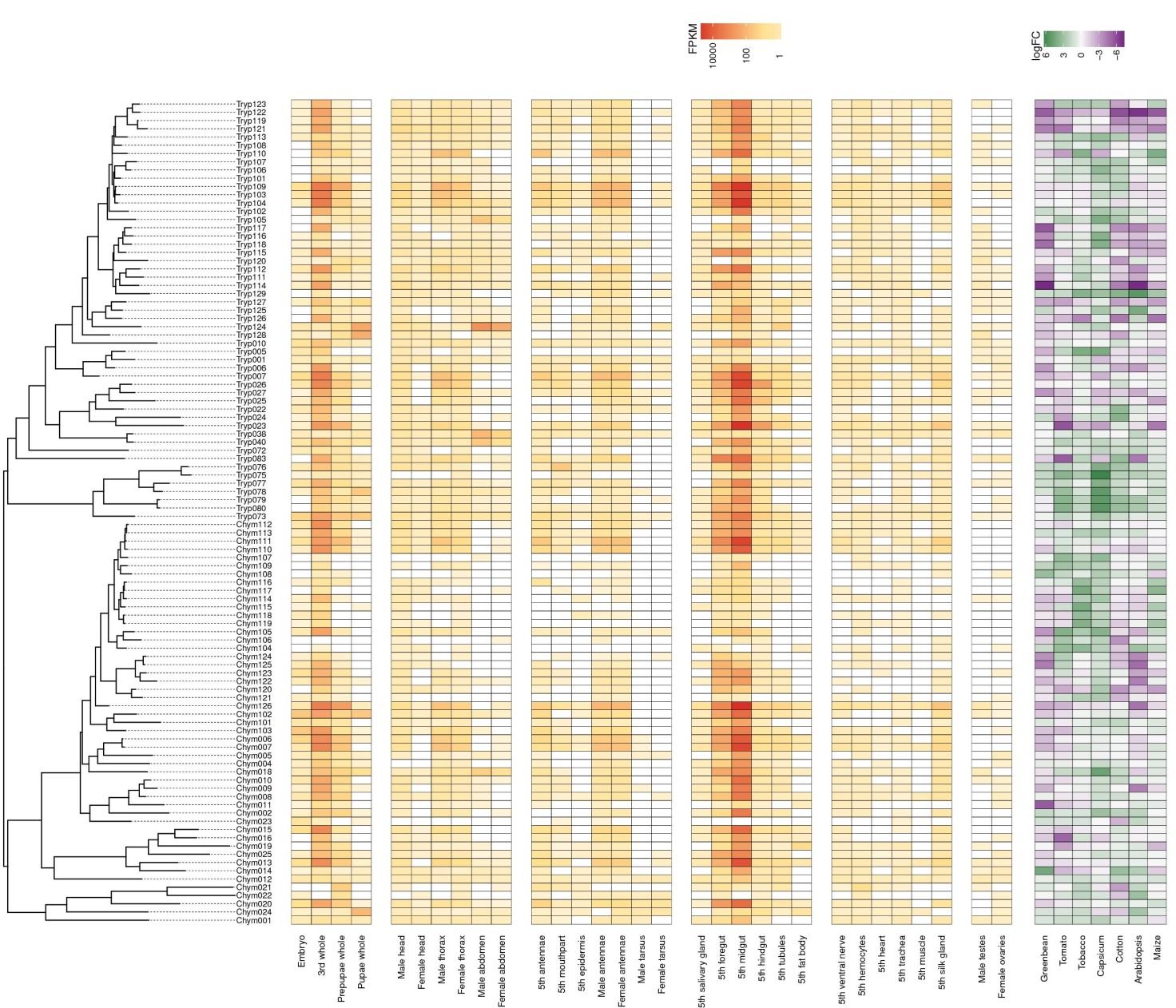
**

**References**

Bown DP, Wilkinson HS, Gatehouse JA (1997) Differentially regulated inhibitor-sensitive and insensitive protease genes from the phytophagous insect pest *Helicoverpa armigera*, are members of complex multigene families. Insect Biochem. Molec. Biol. 27, 625-638.

Bown DP, Wilkinson,HS, Gatehouse JA (2004) Regulation of expression of genes encoding digestive proteases in the gut of a polyphagous lepidopteran larva in response to dietary protease inhibitors. Physiol. Entomol. 29, 278-290.

Broadway RM (1995) Are insects resistant to plant proteinase inhibitors? J. Insect Physiol. 41, 107-116.

Broadway RM (1996) Dietary proteinase inhibitors alter complement of midgut proteases. Arch. Insect Biochem. Physiol. 32, 39-53.

Kuwar SS, Pauchet Y, Vogel H, Heckel DG (2015) Adaptive regulation of digestive serine proteases in the larval midgut of *Helicoverpa armigera* in response to a plant protease inhibitor. Insect Biochem. Molec. Biol. 59, 19-29.

Patankar AG, Giri AP, Harsulkar AM, Sainani MN, Deshpande VV, Ranjekar PK, Gupta VS (2001) Complexities in specificities and expression of *Helicoverpa armigera* gut proteinases explains polyphagous nature of the insect pest. Insect Biochem. Molec. Biol. 31, 453-464.

Ross J, Jiang H, Kanost MR,Wang Y (2003) Serine proteases and their homologs in the *Drosophila melanogaster* genome: an initial analysis of sequence conservation and phylogenetic relationships. Gene 304, 117-131.

Srinivasan A, Giri AP, Gupta VS (2006) Structural and functional diversities in lepidopteran serine proteases. Cell. Mol. Biol. Lett. 11, 132-154.

Zou Z, Lopez DL, Kanost MR, Evans JD, Jiang H (2006) Comparative analysis of serine protease-related genes in the honey bee genome: possible involvement in embryonic development and innate immunity. Insect Molec. Biol. 15, 603-614.

**Section 7. Detailed analysis of lipases in *H armigera*, *H. zea* and *B. mori***

The *H. armigera* acid and neutral lipase genes were identified as described in Materials and Methods in the main text, with additional sequence and phylogenetic analyses as per those previously undertaken for other invertebrate lipases, including those of the lepidopterans *Epiphyas postvittana* and *B. mori* (Christeller et al. 2010)*. H. zea* lipases were identified by homology with the *H. armigera* sequences. Annotation of the *H armigera*, *H. zea* and *B. mori* lipases followed the methods outlined in the main text. Table S17 summarises the results for the three species and Table S18 below gives more detail for each of the *H. armigera* lipases identified, including identities of key motifs. Additional file 6: Table S5 gives the gene models and genome locations for all the lipase genes in both the heliothines.

**Acid lipases**

Twenty eight acid lipase genes were identified in the *H. armigera* genome. *H. zea*, also with 28, has orthologs of each of the *H. armigera* genes. Analysis of the *B. mori* gene set predicted by the NCBI Gnomon pipeline, complemented by further analysis as described in Materials and Methods in the main text, showed *B. mori* only has 21, lacking orthologs of the heliothine genes in each of the three major acid lipase groups (see below). Typically the heliothine genes encode proteins of 450-550 amino acids, as per previously identified insect acid lipases. Because they all appear to have large active site lid loops their likely substrates are triacylglycerols and they are expressed most heavily within the midgut (group 2) to digest such lipids in the diet and elsewhere in the body (groups 1 and 3) to utilise these lipids from stored reserves.

The catalytic function of these lipases is performed by a typical hydrolase triad of serine, aspartate and histidine. The active site serine can be found at position 438. This motif (motif 1) has a consensus sequence of GxSQG(T/M)T. The active site aspartate is at position 706, just upstream of a consensus leucine at position 708 (motif 2). The active site histidine is at position 747 in most sequences, near a consensus aspartate at position 749 (motif 3). A fourth diagnostic motif (motif 4) is a cysteine loop at the N-terminal end of the active site lid sequence. The two cysteines are at positions 552 and 562 and support a loop of between five and nine amino acids.

The sequences cluster into three groups on several criteria, although group 2 is actually split in a phylogeny based on coding sequence only (Table S18, Fig. S20). Group 1, comprising four genes, encodes proteins which all have GHSMG at motif 1, are secreted, and have a cysteine loop containing six to nine amino acids. The genes possess a single exon that includes the sequence coding for the signal peptide. Group 2, comprising ten genes and encodes proteins which are all secretes and have a cysteine loop containing nine amino acids. The genes possess five to eight exons. Group 3 comprises fourteen genes. At least four and possibly six of the corresponding proteins lack an identifiable signal peptide and appear to be intracellular enzymes. All the proteins in this group have a cysteine loop containing five to nine amino acids. Each of the genes possesses a single exon.

Only one gene in Group 1, HarmLip058, produces a protein that is definitely inactive, with alanine replacing serine in motif 1. HarmLip046 and its very similar paralog, HarmLip045, have motif 4 displaced 10 amino acids towards the C-terminal. The rest of the genes appear able to produce proteins with active, consensus-fulfilling proteins across all motifs.

The inactive protein, HarmLip058, can be identified as a storage protein, possibly lipid binding, which is likely to be the major egg yolk protein (MEYP). Lepidoptera are unusual among insects in that their MEYP is derived from an acid lipase and not from a neutral lipase. Inspection of transcriptomes from a wide range of Lepidoptera has never identified more than one MEYP per species (JTC unpublished data). While most of these would be rendered inactive by non-consensus residues at one or more active site positions, this is not essential for MEYP function since *E. postvittana* has all three active site residues intact (JTC unpublished data).

Notably there is a high level of heterogeneity between enzymes, both within and among groups, in their expression on different diets (Fig. S21). Overall however there is a tendency for expression on cotton to be relatively low and that on arabidopsis to be relatively high, compared to the expression level on laboratory diet. These high level features are also seen with the neutral lipases below, and their implications are discussed more fully in connection with the neutral lipases below.

**Table S17.** **Summary of numbers of acid and neutral lipase genes in *H. armigera*, *H. zea* and *B. mori.*** All genes were identified as described in Materials and Methods in the main text. Files showing full alignments of the three species genomes are available from KHJG or JTC on request.

| Type | Group | *H. armigera* | *H. zea* | *B. mori* |
| --- | --- | --- | --- | --- |
| Acid | 1 | 4 | 4 | 2 |
|  | 2 | 10 | 10 | 7 |
|  | 3 | 14 | 14 | 12 |
| Neutral | 1 | 9 | 9 | 2 |
|  | 2 | 9 | 9 | 2 |
|  | 3 | 9 | 9 | 2 |
|  | 4 | 2 | 1 | 1 |
|  | 5 | 5 | 5 | 4 |
|  | 6 | 1 | 1 | 1 |
|  | 7 | 2 | 2 | 1 |
|  | 8a | 2 | 2 | 2 |
|  | 8b | 4 | 4 | 4 |
|  | 9 | 4 | 4 | 2 |
|  | 10 | 4 | 4 | 2 |
|  | 11a | 2 | 2 | 2 |
|  | 11b | 3 | 3 | 3 |
|  | 12 | 5 | 5 | 4 |

**Figure S20**. **Phylogeny of acid lipases from *H. armigera* and *B. mori* .** Phylogenetic methods are as described in Materials and Methods in the main text. *B. mori* sequences are from the whole genome Gnomon annotation available at GenBank under the accession numbers shown.


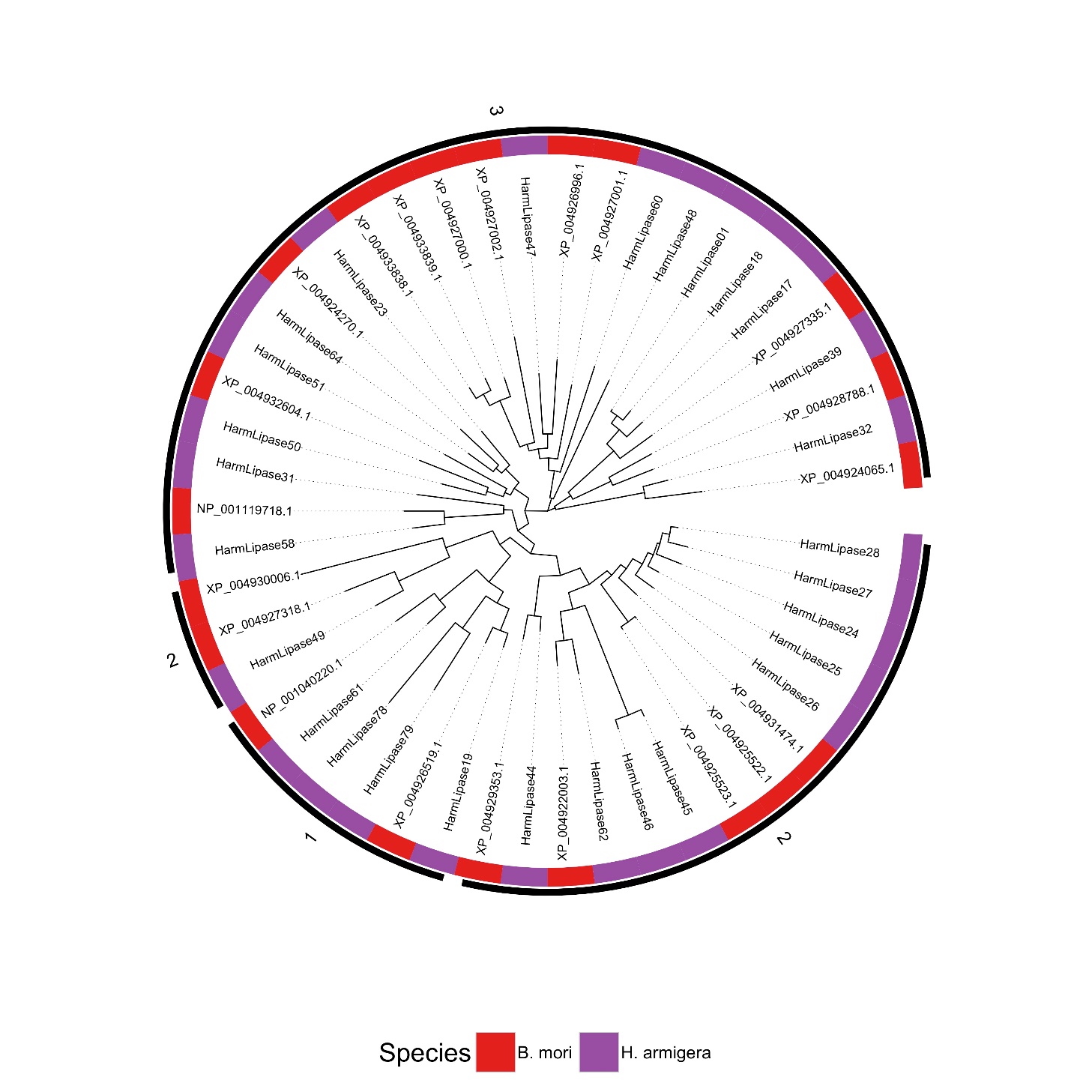


**Figure S21. Transcriptome profiles for the *H. armigera* acid lipases.**


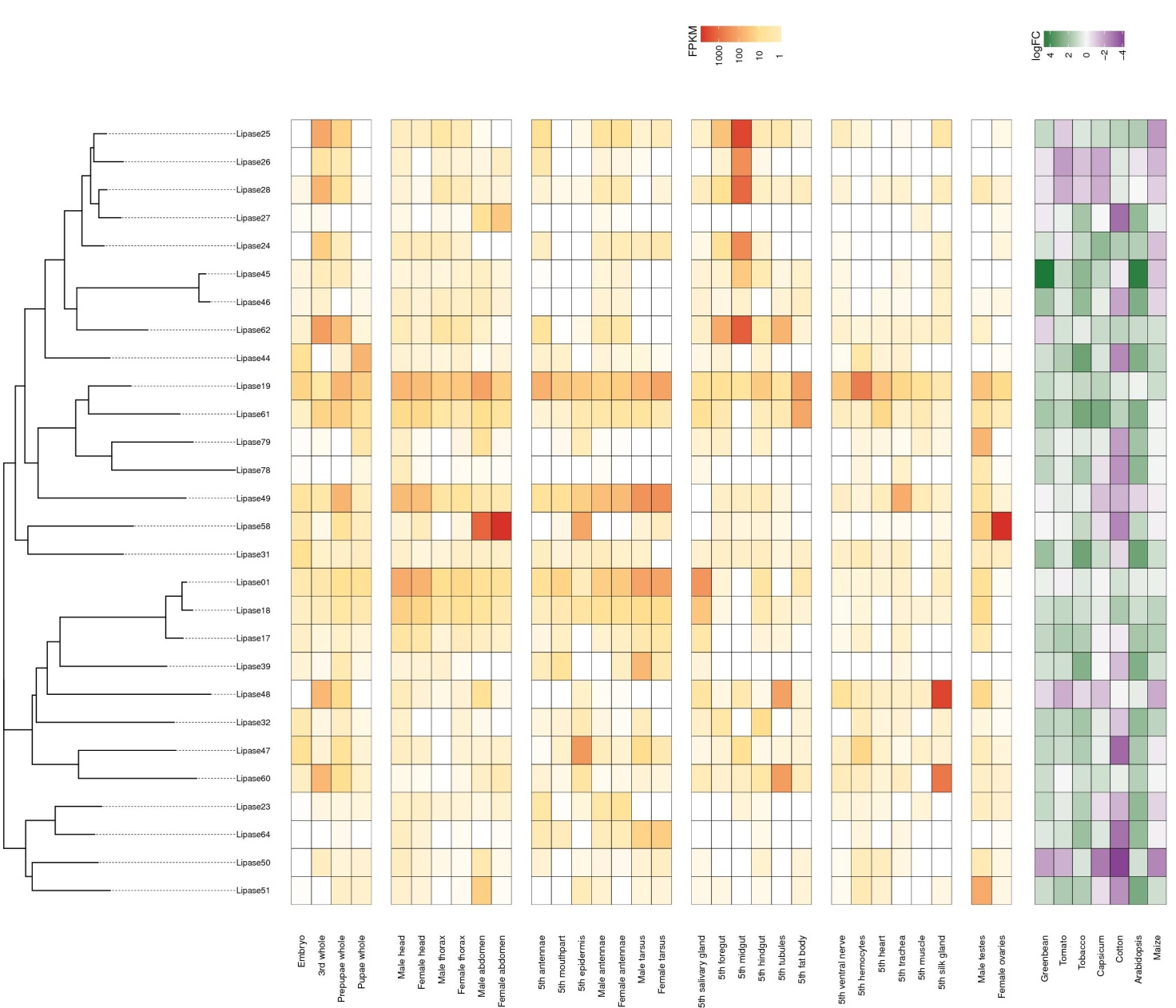


**Neutral lipases**

The neutral lipases can be recognised by consensus motifs surrounding the three catalytic triad residues, firstly the motif GxS(L/V)Gx(A/G/S,H/Q) containing the catalytic serine, secondly (A/G)L(D/E)PA containing the catalytic asparate, and thirdly (M/Q)PGCzzzzCxH containing the catalytic histidine, where zzzz is the variable lid loop. The A at the end of the second motif appears to be essential for steric reasons, at least in classical pancreatic lipase (Roussel et al. 1998), so enzymes having T or S in place of the A are classified as inactive whereas those with G are not. Also, replacing the catalytic D with E in this motif does not cause inactivity (Lowe 1996). Enzymes with only partial serine motifs have also been called inactive, as have enzymes with G in place of the S. An enzyme with G in place of the catalytic H in the third motif is also classified as inactive. As with the acid lipases, studies have also noted a role for the β5 loop within the lid sequences in determining substrate specificity (Carrière et al. 1998; Xiao and Lowe 2015).

*H. armigera* has 61 neutral lipases, and *H. zea* has 60, lacking just one ortholog of the *H. armigera* complement (the catalytically inactive group 4 enzyme HarmLip42; see below), whereas *B. mori* has just 32 (Tables S17, S18). Many of the *B. mori* genes have direct orthologs in the heliothine gene sets. The genes in the three species can be classified into 12 major Groups based on the phylogeny shown in Fig. S22 and the differences between *B. mori* and the heliothines are mainly explained by major expansions in the latter in Groups 1, 2 and 3 (Table S17). Notably, notwithstanding the orthologies found between these three species, levels of orthology between their neutral lipases and those so far recovered from the more distantly related light brown apple moth, *E. postvittana*, are low (data not shown), and the intron/exon structure of the genes (see below) and the clade structure of the phylogeny in *E. postvittana* group are quite different (Simpson et al. 2007; Christeller et al. 2010).

About ten of the neutral lipases in *H. armigera* lack key residues required for activity as defined above. These proteins are scattered across several of the Groups, although, interestingly, both the Group 4 enzymes are classified as inactive.

*H. armigera* has some neutral lipases with large lidded loops. In particular the enzymes in Group 8b have lid sizes of 21-26 amino acids. These lids are similarly-sized to those of well studied triacylglycerol hydrolases such as human pancreatic lipase. Moreover these sequences have full-sized β5 loops, supporting their identity as triacylglycerol hydrolases.

Most members of three other Groups, 7, 9 and 12, have lids of 13-16 amino acids and deletions of about five amino acids in their β5 loops. The substrate specificities of these proteins with intermediate lid sizes is unknown but at least some may also be triacylglycerol hydrolases.

Many other neutral lipases (in Groups 2, 3, 4, 10 and 11) have lid sizes of only about four amino acids and deletions of about five amino acids in their β5 loops. The catalytically active proteins in these groups are highly likely to be galactolipases and phospholipases (Andersson et al. 1996; Withers-Martinez et al. 1996; Sias et al. 2004; Christeller et al. 2010; Christeller et al. 2011). This may also be true of the enzymes in Groups 5, 7 and 8a, with lids of 7-10 amino acids and five amino acid deletions in β5. Notably one Group (1) also has a lid of only four residues but in this case only a single amino acid deletion in theβ5 loop. The largely undeleted loops in this latter Group raise a flag with respect to substrate specificity.

SignalP 4.1 predicts most but not all of the neutral lipases to be secreted. The few that are not, which are scattered across several of the Groups, may be involved in sub-cellular functions such as remobilization of triglycerides within the fat body. The enzymes in Groups 2 and 3 are mainly expressed in the larval guts so are likely heavily involved in digestive functions. It is notable in this respect that these two groups contribute heavily to the greater number of neutral lipases in the heliothines than *B. mori*. Most of the others are more widely expressed. Notably also, several of the inactive enzymes are well expressed, either in the midgut or more widely, which is consistent with the view that at least some of these proteins retain some non-catalytic function.

Interestingly the Group 2 but not Group 3 neutral lipases found in the larval gut are overexpressed in most of the host diets tested relative to the laboratory diet (Fig. S23). However, there is also considerably variation among the enzymes in various of the Groups, whether or not they are heavily expressed in the gut, in their responses to different host diets. As with the acid lipases, overall, many of the neutral lipases are underexpressed on cotton and overexpressed in arabidopsis relative to their expression on laboratory diet.

Dietary factors that might impact on lipase gene expression include the presence or absence of lipase inhibitors (eg lipoxygenase, β-amylase and triterpene acids; (Satouchi et al. 1998, 2002, Christeller et al. 2014), the levels of different lipids (free fatty acids, triglycerides, phospholipids and galactolipids; Christeller et al. 2010, 2011), and the effect of diet on surfactant production (possibly LCFA-glutamate/glutamine, JTC unpublished). The protein inhibitors are more likely in seed-based diets whereas the chemical lipase inhibitors are present in epidermal tissues, particularly fruit. Triglycerides are typically high in seeds and galactolipids in photosynthetic tissue. Phospholipids and free fatty acids are ubiquitous but the latter are generally at high levels in artificial diets, which likely explains the relatively low level of lipases found in larvae feeding on laboratory diet.

**Figure S22**. **Phylogeny of neutral lipases from *H. armigera* and *B. mori*.** Phylogenetic methods are as described in Materials and Methods in the main text. *B. mori* sequences are from the whole genome Gnomon annotation available at GenBank under the accession numbers shown.


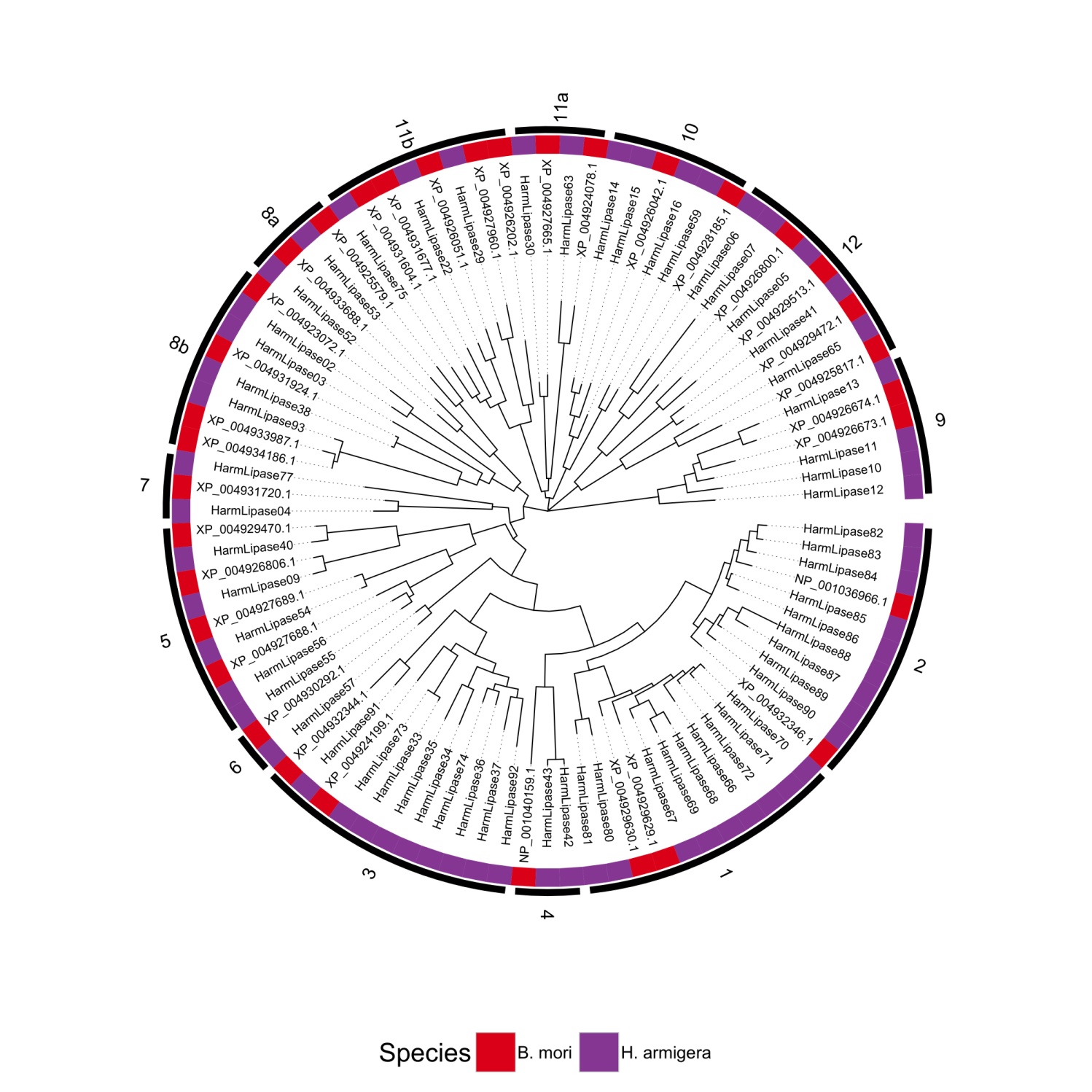


**Figure S23. Transcriptome profiles for the neutral lipases.**


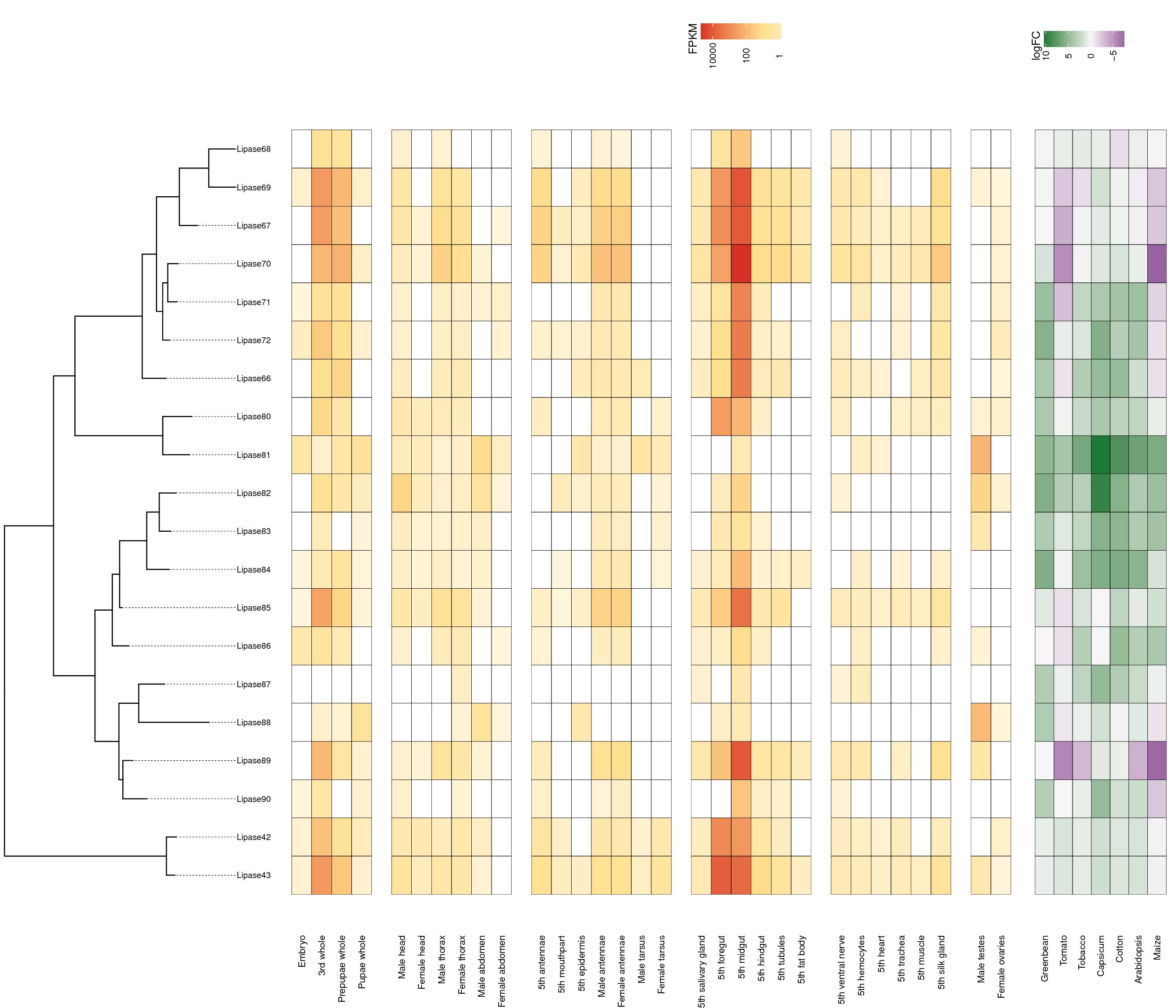


**Table S18.** **Sequence characteristics of the *H. armigera* lipases.** See text for descriptions of diagnostic sequence motifs.

| HarmLip # | OGS# | Group | Active/ Inactive | Catalytic motif M1 (S) | Catalytic motif M2 (D) | Catalytic motif M3 (H) | Cysteine loop motif M4 (lid) | M4 size (#AAs between Cs) | Motif M4 (#AAs deleted in β5) | Signal Peptide (# AAs) |
| --- | --- | --- | --- | --- | --- | --- | --- | --- | --- | --- |
| Acid lipases |  |  |  |  |  |  |  |  |  |  |
| 19 | 200546 | 1 | A | GHSMGTT | DWL | HID |  | 7 |  | Y (49) |
| 61 | 200588 | 1 | A | GHSMGTT | DLL | HLD |  | 9 |  | Y (21) |
| 78 | 200605 | 1 | A | GHSMGAT | DWL | HFD |  | 6 |  | Y (23) |
| 79 | 200606 | 1 | A | GHSMGAT | DWL | THE |  | 6 |  | Y (20) |
| 24 | 200551 | 2 | A | GHSQGTT | DPF | HVD |  | 9 |  | Y (20) |
| 25 | 200552 | 2 | A | GHSQGTT | DPF | HLD |  | 9 |  | Y (19) |
| 26 | 200553 | 2 | A | GHSQGTT | DPF | HLD |  | 9 |  | Y (17) |
| 27 | 200554 | 2 | A | GHSQGTT | DPF | HID |  | 9 |  | Y (19) |
| 28 | 200555 | 2 | A | GHSQGTT | DPF | HID |  | 9 |  | Y (21) |
| 44 | 200571 | 2 | A | GFSQGTT | DWL | HLD |  | 9 |  | Y (23) |
| 45 | 200572 | 2 | A | GHSQGGT | DDL | HSD |  | 9 |  | Y (17) |
| 46 | 200573 | 2 | A | GASQGGT | DDL | HTD |  | 9 |  | Y (17) |
| 49 | 200576 | 2 | A | GYSMGTT | DQL | HLD |  | 9 |  | Y (19) |
| 62 | 200589 | 2 | A | GHSQGGT | DKE | HYD |  | 9 |  | Y (17) |
| 01 | 200528 | 3 | A | GFSQGTG | DGL | HMD |  | 9 |  | N |
| 17 | 200544 | 3 | A | GFSQGAG | DGY | HLD |  | 9 |  | N |
| 18 | 200545 | 3 | A | GFSQGTG | DGL | HLD |  | 9 |  | N |
| 23 | 200550 | 3 | A | GHSQGTT | DWL | HLD |  | 9 |  | Y(17) |
| 31 | 200558 | 3 | A? | GHSQGTT | DWx | ? |  | 6 |  | N |
| 32 | 200559 | 3 | A | GFSQGTA | DWF | HLE |  | 9 |  | N |
| 39 | 200566 | 3 | A | GHSQGTT | DLL | HHN |  | 6 |  | Y (21) |
| 47 | 200574 | 3 | A | GFSEGTT | DKI | HID |  | 8 |  | Y (16) |
| 48 | 200575 | 3 | A | AHSQGVS | DYV | HLD |  | 9 |  | Y(18) |
| 50 | 200577 | 3 | A | GHSQGST | DWL | HID |  | 9 |  | Y(26) |
| 51 | 200578 | 3 | A | GYSQGTT | DWF | HVD |  | 7 |  | N |
| 58 | 200585 | 3 | I | GHAQGTT | DWL | HMD |  | 5 |  | Y(18) |
| 60 | 200587 | 3 | A | GHSEGTS | DQI | HTD |  | 9 |  | Y (19) |
| 64 | 200591 | 3 | A | GFSQGTT | DWL | HFD |  | 9 |  | Y(20) |
| Neutral lipases |  |  |  |  |  |  |  |  |  |  |
| 66 | 200593 | 1 | A | GFSLGAH | ALDPA | CNH | QPGCLTNFC | 4 | 1 | Y (16) |
| 67 | 200594 | 1 | A | GFSLGAH | GLDPA | CNH | QPGCLTNVC | 4 | 1 | Y (18) |
| 68 | 200595 | 1 | A | GLSLGAH | GLDPA | CSH | QPGCQTHVC | 4 | 1 | N |
| 69 | 200596 | 1 | A | GLSLGAH | GLDPA | CNH | QPGCLTNVC | 4 | 1 | Y (18) |
| 70 | 200597 | 1 | A | GFSLGAH | ALDPA | CNH | QPGCLTNIC | 4 | 1 | Y (18) |
| 71 | 200598 | 1 | A | GFSLGAH | ALDPA | CNH | QPGCLTNLC | 4 | 1 | N |
| 72 | 200599 | 1 | A | GFSLGAH | ALDPA | CNH | QPGCLTNLC | 4 | 1 | N |
| 80 | 200607 | 1 | A | GFSLGGH | ALDPA | CSH | MPGCYIPTC | 4 | x | Y (19) |
| 81 | 200608 | 1 | A | GFSLGGH | ALDPA | CSH | MPGCWLNSC | 4 | 5 | Y (20) |
| 82 | 200609 | 2 | A | GFSLGAH | GLDPA | CSH | QPGCGVNHDC | 5 | 5 | N |
| 83 | 200610 | 2 | A | GFSLGAH | GLDPA | CSH | QPGCSVNHDC | 5 | x | Y (18) |
| 84 | 200611 | 2 | A | GFSLGAH | GLDPA | CSH | QPGCSSVDYNC | 6 | 5 | Y (18) |
| 85 | 200612 | 2 | A | GFSLGAH | GLDPA | CSH | QPGCWISTC | 4 | 5 | Y (18) |
| 86 | 200613 | 2 | A | GFSLGAH | GLDPA | CSH | QPGCWISTC | 4 | 5 | Y (16) |
| 87 | 200614 | 2 | A | GFSLGAH | GLDPA | CSH | QPGCWTQATSC | 6 | 5 | Y (16) |
| 88 | 200615 | 2 | A | GFSLGAH | GLDPA | CSH | QPGCWLRPSSC | 6 | 5 | Y (16) |
| 89 | 200616 | 2 | A | GFSLGAH | GLDPA | CSH | QPGCWISTC | 4 | 5 | Y (18) |
| 90 | 200617 | 2 | A | GFSLGAH | GLDPA | CSH | QPGCWISTC | 4 | 5 | N |
| 33 | 200560 | 3 | A | GHSLGGH | SLDPA | CSH | MPGCGDNDC | 4 | 5 | Y (18) |
| 34 | 200561 | 3 | A | GYSVGAH | AMDPA | CDH | MPGCSTALC | 4 | 5 | Y (18) |
| 35 | 200562 | 3 | A | GYSVGGH | GCDPA | CHH | MPGCMTSLC | 4 | 5 | N |
| 36 | 200563 | 3 | A | GHSLGAH | ALEAA | CDH | MPGCNSQEC | 4 | 5 | Y (17) |
| 37 | 200564 | 3 | I | GHGLGAH | ALDAA | CDH | MPGCISQEC | 4 | 5 | Y(15)) |
| 73 | 200600 | 3 | ? | GHSLGGx | x | x | x | x | x | Y (20) |
| 74 | 200601 | 3 | A | GHSLGGH | GLDPA | CDH | MPGCSTQEC | 4 | 6 | Y(16) |
| 91 | 200618 | 3 | I | GVGLGGH | AIDPS | CSH | QNSCAGDVTC | 5 | 5 | Y (19) |
| 92 | 200619 | 3 | I | GHGFGGH | ALDPA | CDH | MPGCNSHAC | 4 | 5 | Y (18) |
| 42 | 200569 | 4 | I | GFGLGAH | GLDPS | CSH | QPGCLTNTC | 4 | 2 | N |
| 43 | 200570 | 4 | I | GFGLGAH | GLDPS | CSH | QPGCLTNTC | 4 | 2 | Y (18) |
| 09 | 200536 | 5 | A | GHSLGAH | GLDPA | CSH | QPYCEDTPNEALC | 8 | 5 | Y (24) |
| 40 | 200567 | 5 | A? | GHSLGAH | GLDPG | CSH | QPGCKGHVMRIARC | 9 | 5 | Y (25) |
| 54 | 200581 | 5 | I | GYSVGAH | GLDPT | CDH | QPGCAHDTIFQTLSC | 10 | 5 | Y (23) |
| 55 | 200582 | 5 | A | GFSLGGQ | GLDPA | CNH | QPGCKATKNQTKSGC | 10 | 5 | Y (21) |
| 56 | 200583 | 5 | A | GFSLGGQ | GLDPA | CNH | QPGCKDTDEKSKSSC | 10 | 5 | Y (22) |
| 57 | 200584 | 6 | A | GHSLGAH | GLDPA | CAH | QPGCGYDIRGLC | 7 | 5 | Y (14) |
| 04 | 200531 | 7 | A | GFSLGAE | GLDPA | CSH | QPGCWVDELIKNREFRFVYGC | 16 | 5 | Y (30) |
| 77 | 200604 | 7 | I | GHSLGAQ | GLDPA | CDG | QPGCELEVVLPQQLLLNKC | 14 | 5 | N |
| 52 | 200579 | 8a | A | GHSLGSH | GLDPA | CSH | QPGCDGAQQVIEGC | 9 | 5 | Y (19) |
| 53 | 200580 | 8a | A | GHSLGAH | GLDPA | CSH | QPGCDNIFKIFEAC | 9 | 5 | N |
| 02 | 200529 | 8b | A | GHSLGAH | GLDPA | CSH | Q+H3:H89P+H3:H87GCFNGTSSWLSLVPYAIRLQQAIC | 21 | 0 | Y (24) |
| 03 | 200530 | 8b | I | GASMPNY | GLDPA | CNH | QHGCSNLFVGAVSDFVLPWAAASPEGRSLC | 25 | 0 | Y (21) |
| 38 | 200565 | 8b | A | GHSLGAH | GLDPA | CNH | QPGCDLTEGPLVPLTLVKQGLEEASRVLVAC | 26 | 0 | Y (27) |
| 93 | 200620 | 8b | A | GHSLGAH | GLDPA | CNH | PGCKQASSPNGGSNDLSYQQVVKYVGC | 23 | 0 | N |
| 10 | 200537 | 9 | A | GHSLGSQ | ALDPA | CSH | QPKCTVSWLPPLLNTLLEPRC | 16 | 5 | Y (18) |
| 11 | 200538 | 9 | A | GHSLGGQ | ALDPA | CNH | QPNCGTVLVPGILDSSLSAKC | 16 | 5 | Y (18) |
| 12 | 200539 | 9 | I? | AMSLGGQ | SLDPA | CSH | QPACDEETDVFRC | 8 | 5 | Y (20) |
| 13 | 200540 | 9 | A | GHSLGSQ | ALDPA | CSH | QPSCHEGYIPGKGESDAAKC | 15 | 5 | Y (19) |
| 14 | 200541 | 10 | A | GHSLGSH | GLDPA | CSH | QPNCLLQTC | 4 | 5 | Y (18) |
| 15 | 200542 | 10 | A | GHSLGSH | GLDPA | CSH | QPNCLFQTC | 4 | 5 | Y (18) |
| 16 | 200543 | 10 | A | GHSLGSH | GLDPA | CSH | QPNCSPTDFSC | 6 | 5 | Y (23) |
| 59 | 200586 | 10 | A | GHSLGSH | ALDPA | CDH | QPGCYISVC | 4 | 5 | N |
| 30 | 200557 | 11a | A | GISLGAH | GLDPA | CSH | QPGCFMPSC | 4 | 5 | Y (22) |
| 63 | 200590 | 11a | A | GFSLGAH | ALDPA | CDH | QPECRDRSISLEC | 8 | 5 | Y (21) |
| 22 | 200549 | 11b | I | GFSLGCH | ALEPS | CSH | QPSDISLYPCTTTC | 3 | 5 | Y (21) |
| 29 | 200556 | 11b | A | GGSLGAH | GLDPA | CSH | IMPCFQLC | 3 | 5 | Y (15) |
| 75 | 200602 | 11b | I | GLSLGAH | GLDPS | CSH | QPGDFFWVPCEVIC | 3 | 5 | Y (23) |
| 05 | 200532 | 12 | A | GHSLGAQ | GLDPA | CNH | QPGCDNKPHPRFSLEDLC | 13 | 5 | Y (17) |
| 06 | 200533 | 12 | A | AHSLGAH | GLDPA | CSH | QPGCPVGDFEMFTKESLC | 13 | 5 | Y (24) |
| 07 | 200534 | 12 | A | GHSLGSH | GLDPA | CSH | QNACVGSTDPMC | 7 | 5 | Y (21) |
| 41 | 200568 | 12 | A | GHSLGSH | ALDPA | CSH | QPGCPRFAPIPLSDDNLC | 13 | 5 | N |
| 65 | 200592 | 12 | A | GHSLGSH | GLDPA | CSH | QPGCLSATVPLTIEDFC | 12 | 5 | Y (18) |

*All HarmLipases have identically numbered *H. zea* orthologs except HaOG200569.

**References**

Andersson L, Carriére F, Lowe ME, Nilsson A, Verger R (1996). Pancreatic lipase-related protein 2 but not classical pancreatic lipase hydrolyzes galactolipids. Biochim. Biophys. Acta 1302, 236-240.

# Carriér, F, Withers-Marinez C, van Tilbeurgh H, Roussel A, Cambillau C, Verger R (1998). Structural basis for the substrate selectivity of pancreatic lipases and some related proteins. Biochim. Biophys. Acta 1376, 417-432.

Christeller JT, Amara S, Carrière F (2011). [Galactolipase, phospholipase and triacylglycerol lipase activities in the midgut of six species of lepidopteran larvae feeding on different lipid diets](https://scholar.google.co.nz/citations?view_op=view_citation&hl=en&user=8eCDIh8AAAAJ&sortby=pubdate&citation_for_view=8eCDIh8AAAAJ:u_35RYKgDlwC) J. Insect Physiol. 57, 1232-1239.

Christeller JT, McGhie TK, Poulton J, Markwick NP (2014). [Triterpene acids from apple peel inhibit lepidopteran larval midgut lipases and larval growth](https://scholar.google.co.nz/citations?view_op=view_citation&hl=en&user=8eCDIh8AAAAJ&sortby=pubdate&citation_for_view=8eCDIh8AAAAJ:D_sINldO8mEC). Arch.Insect Biochem. Physiol. 86, 137-150.

# Christeller JT, Poulton J, Markwick NM, Simpson RM (2010). The effect of diet on the expression of lipase genes in the midgut of the lightbrown apple moth (Epiphyas postvittana Walker; Tortricidae). Insect Molec. Biol. 19, 9-25.

Lowe ME (1996). Mutation of the catalytic site Asp177 to Glu177 in human pancreatic lipase produces an active lipase with increased sensitivity to proteases. Biochim. Biophys. Acta 1302, 177-183.

Roussel A, de Caro J, Bezzine S, Gastinel L, de Caro A, Carrière F, Leydier S, Verger R, Cambillau C (1998). Reactivation of the totally inactive pancreatic lipase RP1 by structure-predicted point mutations. [Proteins](https://www.ncbi.nlm.nih.gov/pubmed/9726421) 32, 523-31.

Satouchi, K., Hirano, K., Fujino, O., Ikoma, M., Tanaka, T., Kitamura, K. (1998) Lipoxygenase-1 from soybean seed inhibiting the activity of pancreatic lipase. Biosci Biotechnol Biochem 62: 1498-1503.

Satouchi, K., Kodama, Y., Murakami, K., Tanaka, T., Iwamoto, H., Ishimoto, M. (2002) A lipase-inhibiting protein from lipoxygenase-deficient soybean seeds. Biosci Biotechnol Biochem 66: 2154-2160.

Sias B, Ferrato F, Grandval P, Lafont D, Boullanger P, de Caro A, Leboeuf B, Verger R, Carrière F (2004). Human pancreatic lipase-related protein 2 is a galactolipase. [Biochemistry.](https://www.ncbi.nlm.nih.gov/pubmed/15287741) 43, 10138-10148.

Simpson RM, Newcomb RD, Gatehouse HS, Crowhurst RN, Chagne D, Gatehouse LN et al. (2007). Expressed sequence tags from the midgut of Epiphyas postvittana (Walker) (Lepidoptera: Tortricidae). Insect Molec. Biol. 16, 675-690.

Withers-Martinez C, Carrière F, Verger R, Bourgeois D, Cambillau C (1996). A pancreatic lipase with a phospholipase A1 activity: crystal structure of a chimeric pancreatic lipase-related protein 2 from guinea pig. [Structure](https://www.ncbi.nlm.nih.gov/pubmed/8939760) 4, 1363-1374.

Xiao X, Lowe ME (2015). [The β5-loop and lid domain contribute to the substrate specificity of pancreatic lipase-related protein 2 (PNLIPRP2).](https://www.ncbi.nlm.nih.gov/pubmed/26494624) J. Biol. Chem. 290, 28847-28856.

**Section 8. Detailed analysis of GR genes in *H. armigera* and *H. zea***

The 213 GR genes which have been identified in the *H. armigera* genome are heavily clustered, with just over half (117) being found on just five scaffolds (Additional file 6: Table S5). The largest cluster, of 53 genes, is found at one end of scaffold_139, with a further 10 on scaffold_88, which is adjacent to scaffold_139 at the beginning of chromosome 20, based on *Bombyx* synteny (our unpublished work). Both these scaffolds also contain other smaller clusters, of 4 and 14 GR genes respectively. Other GR gene clusters of 11 genes each are found on scaffold_395 and scaffold_69. The genes in these clusters have been assigned to the bitter taste receptor family (Xu et al. 2016).

A significant proportion of the *H. armigera* GR genes cannot be found in any of the available *H. zea* genome data. Several lines of evidence suggest that this is mainly due to gene loss in *H. zea* rather than gain of new genes in *H. armigera* post divergence of the two species. The phylogenetic evidence in Fig. S24 is that most of the *H. armigera*/*H. zea* divergences are more recent than duplications generating *H. armigera* paralogs. There are a few cases where both *H. armigera* paralogs lack orthologs in *H. zea*, eg the duplications in the largest GR cluster on scaffold_139 giving rise to HarmGR133/HarmGR134 (HaOG200712/HaOG200718) and HarmGR135/HarmGR154 (HaOG200721/HaOG200696), but the depths of the nodes leading to these branches are greater than for any of the divergences between *H. armigera* and H*. zea* orthologs. There are also some extended tips with *H. armigera* genes but these refer to sequences that are incomplete or not fully predicted*.* These gene losses particularly affect the gene clusters described above, with those on scaffold_139 and scaffold_88 missing a total of 13 genes in *H. zea*. The clusters on scaffold_395 and scaffold_69 have each lost 7 genes in the *H. zea* genome assembly Additional file 6: Table S5).

A subset (11) of the *H. armigera* GRs lacking *H. zea* orthologs, together with paralogs (from both species), were then aligned as nucleotide coding sequences and a phylogram constructed using Starbeast (Fig. S25). This tree clearly shows all the *H. armigera*/*H. zea* divergences covered in this analysis to be more recent than the duplications generating *H. armigera* paralogs. The genes analysed were derived from clusters on six different scaffolds, including the two major clusters on scaffold_139 and scaffold_88. In a separate analysis using the mutation rate for *H. melpomene* (see Materials and Methods), the divergence (shown as node age 0.1851) between the branches leading to HarmGR33/HarmGR40 (HaOG200884/6, on scaffold_395) and HarmGR18/HarmGR15 (HaOG200898/9, on scaffold_4) was dated to 3.3 Mya, ie comparable to the ages found for the other major gene family expansions described in Table S6. This also suggests that subsequent duplications in the *H. armigera* GR gene lineages are among the more recent expansions found in this species, but detailed analysis of these awaits a finalised gene model set for this large and diverse family of challenging small genes.

There is also other evidence that the GRs as a family are rapidly diverging between the two species. Thus, on average, the Ka/Ks statistics for pairs of orthologs in the two species indicate rates of amino acid sequence divergence for GRs that are almost twice as high as those for any other of the other manually curated families. As shown in Table S19, among the major families of chemo-responsive proteins, genes with significantly high Ka/Ks (over >~0.3), i.e. that are evolving faster than most other genes, are to be found among GRs in particular. The larger clusters of GR genes are particularly rich in genes with higher Ka/Ks scores (Additional file 6: Table S5). In contrast very few genes for OBPs (see also Vogt et al. 2015) and ORs show evidence of such rapid evolution, and the other families none at all.

Another intriguing observation is that 12 of the *H. zea* GRs have internal stop codons. These 12 GRs represent 60% of the 20 *H. zea* genes with such internal stop codons among the 2269 genes allocated to gene families. Some of the 20 gene models showing stop codons are likely to be incorrect due to assembly errors that thus affect a very low overall number of gene models, but the high proportion of GRs in this set suggests most of these are correctly described and may therefore be pseudogenes, ie likely to be in the process of being lost.

As a family, the GRs showed considerable changes in gene expression in larvae raised on different plant hosts (Additional file 3: Fig. S26). There was no evidence for clades or sub-groups showing differing expression profiles. Strong increases in expression of almost all the GRs were observed for larvae reared on tobacco and Arabidopsis, compared to that observed for larvae reared on lab standard diet, while overall GR expression decreased when reared on cotton; milder increases were found on greenbean with limited changes on other hosts especially maize, which gave the most uniform pattern. The observed GR expression changes do not correlate obviously with the other measurements of larvae growth on these hosts (see Fig. 5) – e.g. although larvae reared on cotton had the fastest development found in this experiment, they were not the smallest; poorest larval growth was on Arabidopsis and on capsicum, which had different effects on GR expression.

Recent work on the genome-wide transcriptomic response of insect GRs to different host diets (Simon et al. 2015; Eyres et al. 2016), is beginning to increase our understanding of their role in facilitating aversive taste responses to unpalatable compounds (Lunceford and Kubaraek 2015). Overall, it appears that the *H. armigera* GRs are detecting host-specific chemistries which do not immediately correlate with either host toxicity or nutritional value. It is further interesting that the host eliciting the slightest changes in GR expression is maize, given the possible significance of its wild relatives for *H. zea* after its invasion of the Americas (and consistent with the loss of GRs in this species, as discussion in the main text).

**Figure S24. Phylogenetic tree of the *H. armigera* and *H. zea* GRs.** Sequences (amino acid) of the predicted GRs of these two species were aligned using Mafft and the phylogram constructed using RaxML (Stamatakis 2014) (see Materials and Methods).


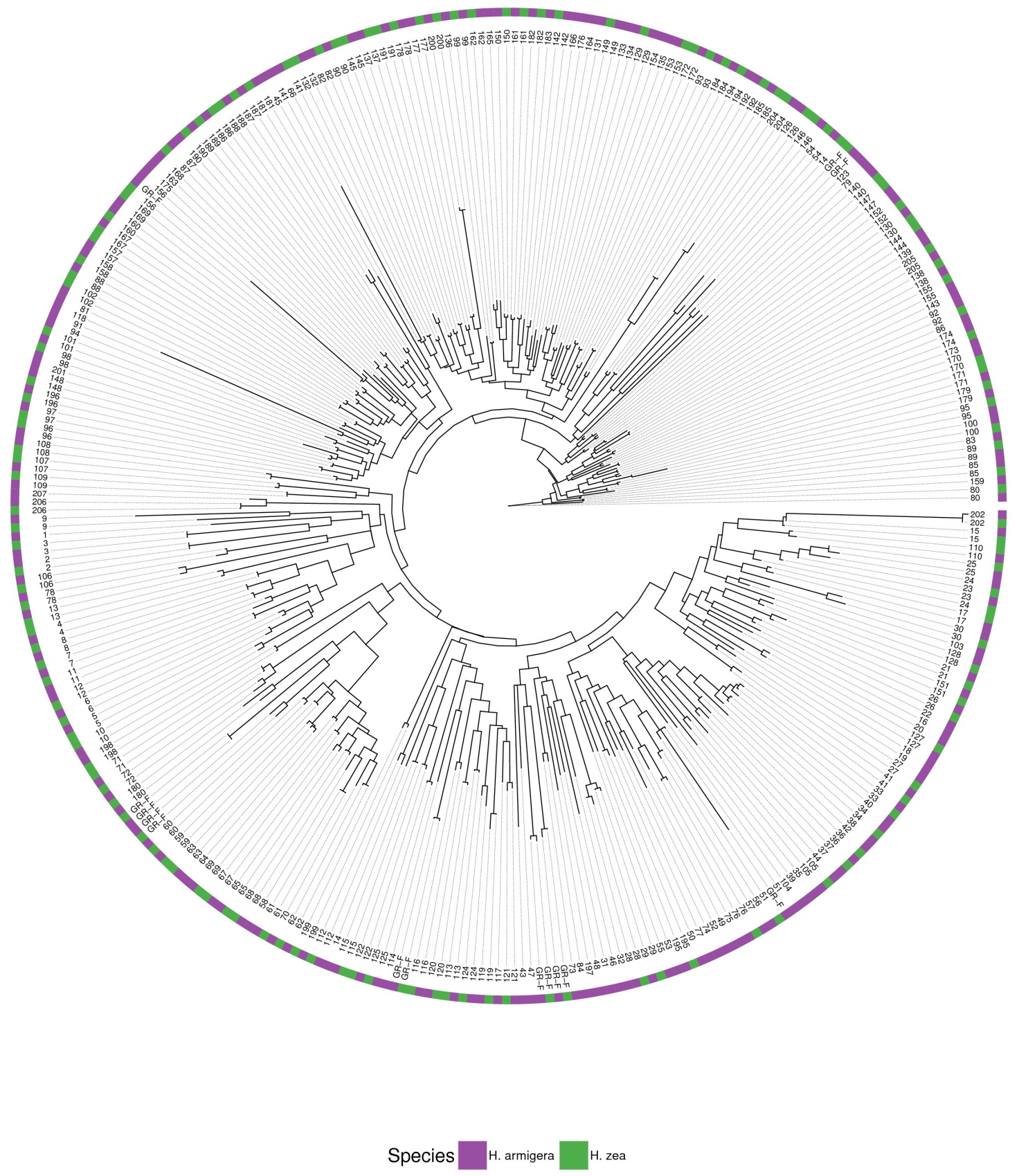


**Figure S25. Phylogenetic tree of selected *H. armigera* and *H. zea* GRs.** As explained in the text, a subset (11 in total) of the *H. armigera* GRs lacking *H. zea* orthologs, together with paralogs (from both species), were selected to compare the interspecific divergence ages with those for the paralogs within *H. armigera*. Nucleotide sequences coding for these predicted GRs of these two species were aligned using the protein alignment derived for Fig. S24 as a guide and the phylogram constructed using Starbeast as described in the Materials and Methods. Numbers indicate node ages from the tips, as calculated by Starbeast.


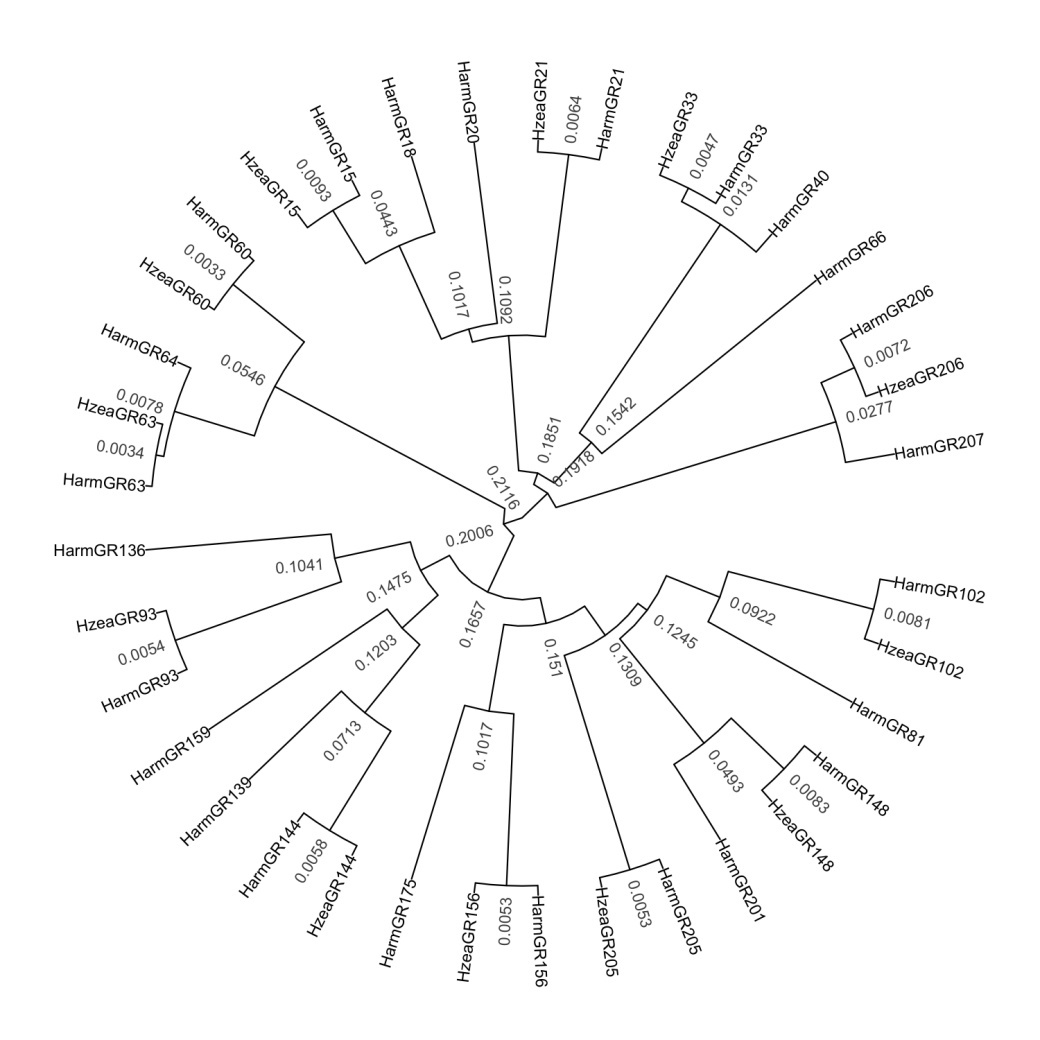


**Table S19. Summary of chemo-response protein families in *H. armigera* and *H. zea* and Ka/Ks distributions.** Gene numbers and Ka/Ks value counts are compiled from the gene list in Additional file 6: Table S5, based on sub-family allocations under the heading “sub-fam”.

| family | Total: *H. armigera* | Total: *H. zea* | Ka/Ks <0.2 | 0.2< Ka/Ks <0.3 | 0.3< Ka/Ks <0.5 | Ka/Ks >0.5 |
| --- | --- | --- | --- | --- | --- | --- |
| GR | 213 | 166 | 49 | 37 | 52 | 18 |
| OR | 84 | 82 | 69 | 2 | 3 | 3 |
| IR | 40 | 40 | 36 | 3 | 0 | 0 |
| OBP | 40 | 40 | 38 | 0 | 2 | 0 |
| CSP | 29 | 29 | 28 | 1 | 0 | 0 |
| PBP | 4 | 4 | 3 | 1 | 0 | 0 |

**References**

Eyres I, Jaquiéry J, Sugio A, Duvaux L, Gharbi K, Zhou J-J, Legeai F, et al. (2016) Differential gene expression according to race and host plant in the pea aphid. Molec. Ecol. 25, 4197–4215.

Lunceford BE, Kubanek J (2015). Reception of aversive taste. Integr.Comp. Biol.55: 507-15.

Simon JC, d’Alençon E, Guy E, Jacquin-Joly E, Jaquiéry J, Nouhaud P, Peccoud J, et al. (2015). Genomics of adaptation to host-plants in herbivorous insects. Briefings Funct. Genomics 14:413-423.

Stamatakis A. (2014). RAxML Version 8: A tool for phylogenetic analysis and post-analysis of large phylogenies. Bioinformatics, 30:1312-3.

Vogt RG, Grosse-Wilde E, Zhou JJ (2015) The Lepidoptera Odorant Binding Protein gene family: gene gain and loss within the GOBP/PBP complex of moths and butterflies. Insect Biochem. Molec. Biol. 62:142-153.

Xu W, Papanicolaou A, Zhang H-J, Anderson A. (2016). Expansion of a bitter taste receptor family in a polyphagous insect herbivore. Scientific Reports 6: 23666.

**Section 9. Detailed analysis of stress response and immunity genes in *H. armigera* and *H. zea***

Stress response genes.

A total of 37 genes for heat shock proteins (HSPs) have been identified in the *H. armigera* genome and named according to size and homology, as listed in Additional file 6: Table S5. All had orthologs in the *H. zea* genome, with all having low Ka/Ks values, under 0.02. Fifteen of the 37 genes in *H. armigera* were found to be DE on different hosts, with most of these being upregulated on Capsicum and to a lesser degree on cotton (Fig. S27). The most heavily upregulated on Capsicum were HarmHSP23.5-214 (HaOG214788), HarmHSP19.9-180 (HaOG207164) and the adjacent paralogous genes HarmHSP20.8-188 and HarmHSP20.4-180.

A number of other stress-related genes showed varying degrees of downregulation on cotton (Fig. S28), with the catalases being most affected.

Immunity-related genes and gene families

We have identified nearly 300 immunity-related genes (including a number of serine proteases) in the *H. armigera* genome. A complete listing is provided in Additional file 6: Table S5 under [biological-role] = “immunity”. They are classified as recognition, modulation, signalling and effector molecules, and harbor the probable orthologs for nearly all members of the Toll, IMD, JAK/STAT and JNK immune signalling pathways (Table S20). It appears that these pathways are conserved throughout different insect orders. The identified total number is greater than that of *D. melanogaster* (248) and *A. gambiae* (278), as well as those found in *B. mori* (174) and *A. mellifera* (117) (Tanaka et al. 2008; Waterhouse et al. 2007). Compared with the *B. mori* genome, most family members containing Toll, fibrinogen-related protein and others, especially the intracellular components, possess precise 1:1 orthology (Tanaka et al. 2008). The Toll-like receptor gene family encodes single-pass transmembrane proteins with the extracellular leucine rich repeats (LRR) domain and an intracellular Toll-interleukin 1 receptor (TIR) domain, and acts as a key player in the Toll pathway (Hetru and Hoffmann 2009). There are 14 members in the toll receptor family in the *H. armigera* and *B. mori* genomes and the counts are the most in comparison with other insect genomes to date. Members of the fibrinogen-related protein (FREP) family are also evolutionarily conserved and extend from invertebrates to mammals (Wang et al. 2005). FREPs exhibit species-specific expansions in *A. gambiae*, which harbors as many as 61 members, compared to *A. mellifera* with 37 and *D. melanogaster* with 14 FREPs. Here we have identified only 3 members in the *H. armigera* genome, which is remarkably lower than those found in the *A. gambiae* and *D. melanogaster* genome. Moreover, two gene families (Eater and IAP2) show a high frequency of lineage-specific expansions at the expense of 1:1 orthology in comparison with other insects.

Very few immunity-related genes were identified as DE on the diets, with the largest group being seven of the serine proteases.

**Figure S27. Transcriptome profiles of the HSPs on plant hosts.** Please see Additional file 6: Table S5 for annotated names.

**
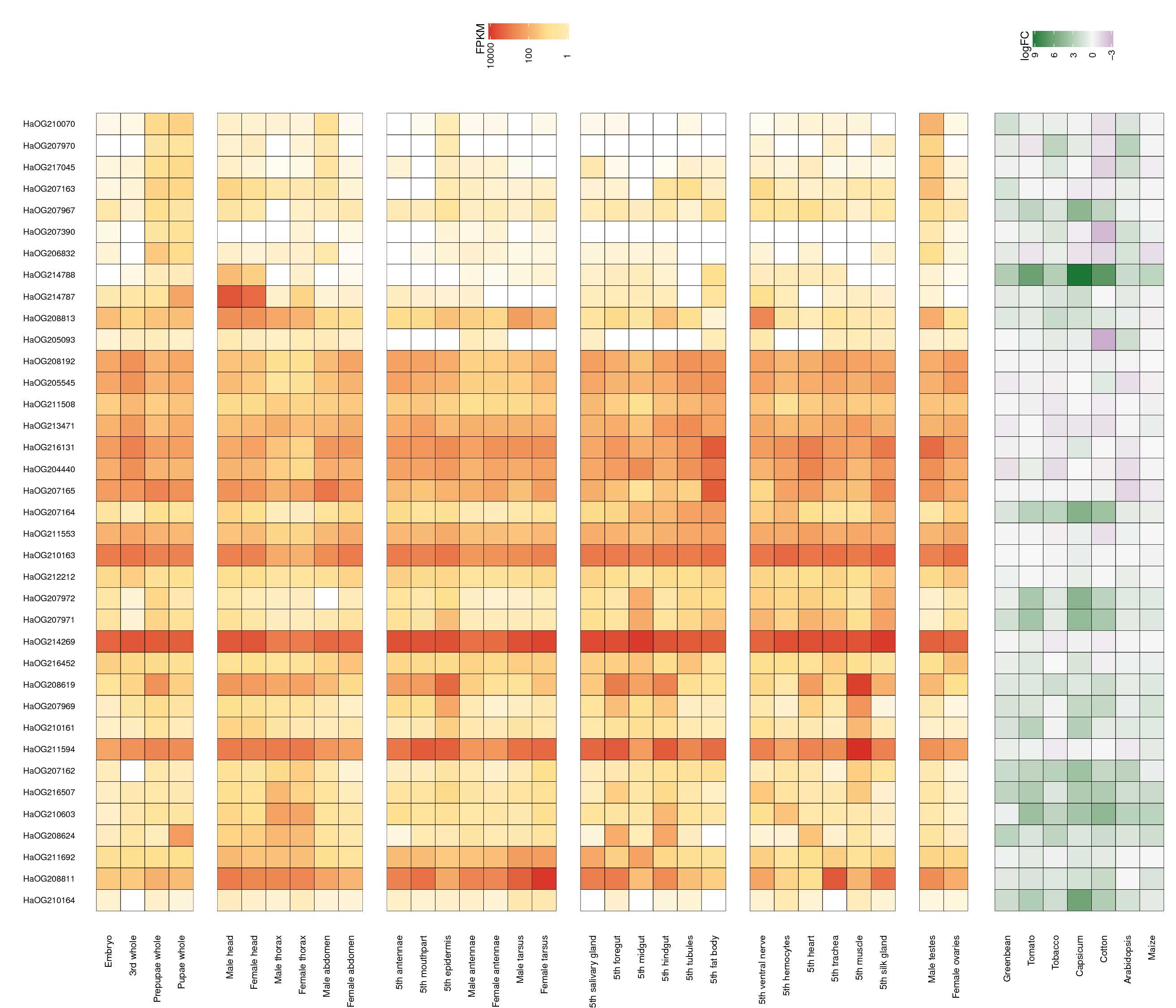
**

**Figure S28. Transcriptome profiles of other stress-response related genes including catalases and peroxidases.** The genes were ordered based on heirarchical clustering of their normalised tissue expression patterns. Please see Additional file 6: Table S5 for annotated names.


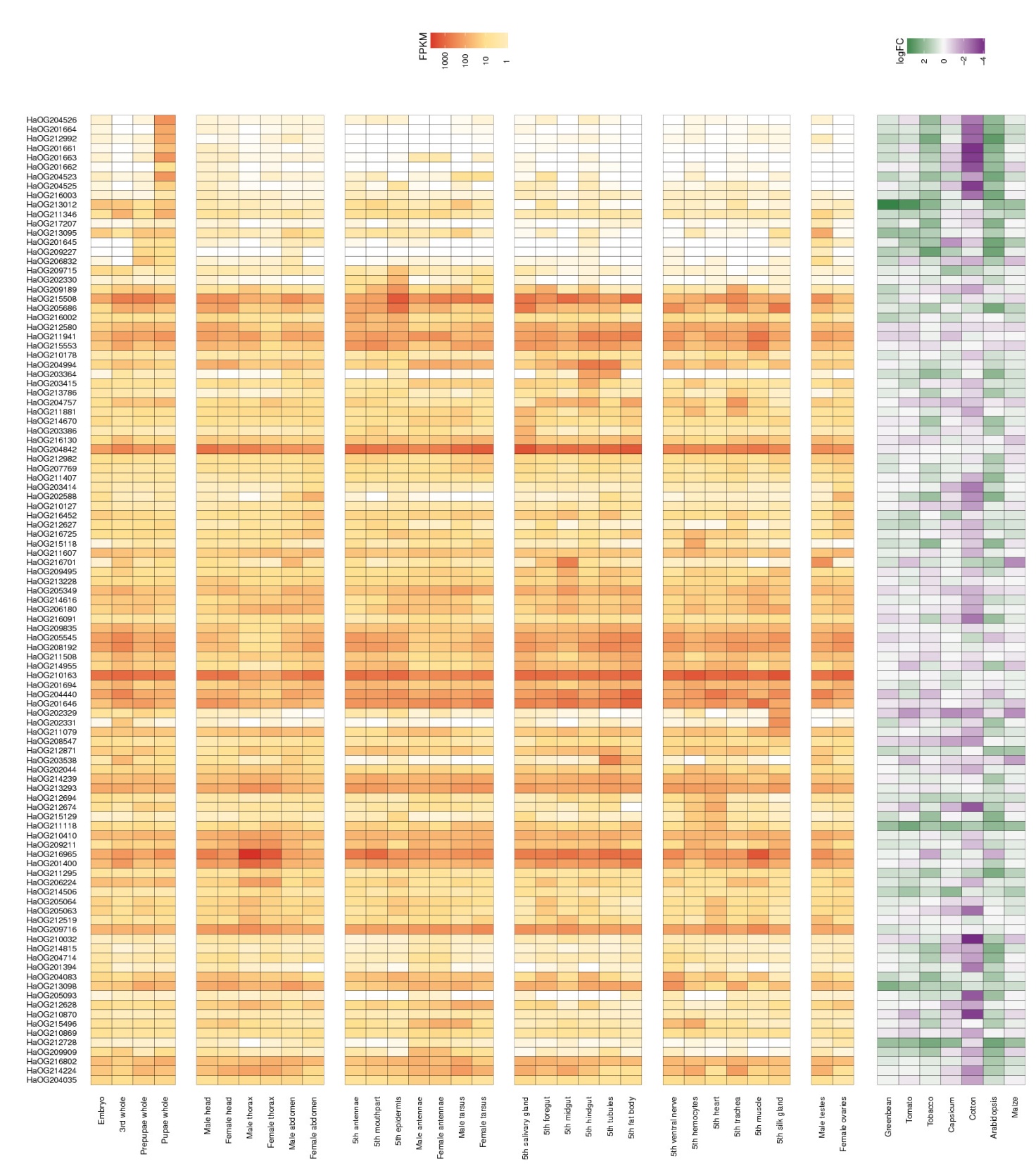


**Table S20.** **Immunity related genes in *H. armigera* and other insect genomes.**

| Gene family | *H. armigera* | *B. mori* | *D. melanogaster* | *A. gambiae* | *A. mellifera* |
| --- | --- | --- | --- | --- | --- |
| **Recognition** |  |  |  |  |  |
| PGRP | 9 | 12 | 13 | 7 | 4 |
| β-GRP/GNBP | 7 | 4 | 3 | 7 | 2 |
| C-type lectin | 41 | 21 | 34 | 25 | 10 |
| Galectin | 4 | 4 | 6 | 8 | 2 |
| Fibrinogen-related protein | 3 | 3 | 14 | 61 | 2 |
| TEP | 3 | 3 | 6 | 15 | 3 |
| Scavenger receptor B | 16 | 13 | 12 | 15 | 10 |
| Eater | 4 | 0 | 1 | 1 | 0 |
| Dscam | 1 | 1 | 1 | 1 | 1 |
| Drapter | 1 | 1 | 1 | 1 | 1 |
| **Modulation** |  |  |  |  |  |
| CLIP serine protease/homolog | 21 | 15 | 37 | 41 | 18 |
| Serpin | 26 | 16 | 30 | 17 | 5 |
| **Signalling** |  |  |  |  |  |
| Toll pathway |  |  |  |  |  |
| Spätzle | 8 | 3 | 6 | 6 | 2 |
| Toll | 14 | 14 | 9 | 10 | 5 |
| MyD88 | 1 | 1 | 1 | 1 | 1 |
| Tollip | 2 | 2 | 1 | 2 | 1 |
| Tube | 1 | 1 | 1 | 1 | 1 |
| Pellino | 1 | 1 | 1 | 1 | 1 |
| Pelle | 1 | 1 | 1 | 1 | 1 |
| TRAF2 | 1 | 1 | 1 | 1 | 1 |
| ECSIT | 1 | 1 | 1 | 1 | 1 |
| Cactus | 1 | 1 | 1 | 1 | 3 |
| Dif/Dorsal | 2 | 1 | 2 | 1 | 2 |
| Imd pathway |  |  |  |  |  |
| IMD | 1 | 1 | 1 | 1 | 1 |
| Dredd | 1 | 1 | 1 | 1 | 1 |
| TAK1 | 1 | 1 | 1 | 1 | 1 |
| FADD | 1 | 1 | 1 | 1 | 1 |
| Tab2 | 1 | 1 | 1 | 1 | 1 |
| IAP2 | 4 | 1 | 1 | 1 | 1 |
| IKKβ | 1 | 1 | 1 | 1 | 1 |
| IKKγ | 1 | 1 | 1 | 1 | 1 |
| Ubc13 | 1 | 1 | 1 | 1 | 1 |
| Relish | 1 | 1 | 1 | 1 | 2 |
| JAK/STAT pathway |  |  |  |  |  |
| Upd3 | 0 | 0 | 1 | 0 | 0 |
| PIAS | 1 | 1 | 1 | 1 | 1 |
| SOCS | 1 | 1 | 1 | 1 | 1 |
| Domeless | 1 | 1 | 1 | 1 | 1 |
| Hopscotch | 1 | 0 | 1 | 1 | 1 |
| STAT | 1 | 1 | 1 | 2 | 1 |
| JNK pathway |  |  |  |  |  |
| Hem | 1 | 1 | 1 | 1 | 1 |
| JNK | 1 | 1 | 1 | 1 | 1 |
| Fos | 1 | 1 | 1 | 1 | 1 |
| Jun | 1 | 1 | 1 | 1 | 1 |
| **Effector** |  |  |  |  |  |
| PPO | 2 | 2 | 3 | 9 | 1 |
| Defensin | 1 | 1 | 1 | 1 | 2 |
| Cecropin | 2 | 13 | 4 | 4 | 0 |
| Attacin | 1 | 2 | 4 | 1 | 0 |
| Gloverin | 2 | 4 | 0 | 0 | 0 |
| Moricin | 1 | 1 | 0 | 0 | 0 |
| Lebocin | 1 | 1 | 0 | 0 | 0 |
| Lysozyme | 6 | 1 | 8 | 4 | 3 |
| Other Amp | 0 | 0 | 11 | 1 | 4 |
| Glutathione Oxidase | 5 | 4 | 2 | 3 | 2 |
| Peroxiredoxin | 6 | 4 | 8 | 5 | 5 |
| Superoxide dismutase | 4 | 3 | 4 | 5 | 3 |
| **Total** | 222 | 174 | 248 | 278 | 117 |

**References**

Hetru C, Hoffmann JA (2009) NF-kappaB in the immune response of Drosophila. Cold Spring Harbor Perspectives Biol. 1: a000232

Tanaka H, Ishibashi J, Fujita K, Nakajima Y, Sagisaka A, Tomimoto K, Suzuki N, et al. (2008) A genome-wide analysis of genes and gene families involved in innate immunity of Bombyx mori. Insect Biochemi. Molec. Biol. 38: 1087-1110

Wang XG, Zhao Q, Christensen BM (2005) Identification and characterization of the fibrinogen-like domain of fibrinogen-related proteins in the mosquito, Anopheles gambiae, and the fruitfly, Drosophila melanogaster, genomes. BMC Genomics 6:114.

Waterhouse RM, Kriventseva EV, Meister S, Xi Z, Alvarez KS, Bartholomay LC, Barillas-Mury C, et al. (2007) Evolutionary dynamics of immune-related genes and pathways in disease-vector mosquitoes. Science 316: 1738-1743.

**Section 10. Detailed analysis of some gene families related to larval growth**

The following sets of genes were studied in relation to diet effects on larval growth and development; a summary of the main categories identified is in Table S21.

1. A total of 156 genes annotated with GO terms (Additional file 2: Table S2) related to growth, including development and morphogenesis, were identified (Additional file 6: Table S5). See Additional file 3: Fig. S29 for transcriptome expression data.

2. Transcription factors (TFs) were identified by PFAM domain analysis (see main Materials and Methods) and confirmed by mapping against the *D. melanogaster* TFs listed in Rhee et al. (2014). This yielded a total of 240 likely or putative identified TFs, only 5 of these not being found in the *H. zea* genome assembly. Of these 240, 129 were identified as homologous to *D. melanogaster* TFs assigned to networks by Rhee et al. (2014); see the complete list in Additional file 10: Table S23. Transcriptome expression data in tissues and on hosts are shown in Additional file 3: Fig. S30.

3. Ribosomal proteins (RPs, which are the predominant group with the GO term biogenesis) were identified for both cytoplasmic (79) and mitochondrial (69) ribosomes. See Additional file 6: Table S5 for a complete list of all RP genes and Additional file 3: Fig. S31 for transcriptome expression data of the cytoplasmic RPs.

4. Cuticular proteins were identified by homology and sequence searches based on the motifs described by Willis (2010); these include the proteins containing the Rebers-Riddiford motif (sub-families RR1 (68 members), and RR2 (102 members), plus one RR3 gene) and several other sub-families (Additional file 6: Table S5).

Few differences were evident among the two heliothines in the numbers of genes falling in these functional groups (Table S21). The *B. mori* gene numbers identified on the basis of orthology in this study were lower for some groups (Table S21); a more comprehensive analysis of these groups would be required to assess the significance of this. One class of genes for cuticular proteins includes members of the largest single cluster of duplicated genes we have identified in the *H. armigera* genome, comprising 60 cuticular protein RR-2 genes located on the adjacent scaffold_85 and scaffold_94. Fifty-eight of these genes are present in *H. zea* on five scaffolds, one of which (scaffold_290) bridges the two *H. armigera* scaffolds. The corresponding cluster in *B. mori*, located on chromosome 22, contains 54 genes.

For most of these gene categories, the larval transcriptomic response specifically associated with cotton differs significantly from that observed on the other hosts. Genes most downregulated overall include those involved in cell development and differentiation, and in gene regulation. As noted in the main text, larvae reared on cotton developed most rapidly in progressing through instars, but failed to grow to more than half the size of larvae reared on the control diet. It has been observed that heliothine larvae (of *Heliothis virescens*) feeding on cotton shed midgut cells at an unusually high rate (Hoover et al. 2000) suggesting that our observations reflect gene expression patterns resulting from this process and the likely subsequent enhanced rate of replacement of midgut cells. Enhanced cell shedding and replacement have also been observed in the midgut of *H. armigera* larvae in response to infection by a midgut-specific RNA virus, the *Helicoverpa armigera* stunt virus (Brooks et al. 2002).

The overall downregulation of TFs on cotton (Additional file 3: Fig. S30) contrasts with the more complex responses seen on most hosts, or the overall lack of change (compared to lab diet) evident in particular on maize. Interestingly a number of TFs are upregulated to similar extents on both tobacco and Arabidopsis. The TFs in the three diet modules D8, D10 and D25 which were significantly enriched for the digestion and detoxification genes, are listed in Table S22. Interestingly although these module are up-regulated overall on the more challenging hosts (see main text), several of these transcription factors showed decreased expression on cotton, in particular HaOG204484 (TF AP-2-beta), which is a DE gene, and to a lesser extent HaOG212901(TF Sox-5-like) and HaOG210037 (the p53 homolog).

Expression of the RPs, which is generally high in all tissues and stages, shows a striking overall increase on cotton, likely indicating active cell enlargement; in contrast, RP expression is overall decreased on Arabidopsis. The other hosts result in mild declines, with (for Caspicum) some moderate increases evident too. Larvae growing on maize show RP expression profiles little changed from those on the standard laboratory diet.

The cuticular proteins are highly enriched in diet module D23, which is most highly expressed on cotton and also upregulated on tomato and maize (Fig. 7). See Additional file 3: Fig. S32 for transcriptome expression data of the cuticular proteins. This upregulation on cotton is likely a consequence of the rapid development seen on cotton, with more frequent moults requiring greater levels of cuticle production.

**Table S21. Genes associated with larval growth.** See Additional file 6: Table S5 for details of all genes in these families and categories; the *B. mori* gene counts are based on orthologs identified among NCBI gene models as given in Additional file 1: Table S1.

| Category | Sub-family or group | Biological role | *H. armigera* genes | *H. zea* genes | *B. mori* genes |
| --- | --- | --- | --- | --- | --- |
| Growth | cellular | Cell growth | 9 | 9 | 9 |
| Growth | Var. development | development | 110 | 108 | 77 |
| Growth | Var. morphogenesis | morphogenesis | 30 | 30 | 23 |
| Growth | Wingless (Wnt) | development | 7 | 7 | 5 |
| Ribosomal-prot | cytoRP |  | 79 | 79 | 76 |
| Ribosomal-prot | mitoRP |  | 69 | 69 | 68 |
| Cuticle proteins | RR1 |  | 68 | 66 | 45 |
| Cuticle proteins | RR2 |  | 102 | 98 | 67 |
| Cuticle proteins | RR3 |  | 1 | 1 | 1 |
| Cuticle proteins | gly-rich |  | 23 | 23 | 9 |
| Cuticle proteins | Other (incl hypothetical) |  | 58 | 57 | 42 |

**TABLE S22. *H. armigera* Transcription factors in key host-response expression modules.**

| HaOGS2-gene | Transcription factor name | Dm-network? | module | DE? |
| --- | --- | --- | --- | --- |
| HaOG204484 | transcription factor AP-2-beta [FBgn0261953] | no | D10 | y |
| HaOG207791 | Ets98B [FBgn0005659] | Network | D10 |  |
| HaOG212901 | transcription factor Sox-5-like | no | D10 |  |
| HaOG211044 | putative transcription factor SOX-15-like | no | D8 |  |
| HaOG216165 | transcription factor E74 | no | D8 |  |
| HaOG216802 | HarmHSP-TFd [FBgn0001222] | Network | D8 |  |
| HaOG210037 | p53 [FBgn0039044] | Network | D25 |  |

**TABLE S23. List of 129 *H. armigera* TFs mapped to *D. melanogaster* TFs in networks.** This table (Additional file 10) is suitable for use with the Rhee et al. (2014) networks in Cytoscape[.](ftp://ftp.csiro.au/Helicoverpa/TF-129-Ha-to-Dm-networks.tab)

**References**

Brooks EM, Gordon KHJ, Dorrian SJ, Hines ER, Hanzlik TN (2002). Infection of its lepidopteran host by the *Helicoverpa armigera* stunt virus (Tetraviridae). *J. Invert. Pathol*. 80: 97-111.

Hoover K, Washburn JO, Volkman LE (2000). Midgut-based resistance of *Heliothis virescens* to baculovirus infection mediated by phytochemicals in cotton. J. Insect Physiol. 46: 999–1007.

Rhee DY, Cho DY, Zhai B, Slattery M, Ma L, Mintseris J, Wong CY, et al. (2014). Transcription factor networks in *Drosophila melanogaster*. Cell Reports 8: 2031–2043.

Willis JH. (2010). Structural cuticular proteins from arthropods: annotation, nomenclature, and sequence characteristics in the genomics era. Insect Biochem. Molec. Biol. 40: 189–204. doi:10.1016/j.ibmb.2010.02.001.

**Section 11. Additional insights from diet transcriptome modules**

Two other diet modules providing some specific additional insights into the genetic basis of host response are D20 and D3 (Fig. S33).

D20, which is upregulated on several hosts (particularly tobacco, Capsicum andcotton), was highly enriched (27 of its 34 members) for DE genes but not significantly enriched for either of the other two candidate gene sets. While it did include two clade1 CCEs, a clade1 trypsin, and a clan 3 P450, it revealed other previously unsuspected sets of genes involved in the responses to different hosts including five immunity-related genes, a cadherin and a GO-development annotated ubiquitin ligase.

D3, like module D32 which has been discussed in the main text, was found to be enriched for genes in the developmental stage modules (predominantly T5 and T3, respectively, see Additional file 8: Table S9). D3 showed enrichment for several types of cuticular proteins, while D32 was enriched for genes involved in the immune response, and in olefin/coumarin/melanin biosynthesis (Additional file 9: Table S10b). Genes in these modules also showed significant expression responses on some hosts (Additional file 8: Table S9), with many in D3 (Fig. S33) being upregulated on all hosts other than Arabidopsis and Capsicum, the largest number being on tomato, while many in D32 (Fig. 7) were down-regulated on all hosts other than cotton, the largest number being on Arabidopsis.

Notwithstanding the cases of coordinated expression of some members of the P450, CCE and midgut protease clusters seen in modules such as D25 and D37, we also note that the major families of detoxification and digestion-related families were distributed across several of the DE modules (and in the case of the detoxification families, also some that were not differentially expressed). Of the 79 CCEs in the dietary/detoxification clades, for example, 51 are distributed across 20 DE modules (data not shown). This concurs with the diversity of expression patterns seen in our earlier analyses of this family (Section 2), is consistent with the fact that many members of this family do not have detoxification functions, and indicates that those that do can do so in a variety of ways (Oakeshott et al. 2005). As the gene models (based on RNA-seq data) in Fig. S34 show, one remarkable example involves three clade CCE011 genes in the DE module 21. These genes are clustered on scaffold_155 and share an initiating exon and therefore promoter. Consistent with this genome organization, they show coordinated expression on every host, being particularly upregulated on cotton. However the fourth CCE gene in this cluster encodes a completely discrete transcript, albeit contained within the long first exon of the two downstream-co-initiating genes, and shows no co-expression with the other three, being not found in any expression module and down-regulated on tobacco.

**Figure S33. Expression profiles for genes identified as DE on the diets and as co-expressed within modules D20 and D3.**  Expression (log_2_FC) profiles for each module are shown on the left. The composition of each module is described in the central panels, showing the total number (N) of genes per module, the number that are DE, the number in the five tissue/developmental stage modules T1-5 (TM) and the number in the major gene family (GF) classes defined by the key below. Major functions enriched in each module are noted on the right of the figure.

**
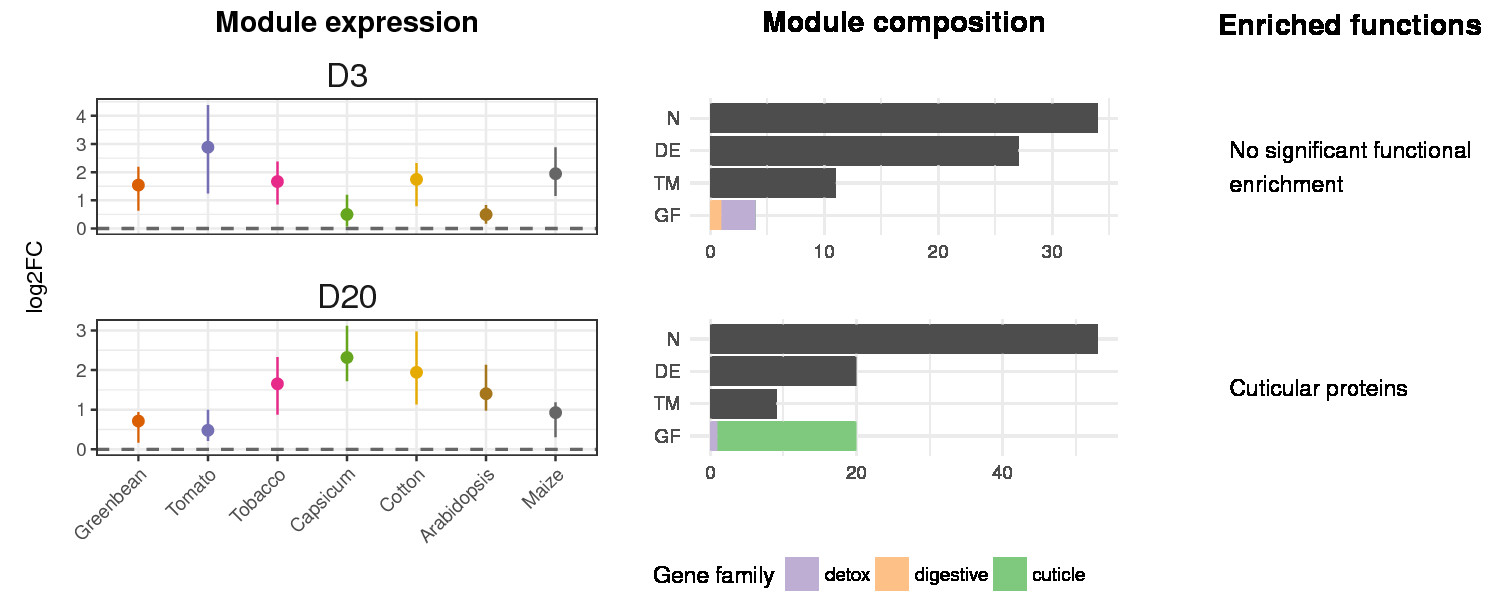
**

**Figure S34. Organisation and expression of the four HarmCCE011 genes on scaffold_155.** (A) Genome organization, showing the three genes that share a promoter (HaOG200178/HarmCCE011d, HaOG200177/HarmCCE011c and HaOG200133/HarmCCE011a) and the fourth, independently expressed, gene, HaOG200134/HarmCCE011b. (B) Expression profiles of the four HarmCCE011 genes across tissues and developmental stages, and on the host plants. The key provided below the figure shows expression levels.

**A.**

**
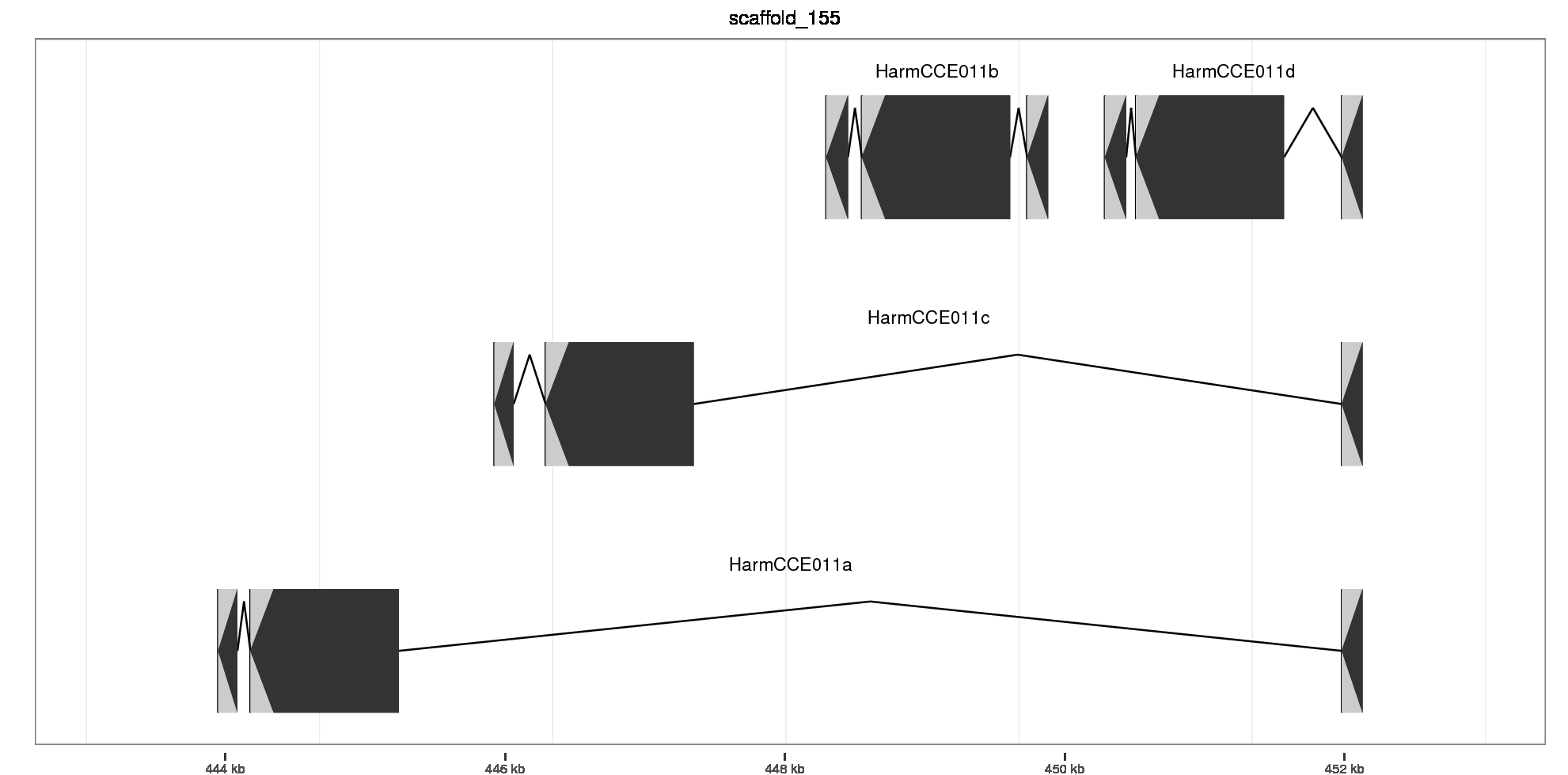
**

**B.**

**
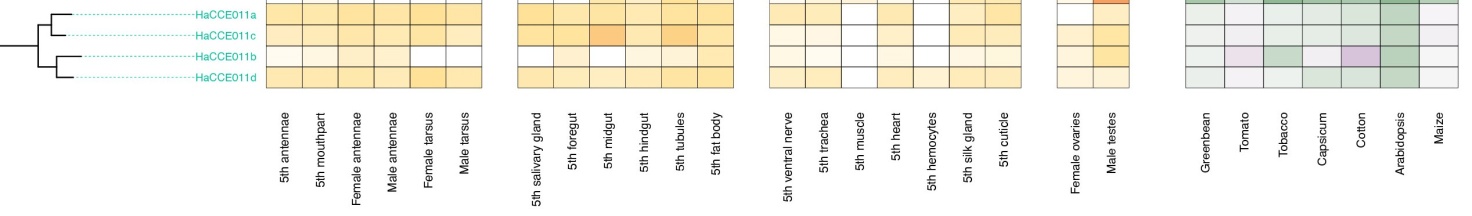
**


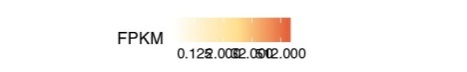


**
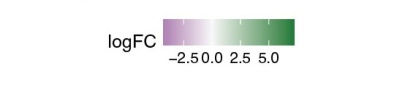
**

**Reference.**

Oakeshott JG, Claudianos C, Campbell PM, Newcomb RD, Russell RJ (2005) Biochemical genetics and genomics of insect esterases. In (Gilbert LI, Iatrou K, Gill SS, eds) Comprehensive Insect Molecular Science Vol 5, Pharmacology. Elsevier pp 309-380.

**Section 12. Detailed analysis of genes related to insecticide resistance in *H. armigera* and *H. zea***

Genes associated with known *H. armigera* resistances

Table S24 details the names and locations of genes directly or potentially implicated in insecticide resistance in *H. armigera* or *H. zea* and Fig. S35 details the expression profiles for those genes in *H. armigera*. Also shown in Table S24 are specific mutations in those genes known to contribute or reasonably suspected of contributing to the respective resistances.

Most of the genes included in these displays are either ones where there is direct evidence for involvement in resistance in *H. armigera* and for which specific resistance conferring mutations have been previously identified for this species (CYP337B3 for metabolic resistance to pyrethroids and cadherin and ABCA2 for resistances to Bt toxins) or ones where bioassay and biochemical data clearly indicate a resistance mechanism for which multiple precedents in other species indicate a prime candidate resistance gene and specific mutations in that gene which confer resistance (eg *para*, *Rdl1* and *Rdl2* and *AChE1* for target site resistances to pyrethroid, cyclodienes and organophosphates/carbamates respectively). The carboxylesterase CCE001g is included because increased esterase activity has been implicated in the resistance in question and this gene is the most strongly upregulated of several clade 1 CCEs showing upregulation associated with the resistance (Han 2014).

Several polymorphisms in the voltage-sensitive sodium channel gene *para* have been associated with pyrethroid resistance in various insect species. The best characterised of these are the *knock-down resistance*, (*kdr*) allele, which is due to an L1014F/H/S substitution (numbered by alignment to the *Musca domestica* Vssc1 homolog AAB47604) in domain IIS6, and the *super-kdr* allele, which is due to this change in conjunction with M918T (Dong et al. 2014). Neither of these alleles were found in any of the *H. armigera* or *H. zea* sequences. Other sites in *para* previously reported to be associated with resistance to pyrethroids, two from *H. zea* (I936V, V410M/A/G) (Hopkins and Pietrantonio 2010) and two in *H. armigera* (D1549V + E1553G) (Head et al. 1998) were also found to be wildtype in both sequenced genomes.

Three copies of the γ-aminobutyric acid receptor gene *Resistance to dieldrin* (*Rdl*) were found in both the *H. armigera* and *H. zea* genomes (Fig. S36), as is the case in *B. mori* (Yu et al. 2010). Outside the Lepidoptera there is usually only one *Rdl* gene per genome, but duplicate copies have been associated with cyclodiene resistance in some species (Anthony et al. 1998; Remnant et al. 2013). In the latter an intermediate level of cyclodiene resistance is conferred by one of several mutations of the Ala302 residue, often Ser302 (ffrench-Constant et al. 1994), in one copy of the gene, while the other copy retains an Ala302 residue. The latter enables sufficient wild-type versions of the γ-aminobutyric acid receptor to be assembled to discharge its normal function and avoid a fitness cost in the absence of the insecticide. In Lepidoptera, the *Rdl1* and *Rdl2* genes are in close proximity on the Z chromosome, to which *H. armigera* scaffold_197 has been assigned by synteny to *B. mori* (Additional file 1: Table S1), and have Ala302 and Ser302, respectively, as wild-type sequences. The *Rdl3* gene has now been identified in the two heliothines, in *B. mori*, where it is located on an autosomal chromosome, and in *M. sexta* (Ms OGS2: Msex2.14174-RA). The two latter species have not been exposed to cyclodienes nor been reported to show any resistance to these insecticides. No *Rdl3* has yet been found in other Lepidoptera, including the available nymphalid genomes listed in Additional file 3: Figure S8. Although polymorphism for resistance to cyclodienes was reported in early literature on both the heliothines (Sparks 1981; Gunning and Easton 1994), the molecular mechanism was not reported and the resistance disappeared when use of these chemistries was subsequently discontinued. We therefore conclude that the variation found at residue 302 among the *Rdl* genes predates extensive divergence among lepidopteran lineages and is irrelevant to resistance in the heliothines.

Both *H. armigera* and *H. zea* genomes contain two acetylcholinesterase genes, *AChE1* (*Ha/HzOG200211*) and *AChE2* (*Ha/HzOG200212*). The sequences of the proteins are 99.2% and 100% identical and critical residues forming the catalytic triad are all conserved (S238, H480 and E367 using the processed *D. melanogaster* protein numbering; Oakeshott et al. 2005). Carbamate and organophosphate insecticides both act on acetylcholinesterase and multiple cases of resistance to these compounds have been reported across many insect species, almost always involving *AChE1* (Oakeshott et al. 2005; 2010). No substitutions associated with resistance in other Lepidoptera were found. One substitution associated with resistance outside Lepidoptera, S276G, was found in AChE1 (Table S25) but, as with the Ser302 change in *Rdl2* and *Rdl3* above, we find this to be the consensus residue in lepidopteran genomes, regardless of their resistance status.

Also included in Table S24 and Fig. S35 is information for two other P450 genes. For one of these, CYP303A1, there is no direct evidence of involvement in a particular resistance but a recent strong selective sweep has suggested a potential role in resistance to some modern insecticide(s) (Song et al. 2015). The other, CYP6AE14, has been implicated in the tolerance of *H. armigera* for gossypol and possibly other insecticidal secondary chemicals that are produced by the species’ host plants.

Genes associated with resistances in other species for insecticides now used on *H. armigera*

Additional file 6: Table S5 and Fig. S35 also include details of some other manually curated genes for which homologs in other species are known to confer resistance to particular insecticides but for which there is no evidence as yet for their resistance involvement in *H. armigera* or *H. zea*. This includes genes encoding sodium channel subunits targeted by neonicotinoids and spinosyn and genes encoding lipocalin, alkaline phosphatase and aminopeptidases associated with various Bt toxin resistances. No neonicotinoid or spinosyn resistance has yet been reported in the two heliothines. The Bt resistances characterised in them thus far have not implicated lipocalin, alkaline phosphatase or aminopeptidases.

Nicotinic acetylcholine receptors (nAChRs): nAChRs are Cys-loop ligand gated ion channels, a gene family present from bacteria to mammals. They mediate fast synaptic transmission in insect neurons and are targeted by insecticides. Resistance to neonicotinoid and spinosyn class insecticides has been associated with the modification or loss of function for genes in several nAChR subunit clades (neonicotinoids with the α1, α8 and β1 clades; Liu et al. 2005, Perry et al. 2008, Bass et al. 2011: spinosyns with the α6 clade; Perry et al. 2007). Twelve genes have been identified in the *H. armigera* genome, similar to the complement of other insects. Many are highly conserved in protein sequence to the 9 α subunits (Harα1-9) and the Harβ1 subunit found in *Bombyx mori* (Table S25). Divergent 2 clade subunits (Harβ2 *and* Harβ3) display low level identity and moderate similarity to *B. mori* orthologs.

Transcript expression, as detected by RNAseq, is low (Fig. S35), with the expression of most subunits concentrated in ventral nerve cord, with a few exceptions. The enrichment of two divergent 2 clade subunits, *Harβ2* and *Harβ3* in the larval heart concurs with reported expression of the *D. melanogaster* *Dβ3* divergent 1 clade subunit that is highly enriched in adult heart (Chintapilli et al. 2007). The other divergent subunit, *Harα9* appears to be expressed at an enriched level in the male testes and the male abdomen (probably due to the testes expression). This is another non-neuronal tissue where expression of nAChR subunit has been identified in *D. melanogaster*, but for which no functional role is known. The congruence of expression patterns between distantly related species suggests that the functional roles of most of these subunits are being maintained and, hence, functional studies conducted in one species may be of relevance to others.

Analysis of the effects of diet on transcript expression, despite not showing statistical significance, suggest nAChR subunit levels are impacted quite differently by various plant hosts (Fig. S35). Most noticeably tobacco leads to a trend in transcriptional up-regulation of many subunits (as nicotine does to protein levels of these receptors in mammals). Another striking result is the trend from cotton diet for higher expression levels of the heart enriched *Harβ2* and *Harβ3* subunits and possibly lower levels of neural expressed nAChR gene subunits. Another dietary source, Arabidopsis, also might be impacting expression levels of several subunits, suggesting it produces compounds that regulate the level, and possibly effect function, of insect nAChRs. The regulatory changes in these subunits could suggest that adaptive mechanisms for the polyphagous capacity of *H armigera* include transcriptional level alterations in expression of these subunits.

**Table S24. Genes associated with resistance to insecticides in *H. armigera* and *H. zea*.**

| Insecticide/  type | Gene family | Gene-name | HaOG number | *H. zea** | Tissue expression | Host-use DE | Mutation? | Ref |
| --- | --- | --- | --- | --- | --- | --- | --- | --- |
| Target-site | Na Channel | Paralytic, voltage-gated Na channel | HaOG202434 | y | antennae, ventral nerve |  | V418/I948 /L106 all wt** | 1,2 |
| Target-site | Z-linked GABA/ gcc | RDL1 | HaOG205336 | y | ventral nerve |  | A302 wt*** | 3 |
| Target-site | Z-linked GABA/ gcc | RDL2 | HaOG205341 | y | head, ventral nerve |  | S302 wt*** | 3 |
| Target-site | Z-linked GABA/ gcc | RDL3 | HaOG216493 | y | head, antennae, ventral nerve |  | Q302 wt*** | 3 |
| Target-site | ACE | AChE1 | HaOG200211 | y | antennae, ventral nerve, testes |  | wt**** | 4 |
| Target-site | ACE | AChE2 | HaOG200212 | y | antennae, ventral nerve |  | wt**** | 4 |
| Synthetic pyrethroids/ metabolic | CCE | CCE001g | HaOG200202 | y | foregut, midgut |  |  | 6 |
| Synthetic pyrethroids/ metabolic | P450 | CYP337B3 | n/a | n | n/a |  |  | 5 |
| Resistance? metabolic | P450 Z-linked | HaCYP303A1 | HaOG200007 | y | pupae, antennae |  |  | 7 |
| Gossypol/ metabolic | P450 | HaCYP6AE14 | HaOG200089 | n | Foregut, midgut | weak up/down on plants |  | 8; 9 |
|  |  |  |  |  |  |  |  |  |
| Cry1A type | cadherin | cadherin | HaOG208408 | y | midgut | up on tomato, capsicum, Arabidopsis | wt | 10, 11 |
|  |  |  |  |  |  |  |  |  |
| Cry2Ab | ABCA | ABCA2 | HaOG200329 | y | midgut | variably up on plants | wt | 12 |

* Whether an ortholog has been identified in the *H. zea* genome assembly.

** Numbering based on the genome-derived *H. armigera* gene model.

*** See text; numbering based on canonical sequence (ffrench-Constant et al. 1993)

**** See Oakeshott et al. (2005) for details of the sites identified as mutated in resistant *Drosophila*.

Table References: 1 Dong et al. (2007); 2 Hopkins and Pietrantonio (2010); 3 Daly and Fisk (1998); 4 Gunning et al. (2006); 5 Joußen et al. (2012); 6 Teese et al. (2010); 7 Song et al. (2015); 8 Mao et al. (2011); 9 Krempl et al. (2016); 10 Gomez et al. (2014); 11 Zhao et al. (2010); 12 Tay et al. (2015).

**Table S25. Protein similarity between *H. armigera* and *B. mori* nAChR subunit genes**

| *H. armigera gene* | *B. mori (*% identity / % similarity) |
| --- | --- |
| Harmα1 | 98/99 |
| Harmα2 | 95/98 |
| Harmα3 | 91/93 |
| Harmα4 | 88/90 |
| Harmα5 | 59/61 |
| Harmα6 | 96/98 |
| Harmα7 | 94/95 |
| Harmα8 | 94/96 |
| Harmα9 | 69/81 |
| Harmβ1 | 97/98 |
| Harmβ2 | 27/51 |
| Harmβ3 | 35/56 |

**Figure S35. Transcriptome profiles of genes related to insecticide susceptibility and resistance.** The genes shown are as listed in Table S24.


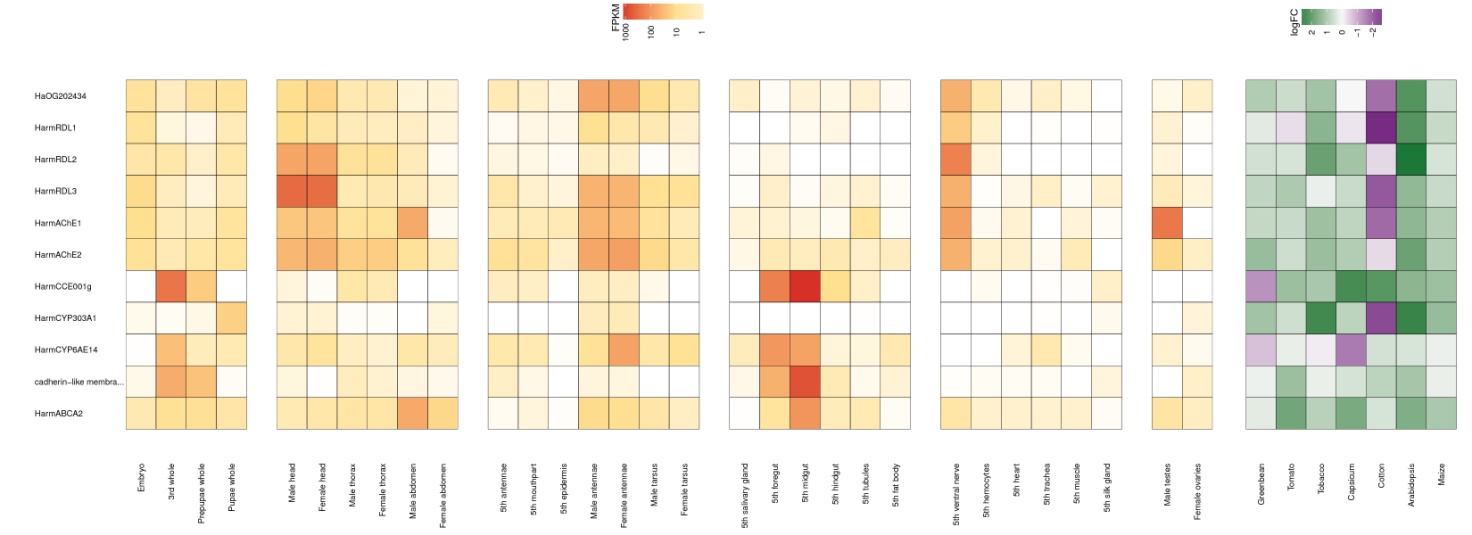


**Figure S36. Complete sequences for major isoforms of the *H. armigera* RDLs.** The sequences predicted from the genome were corrected using available transcriptome data. The *RDL-2* gene is incomplete in the genome assembly, with the 5’ portion missing due to the presence of a gap. The residues corresponding to A302 (*RDL1*), S302 (*RDL2*) and Q302 (*RDL3*) are marked in red.

RDL1 (isoform-3A6A_1, replaces HaOG205336)

MSGARPRSAPLLLALAAAFLPQANHVAGAGGGGMFGDVNISAILDSFSISYDKRVRPNYG

GPPVEVGVTMYVLSISSLSEVKMDFTLDFYFRQFWTDPRLAYKKRTGVETLSVGSEFIKN

IWVPDTFFVNEKQSYFHIATTSNEFIRIHYSGSITRSIRLTITASCPMNLQYFPMDRQLC

HIEIESFGYTMRDIRYHWKDGVTSVGMSNEVQLPQFRVLGHRQRATVVTLTTGNYSRLAC

EIQFVRSMGYYLIQIYIPSGLIVIISWVSFWLNRNATPARVALGVTTVLTMTTLMSSTNA

ALPKISYVKSIDVYLGTCFVMVFASLLEYATVGYMAKRIQMRKQRFVAIQKIASEKKIPV

DCPPVGDPHTLSKMGTLGRCPPGRPSEVRFKVHDPKAHSKGGTLENTINGGRSGAEEENP

GPPPHILHPGKDISKLLGMTPSDIDKYSRIVFPVCFVCFNLMYWIIYLHVSDVVADDLVL

LEEDK

RDL2 (isoform-3A_1, replaces HaOG205341)

MHTSRPRGVHSIALVLSLAIAWLPHADHAAGAGGGGMFGDVNISAILDSLSVSYDKRVRP

NYGGPPVDVGVTMYVLSISSLSEVKMDFTLDFYFRQFWTDPRLAYKKRTGVETLSVGSEF

IRNIWVPDTFFVNEKQSYFHIATTSNEFIRIHHSGSITRSIRLTITASCPMDLQYFPMDR

QLCNIEIESFGYTMRDIRYKWNEGPNSVGVSSEVSLPQFKVLGHRQRAMEISLTTGNYSR

LACEIQFVRSMGYYLIQIYIPSGLIVIISWVSFWLNRNATPARVSLGVTTVLTMTTLMSS

TNAALPKISYVKSIDVYLGTCFVMVFASLLEYATVGYMAKRIQMRKQRFTAVQKMAAEKK

MQIDGPPGSAEPVPPPRTSTLSRPPPPSRLSEVRFKVHDPKAYSKGGTLENTINGARGPA

PGPAPPADEEAGPPPHLVHASKGINKLLGTTPSDIDKYSRIVFPVCFVCFNLMYWIIYLH

VSDVVADDLVLLGEEN

RDL3 (isform-3A6B_1, replaces HaOG216493)

MSALRPRARRAPLLLALAAALLPHADRVAGGGGGGSMFGDVNISSILDSFSISYDKRVRP

NYGGPPVEVGVTMYVLSISSLSEVQMDFTLDFYFRQFWTDPRLAYKKRAGVETLSVGSEF

IRNIWVPDTFFVNEKQSYFHTATTSNEFIRIHHSGSITRSIRLTITASCPMNLQYFPMDR

QLCHIEIESFGYTMRDIRYKWNEGPNSVGVSNEVSLPQFKVLGHRQRAMEISLTTGNYSR

LACEIQFVRSMGYYLIQIYIPSGLIVIISWVSFWLNRNATPARVQLGVTTVLTMTTLMSS

TNAALPKISYVKSIDVYLGTCFVMVFTSLLEYATVGYMSKRIQMRKQRFVAIQKIMSEKK

IPAECAPPWDGHSLSKGGSLGRGARPPHGSHHGSHGSHGSHGSHGAPAPPPRGCGRHLQE

VRYKVRDPKAHSKGGTLESSIEDAPGPPLHALQPTKDYGKFLGMTPSDIDKYSRIVFPVC

FVCFNLMYWIVYLHVSDVVADDLVLLEENK

**References**

Anthony N, Unruh T, Ganser D, ffrench-Constant R(1998) Duplication of the Rdl GABA receptor subunit gene in an insecticide-resistant aphid, *Myzus persicae*. Molec. Gen. Genet. 260: 165-75.

Bass C, Puinean AM, Andrews M, Cutler P, Daniels M, Elias J, Paul VL, et al. R (2011) Mutation of a nicotinic acetylcholine receptor β subunit is associated with resistance to neonicotinoid insecticdes in the aphid *Myzus persicae*. BMC Neurosci. 12: 51.

Chintapalli VR, J Wang, Dow JA (2007)., Using FlyAtlas to identify better Drosophila melanogaster models of human disease. Nat. Genet. 39: 715-720.

Daly JC, Fisk JH (1998). [Sex-linked inheritance of endosulphan resistance in Helicoverpa armigera](https://scholar.google.com.au/citations?view_op=view_citation&hl=en&user=Fq7eFLAAAAAJ&cstart=40&citation_for_view=Fq7eFLAAAAAJ:Wp0gIr-vW9MC). Heredity 81: 55-62.

Dong K (2007). Insect sodium channels and insecticide resistance. Invert Neurosci 7: 17-30.

Dong K, Du Y, Rinkevich F, Nomura Y, Xu P, Wang L, Silver K, et al. (2014) Molecular biology of insect sodium channels and pyrethroid resistance. Insect Biochem. Molec. Biol. 50: 1-17.

Dvir H, Silman Y, Harel M, Rosenberry TL, Sussman JL (2010) Acetylcholinesterase: from 3D structure to function. Chem.Biol. Interact. 187: 10-22.

ffrench-Constant RH (1994) The molecular and population genetics of cyclodiene insecticide resistance. Insect Biochem. Molec. Biol. 24: 335-345.

ffrench-Constant RH, Rocheleau TA, Steichen JC, Chalmers AE (1993). A point mutation in a Drosophila GABA receptor confers insecticide resistance. Nature 363:449 – 451.

Gomez I, Sanchez J, Munoz‐Garay C, Matus V, Gill SS, Soberon M, Bravo A (2014). Bacillus thuringiensis Cry1A toxins are versatile proteins with multiple modes of action: two distinct prepores are involved in toxicity. Biochem. J. 459: 383-396.

Gunning RV, Easton CS (1994). Endosulfan resistance in Helicoverpa armigera (Hubner) (Lepidoptera: Noctuidae) in Australia. J. Aust. Ent. Soc. 33: 9-12.

Gunning RV, Moores GD, Devonshire A (1996). Insensitive acetylcholinesterase and resistance to thiodicarb in Australian Helicoverpa armigera Hübner (Lepidoptera: Noctuidae). [Pestic. Biochem. Physiol](http://www.sciencedirect.com/science/journal/00483575). 55: 21-28.

Han Y (2014). Resistant mechanisms to fenvalerate in *Helicoverpa armigera* from China. PhD thesis, Nanjing Agricultural University, People’s Republic of China.

Head DJ, McCaffery AR, Callaghan A. (1998). Novel mutations in the para-homologous sodium channel gene associated with phenotypic expression of nerve insensitivity resistance to pyrethroids in heliothine Lepidoptera. Insect Molec. Biol. 7: 191-196.

# Hopkins BW, Pietrantonio PV (2010). The *Helicoverpa zea* (Boddie) (Lepidoptera: Noctuidae) voltage-gated sodium channel and mutations associated with pyrethroid resistance in field-collected adult males. Insect Biochem. Molec. Biol. 40: 385-393.

Joußen N, Agnolet S, Lorenz S, Schöne SE, Ellinger R, Schneider B, Heckel DG (2012). Resistance of Australian Helicoverpa armigera to fenvalerate is due to the chimeric P450 enzyme CYP337B3. [Proc. Natl. Acad. Sci. USA](https://www.ncbi.nlm.nih.gov/pmc/articles/PMC3458352/). 109: 15206–15211.

Krempl C, Heidel-Fischer HM, Jiménez-Alemán GH, Reichelt M, Menezes RC, Boland W, Vogel H, et al. (2016). Gossypol toxicity and detoxification in *Helicoverpa armigera* and *Heliothis virescens*. Insect Biochem. Molec. Biol. 78: 69-77.

Liu ZW, Williamson MS, Lansdell SJ, Denholm I, Han ZJ, Millar NS (2005). A nicotinic acetylcholine receptor mutation conferring target-site resitance to imidacloprid in Lilaparvata lugens (brown planthopper). Proc. Natl. Acad. Sci. USA 102: 8420-8425.

Mao YB, Tao XY, Xue XY, Wang LJ, Chen XY (2011). Cotton plants expressing CYP6AE14 double-stranded RNA show enhanced resistance to bollworms. Transgenic Res. 20: 665-673.

Oakeshott JG, Claudianos C, Russell RJ (2010). New genomic perspectives on insect esterases and insecticide resistance. In (Gilbert LI, Iatrou K, Gill SS eds) Comprehensive Molecular Insect Science, vol 5 Pharmacology. Elsevier BV, Oxford UK pp 302-306.

Oakeshott JG, Claudianos C, Campbell PM, Newcomb RD, Russell RJ (2005). Biochemical genetics and genomics of insect esterases. In (Gilbert LI, Gill SS eds) Pharmacology: Channels, Receptors, Toxins and Enzymes. Elsevier BV, Oxford UK pp 309-381.

Perry T, McKenzie JA, Batterham P (2007). A Dα6 knockout strain of *Drosophila melanogaster* confers a high level of resistance to spinosad.  Insect Biochem. Molec. Biol. 37: 184-188.

Perry T, Heckel DG, McKenzie JA, Batterham P (2008). Mutations in Dα1 or Dβ2 nicotinic acetylcholine receptor subunits can confer resistance to neonicotinoids in Drosophila melanogaster.  Insect Biochem. Molec. Biol. 38: 520-528.

Remnant EJ, Good RT, Schmidt JM, Lumb C, Robin C, Daborn PJ, Batterham P (2013). Gene duplication in the major insecticide target site, Rdl, in *Drosophila melanogaster*. Proc. Natl. Acad. Sci. USA. 110: 14705-14710.

Song SV, Downes S, Parker T, Oakeshott JG, Robin C (2015). High nucleotide diversity and limited linkage disequilibrium in *Helicoverpa armigera* facilitate the detection of a selective sweep. Heredity 15:460-470.

Sparks TC (1981) Development of resistance in Heliothis zea and Heliothis virescens in North America. Bull. Ent. Soc. Amer. 27: 186-192.

Tay WT, Mahon RJ, Heckel DG, Walsh TK, Downes S, James WJ, Lee SF, et al. (2015). Insect resistance to Bacillus thuringiensis toxin Cry2Ab is conferred by mutations in an ABC transporter subfamily A protein. [PLoS Genet](https://www.ncbi.nlm.nih.gov/pmc/articles/PMC4652872/). 11: e1005534.

Teese MG, Campbell PM, Scott C, Gordon KHJ, Southton A, Hovan D, Robin C, et al. (2010) Gene identification and proteomic analysis of the esterases of the cotton bollworm, *Helicoverpa armigera*. Insect Biochem. Molec. Biol. 40: 1-16.

Yu LL, Cui YJ, Lang GJ, Zhang MY, Zhang CX (2010), The ionotropic & gamma-aminobutyric acid receptor gene family of the silkworm, *Bombyx mori*. Genome 53: 688-97.

Zhao J, Jin L, Yang Y, Wu Y (2010). Diverse cadherin mutations conferring resistance to *Bacillus thuringiensis* toxin Cry1Ac in *Helicoverpa armigera*, Insect Biochem. Molec. Biol. 40: 113-118.

**Section 13. Detailed methods for the *Helicoverpa* genome assemblies and annotation**

***H. armigera* genome sequencing and assembly**

Material

The general rearing strain (known as GR) has been in culture in Canberra since the mid-1980s. It is derived from material collected from cotton fields in the Namoi Valley, northern NSW, Australia. To maintain diversity, it has subsequently been supplemented with additional collections from the same area, although not in the past 10 years, over which time it has remained stable in the laboratory.

Library construction and reads available for assemblies

DNA was extracted from several individual male pupae derived from a single pair mating of GR individuals using a phenol chloroform extraction protocol. The DNA was quantified using a Qubit fluorometer (Life Technologies, Carlsbad, CA) and run on an agarose gel to confirm quality.

Library construction and sequencing were performed at the BCM HGSC. A number of different types of sequencing libraries on both the 454 and Illumina platforms were generated at the BCM HGSC using standard protocols. The libraries are listed in Table S26.

DNA from a single individual, Male_#8, was the source of the 300 b paired end (PE) and 3 kb mate pair (MP) Illumina reads; the DNA providing the Illumina 6 kb and 8 kb reads all derived from individual Male_#2; and that for the 180 b PE and 454-XLR fragment reads all derived from individual Male_# 1. Sequencing runs were as follows:

Male_#1 (Genbank Biosample # 6608622): 3 runs of 454 Titanium, unpaired; 2 lanes of HiSeq, 2x100, 180 b PE

Male_#8 (Genbank Biosample # 6608624): 1 lane of GA II, 2x95, 300 b PE; 1 lane of HiSeq, 2x100, 300 b PE; 2 lanes of HiSeq, 2x100, 3 kb MP

Male_#2 (Genbank Biosample # 6608623): 1 lane of GA II, 2x125, 8 kb MP; 1 lane of HiSeq, 2x100, 6 kb MP

Duplication rates in all libraries and paired-end contamination in the MP libraries were determined using standard in-house processes at BCM-HGSC and FASTQC. The 180 b PE and the 3 kb MP reads were acceptable without additional processing but the 6 and 8 kb MP libraries required cleaning using standard in-house processes at BCM-HGSC due to high duplication rates and some PE contamination.

An additional 20 kb insert size MP library was generated for 454-XLR sequencing using DNA from a single offspring of a different single pair cross within GR (Biosample # 6608625).

**Table S26. *H. armigera* assemblies, showing reads used, assembler and assembly metrics.**

|  | MP Reads: insert size(b) | JCVI-01-Moth | BCM-1 | BCM-2 | csiro4bp** |
| --- | --- | --- | --- | --- | --- |
| Assembly date |  | Apr-2012 | Oct-2011 | Oct-2011 | Jan-2012 to April-2015 |
| Assembler used |  | CABOG | Allpaths-LG | Allpaths-LG | Allpaths-LG |
| Reads: 454 |  | 3.8M (6x) | - | - | - |
| Reads: Illumina 180 b PE * | 180 | 818M (240x) | 374M (80.3x) | 374M (80.3x) | 374M (80.3x) |
| Reads: Illumina 300 b | 280 | 304M (88x) | 304M (62.3x) | 304M (62.3x) | - |
| Reads: Illumina  3 kb MP | 2,505±444 | 435M (128x) | 435M (128x) | 435M (128x) | 435M (128x) |
| Reads: Illumina 6 kb MP | 5,204±515 | 129M (38x) | - | 129M (38x) | 129M (38x) |
| Reads: Illumina 8 kb MP | 6,570±886 | 58M (21x) | - | 58M (21x) | 58M (21x) |
| Reads: 454 20 kb MP | 19,800±4000 |  |  |  | 3.14M |
| Scaffolds: |  |  |  |  |  |
| Total length (b) |  | 413,696,214 | 363,659,696 | 400,937,480 | 337,072,266 |
| No. of Ns (gaps) |  | 1.5 Mb | 21.3 Mb | 48.5 Mb | 37.1 Mb |
| No. of scaffolds |  | 34,444 | 3441 | 921 | 997 |
| Largest scaffold |  | 477,680 | 1,530,003 | 3,561,802 | 6,146,628 |
| N50 length (number) |  | 41,222 (1,923) | 137,819 (660) | 625,844 (144) | 1,000,414 (93) |
| N90 length (number) |  | 13,339 (8,303) | 45,082 (2,360) | 155,014 (603) | 175,335 (417) |
| Contigs: |  |  |  |  |  |
| Contigs –total |  | 412,201,358 | 341,311,048 | 352,434,760 | 299,631,163 |
| No of contigs |  | 69,570 | 68,110 | 64,882 | 24,228 |
| Largest contig |  | 121,777 | 258,359 | 166,742 | 291,622 |
| N50 length (number) |  | 17,867 (8,447) | 16,016 (5,653) | 17,851 (5,190) | 18,556 (4,899) |
| N90 length (number) |  | 5683 (23,974) | 3,620 (22,486) | 4,666 (19,415) | 1,500 (>24,228) |

N50 and N90 are computed on the csiro4bp assembly size (337 Mb).

* reads listed as total sequence, with genome coverage in brackets

** see text for details of data treatment; the final patched version csiro4bp is file csiro4b-r-04-04-2015.fas

Allpaths-LG assemblies at Baylor

After a series of trial assemblies, two final assemblies were produced using Allpaths-LG (Gnerre et al. 2010) at BCM-HGSC. They differed in the addition of the 6 kb and 8 kb MP reads to the second assembly (Table S26). This resulted in a very large increase in the scaffold N50, from 138 kb to 626 kb, while decreasing the number of scaffolds from 3441 to 921. The overall genome length increased only slightly from 364 Mb to 401 Mb.

Allpaths-LG assemblies at CSIRO

The first step in pre-processing the raw reads involved removal of the 3’ padding provided by Illumina using JustPreprocessMyReads (http://justpreprocessmyreads.sourceforge.net). Then k-mer histograms were produced for each readset and, for the 454 data only, k-mer-based error correction was performed by a pre-publication version of Blue (Greenfield et al. 2014) using the Illumina readsets as a source of correct k-mers.

Assemblies were performed using Allpaths-LG (v39099). A preliminary Allpaths-LG assembly was used to correct the size distribution statistics of each library, to purge small insert contaminant reads from the MP libraries and perform quality control of the raw data. A derivative readset from MP data was produced by creating ‘unipaths’ using only the PE data and then using Allpaths ErrorCorrection to purge any MP data not supported by the unipaths. This was done to purge any haplotype blocks that were specific to the individual genome from which the MP data were generated. Subsequent assemblies were conducted manually using the Allpaths-LG software but not the Allpaths-LG automated pipeline.

The assemblies produced used different combinations of the input data, including at times data produced by other sequencing technologies (single-ended Illumina and 454) or the CABOG assembler (see below). For each assembly the raw data was back-aligned and a process of quality assessment carried out in order to select a final, preferred assembly. This included: 1) using hagfish (https://github.com/mfiers/hagfish) to scan for regions where the distance of paired reads was above or below expectations; 2) protein alignments to assess accuracy in assembling clustered and closely related paralogs from a core detoxification gene set; 3) assessing the number of linkage group violations from an earlier version of a female informative linkage map based on Rad-tags (our unpublished work); 4) reviewing some automated statistics such as N50 and G50 (where G is the genome size as estimated by flow cytometry) and the number of alignments from RNA-Seq reads or proteins from a manually curated gene set (see below).

The final assembly (‘csiro4b’) was chosen for further analysis based on the above analyses. This assembly was composed of the following libraries: the 180 b PE library, the 3 kb, 6 kb and 8 kb MP libraries purged of haplotype blocks using Allpaths ErrorCorrection as described above, and the 20 kb MP 454 libraries. For this assembly, the 6 and 8 kb data were introduced as ‘long jumps’ to Allpaths-LG and therefore did not contribute to base sequence composition. All MP libraries were purged of data not supported by unipaths formed by the 180 b PE library. Other assemblies, such as those including more data (eg the 300 b PE reads), were deemed of lower quality but kept to be used in improving the annotation effort below.

*H. armigera* assembly with CABOG

Sequence data (see Table S26) were assembled with CABOG 6.1 (Miller et al. 2008), a 454-enabled variant of the Sanger-era Celera Assembler (Myers et al. 2000). The CABOG assembly used primarily the 454 data for computing unitig and contig connectivity and consensus sequences. The assembly used the Illumina paired end data only for trimming the 454 reads. The assembly scaffold step used a reduced set of Illumina mate pairs, those that mapped to separate unitigs, plus all the 454 mate pairs. All Illumina data were pre-processed with CABOG’s fastqAnalyze, fastqToCA, and merTrim tools. The Illumina mate pairs were additionally processed with CABOG’s dedupe and classifyMates tools. The 454 data were pre-processed with CABOG’s sffToCA (with options –clear 454 and –trim hard in order to remove proprietary adapter) and CABOG’s overlap-based-trimming (OBT) process. The OBT step was configured to use Illumina data as evidence and 454 data as target. Finally, the Celera Assembler pipeline was run with the following parameters: obtMerSize = 22, ovlMerSize = 22, doFragmentCorrection = 0, unitigger = bogart.

BAC sequencing

No complete and error-free assembly was available from any of the above approaches for three genomic regions encoding the major gene expansions of the clade1 CCEs, trypsins and chymotrypsins. Each of these regions contains numerous very closely related genes, with the assembly challenge exacerbated by the likely presence of multiple haplotypes in the individuals sequenced. To provide additional sequence information derived from a single haplotype origin for each of these three regions, two BACs covering each of the regions were selected from the available library described by d’Alencon et al. (2010) using probes derived from genes in each of these regions. BAC DNA was purified and sequenced at BCM-HGSC using the Illumina platform to generate 180b PE reads. Due to the limited data resulting, only short contigs spanning several gene lengths could be assembled. However these allowed correction of several genes in the final assembly (below).

Correction of the csiro4b assembly to give csiro4bp

The csiro4b assembly was used in the initial generation of gene models. These models were used in the comprehensive genome annotation process outlined below, which also used all the other available genome and transcriptome data and assemblies. Upon completion of this annotation process, a set of 100 sequence corrections were collected corresponding to genes whose sequence had been incorrectly assembled in csiro4b (in part due to heterozygosity within the samples sequenced). A total of 85 locations in 46 scaffolds were affected, with 19 of these having multiple corrections. A custom perl script was used to replace the appropriate part of the csiro4b sequence with corresponding corrected sequences in such a manner (specifically, by modifying the lengths of internal or adjacent gaps) that no change was made to the coordinates of downstream sequences. The intermediate assemblies are available upon request from the authors.

***H. zea* genome sequencing and assembly**

Material

Pupae/pharate adults from a highly inbred line of *H. zea* at USDA-ARS, Stoneville, Ms, were used for isolation of genomic DNA. The colony had been inbred for about 20 years without any introduction of outside genetic material. About 20 pupae were selected for DNA extractions. After quality checking, gDNA from eight separate individuals was sent to BCM-HGSC for library construction and sequencing.

Three libraries were sequenced, generating 180 b PE reads and 3 kb and 5 kb MP reads. Each library used DNA from a different individual, giving the coverage shown in Table S27. The three individuals used were H-zea-1 (Biosample # 6608705), H-zea-3 (Biosample # 6608706) and H-zea-7 (Biosample # 6608707).

Assembly using Allpaths-LG

Assemblies of the *H. zea* genome using Allpaths-LG are listed in Table S27. The first draft assembly from the BCM HGSC was gap-filled using the in-house tool Atlas gap-fill v.2.2 (<https://www.hgsc.bcm.edu/software/atlas-gapfill>), and for the second Allpaths-LG was run in haploidified mode. Several further assemblies were done at CSIRO using the procedures detailed above for the *H. armigera* assemblies.

A further *H. zea* genome assembly (Hz5d in Table S27) used the revised Pilon implementation of the de-Bruijn graph based assembler (Walker et al. 2014) to correct and gap-fill the preferred genome assembly (hz5, see below) and SNAP-aligner to map reads to the genome and maintain unique alignments. Indel error correction and gap-filling was carried out using five consecutive runs of Pilon operating in local realignment, gap filling and fix ambiguous base modes. A sixth and final run was used to *de novo* reassemble the remaining reads into scaffolds for increased gene recovery.

Assembly correction

Assembly Hz-csiro5 (hz5) was selected as the reference assembly for annotation after quality analysis as described above for the *H. armigera* genome and subsequent correction. The genes and genome regions requiring correction were identified after mapping the OGS2 gene models from *H. armigera* onto the hz5 assembly using Scipio as described under Annotation below. The other assemblies (Table S27) and available transcriptome data were then screened in the same manner and the genome carrying a sequence which best corresponded to each *H. armigera* model in length and aminoacid sequence homology was identified. These sequences were then patched into the reference sequence (hz5) as per the methods for *H. armigera* above to give the final corrected version, hz5p5. The intermediate assemblies are available upon request from the authors.

**Table S27. *H. zea* genome assemblies.**

|  | Indiv. | BCM-Hz1 | BCM-Hz-hap | Hz-csiro5 | Hz-csiro6 | Hz-csiro7 | Hz5d | hz5p5 |
| --- | --- | --- | --- | --- | --- | --- | --- | --- |
|  |  | Gap-fill | haploidified | diploid | Haploidified |  | Pilon infilled | Patched csiro5 |
| date |  | 29-Feb-2012 | 17-May-2012 | Feb-2012 | Feb-2012 | Feb-2012 | Mar-2015 | 2015 |
| Reads |  |  |  |  |  |  |  |  |
| 180 b PE | H-zea-3 | 314M (78.7x) | 314M (78.7x) | 314M (78.7x) | 314M (78.7x) | 314M (78.7x) |  | 314M (78.7x) |
| 3 kb MP (Insert: 2.2 kb) | H-zea-1 | 188M (34.7x) | 188M (34.7x) | 188M (34.7x) | 188M (34.7x) | 188M (34.7x) |  | 188M (34.7x) |
| 5 kb MP (Insert: 4 kb) | H-zea-7 | 206M (22.3x) | 206M (22.3x) | 206M (22.3x) | 206M (22.3x) | 206M (22.3x) |  | 206M (22.3x) |
| scaffolds |  |  |  |  |  |  |  |  |
| Total length (b) |  | 373,248,076 | 363,239,500 | 335,770,044 | 332,240,745 | 333,394,411 | 334,372,984 | 341,147,348 |
| No. of Ns (gaps) |  |  |  | 34.9 Mb | 28.8 Mb | 35.4 Mb |  | 34.9 Mb |
| No. of scaffolds |  | 3,504 | 5,715 | 2,976 | 2,460 | 2,870 | 2976 | 2,975 |
| Largest scaffold |  | 2,822,074 | 1,571,763 | 1,818,188 | 1,770,424 | 1,479,724 | 1,827,266 | 1,847,547 |
| N50 length (number) |  | 377,780 (271) | 172,452 (546) | 195,048 (481) | 250,073 (356) | 196,498 (482) | 192,764 (484) | 201,477 (469) |
| N90 length (number) |  | 117,292 (913) | 50,740 (1,941) | 46,599 (1,848) | 49,560 (1,527) | 45,348 (1,859) | 44,349 (1,875) | 52,415 (1,752) |
| Genes |  |  |  |  |  |  |  |  |
| Genes* used |  | 1695 | 430 | 11599 | 1494 | 686 | 410 | (other:19)** |
| Genes in families*** |  | 170 | 55 | 1698 | 208 | 89 | 36 | (other: 13)** |

N50 and N90 are computed on the hz5p5 assembly size (341 Mb).

* Number of genes per assembly used to generate the patched assembly hz5p5.

** For those genes not obtained from one of the assemblies listed, the best available coding sequence was derived from the transcriptome data.

*** Number per assembly of genes in annotated gene families used to generate the patched assembly hz5p5.

***H. armigera* genome annotation**

Automated annotations

Annotation proceeded through a series of steps which are described in detail below. Initial annotation was performed using a series of MAKER runs, following by the use of a customized method, built on OrthoMCL+CD-HIT, to integrate the resulting gene sets. The resulting output became an evidence input into PASA2 to yield an improved complete gene set. This was then reconciled with manually validated gene models for key families (mapped back against the genome using Scipio) to produce the final official gene set.

*Annotation with MAKER.* The initial annotations of the csiro4b assembly were carried out using the MAKER pipeline (Cantarel et al. 2008). The main steps include masking repetitive elements, aligning EST, RNA-Seq assemblies and protein homology evidence, *ab initio* gene prediction, and finally producing a consensus set of gene models based on the alignment evidences and gene models predicted by each individual gene predictor. Three well-known *ab initio* gene prediction tools, Augustus (Stanke et al. 2008), SNAP (Korf 2004) and GeneMark (Lomsadez et al. 2005) were chosen to embed in MAKER. In order to increase the gene prediction performance, Augustus and SNAP were trained using a set of 366 manually curated homologous proteins (curated proteins) from *Drosophila melanogaster* (Dmel) and *Heliconius. melpomene* (Hmel), which also served as an evidence databases.

The first MAKER run yielded 10,059 gene models. After adding the *Danaus plexippus* genome data and 2,670 *H.armigera* RNA-Seq gene model assemblies (selected from the complete RNA-seq data) to the evidence database, 13,049 gene models were produced from the second MAKER run. Later on, Augustus and SNAP were re-trained using the RNA-seq assemblies with the expectation of quantity improvement, however, it tended to produce less gene models in the third MAKER run. The advanced feature of re-annotation in MAKER was employed in three following MAKER runs (Table S28), which fed the annotated gene models from a finished MAKER run as a starting point to the new run. Such a reiterative approach, together with extra evidence datasets from non-lepidopteran species (listed in Table S28) added to the pipeline, finally generated 17,357 gene models in the sixth MAKER run. Moreover, 2,577 non-redundant training genes were extracted from the curated proteins and RNA-Seq assemblies with the purpose to further optimize the prediction models in Augustus and SNAP, which was used in the seventh MAKER run. Then, 2/3 of the non-redundant set genes were randomly chosen to train Augustus and SNAP in the eighth and ninth MAKER run, respectively (Table S28). The number of predicted genes and the average length of proteins reported by MAKER (see Table S29 for examples) turned out to be stable (around 15,700 and 495 amino acids) in the last three runs.

*OrthoMCL+CD-HIT.* A customized method, OrthoMCL+CD-HIT, (Fig. S37) was developed to assess the quality of predicted genes and eventually consolidate similar genes among them from multiple MAKER runs into consensus sets. OrthoMCL (Li et al. 2003), an identification tool for orthologous or paralogous proteins from eukaryotic genomes, was used to group annotated proteins from the second MAKER run to the ninth MAKER run. The first MAKER run was not involved in this process, since it only produced 10,059 candidate genes. Three major steps were included in OrthoMCL: BLAST all-vs-all comparison to determine sequence similarity, a rule-based approach to draw sequence relationships, and the formation of ortholog groups using the clustering algorithm MCL (Van Dongen 2000). To increase the stringency of the grouping, any cluster contained proteins from less than five different MAKER runs was filtered out, which led to a total number of 16,515 ortholog groups.

Then, CD-HIT (Li and Godzik 2006), a cluster program based on sequence similarity, was used to further cluster proteins from each ortholog group. It is quite possible that more than one proteins from a single MAKER run fell into one ortholog group, which might cause clustering bias and pattern over-representation. Thus, CD-HIT only scanned ortholog groups that comprised one and only one protein per MAKER run. Each group was clustered into sub-groups with a threshold of 100% similarity. Sub-groups, which only contained a single protein, were removed after CD-HIT.

The nine MAKER runs and the OrthoMCL+CD-HIT approach together produced 18,636 novel proteins (Table S30), which were then included as evidence in the final annotation using EvidenceModeler (Haas et al. 2008) and PASA2 (Haas et al. 2003), and the manual curation analyses, as described below and in the Materials and Methods of the main text.

Functional annotation of gene models in key families

The automatically generated gene models for the key gene families were cross-checked and manually curated using all available sequences, cDNAs and gene models. For the major detoxification and insecticide resistance gene families, ie the cytochrome P450s (P450s), carboxyl/cholinesterases (CCEs), glutathione S-transferases (GSTs), UDP-glucuronosyltransferases (UGTs) and ATP-binding cassette (ABC) transporters, the automatically generated gene models for *H. armigera* were cross-checked and manually curated by domain specialists using available sequences, cDNAs and gene models generated by a specially developed gene finding and alignment pipeline (Clarke et al. as cited in Rane (2017) and Rane et al. (2017)). Briefly, this involved exhaustive exonerate analysis (Slater and Birney 2005) of the genome and transcriptome data for those species using all available gene models for those families from other insect species. The resulting hits were then reconciled and integrated prior to checking for model completeness using Blast (Altschul et al. 1990) and, finally, careful inspection from experts in the particular gene families. This enabled us to derive a unified set of gene models for each locus in the two heliothines for the gene families in question. As noted, where these models differed from those in the final assemblies, the latter were then patched appropriately. Other families listed in the comprehensive family annotation table (Additional file 6: Table S5) were annotated based on either the use of custom perl scripts to identify proteins with specific motifs (eg the cuticular proteins) or by the semi-automated screening of BLAST-derived annotations.

***H. zea* genome annotation**

In the first instance the annotation process for *H. zea* was based on PASA2. In this case the transcriptome data was as described in the Materials and Methods of the main text. RNA was pooled from mixed life stages (24-48 hour embryos, all larval stages, pupae, and adult males and females). RNA sequence data was used to assemble transcripts using Trinity (version trinityrnaseq_r20140413p1) and genome-guided and *de novo* assembly methods. However the protein models generated by PASA2 were more numerous (27,737 protein models from 20,846 gene loci), shorter in length (encoding an average of 418 vs 509 amino acid residues) and generally not of the same quality as those from *H. armigera*. This was probably because much less genome sequence and RNA-Seq data were available for *H. zea*.

The PASA2 gene set derived for *H. zea* was therefore set aside and overall genome annotation conducted using the *H. armigera* OGS2. The protein sequences encoded by the *H. armigera* official gene set were mapped directly to the hz5 genome sequence using Scipio. Scipio proved very useful because it is capable of annotating genes across multiple scaffolds to account of fragmented genes in draft assemblies. Most of the *H. armigera* gene models could be mapped in this way and a majority of the orthologous *H. zea* genes obtained were of comparable quality and similar sequence and length (see Results in main text). Manually annotated gene families were finally used to replace corresponding genes to form the first official gene set (OGS), consisting of a total of 15,205 gene models derived from the *H. zea* assembly (after patching as described above). This involved repeating the process of Scipio mapping of genes using the available *H. zea* genome assemblies in Table S27. Of these 15,205, 14,371 gene models were present on a single scaffold, 790 bridged two scaffolds and 40 bridged three or more scaffolds. Most (11,599) of the gene models were derived from hz5, with the others being as in Table S27. If only gene models corresponding to those from *H. armigera* annotated as being in gene families are considered, again most (1,698) were derived from hz5 (Table S27).

**Figure S37. Overview of OrthoMCL+CD-HIT workflow for *H. armigera.***


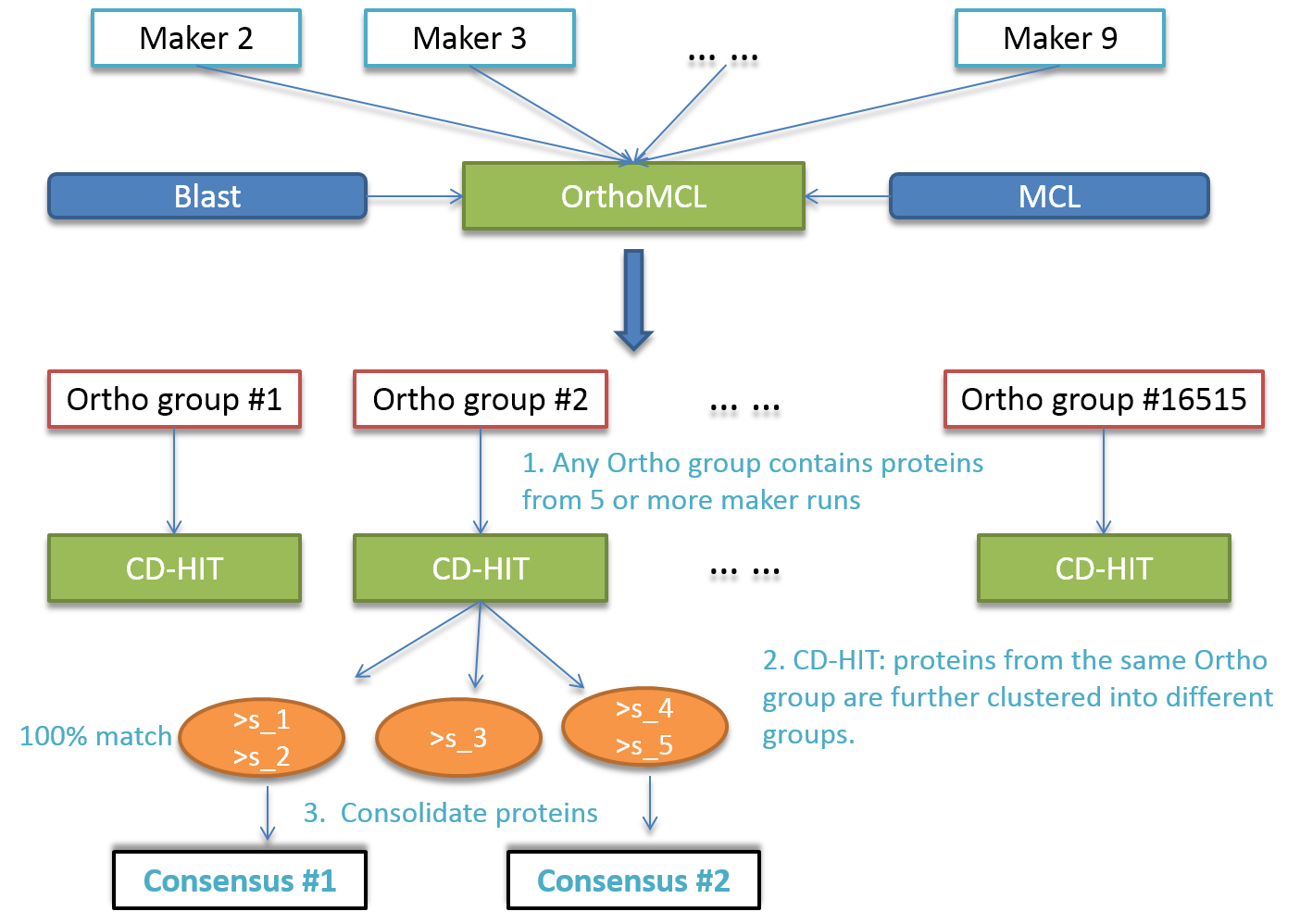


**Table S28. Statistics for nine MAKER runs for *H. armigera.***

| Maker Run | Training Gene Set | Re-annotated | Homology Ref | Predicted Proteins | Average Length (amino acids) | RNA-Seq |
| --- | --- | --- | --- | --- | --- | --- |
| 1 | Curated set: 366 | No | Dmel, Hmel | 10059 | 521 | No |
| 2 | Curated set: 366 | No | Dmel, Hmel,Dple | 13049 | 516 | Yes |
| 3 | RNA-Seq set: 2670 | No | Dmel, Hmel,Dple | 13017 | 526 | Yes |
| 4 | Curated set: 366 | Yes  (Maker 2) | Dmel, Hmel,Dple | 14109 | 506 | Yes |
| 5 | Curated set: 366 | Yes  (Maker 4) | Dmel, Hmel, Dple, Apis, Amel, Bmor, Tcas | 15719 | 489 | Yes |
| 6 | Curated set: 366 | Yes  (Maker 5) | Dmel, Hmel, Dple,Apis, Amel, Bmor, Tcas | 17357 | 480 | Yes |
| 7 | Curated set + RNA-Seq: 2577 | No | Dmel, Hmel, Dple, Apis, Amel, Bmor, Tcas | 15544 | 500 | Yes |
| 8 | 2/3 of (Curated set + RNA-Seq): 1804 | No | Dmel, Hmel, Dple, Apis, Amel, Bmor, Tcas | 15767 | 491 | Yes |
| 9 | 2/3 of (Curated set + RNA-Seq): 1803 | No | Dmel, Hmel, Dple, Apis, Amel, Bmor, Tcas | 15878 | 495 | Yes |

**Table S29. Detailed gene model statistics of three selected MAKER runs for *H. armigera.***

| Gene Stats | Maker 3 | Maker 6 | Maker 7 |
| --- | --- | --- | --- |
| Count | 13,017 | 17,332 | 15,544 |
| Average Length | 7,755.88 | 6,637.76 | 7,061.14 |
| Median Length | 4,452 | 3,664 | 3,991 |
| Total Length | 100,958,297 | 115,045,682 | 109,758,411 |
| Average Coding Length | 1,580.59 | 1,456.87 | 1,503.67 |
| Median Coding Length | 1,170 | 1,002 | 1,077 |
| Total Coding Length | 20,574,549 | 25,250,502 | 23,373,123 |
| Ave Exons Per | 7.22 | 6.28 | 6.59 |
| Med Exons Per | 5 | 4 | 4 |
| Total Exons | 93,994 | 108,796 | 102,440 |

**Table S30. Statistics for the gene models obtained in each step of OrthoMCL+CD-HIT for *H. armigera.***

| Maker proteins per Ortho group (one and only one per Maker run) | Ortho-groups | CD-hit clusters | Total number of proteins across all groups | Consensus proteins (CD-hit collapsed set) |
| --- | --- | --- | --- | --- |
| 8 | 9,727 | 23,923 | 77,816 | 15,222 |
| 7 | 397 | 909 | 2,779 | 567 |
| 6 | 664 | 1,228 | 3,864 | 843 |
| 5 | 1,630 | 2,769 | 8,150 | 2,004 |
| Total number of gene models generated | 12,418 | 28,829 | 92,609 | 18,636 |

**References**

D’Alencon E, Sezutsu H, Legeai F, Permal E, Bernard-Samain S, Gimenez S, Cagneur C, et al. (2010). Extensive synteny conservation of holocentric chromosomes in Lepidoptera despite high rates of local genome rearrangements. Proc. Natl. Acad. Sci. USA 107: 7680-7685.

Besemer J, Borodovsky M (2005). GeneMark: web software for gene finding in prokaryotes, eukaryotes and viruses. Nucleic Acids Res. 33: W451-W454.

Cantarell BL, Korf I, Robb SM, Parra G, Ross E, Moore B, Holt C, et al. (2008). MAKER: an easy-to-use annotation pipeline designed for emerging model organisms. Genome Res. 1: 188-196.

Gnerre S, MacCallum I, Przybylski D, Ribeiro FJ, Burton JN, Walker BJ, Sharpe T, et al. (2010). High-quality draft assemblies of mammalian genomes from massively parallel sequence data. Proc.Natl. Acad.Sci. USA 108: 1513–1518.

Greenfield P, Duesing K, Papanicolaou A, Bauer DC (2014). Blue: correcting sequencing errors using consensus and context. Bioinformatics 30: 2723–2732.

Haas BJ, Delcher AL, Mount SM, Wortman JR, Smith RK Jr, Hannick LI. et al. (2003). Improving the Arabidopsis genome annotation using maximal transcript alignment assemblies. Nucleic Acids Res. 31:5654–5666.

[Haas BJ, Salzberg SL, Zhu W, Pertea,M Allen JE, OrvisJ, Wortman JR (2008). Automated eukaryotic gene structure annotation using EVidenceModeler and the Program to Assemble Spliced Alignments. Genome Biol. 9: R7.](http://doi.org/10.1186/gb-2008-9-1-r7)

Hatje K, Kollmar M (2014). Kassiopeia: a database and web application for the analysis of mutually exclusive exomes of eukaryotes. BMC Bioinformatics 15: 115.

Keller O, Odronitz F, Stanke M, Kollmar M, Waak S (2008). Scipio: using protein sequences to determine the precise exon/intron structures of genes and their orthologs in closely related species. BMC Bioinformatics 9: 278.

Korf I (2004). Gene finding in novel genomes. BMC Bioinformatics 5: 59.

Lomsadze A, Ter-Hovhannisyan V, Chernoff Y, and Borodovsky M (2005). Gene identification in novel eukaryotic genomes by self-training algorithm. Nucleic Acids Res. 33: 6494-6506.

Li L, Stoeckert Jr, and Roos S (2003). OrthoMCL: Identification of ortholog groups for eukaryotic genomes. Genome Res., 13: 2178-2189.

Li W, Godzik A (2006). Cd-hit: a fast program for clustering and comparing large sets of protein or nucleotide sequences. Bioinformatics 22: 1658-1659.

Miller JR, Delcher AL, Koren, S, Venter E, Walenz BP, Brownley A, Johnson J, et al. (2008). Aggressive assembly of pyrosequencing reads with mates. Bioinformatics 24: 2818-2824.

Myers EW, Sutton GG, Delcher AL, Dew IM, Fasulo DP, Flanigan MJ, Kravitz SA, et al. (2000). A whole-genome assembly of Drosophila. Science 287: 2196-2204.

Rane RV (2017) The genomic basis of climate and host adaptation. phD thesis, University of Melbourne.

Rane RV, Oakeshott JG, Nguyen T, Hoffmann AA, Lee SF (2017). Orthonome – a new pipeline for predicting high quality orthologue sets applicable to complete and draft genomes. BMC Genomics in press.

Stanke M, Diekhans M, Baertsch R, Haussler D (2008). Using native and syntenically mapped cDNA alignments to improve *de novo* gene finding. Bioinformatics, 24: 637-644.

Van Dongen S (2000). Graph clustering by flow simulation*.* Ph.D thesis, University of Utrecht, The Netherlands.

Walker BJ, Abeel T, Shea T, Priest M, Abouelliel A, Sakthikumar S, Cuomo CA, et al. (2014). Pilon: An integrated tool for comprehensive microbial variant detection and genome assembly improvement. PLoS ONE 9: e112963.
