# Supplementary figures and images for: Genomic innovations, transcriptional plasticity and gene loss underlying the evolution and divergence of two highly polyphagous and invasive Helicoverpa pest species
Source: BMC Biol. 2017 Jul 31;15:63. doi: 10.1186/s12915-017-0402-6 (PMC5535293; doi:10.1186/s12915-017-0402-6)

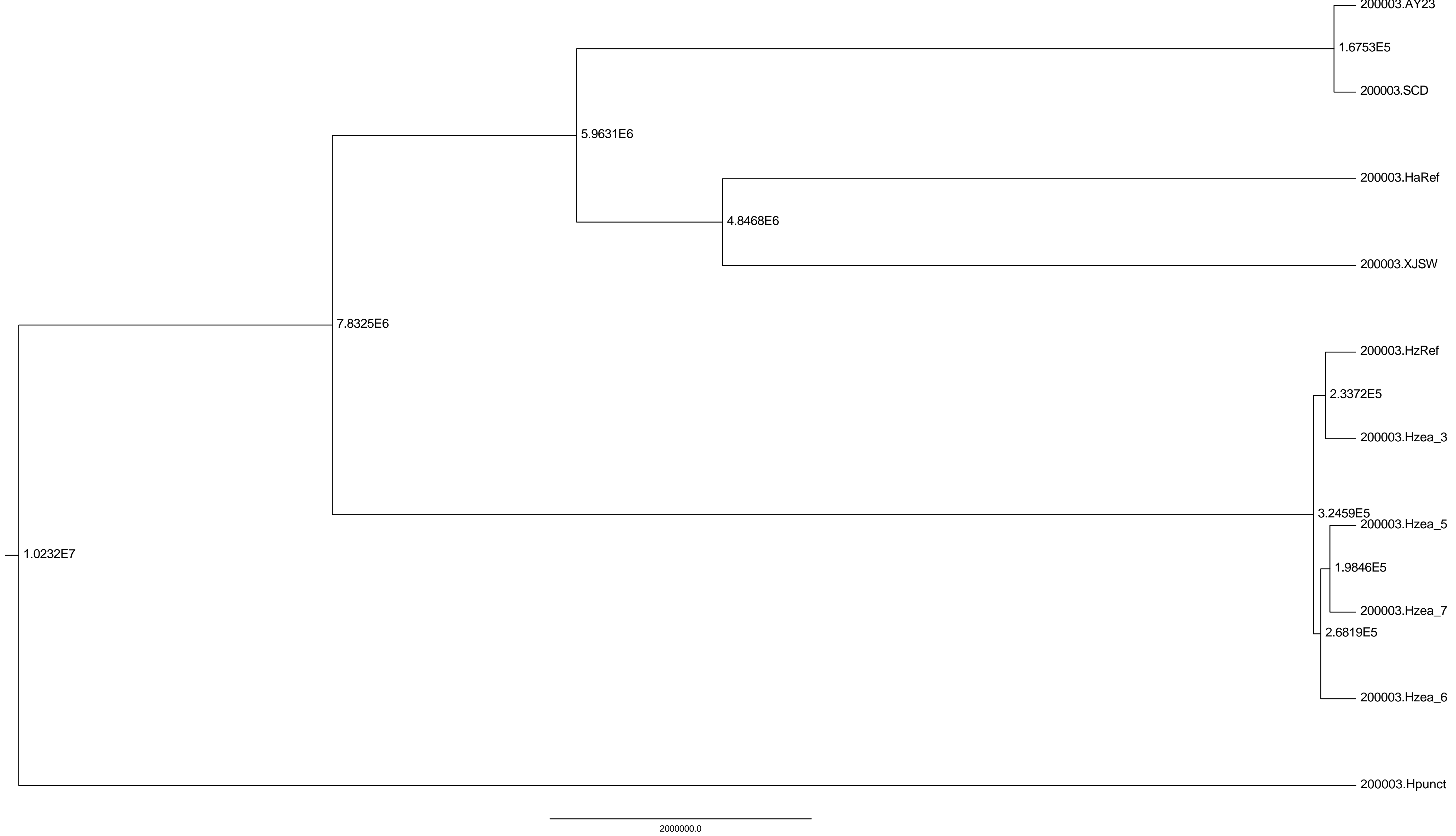

Supplement: Supplementary file 10 — List of 129 H. armigera transcription factors (TFs) mapped to D. melanogaster TFs in networks. (ZIP 19 kb) [file 12915_2017_402_MOESM10_ESM.zip › 200003.pdf]

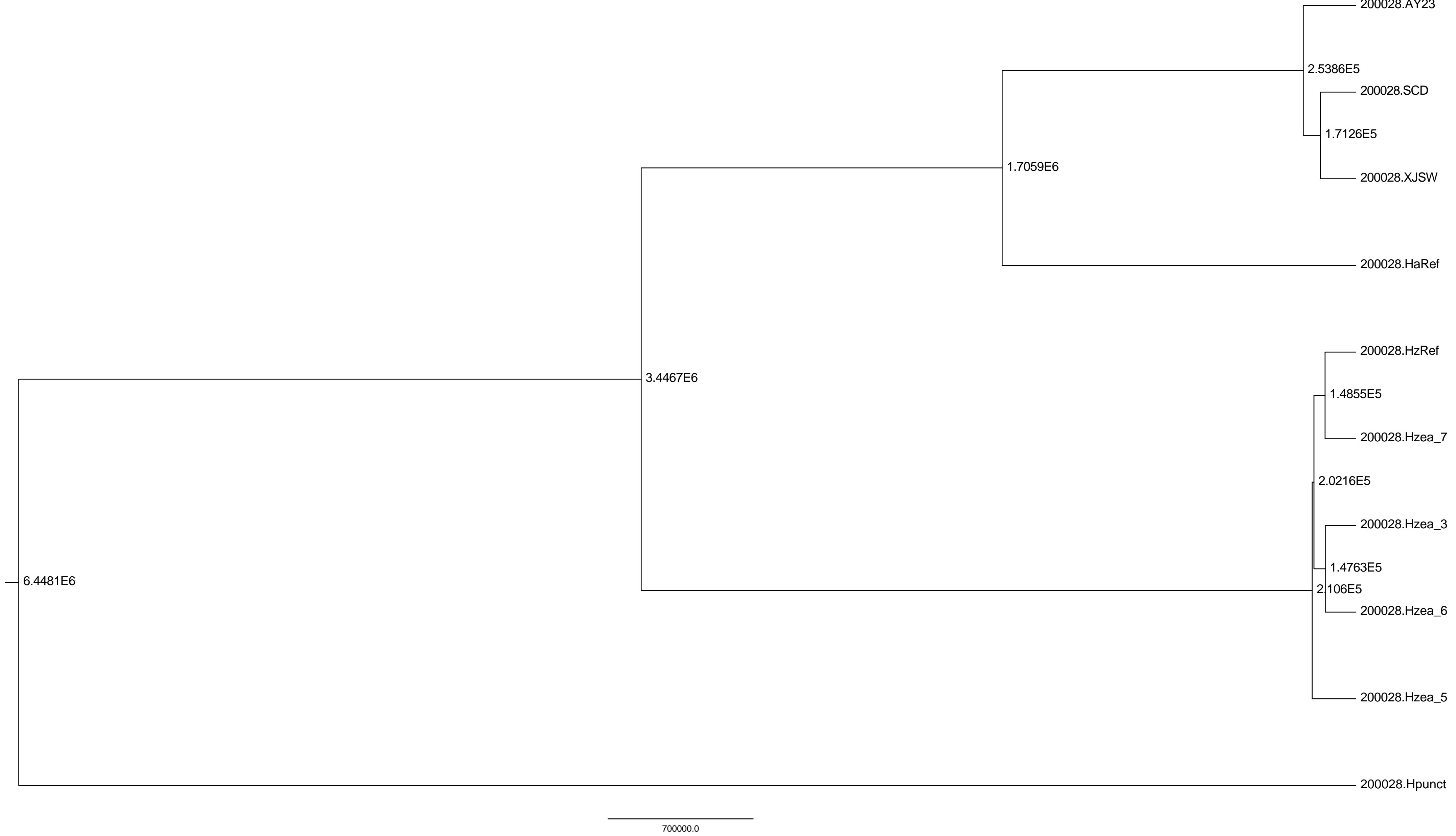

Supplement: Supplementary file 10 — List of 129 H. armigera transcription factors (TFs) mapped to D. melanogaster TFs in networks. (ZIP 19 kb) [file 12915_2017_402_MOESM10_ESM.zip › 200028.pdf]

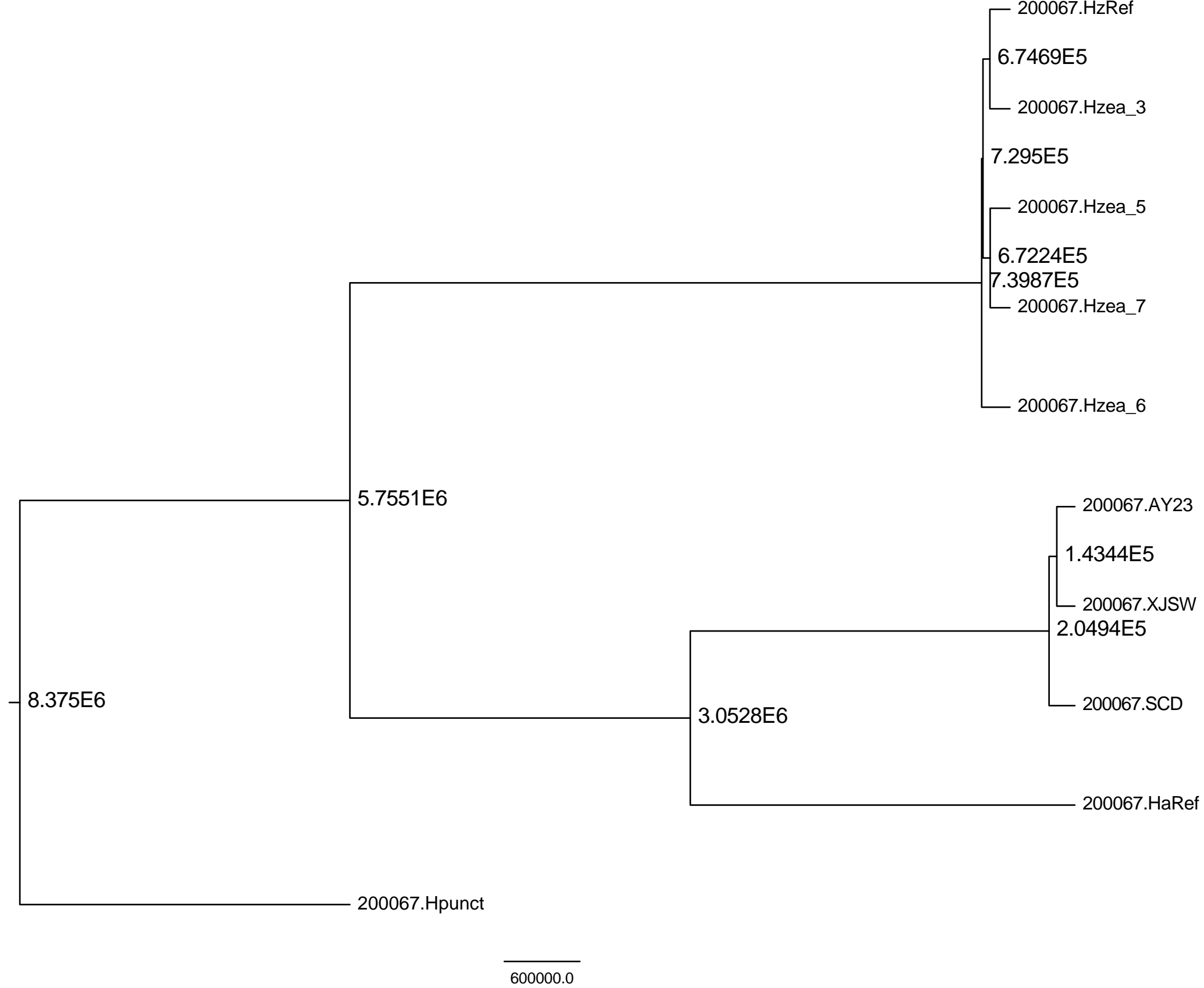

Supplement: Supplementary file 10 — List of 129 H. armigera transcription factors (TFs) mapped to D. melanogaster TFs in networks. (ZIP 19 kb) [file 12915_2017_402_MOESM10_ESM.zip › 200067.pdf]

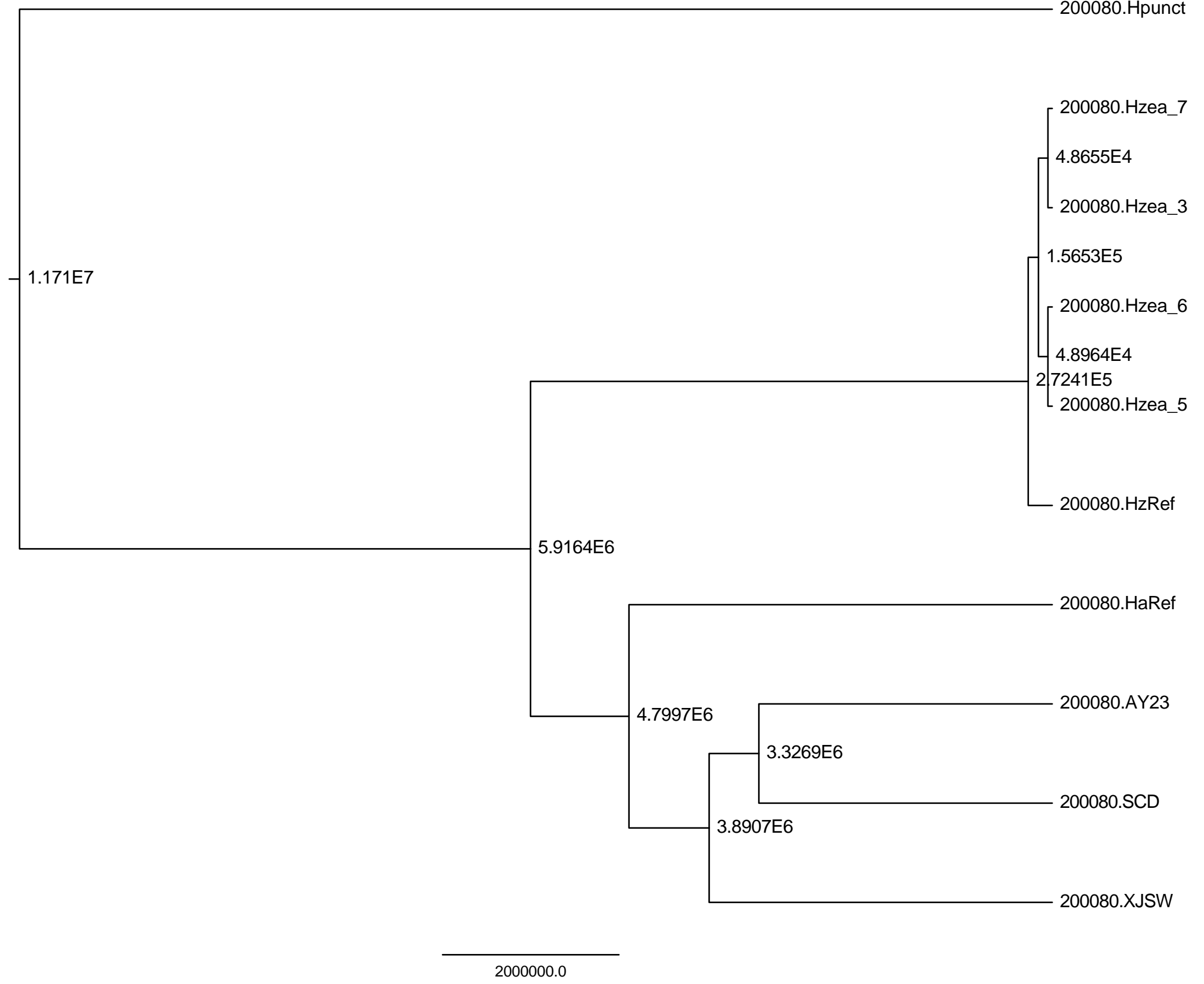

Supplement: Supplementary file 10 — List of 129 H. armigera transcription factors (TFs) mapped to D. melanogaster TFs in networks. (ZIP 19 kb) [file 12915_2017_402_MOESM10_ESM.zip › 200080.pdf]

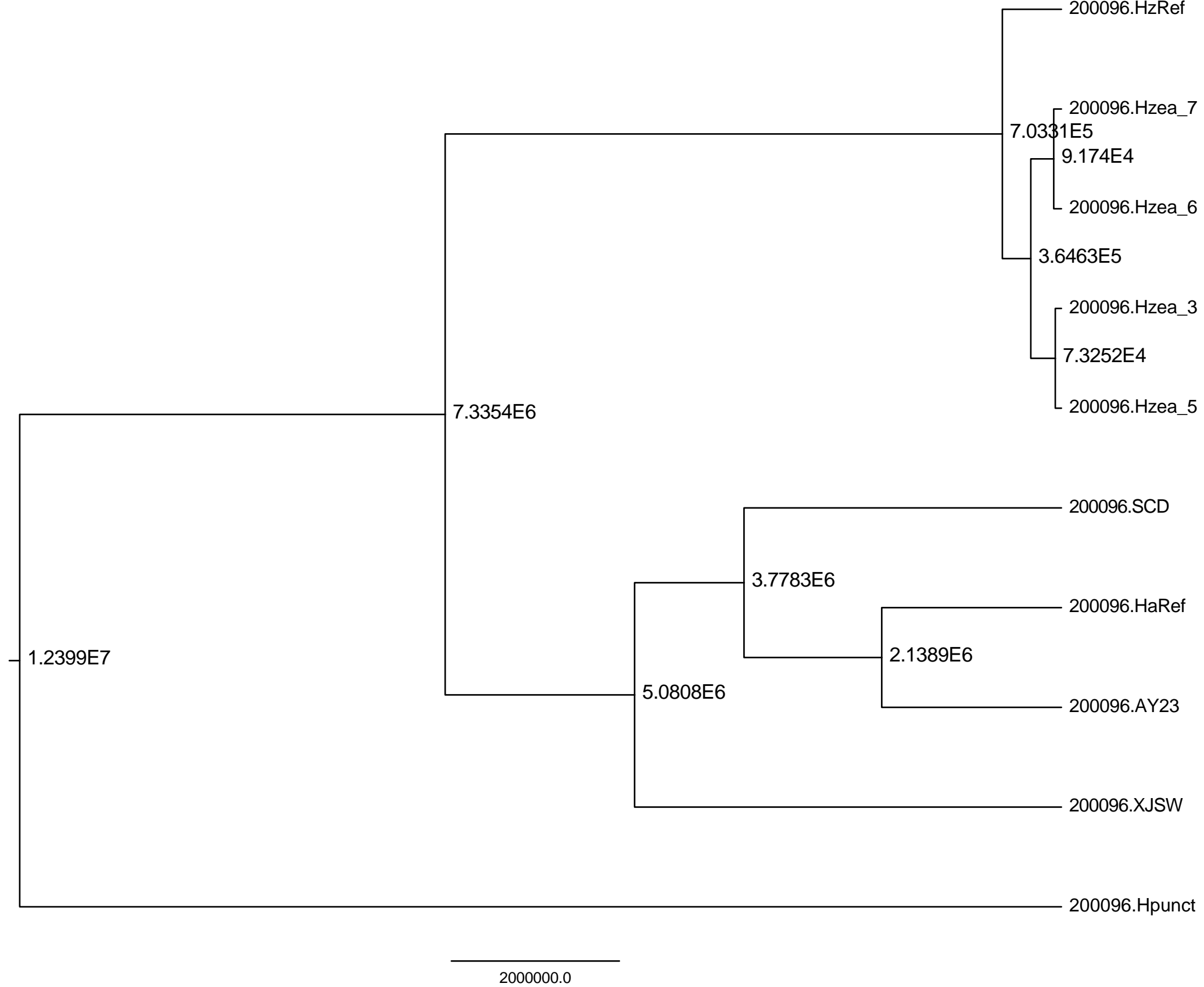

Supplement: Supplementary file 10 — List of 129 H. armigera transcription factors (TFs) mapped to D. melanogaster TFs in networks. (ZIP 19 kb) [file 12915_2017_402_MOESM10_ESM.zip › 200096.pdf]

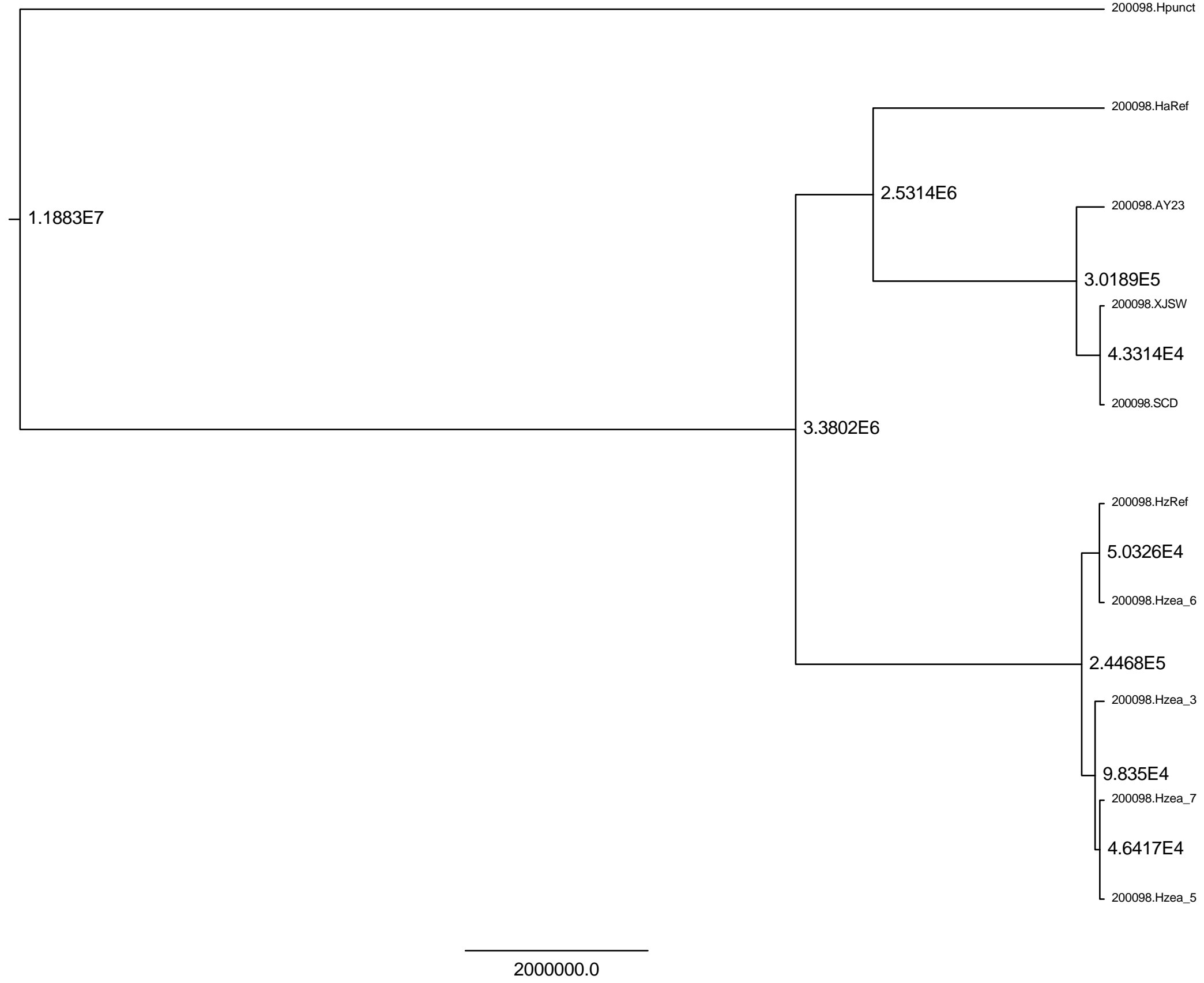

Supplement: Supplementary file 10 — List of 129 H. armigera transcription factors (TFs) mapped to D. melanogaster TFs in networks. (ZIP 19 kb) [file 12915_2017_402_MOESM10_ESM.zip › 200098.pdf]

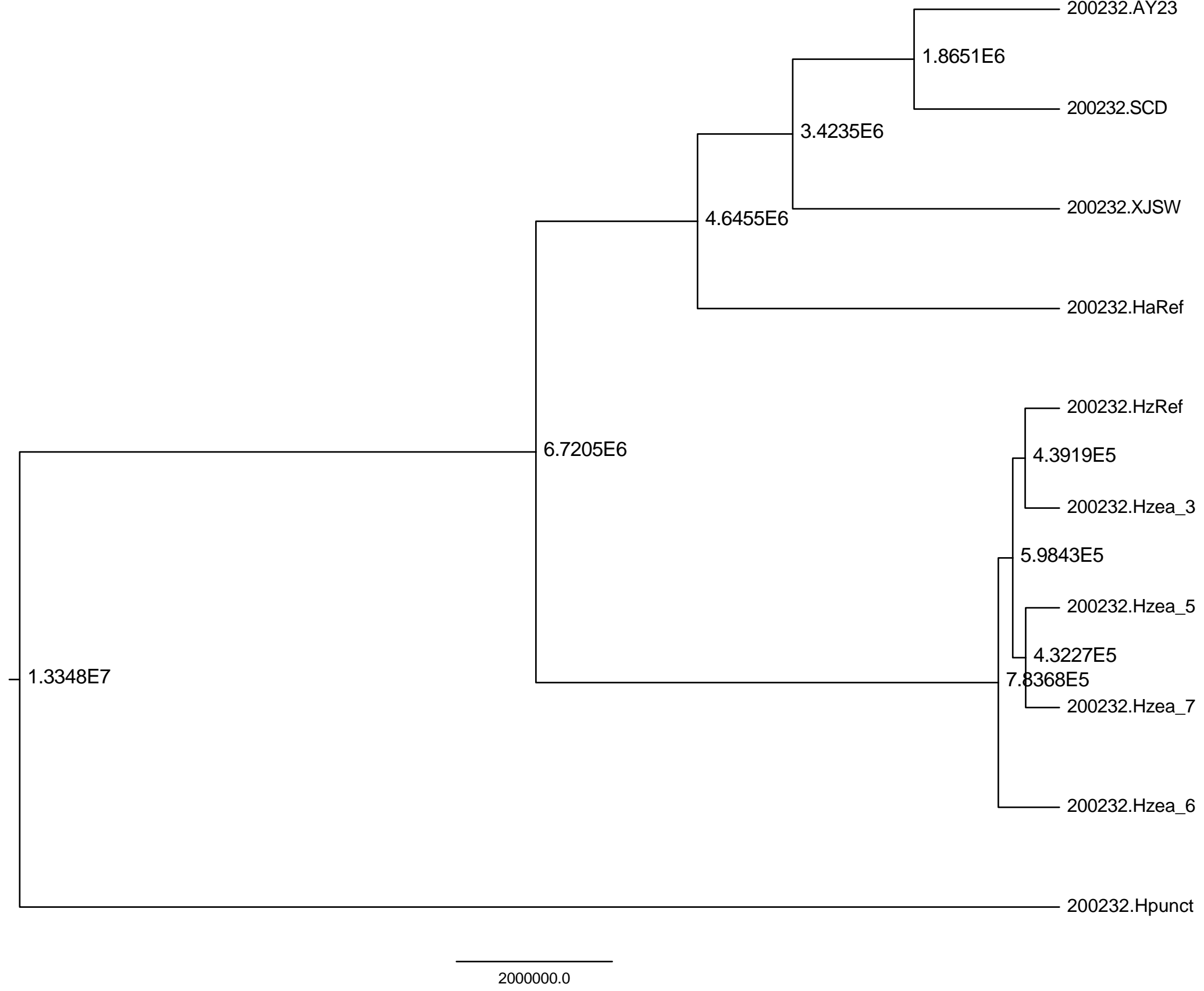

Supplement: Supplementary file 10 — List of 129 H. armigera transcription factors (TFs) mapped to D. melanogaster TFs in networks. (ZIP 19 kb) [file 12915_2017_402_MOESM10_ESM.zip › 200232.pdf]

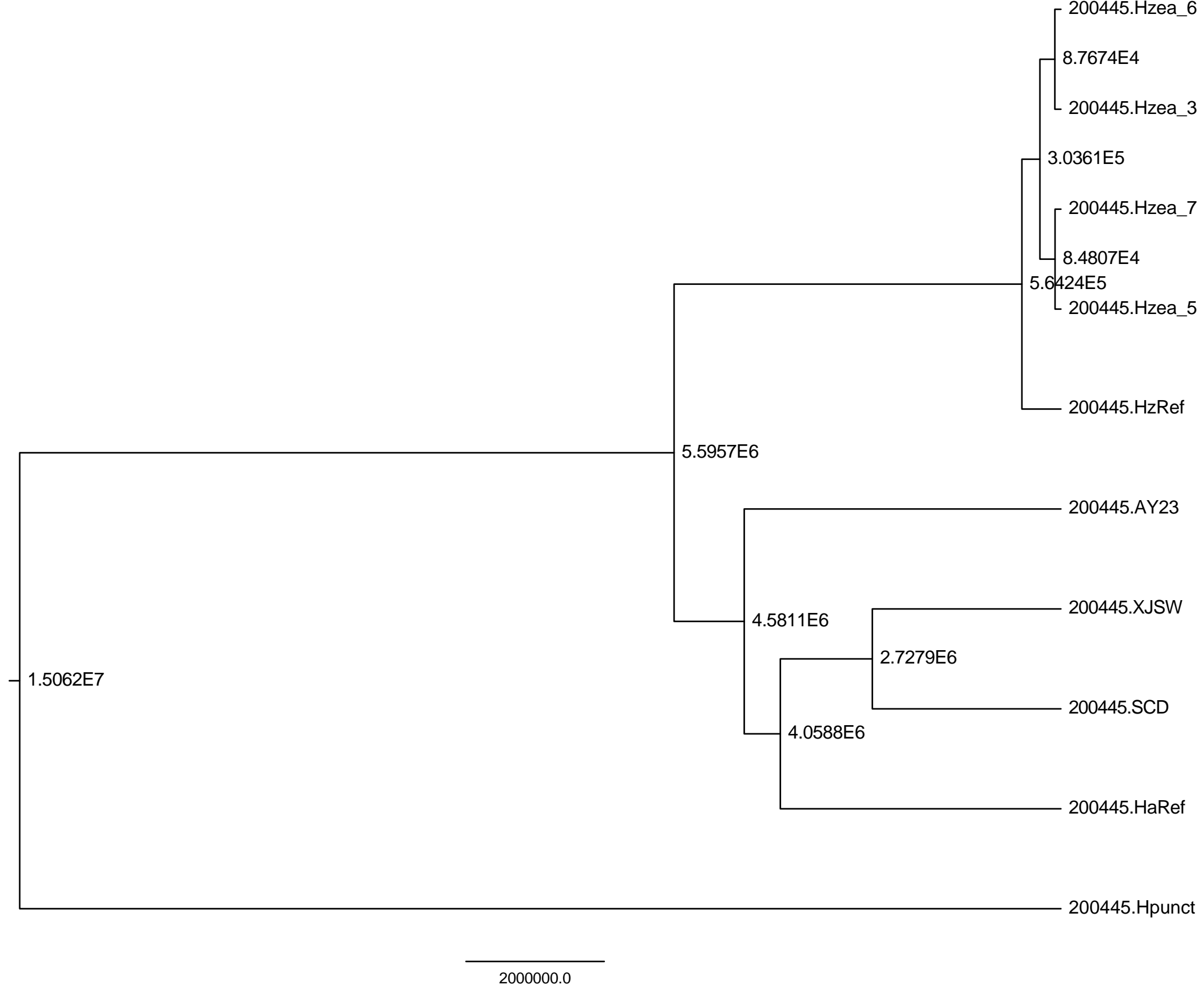

Supplement: Supplementary file 10 — List of 129 H. armigera transcription factors (TFs) mapped to D. melanogaster TFs in networks. (ZIP 19 kb) [file 12915_2017_402_MOESM10_ESM.zip › 200445.pdf]

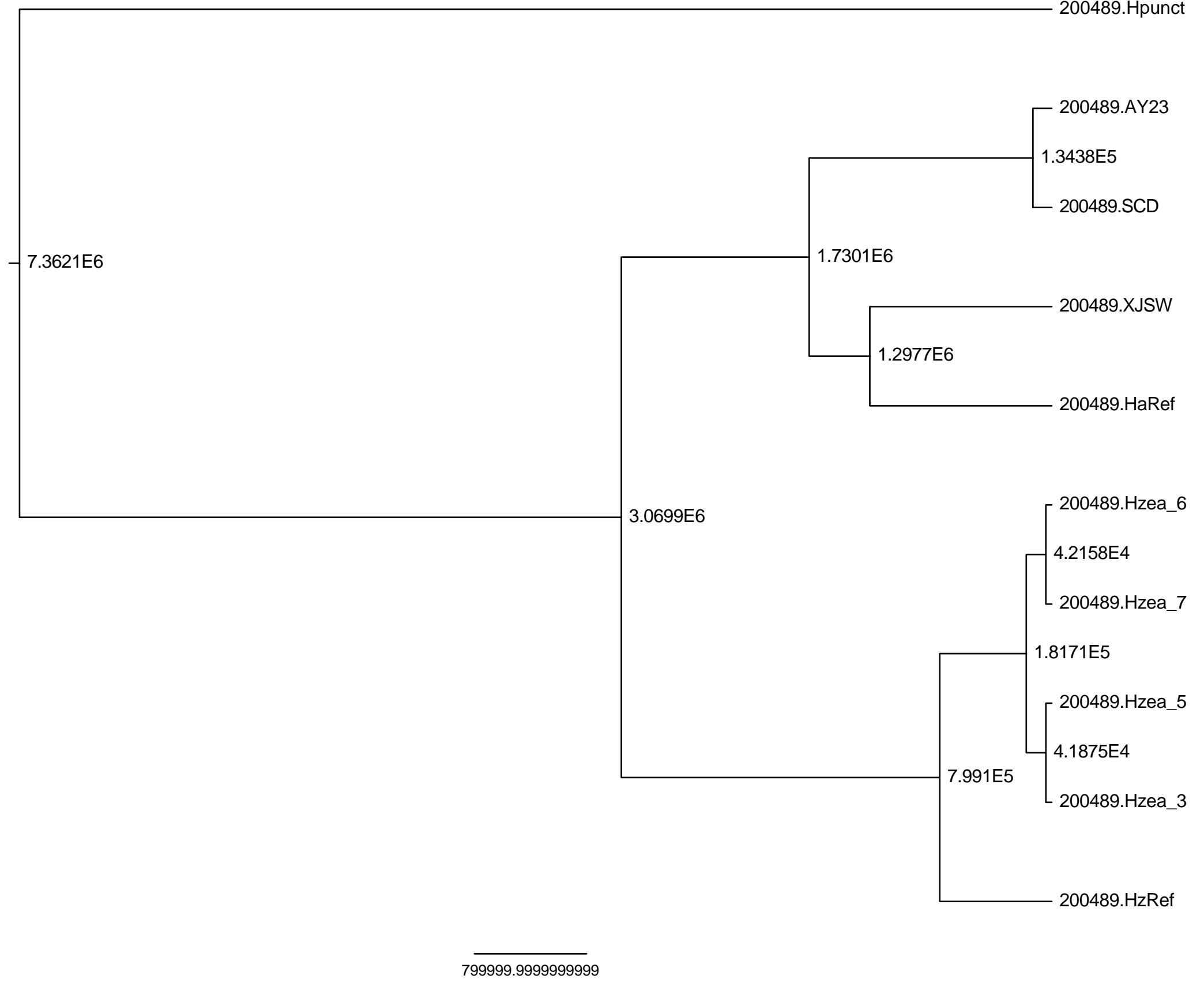

Supplement: Supplementary file 10 — List of 129 H. armigera transcription factors (TFs) mapped to D. melanogaster TFs in networks. (ZIP 19 kb) [file 12915_2017_402_MOESM10_ESM.zip › 200489.pdf]

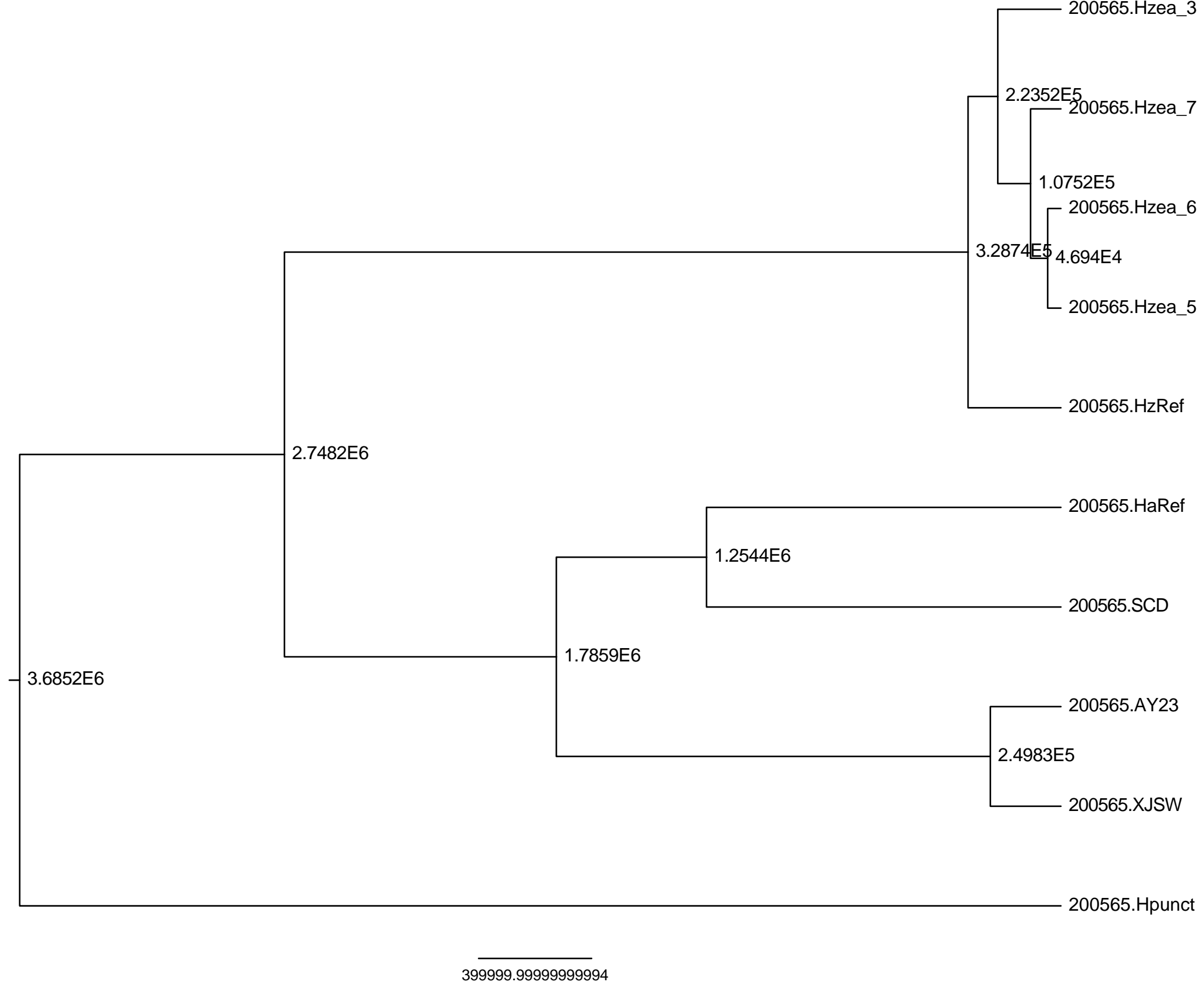

Supplement: Supplementary file 10 — List of 129 H. armigera transcription factors (TFs) mapped to D. melanogaster TFs in networks. (ZIP 19 kb) [file 12915_2017_402_MOESM10_ESM.zip › 200565.pdf]

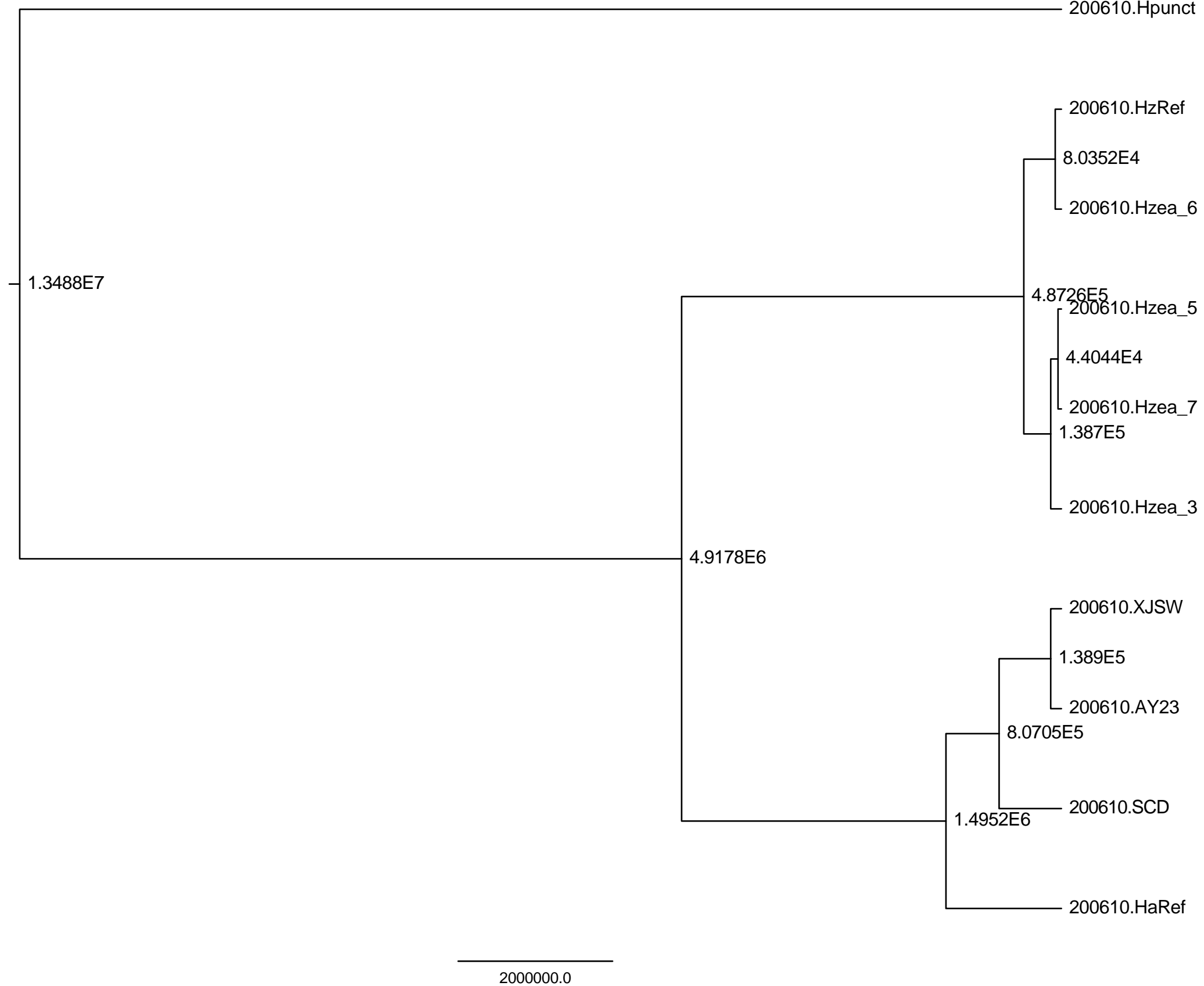

Supplement: Supplementary file 10 — List of 129 H. armigera transcription factors (TFs) mapped to D. melanogaster TFs in networks. (ZIP 19 kb) [file 12915_2017_402_MOESM10_ESM.zip › 200610.pdf]

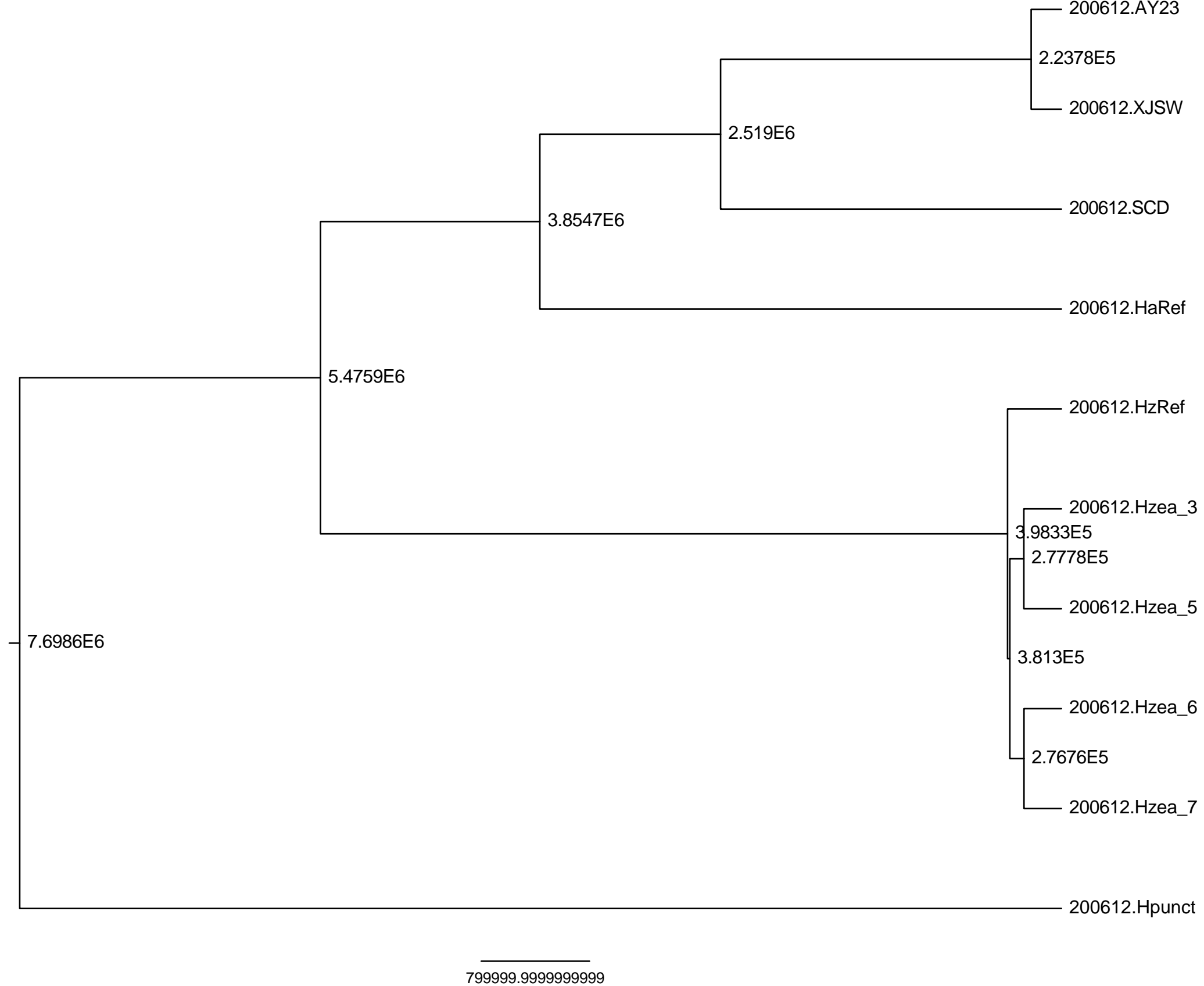

Supplement: Supplementary file 10 — List of 129 H. armigera transcription factors (TFs) mapped to D. melanogaster TFs in networks. (ZIP 19 kb) [file 12915_2017_402_MOESM10_ESM.zip › 200612.pdf]
